# Sulfamate Acetamides as Self-Immolative Electrophiles for Covalent Ligand-Directed Release Chemistry

**DOI:** 10.1021/jacs.2c08853

**Published:** 2023-02-04

**Authors:** Rambabu N. Reddi, Adi Rogel, Ronen Gabizon, Dattatraya Gautam Rawale, Battu Harish, Shir Marom, Barr Tivon, Yamit Shorer Arbel, Neta Gurwicz, Roni Oren, Keren David, Jingjing Liu, Shirly Duberstein, Maxim Itkin, Sergey Malitsky, Haim Barr, Ben-Zion Katz, Yair Herishanu, Idit Shachar, Ziv Shulman, Nir London

**Affiliations:** † Dept. of Chemical and Structural Biology, The Weizmann Institute of Science, Rehovot 7610001, Israel; ‡ Sackler Faculty of Medicine, Tel Aviv University, Tel-Aviv 6997801, Israel; § Dept. of Systems Immunology, The Weizmann Institute of Science, Rehovot 7610001, Israel; ∥ Department of Veterinary Resources, The Weizmann Institute of Science, Rehovot 7610001, Israel; ⊥ Wohl Institute for Drug Discovery of the Nancy and Stephen Grand Israel National Center for Personalized Medicine, The Weizmann Institute of Science, Rehovot 7610001, Israel; # Life Sciences Core Facilities, The Weizmann Institute of Science, Rehovot 7610001, Israel; ∇ Department of Hematology, Tel Aviv Sourasky Medical Center, Tel Aviv 6423906, Israel

## Abstract

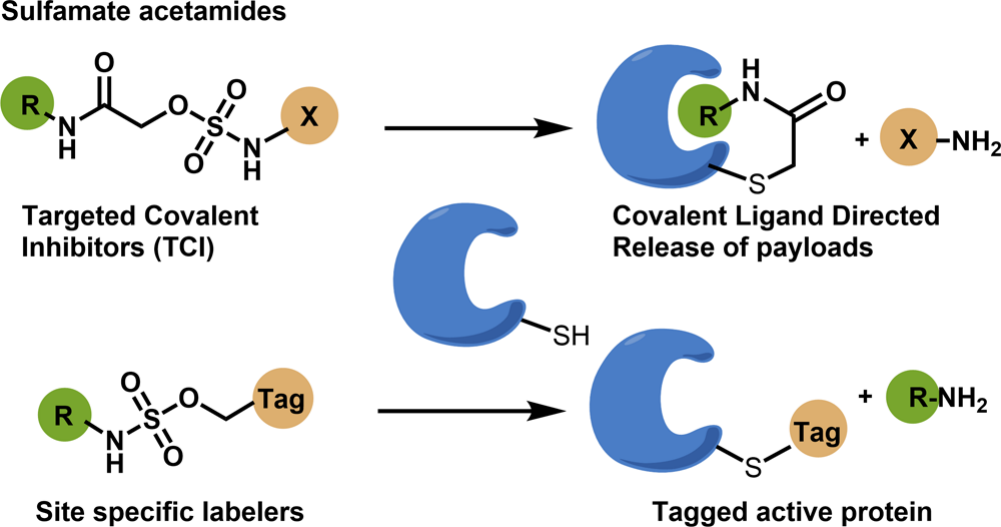

Electrophiles for covalent inhibitors that are suitable for in vivo administration are rare. While acrylamides are prevalent in FDA-approved covalent drugs, chloroacetamides are considered too reactive for such purposes. We report sulfamate-based electrophiles that maintain chloroacetamide-like geometry with tunable reactivity. In the context of the BTK inhibitor ibrutinib, sulfamate analogues showed low reactivity with comparable potency in protein labeling, in vitro, and cellular kinase activity assays and were effective in a mouse model of CLL. In a second example, we converted a chloroacetamide Pin1 inhibitor to a potent and selective sulfamate acetamide with improved buffer stability. Finally, we show that sulfamate acetamides can be used for covalent ligand-directed release (CoLDR) chemistry, both for the generation of “turn-on” probes as well as for traceless ligand-directed site-specific labeling of proteins. Taken together, this chemistry represents a promising addition to the list of electrophiles suitable for in vivo covalent targeting.

## Introduction

**Figure 1:**
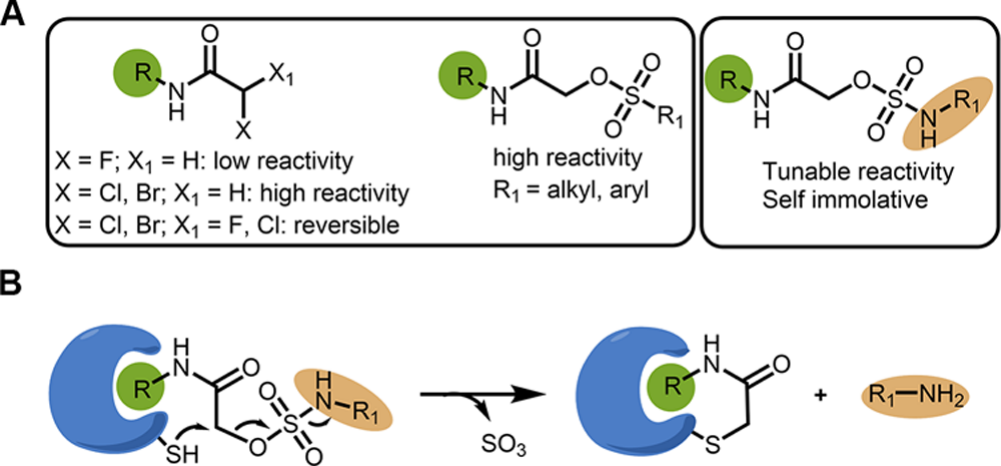
Sulfamate acetamides as electrophiles for targeted covalent inhibitors and CoLDR chemistry: (A) Reactivity pattern of α-substituted acetamides. (B) Schematic representation of the reaction of a target cysteine with α-sulfamate acetamides through CoLDR chemistry.

Electrophilic small molecules that are able to form covalent bonds with nucleophilic amino acids like cysteine, lysine, and tyrosine play a pivotal role in chemical biology.^1,2^ Such electrophiles have been successfully used in bioconjugation for the synthesis of antibody-drug conjugates,^3,4^ used as probes for chemoproteomics^5−8^ activity-based protein profiling (ABPP)^9,10^ and as covalent warheads in the design of targeted covalent inhibitors (TCIs).^11−13^ Highly reactive and residue-selective electrophiles are useful for bioconjugation^14−16^ and proteomics applications, while low reactivity and highly stable electrophiles are suitable for TCIs. Relatively few electrophiles meet the criteria to be used in TCIs. In spite of the therapeutic benefits of covalent inhibitors like enhanced and sustained pharmacological potency and protein isoform selectivity compared to their reversible counterparts, their potential toxicity due to the off-target reactivity is a key concern.^2,17^ Some of the most commonly used electrophiles in designing targeted covalent inhibitors are acrylamides and chloroacetamides, which react with cysteines (Figure S1).^18−21^ While acrylamide-based electrophiles are known to be able to achieve sufficiently low reactivity, chloroacetamides are more reactive^18,19^ as covalent “warheads”. This greatly limits their application in designing TCIs. Consequently, fluorochloro-acetamide,^22^ α-substituted chloroacetamide,^2,23^ and di- and tri-halo acetamide^24^ warheads have been reported as less reactive alternatives (Figure 1A). Although these warheads showed improved selectivity, it was typically at the cost of reduced potency. Tunability of the electrophile reactivity can help find the optimal balance between selectivity and potency. However, there are very few degrees of freedom with chloroacetamides. Herein, we report α-sulfamate acetamides as highly stable warheads with tunable reactivity and similar geometry to chloroacetamides (Figure 1A).

Several strategies were reported for the functionalization of covalent binders beyond just enzyme inhibition.^25^ In this context, covalent inhibitor-based fluorescent turn-on probes have been developed and used in protein profiling and sensing applicaitons.^26−28^ Hamachi et al. recently developed *N*-acyl-*N*-alkyl sulfonamide (NASA) electrophiles that have been used for site-selective labeling of a protein of interest (POI) while eliminating the recognition element.^29^ We have previously developed substituted methacrylamides as an electrophilic warhead, which enabled covalent ligand directed release (CoLDR). Using this chemistry, fluorescent or chemiluminescent payloads were released in their active form upon reacting with the target cysteine.^30^ Substituted methacrylamides also allowed the site-specific labeling of proteins in their active form.^31^ However, this chemistry is limited to acrylamide-based covalent inhibitors. The sulfamates we describe below allow us to expand the CoLDR chemistry concept to analogues of α-halo acetamide electrophiles.

Sulfamates (-O-SO_2_-NH-) are prevalent in medicinal chemistry and many bioactive and drug molecules contain this functionality.^32^ Sulfonate esters (-O-SO_2_-R), due to their highly electrophilic nature, have been used before as chemoproteomic probes,^33,34^ and site-selective labeling reagents of proteins in cells; however, they are promiscuous and potentially react with several amino acids.^35^ On the other hand, sulfamates, with a similar structure where the alkyl group of sulfonate is replaced by a nitrogen atom, were not explored neither as electrophilic warheads nor for chemical biology applications. We postulated that such sulfamate compounds can have varied reactivity based on the nature of the amine group and can potentially act as electrophilic warheads. Further, when these electrophiles react with cysteine, they release sulfamic acid, which will dissociate into sulfur trioxide and a free amine (Figure 1B).^36^ This “self-immolative” property could position them for use in covalent ligand-directed release chemistry. Hence, we explored sulfamate acetamide as an electrophilic warhead with varied reactivity. We have demonstrated the utility of these warheads in the context of covalent inhibitors of BTK (ibrutinib)^37^ and Pin1 (sulfopin).^38^ Since they release an amine functional group after the formation of a covalent bond with a target cysteine, we have used them to release a “payload” and developed a fluorescent turn-on probe for BTK. In the other direction, we demonstrated that sulfamates can be used for ligand-directed site-specific traceless labeling of BTK in its active form.

## Results

### Sulfamate Acetamides Are Tunable and Low-Reactivity Electrophiles

**Figure 2:**
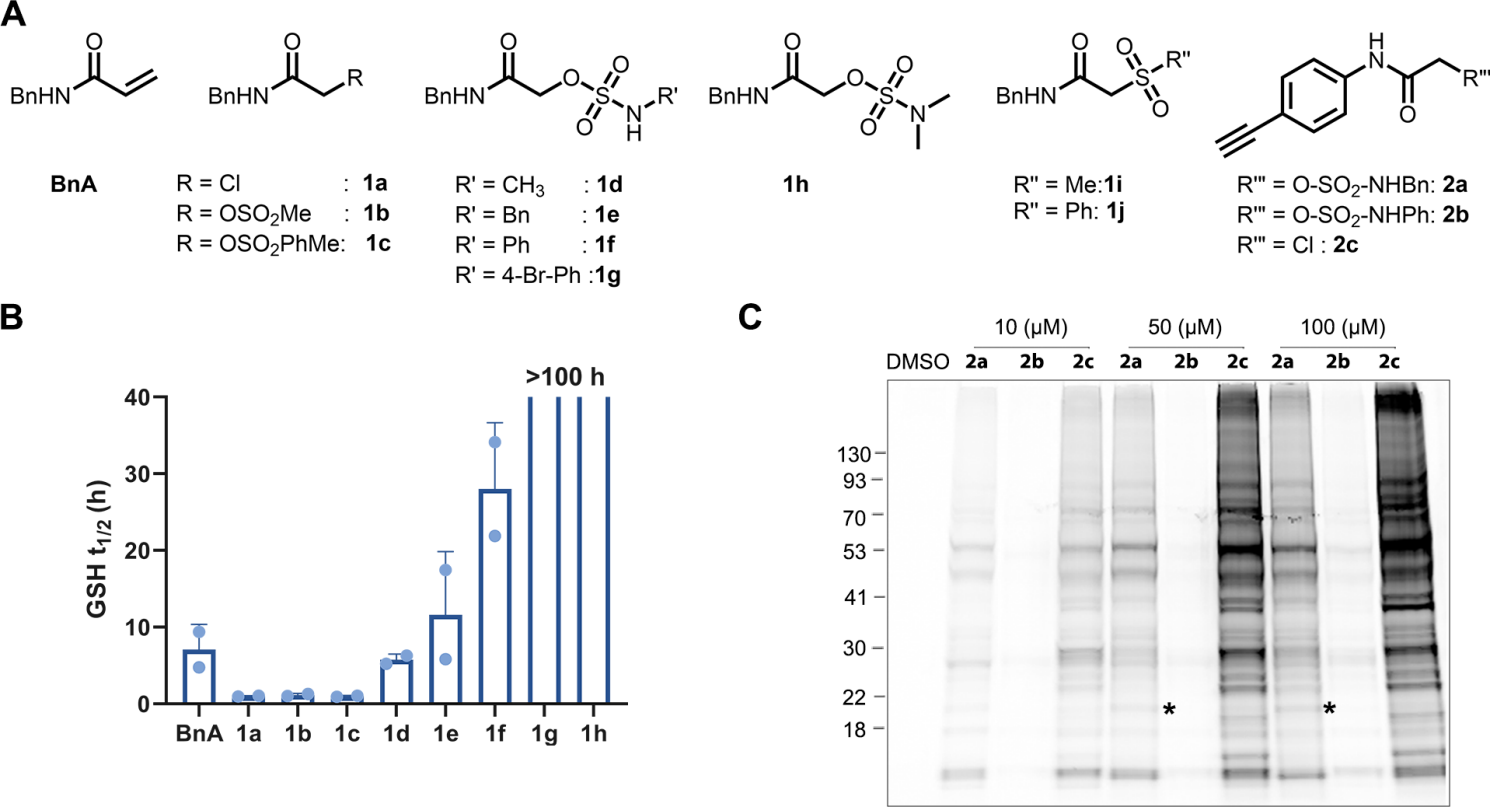
α-Sulfamate acetamides can show up to two orders of magnitude less reactivity towards GSH than chloroacetamide. (A) Chemical structures of model α-sulfamate/sulfonate/sulfone acetamides. (B) Half-life (*t*_1/2_) of the model compounds (**1a–1j**) assessed by GSH consumption assay via LC/MS (Figure S3B). (C). In situ proteomic labeling with alkyne probes (**2a**–**2c**). Mino cells were treated for 2 h with either DMSO or **2a**–**2c**, then lysed, reacted with TAMRA-azide using CuAAC, and imaged via in-gel fluorescence (532 nm). Bands that are selectively detected only by compound **2a** are indicated by asterisks.

We synthesized nine sulfo-based model electrophiles including two sulfonates (**1b** and **1c**) five sulfamates (**1d**–**1h**) and two sulfones (**1i** and **1j**; Figure 2A and Figure S2). We then conducted GSH consumption assays (5 mM GSH, 200 μM electrophile, pH 8, 14 °C; 4-nitro cyano-benzene was used as an internal standard) for all the sulfo compounds as well as benzyl acrylamide (**BnA**) and chloroacetamide (**1a**). A sample from the reaction mixture was injected to an LC/MS every hour, and we quantified the decrease in starting material over the course of the reaction. For example, the LC/MS chromatogram (at 220 nm) of sulfamate **1d** at *t* = 0 h and at *t* = 5 h shows an increase in GSH adduct and a decrease in starting material (Figure S3A). The sulfonate esters, mesyl (**1b**) and tosyl (**1c**) groups, showed similar reactivities to the chloroacetamide (**1a**) with a half-life of 50 min. On the other hand, methyl sulfamate (**1d**) and benzyl sulfamate (**1e**) showed an order of magnitude less reactivity than chloroacetamide (**1a**) with equal or lower reactivity to that of the unsubstituted acrylamide (**BnA**; Figure 2B).

Surprisingly, phenyl sulfamate (**1f**; *t*_1/2_ = ∼25 h) and 4-bromo phenyl sulfamate (**1g**; *t*_1/2_ > 100 h) exhibit much lower reactivity, possibly because the release of electrons from the amine to the sulfur increases conjugation and stabilization (Figure S3E). Finally, dimethyl sulfamate (**1h**) also did not react under these reaction conditions (*t*_1/2_ > 100 h). α-Sulfone acetamides (**1i** and **1j**) did not form a covalent bond with GSH under the assay conditions or even at temperatures up to 37 °C for 24 h (Figure S4). We have also assessed the thiol reactivities of these model compounds using a DTNB reactivity assay^19^ (Figure S3C), which showed similar results to the GSH consumption assay (Figure 2B and Figure S3D).

To understand the selectivity of these electrophiles toward cysteine over other nucleophilic amino acids, we have reacted glutamic acid and lysine with five model compounds (**BnA**, **1a**, **1b**, **1d**, and **1e**) under stringent reaction conditions (pH 8, 37 °C; 4 days). Under these conditions, glutamic acid did not react with any of these compounds (Figure S5), whereas lysine reacted with **BnA**, **1a**, and **1b** (forming 15, 12, and 9%, respectively, of the corresponding lysine adduct). All three sulfamates formed <5% product, suggesting that these compounds are more selective toward cysteine than lysine (Figure S6). Moreover, these sulfamate acetamides showed high buffer stability and did not undergo hydrolysis (<5%) after 2 days, whereas chloroacetamide **1a** hydrolyzed (12%) as well as sulfonate acetamides **1b** and **1c** (26 and 50%, respectively; Figure S7).

### Sulfamate Acetamides Show Attenuated and Tunable Proteomic Reactivity

To assess proteomic selectivity in cells, we have synthesized two sulfamate acetamides (**2a** and **2b**) and a chloroacetamide (**2c**) with an alkyne functionality (Figure 2A and Figure S8). We treated Mino cells for two hours with either DMSO or **2a**–**2c**. We then lysed the cells, labeled the alkynes via copper-catalyzed “click chemistry” (CuAAC) with TAMRA-azide, and imaged the adducts via in-gel fluorescence (Figure 2C). Similar to their reactivity pattern in the GSH reactivity assay, these compounds labeled various amounts of proteins under cellular conditions. The most reactive chloroacetamide labeled the highest number of proteins, followed by benzyl sulfamate acetamide and phenyl sulfamate acetamide. Interestingly, there is at least one example in which sulfamate **2a** labeled a distinct protein that was not labeled by the chloroacetamide, potentially due to having extra recognition mediated through the sulfamate.

### Sulfamate Acetamides Are Suitable for Late-Stage Optimization of Covalent Inhibitors

**Figure 3:**
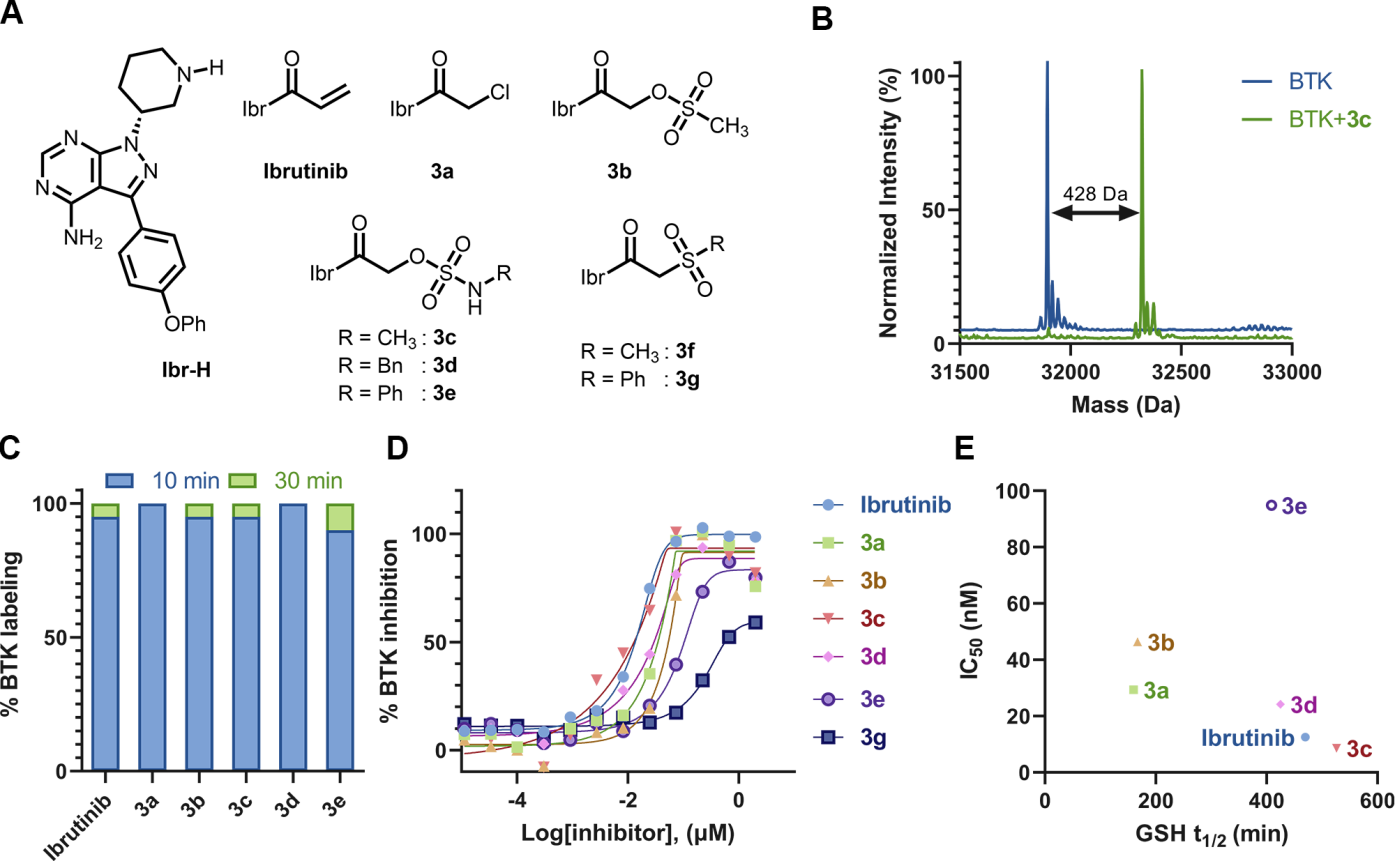
Ibrutinib sulfamates as potent BTK inhibitors: (A) Chemical structures of ibrutinib, **3a**–**3g.** (B) Deconvoluted LC/MS spectrum of BTK (2 μM) incubated with **3c** (2 μM) at pH 8, 25 °C, 30 min. The adduct mass corresponds to a labeling event in which methyl sulfamic acid was released, validating the proposed mechanism. (C) % of labeling of BTK (2 μM) with the probes (**3a**–**3e**; 2 μM) at 10 min (blue bar) and 30 min (green bar) in 20 mM Tris buffer at pH 8, 25 °C. (D) In vitro kinase activity assay (0.5 nM BTK, 5 μM ATP) for **3a**–**3g** (see Figure S11 for IC_50_ values). (E) Correlation of GSH half-life (*t*_1/2_) of ibrutinib sulfamates with measured IC_50_s in a kinase inhibition assay.

To show that sulfamates can work as electrophilic warheads in targeted covalent inhibitors, we have chosen ibrutinib, an acrylamide-based covalent inhibitor for Bruton’s tyrosine kinase (BTK), and replaced its acrylamide electrophile with sulfamate acetamides. Ibrutinib is an FDA-approved drug for B-cell malignancies and inhibits BTK phosphorylation by forming an irreversible bond at Cys481.^37,39^ The synthesis of ibrutinib-based sulfamate acetamides (Figure 3A; **3c**–**3e**) is straightforward, starting by coupling the amine precursor Ibr-H with hydroxy acetic acid followed by a reaction with various sulfamoyl chlorides (Figure S9). In addition to sulfamates, we have also synthesized chloro- (**3a**), sulfonate- (**3b**), and sulfone (**3f** and **3g**) acetamide analogues of ibrutinib (Figures S9).

To assess their covalent labeling efficiency, we have conducted intact protein mass spectrometry experiments with recombinant BTK (2 μM) and compounds **3a**–**3g** (2 μM, buffer: 20 mM Tris, 50 mM NaCl, pH 8; 25 °C). All three sulfamate- (**3c**–**3e**) and sulfonate- (**3b**) acetamides labeled BTK by more than 95% within 10 min with the elimination of sulfamic acid or sulfonic acid leaving groups (Figure 3B,C and Figure S10). The labeling efficiency of these compounds is similar to ibrutinib and the chloroacetamide **3a**. The two sulfone acetamides (**3f** and **3g**) failed to react covalently with BTK under the reaction conditions.

To understand the potential of these compounds as BTK inhibitors, we conducted in vitro kinase activity assays for all ibrutinib derivatives against BTK (Figure 3D). The alkyl sulfamate compounds (**3c** and **3d**) showed similar IC_50_ to ibrutinib (around 10 nM; Figure 3D,E and Figure S11), whereas the phenyl sulfamate (**3e**) showed a 10-fold weaker IC_50_ (100 nM). **3a** and **3b** also showed potent BTK inhibition. The presumably non-covalent sulfone compound (**3g**) shows poor inhibition of BTK with IC_50_ = 0.5 μM (Figure S11). We should note that in previous studies,^30,31^ ibrutinib’s IC_50_ was much lower (<1 nM). The current result likely reflects slightly different assay conditions; however, the relative ranking of the various analogues is the important factor. To understand the importance of covalent bond formation and off-target selectivity, we conducted the same assay with ibrutinib, **3a**, **3c**, **3d**, and **3e** against BTK C481S mutant and EGFR, a therapeutically relevant off-target of ibrutinib. Compounds **3a**, **3c**, and **3d** lost 30- to 85-fold potency against the C481S mutant (Figure S11), indicating their dependence on covalent bond formation, while the chloroacetamide **3a** showed only ∼10-fold selectivity against EGFR, the sulfamates showed 20- to 30-fold selectivity.

To assess the thiol reactivity of these analogues, we performed a GSH consumption assay and found that these compounds follow a similar reactivity pattern to the model compounds. The sulfamate compounds (**3c–3e**) showed GSH half-lives (*t*_1/2_ ≈ 8 h) similar to ibrutinib (Figure 3E and Figure S12A and S13). On the other hand, **3a** and **3b** showed 2.5-fold higher reactivity than Ibr-sulfamates (*t*_1/2_ ≈ 3 h; Figure 3E and Figure S12B). When correlating the reactivity of these compounds with their kinase activities, we found that the sulfamate compounds **3c** and **3d** are potent inhibitors with relatively low thiol reactivity. Further, we have also found that these sulfamate electrophiles show high buffer stability (<5% hydrolysis) compared to chloro- (25% hydrolysis) and sulfonate (75% hydrolysis) electrophiles (37 °C; 4 days; Figure S14). Moreover, the sulfamate analogues displayed improved metabolic stability when incubated with human liver microsomes (Figure S15). In particular, over 30% of methyl sulfamate (**3c**) remained intact after a 5 min incubation, whereas ibrutinib was completely degraded (<5%).

### Sulfamate Acetamides Are Compatible with Cells and In Vivo Administration

**Figure 4:**
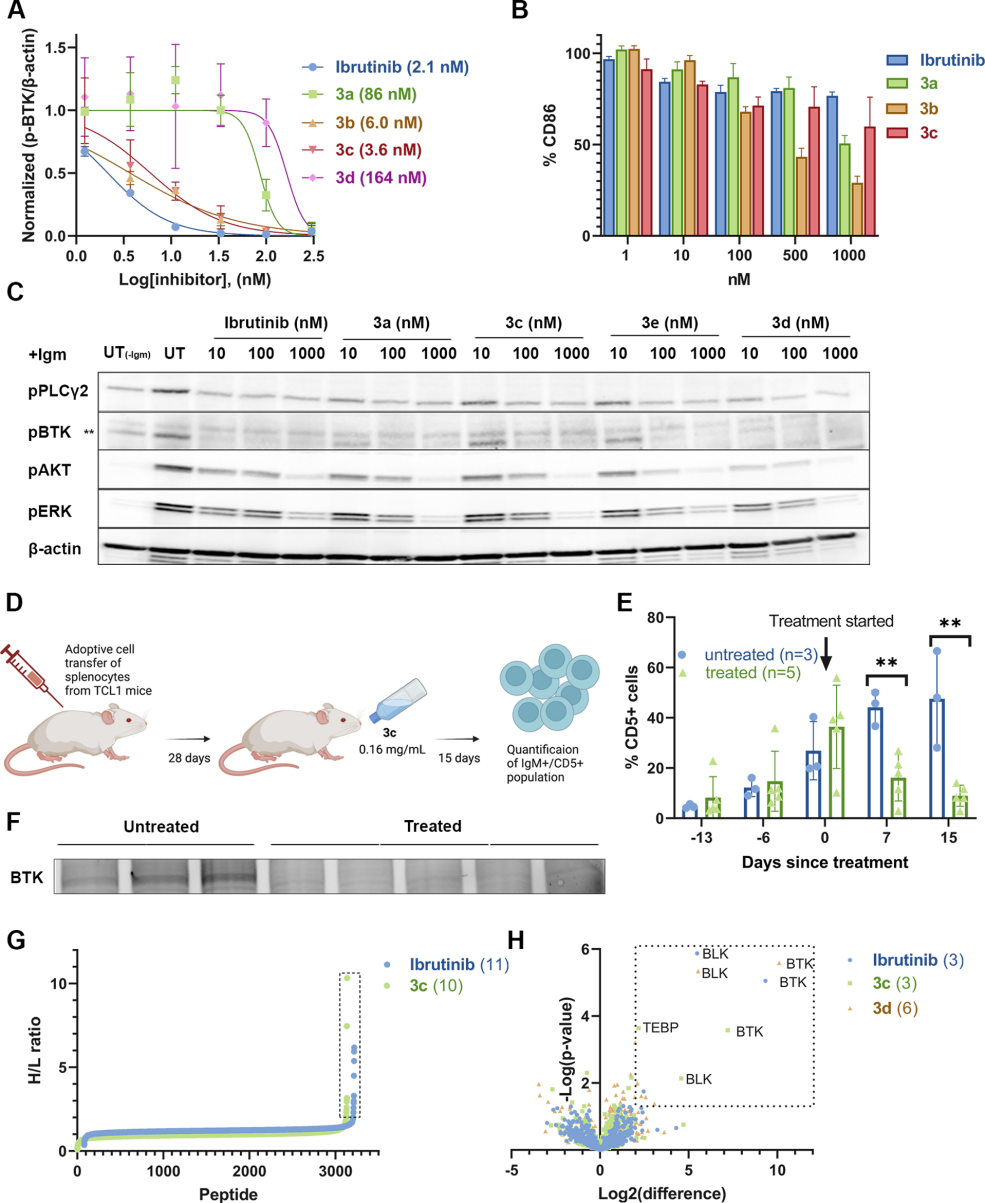
Ibrutinib sulfamate acetamide analogues are highly potent in cells and in vivo. (A) Dose-dependent BTK activity assay in Mino cells as measured by autophosphorylation of BTK. The cells were incubated for 2 h with either 0.1% DMSO, various concentrations of ibrutinib, or **3a**–**3d**. The cells were activated with anti-IgM, and BTK autophosphorylation was quantified by Western blot and normalized with respect to β-actin. IC_50_s were calculated by fitting the data to a dose–response curve using Prism software. (B) Dose-dependent inhibition of B-cell response (as measured by CD86 expression) after anti-IgM-induced activation and treatment with ibrutinib analogues (**3a–3d**) for 24 h (*n* = 3; error bars indicate standard deviation). (C) Dose-dependent inhibition of pBTK and its downstream pathways (pPLCγ2 , pAkt, and pERK) by ibrutinib derivatives (**3a**, **3c**, **3d**, and **3e**) in CLL patient samples. CLL cells (20 × 10^6^/mL) were incubated with ibrutinib or ibrutinib-based compounds at the indicated doses at 37 °C. DMSO-treated cells served as controls. After 2 h of incubation, the cells were either stimulated with goat F(ab′)2 anti-human IgM (10 μg/mL) for 15 min or left untreated. Proteins were then extracted and subjected to Western blot analysis. (D) Schematic representation of the in vivo mice experiment. Cells isolated from old TCL1 mice spleens, with a malignant cell population higher than 60%, were injected into the tail vein of 6 week old recipient mice. The mice were given a solution containing sulfamate **3c** (0.16 mg/mL in 1% cyclodextrin water) ad libitum in drinking water*.* Progression of the disease was followed in the peripheral blood (PB) by using flow cytometry for quantification of the IgM+/CD5+ population (created with BioRender.com). (E) IgM+/CD5+ cell population is significantly lowered in **3c**-treated mice (*n* = 5) compared to untreated (*n* = 3). **p = 0.002 for days 7 and 15 (single-tailed Student’s *t* test) (F) BTK engagement of compound **3c** in vivo. Dissected spleens were extracted with RIPA buffer and incubated with an ibrutinib alkyne analogue (probe-4)^40^ for 1 h followed by CuAAC reaction with TAMRA-azide in lysate before imaging. (G) IsoDTB-ABPP experiment with ibrutinib and sulfamate **3c**. Mino cells were treated with 1 μM of either ibrutinib or **3c** for 2 h, followed by incubation of iodoacetamide alkyne and CuAAC click reaction with heavy/light isoDTB tags. The labeled peptides were pulled down with streptavidin beads and quantified via LC/MS/MS (Figure S21; *n* = 4). Proteins in the box have a heavy-to-light (H/L) ratio of ≥ 2. Only peptides detected in at least three out of four repetitions are presented (Dataset S1). (H) Selectivity of ibrutinib, **3c**, and **3d** quantified via a competitive pull-down proteomics experiment. Mino cells are treated with 1 μM compound for 1 h and 10 μM ibrutinib alkyne for an additional 1 h (*n* = 4). Proteins were quantified using label-free quantification. Proteins in the box show a significant change (Fold change > 4; *p* < 0.05).

To assess the cellular efficacy of these compounds, we followed the inhibition of BTK autophosphorylation in Mino cells. After 1 h of pre-incubation with the inhibitors and B-cell activation with an anti-IgM antibody, BTK autophosphorylation was followed by Western blot. All of the compounds completely inhibited phosphorylation at 100 nM except **3g** (Figure S16). The dose-dependent treatment of compounds **3c** (IC_50_ = 3.6 nM) and **3b** (IC_50_ = 6 nM) showed excellent inhibition, similar to ibrutinib (IC_50_ = 2.1 nM; Figure 4A and Figure S17). The structurally similar and more reactive analogue **3a** showed 20-fold less potency in cellular pBTK inhibition (IC_50_ = 86 nM), potentially due to reaction with off-targets.

We evaluated B-cell receptor signaling inhibition in primary mouse B-cells by ibrutinib as well as four of its analogues. Mouse splenocytes were incubated (24 h; 37 °C) with the inhibitors at various concentrations and treated with anti-IgM. To examine the effect specifically on B-cells, we gated on B220+ cells and assessed activation by flow cytometry detection of CD86 expression. All five derivatives (**3a**–**3e**) showed similar inhibition of B-cell activation to ibrutinib (Figure 4B and Figure S18).

To assess the efficiency of these new inhibitors in a clinically relevant model, we tested the potency of the three sulfamate analogues (**3c**–**3e**) along with ibrutinib and **3a** in chronic lymphocytic leukemia (CLL) primary patient samples for the inhibition of BTK phosphorylation and its downstream pathway targets pPLCγ2, pAkt, and pERK.^41^ CLL cells (20 × 10^6^/mL) were incubated with DMSO, ibrutinib, or ibrutinib-based sulfamates (**3c**–**3e**) at the indicated doses at 37 °C. After 2 h of incubation, the cells were stimulated for 15 min or left untreated. Cell lysates were extracted and analyzed by Western blot. All of the compounds inhibited p-BTK (>80%) at 100 and 1000 nM concentrations (Figure 4C). Downstream targets p-PLCγ2, p-Akt, and p-ERK were also dose-dependently downregulated; **3c**, **3d**, as well as **3a** showed p-ERK and p-Akt inhibition at 100 nM and 1 μM similar to ibrutinib (Figure 4C and Figure S19).

Taken together, these three cellular experiments indicate that sulfamate acetamides show target engagement in cells, stability to cellular conditions, and comparable potency to ibrutinib (Figure 4A–C).

Encouraged by the cellular results, we proceeded to evaluate the effect of the ibrutinib sulfamates in vivo. We tested compound **3c** in the TCL1 adoptive transfer mouse model for CLL.^42^ In this experiment, immune-competent healthy mice received an adoptive transfer of 4 × 10^7^ TCL1 splenocytes obtained from full leukemic TCL1 transgenic mice by intravenous injection (Figure 4D). IgM+/CD5+ cells were monitored weekly, and treatment (or mock treatment) was started 4 weeks after transplantation when CD5+ were > 30% in the blood. The mice received a solution containing sulfamate **3c** (0.16 mg/mL in 1% cyclodextrin water) ad libitum in the drinking water. The CD5+ cell count significantly decreased following treatment with **3c** compared to untreated mice in which the cell count increased (*p* = 0.002; Figure 4E and Figure S20A). Moreover, spleens were isolated from the mice after 2 weeks of treatment and quantified. The spleens isolated from treated mice were visually smaller than untreated mice (Figure S20B). To evaluate BTK engagement of the probe in vivo, the dissected spleens were extracted with RIPA buffer and incubated with an ibrutinib alkyne analogue,^40^ followed by the click reaction with TAMRA-azide, and imaged via gel fluorescence (Figure 4F and Figure S20C). The three untreated mice showed a prominent BTK band, while **3c**-treated mice do not show probe labeling, which confirms engagement of BTK by **3c**. These results suggest that the sulfamate acetamide electrophile is compatible with in vivo administration and shows oral bioavailability, sufficient exposure and in this model, and a pronounced therapeutic effect.

In order to check the cellular selectivity and identify potential off-targets, we performed isoDTB ABPP^5,43^ experiments with ibrutinib and compound **3c** (Figure 4G and Figure S21). In this experiment, the compounds showed similar proteomic selectivity. We detected only 11 and 10 off-targets for ibrutinib and **3c**, respectively (H/L ratio > 2; Dataset S1), out of which five proteins were shared between the two compounds. However, we could not identify BTK in this experiment. In order to identify BTK and other relevant kinase off-targets, we performed a competitive pull-down experiment (Figure 4H and Dataset S2), in which we first incubated Mino cells with either ibrutinib, **3c**, **3d** (1 μM), or DMSO control, followed by incubation with an ibrutinib alkyne probe^40^ (10 μM). This was followed by a reaction with biotin-azide using CuAAC, enrichment of the labeled proteins, and their quantification by tryptic digestion and LC/MS/MS analysis. This experiment identified BTK as the most prominent target for all three compounds. BLK was also identified as a known off-target. Overall, with a threshold of fold-enrichment of >4 and *p*-value of <0.05, only 3, 3, and 6 targets were identified for **3c**, ibrutinib, and **3d**, respectively, indicating again that the sulfamates have comparable proteomic selectivity.

### Sulfopin Sulfamate Analogues as Potent Inhibitors of Pin1

To demonstrate the generality of this approach, we have chosen an additional challenging target—peptidyl-prolyl isomerase NIMA-interacting-1 (Pin1), an important cancer target.^44,45^ Recently, we have developed sulfopin as a selective covalent inhibitor of Pin1, which blocks Myc-driven tumors in vivo.^38^ Sulfopin has a chloroacetamide electrophile, and previous attempts to switch it to an acrylamide or alternative warheads did not result in efficient covalent binders (data not shown). Here, we have synthesized sulfopin-based derivatives including one sulfonate (**4a**), two alkyl sulfamates (**4b** and **4c**), and four aryl sulfamate (**4d–4g**) as potential Pin1 inhibitors (Figure S22).

**Figure 5:**
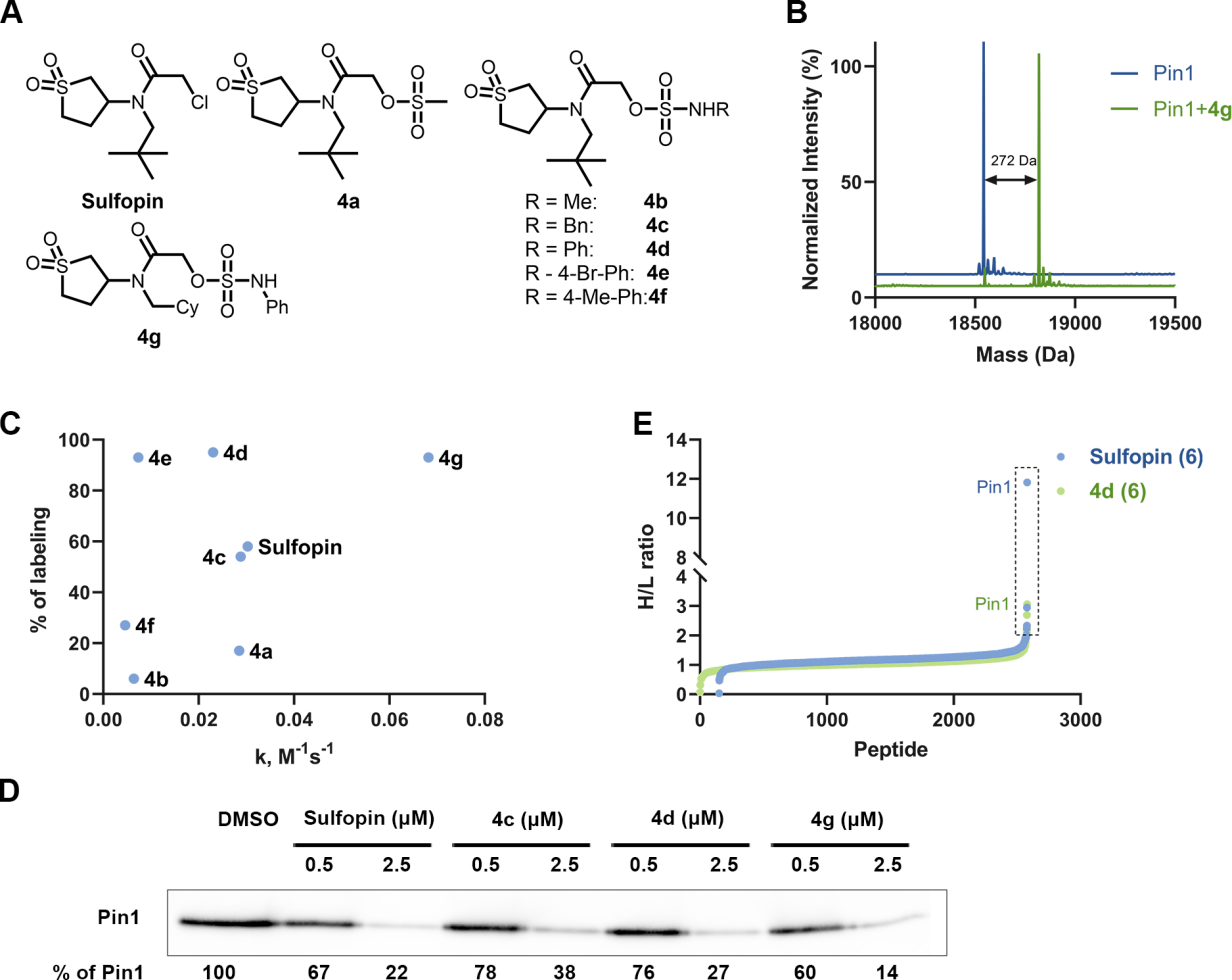
Sulfopin sulfamate acetamides as potent and selective Pin1 inhibitors. (A) Chemical structures of the sulfopin analogs (**4a**–**4g**). (B) Deconvoluted LC/MS spectrum for Pin1 (2 μM) incubated with **4g** (2 μM) at pH 7.5, 25 °C, 1 h. The adduct mass corresponds to a labeling event in which the sulfamate group was released. (C) Percent of Pin1 labeling (2 μM) with the probes (**4a–4g**; 2 μM; *y* axis) compared to their intrinsic thiol reactivity as assessed by their rate of reaction in a DTNB assay (*x* axis). (D) Cellular engagement of the sulfopin sulfamates. OCI-AML2 cells were treated with DMSO, sulfopin, or sulfamates (**4c**, **4d**, and **4 g**) at 0.5 and 2.5 μM concentration for 4 h. Lysates were then incubated with a sulfopin DTB probe^38^ (1 μM; Figure S29) pulled down using streptavidin beads before running a Western blot against Pin1. (E) IsoDTB ABPP experiment with sulfopin and sulfamate compound **4d**. PATU-8988 T cells were treated with 2.5 μM compound for 4 h followed by incubation of iodoacetamide alkyne and CuAAC click reaction with heavy/light azides containing DTB tags. The labeled peptides were pulled down with streptavidin beads and analyzed by LC/MS/MS (similar protocol to BTK; see Figure S21; *n* = 4). Proteins in the box have a heavy to light (H/L) ratio of ≥ 2. Only peptides detected in three out of four repetitions are presented.

Initially, we incubated these compounds (**4a**–**4g**; 2 μM) with Pin1 (2 μM; 50 mM Tris buffer; pH 7.5, 1 h; 25 °C) to check covalent labeling by intact protein MS (Figure 5B,C; Figure S23). Under these conditions, sulfopin labeled 59% as previously reported;^38^ the sulfonate (**4a**) labeled 18%, whereas alkyl sulfamates (**4b** and **4c**) labeled 16 and 56%, respectively. Phenyl sulfamate (**4d**) and 4-bromo phenyl sulfamate (**4e**) showed >90% labeling; however, the 4-me phenyl sulfamate (**4f**) labeled less than 30%. We have previously reported that replacing the *tert*-butyl group in sulfopin by a cyclohexyl increases the labeling along with intrinsic thiol reactivity. Compound **4g** contains a cyclohexyl group instead of the *tert*-butyl and a phenyl sulfamate electrophile and was able to label >95% of Pin1 under these conditions.

We characterized the kinetics of Pin1 binding by incubating Pin1 (0.5 μM) with various concentrations of sulfopin or **4d** (100 mM Tris buffer; pH 7.4; 14 °C) and monitoring the reaction by LC/MS at different time points and determined the *K*_I_ and *K*_inact_ for both compounds. Although the *K*_inact_ values for both sulfopin (2.39 s^–1^) and **4d** (2.41 s^–1^) are similar, the *K*_I_ value of **4d** (43.6 μM) is better than for sulfopin (216.9 μM; Figure S24), perhaps suggesting that the sulfamate group mediates additional non-covalent interactions with the protein.

To assess the reactivity of these sulfopin-based sulfamates, we carried out a DTNB thiol reactivity assay (Figure S25) and found that compounds **4c**, **4d**, and **4e** showed lower thiol reactivity than sulfopin, whereas **4g** showed slightly higher reactivity (Figure 5C). To understand the binding mode of these compounds, we modeled the pre-reacted compounds in complex with Pin1 (Figure S28). The modeling suggests that the sulfamate group has the potential to form additional hydrogen bonds with the protein and the sulfamate side-chain has room to propagate into an additional pocket on the protein that may mediate extra recognition, which can explain the increased labeling despite lower thiol reactivity.

Next, we examined the buffer stability of the sulfopin sulfamate acetamides in PBS buffer (pH 8; 37 °C). Similar to the model compounds, the chloroacetamide (sulfopin) and sulfonate (**4a**) warheads show 30 and 15% hydrolysis after 2 days, respectively (Figure S26). Sulfopin sulfamate analogues (**4b**–**4g**) showed high buffer stability even after 4 days. Moreover, both sulfopin and the sulfamate analogues (**4d** and **4g**) showed good metabolic stability (Figure S27).

Using our previously reported sulfopin-DTB probe^38^ (Figure S29A), we evaluated the cellular engagement of these compounds with Pin1 in OCI-AML2 cells. Compounds **4c**, **4d**, **4e**, and **4g** and sulfopin were incubated in OCI-AML2 cells (0.5 and 2.5 μM) followed by lysis and treatment with sulfopin-DTB (1 μM). The lysates were pulled down using streptavidin beads, and Pin1 was imaged by Western blot. Similar to sulfopin, compounds **4d** and **4g** show ∼80% of Pin1 engagement at 2.5 μM, whereas **4c** and **4e** show partial cellular labeling (Figure 5D and Figure S29B). At 0.5 μM, all of these compounds show only ∼30% Pin1 engagement. To assess the selectivity of these compounds, we have conducted a competitive isoDTB-ABPP experiment^5,43^ in PATU-8988T cells with sulfopin as well as compound **4d**. In this experiment, out of about 3000 cysteines identified by the iodoacetamide probe, Pin1 Cys113 showed the highest competition ratio for both compounds (Dataset S3). Both sulfopin and **4d** labeled only six peptides with an H/L ratio of >2. Despite Pin1 being identified as the top target for both compounds, the H/L ratio for sulfopin indicates more complete target engagement under these conditions.

### Sulfamate Warheads for Covalent Ligand-Directed Release Chemistry

**Figure 6:**
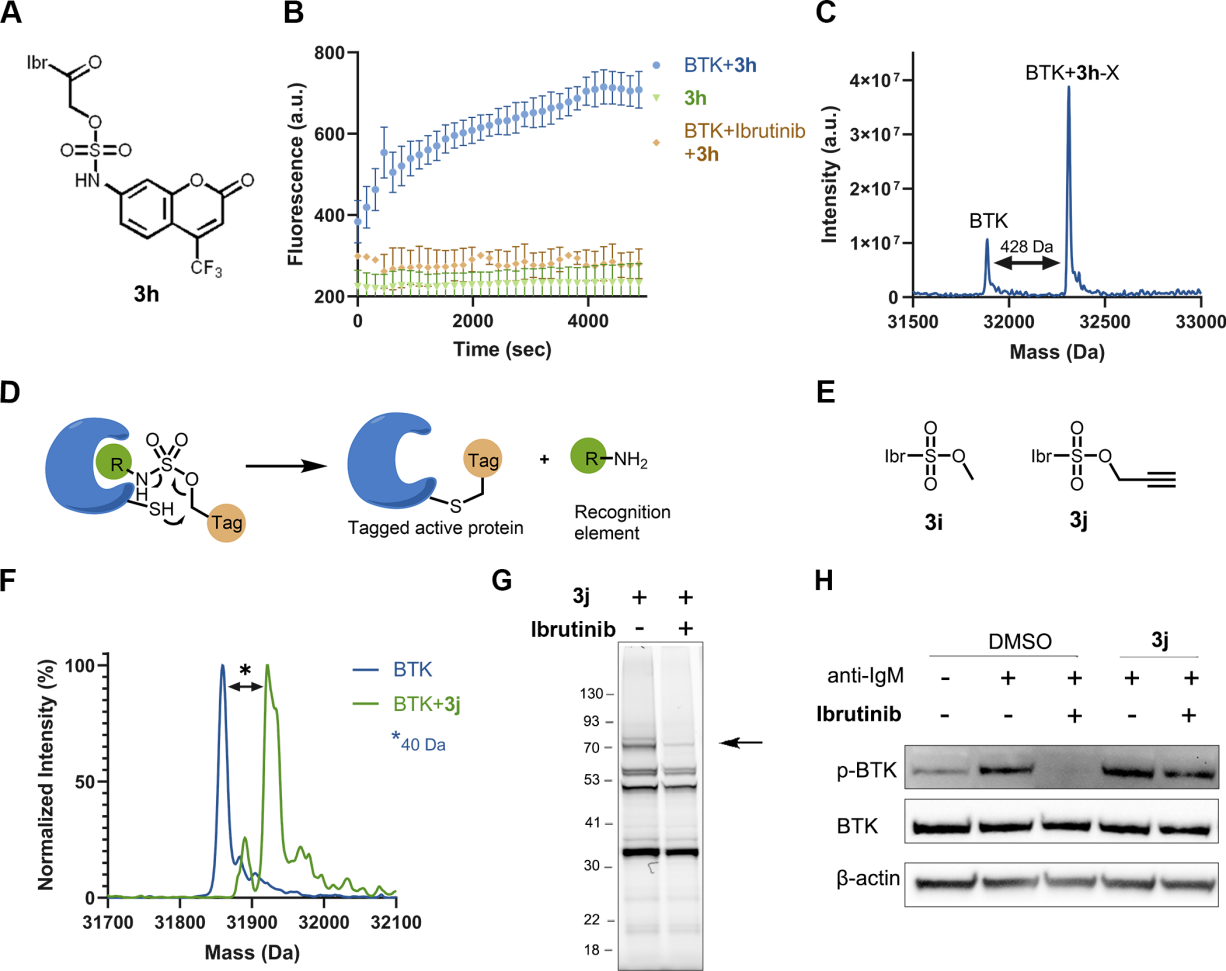
Sulfamate chemistry-based covalent ligand-directed release (CoLDR) probes. (A) Chemical structure of ibrutinib-based “turn-on” releasing probe (**3h**). (B) Time-dependent increase of fluorescence intensity (representing the release of the coumarin moiety) measured at Ex/Em = 385/435 nm (*n* = 3). The compound in and of itself (2 μM) is not fluorescent (green). Upon mixing of probe and target (2 μM; blue), we see an increase in fluorescence. Pre-incubation of the protein with ibrutinib prevents the fluorescence (orange). (C) Deconvoluted LC/MS spectra for BTK incubated with **3h** at the end of the fluorescence measurement. The adduct mass corresponds to a labeling event in which the coumarin sulfamate (indicated as X) moiety was released, validating the proposed mechanism. (D) Schematic representation of the tagging of proteins with the release of ligand. The target cysteine reaction at the electrophilic sulfamate center is followed by the concomitant release of the ligand through CoLDR chemistry. (E) Chemical structures of ibrutinib-directed sulfamates with methyl and alkyne tag. (F) Deconvoluted LC/MS spectrum shows the labeling of alkyne probe (**3j**) and demonstrates Ibr-H leaving (2 μM BTK, 2 μM **3j**, pH 8.0, 25 °C, 10 min). (G) Cellular labeling profile of **3j** (100 nM) after 2 h of incubation with Mino cells. The samples were further reacted with TAMRA-azide in lysate before imaging. An arrow indicates BTK’s MW. Upon competition with ibrutinib (preincubated for 30 min; 1 μM), BTK labeling by **3j** is lost. (H) BTK activity assay: Mino cells were incubated for 2 h with either DMSO or 1 μM **3j** and then incubated for 45 min with ibrutinib (100 nM). The cells were washed before induction of BTK activity by anti-IgM. The CoLDR probe was able to rescue BTK activity from inhibition by ibrutinib.

The use of site-specific labeling of a protein for the release of fluorescent or toxic payloads in their active form is an elegant strategy for developing diagnostics and pro-drugs. Since α-acetamido sulfamates can act as self-immolative leaving groups after a reaction with cysteine-containing proteins, we hypothesized that we could use this for the release of functional payloads. As a proof of concept of this approach, we synthesized an ibrutinib-attached sulfamate containing 4-trifluoro 7-amino coumarin (**3h**; Figure 6A and Figure S30A). We incubated compound **3h** (2 μM) with BTK (2 μM; pH 8) and measured the fluorescence over time at 435 nm. We observed a significant increase in fluorescence (4-fold) in the presence of BTK due to the release of 4-trifluoro 7-amino coumarin (Figure 6B). Pre-incubation of BTK with ibrutinib abrogated the increase in fluorescence, demonstrating that binding to the BTK active-site and/or Cys481 are required for the release of coumarin. LC/MS measurement at the end of the fluorescence experiment showed an increase in the molecular weight of the protein correlating to the molecular weight of the compound absent the coumarin sulfamate (Figure 6C). To identify the released coumarin derivative, we reacted compound **3h** with GSH (5 mM, pH 8, 37 °C, 20 mM Tris buffer) and analyzed by LC/MS at 0 h and 48 h. The LC/MS spectrum clearly shows the formation of a GSH adduct and released 4-trifluoro 7-amino coumarin after 48 h (Figure S30B).

### Traceless Labeling of Endogenous Proteins Using Sulfamate CoLDR

Site-specific labeling of endogenous proteins concomitant with the release of a directing ligand allows the tagging of proteins in their active form. Although many ligand-directed chemistries have been reported for tagging the proteins in their apo form,^29^ they have disadvantages like targeting amino acids far from the active site, large activating groups, and less than complete control on the site of labeling. Recently, methacrylamide-based CoLDR chemistry allowed targeting cysteines, specifically close to the active site of BTK.^31^ However, this method leaves an additional acrylamide moiety on the protein. In this context, we have used sulfamate chemistry for site-specific labeling of proteins where the amine group of the ligand was functionalized with a sulfo group-containing tag. When such a ligand binds to the target protein, the cysteine attacks the electrophilic carbon next to the sulfamate group and eliminates the directing ligand, leaving no or a very minimal linker to the covalently bound tag (Figure 6D). Based on the reversible binding element of ibrutinib (Ibr-H), we synthesized sulfamate analogues with a methyl group (**3i**) or an alkyne tag (**3j**; Figure 6E and Figure S31). To assess irreversible labeling and validate the proposed ligand release mechanism, we incubated **3j** (2 μM) with recombinant BTK (2 μM) and monitored the reaction via LC/MS. The analysis of the reaction with **3j** verified that the shift in mass corresponds to labeling BTK with the alkyne group and release of Ibr-H (Figure 6F). **3i** installed a single methyl on BTK (Figure S32A). We have assessed the reactivity of **3j** using a GSH consumption assay along with ibrutinib. We have found that, under the same conditions, compound **3j** did not undergo any reaction with GSH, indicating its low thiol reactivity (Figure S32B).

In addition to the in vitro labeling of BTK by our probe, we also tested its engagement in cells. Mino cells were incubated with probe **3j** for 2 h, followed by CuAAC with TAMRA-azide to the alkyne tag in lysate. The samples were imaged via gel fluorescence. Probe **3j** showed BTK labeling (70 kDa) at a concentration of 100 nM (Figure 6G and Figure S33A). When cells were pre-incubated with ibrutinib, the band at 70 kDa disappeared, confirming that the probe was indeed labeling BTK. Despite its low reactivity, the sulfamate probe **3j** has three apparent off-targets that ibrutinib did not compete. We conducted this cellular labeling experiment at higher concentrations to assess the saturation of BTK binding (Figure S33B). We see similar levels of labeling at 1 and 3 μM with additional off-targets. At 10 μM, compound **3j** presented much stronger fluorescence both for BTK and for the off-targets, similar to the acrylamide alkyne probe (Figure S33C and ref (40)). In addition, it seems that the intrinsic fluorescence for **3j** is stronger than for the acrylamide-alkyne probe, maybe as the result of the Ibr-H moiety leaving (compare Figure S33B,C). In order to examine the effect of BTK modification by these probes on its cellular activity, we performed BTK activity assays. Mino cells were incubated with probe **3j** followed by BTK activation using anti-human IgM. BTK autophosphorylation was followed by Western blot to assess its activity. While ibrutinib completely abolished BTK autophosphorylation, BTK remained active after labeling with **3j**. Further, to ensure that the activity did not originate from unlabeled BTK, cells were incubated with 100 nM ibrutinib for 45 min before activation with IgM. While ibrutinib alone completely inhibited BTK’s activity, we show that sulfamate CoLDR probe **3j** rescued this inhibition, which confirms the cellular engagement of the BTK by the probe in its active form. A slight reduction in phosphorylation upon ibrutinib incubation is observed, which may indicate incomplete BTK labeling by the probe (Figure 6H and Figure S34).

## Discussion

We have demonstrated that α-sulfamate acetamides are a new type of electrophile that can be useful for targeted covalent inhibitor design. This electrophile presents advantages over conventional electrophiles such as acrylamides and chloroacetamides in terms of the tunability of its intrinsic thiol reactivity, amino acid selectivity, and buffer/metabolic stability. Acrylamides, including ibrutinib,^46^ can be oxidatively metabolized to epoxides, which are extremely reactive. Although such epoxides are rapidly destroyed by hydrolysis or GSH conjugation, they could potentially react with proteins resulting in haptenization.^47^ By contrast, sulfamate-acetamides are not likely to be converted into more reactive metabolites. Moreover, the sulfamic acid leaving group can self-immolatively dissociate into an amine functionality with the release of sulfur trioxide (Figure 1B),^36^ allowing further functionalization.

We have shown that substituted sulfamate compounds (**1d**–**1h**) are an order of magnitude less reactive than the corresponding chloroacetamide (**1a**) and sulfonate compounds (**1b** and **1c**), whereas the aryl-substituted and secondary amine substituted sulfamates show even drastically lower reactivity (Figure 2B). This may be because the electronics of the amine reduces the electrophilicity of the α-carbon of the sulfamate acetamides (Figure S3E). The proteomic reactivity of the sulfamates reflected similar reactivity trends with the more reactive chloroacetamide (**2c**) and benzyl sulfamate acetamide (**2a**) labeling more proteins than the low reactivity phenyl sulfamate acetamide (**2b**; Figure 2C).

The fact that additional bands were detected with the phenyl sulfamate acetamide compared to the chloroacetamide may suggest that the extra recognition of the phenyl sulfamate mediates additional interactions with some protein targets. Similar to substituted methacrylamides,^30^ these compounds also leave identical adducts on proteomically labeled cysteines. Hence, mixtures of sulfamates can serve as probes for quantitative chemoproteomics with potentially increased coverage.

In the context of targeted covalent inhibitors, the ibrutinib sulfamate analogues (**3c**–**3e**) showed similar labeling efficiency and inhibition toward BTK compared to ibrutinib. Ibr-sulfamates showed a thiol reactivity pattern similar to model compounds. Interestingly, the low reactivity sulfamate acetamides (**3c** and **3d**) show higher kinase inhibition than chloro- and sulfonate acetamides (Figure 3D), as well as better cellular pBTK inhibition profile than the chloroacetamide (Figure 4A). This may suggest that the sulfamate group contributes to additional recognition of the protein.

The ibrutinib methyl sulfamate analogue (**3c**) showed equivalent performance to ibrutinib in many settings: in vitro kinase activity assay (Figure 3D), tissue culture, primary mouse B cells (Figure 4A,B), and CLL patient samples (Figure 4C), while displaying similar proteomic selectivity (Figure 4G,H) and improved metabolic stability (Figure S15).

When **3c** was administered orally to mice with an accepted model of CLL, a reduction in B-cell numbers and spleen sizes was observed similar to previous reports with ibrutinib^48−50^ or Acalabrutinib^51^ at the same concentration, demonstrating the oral bioavailability of this compound, as well as its suitability for in vivo administration. Taken together, this data makes a strong case for sulfamate acetamides to be considered as appropriate electrophiles for therapeutic development of covalent inhibitors.

While BTK accommodated an electrophile switch from an acrylamide to a chloroacetamide (and sulfamate acetamide), many protein targets do not tolerate covalent binding to both types of electrophiles. Recently, many chloroacetamide inhibitors have been identified by covalent fragment screening.^18−21,52,53^ Chloroacetamide electrophiles may have low buffer stability and high reactivity (Figure 2B and Figure S3). Hence, α-sulfamate acetamides can be a promising alternative to chloroacetamide inhibitors based on the fact that they maintain the same binding geometry. This will make them a useful substitution strategy in covalent medicinal chemistry campaigns. Pin1 exemplifies this well.

Pin1 has proven to be a challenging target, and only a handful of Pin1 inhibitors, either reversible^54−56^ or irreversible^57,58^ have been reported. Low specificity, permeability, and liver microsomal stability made them unsuitable for in vivo applications. Recently, we have reported a highly selective Pin1 covalent inhibitor with a chloroacetamide warhead.^38^ The sulfopin sulfamate analogues (**4c**–**4g**) showed high labeling of Pin1 and lower thiol reactivity compared to the already mild sulfopin (Figure 5C and Figure S25A,B). Modeling studies of these sulfopin analogues bound to Pin1 suggested a secondary pocket next to the active site that can accommodate sulfamate substitutions (Figure S28), potentially mediating additional interactions with the protein, which is also supported by a 5-times better K_I_ value for **4d** over sulfopin (Figure S24). These analogues also showed similar proteomic selectivity and cellular engagement to sulfopin. We should note that previously, any substitution of the chloroacetamide in sulfopin to another electrophile abrogated its activity. Thus, sulfamate acetamides constitute a promising new approach for optimization of Pin1 covalent inhibitors.

A final advantage of sulfamate acetamides as electrophiles is their potential for the functionalization of covalent binders for various chemical biology applications. Over the last decade, Hamachi and co-workers have developed many ligand-directed chemistries for site-selective labeling of proteins in cells.^59−61^ The same group has developed *N*-acyl-*N*-alkyl sulfonamide chemistry that has been used both as covalent warhead^62^ and as a labeling method.^29^ Recently, we have developed methacylamide-based covalent warheads used not only for the development of potential inhibitors but also for covalent ligand-directed release (CoLDR) chemistry. This chemistry allowed distinct applications such as the release of some functional moiety (such as a fluorophore) following the reaction with the target amino acid.^30^ Another use was for site-specific labeling of endogenous proteins in cells.^31^ However, it is limited to acrylamide-based covalent inhibitors.

Sulfamate acetamides allow similar applications in covalent ligand-directed chemistry. Upon cysteine attack of the electrophilic site, the self-immolative sulfamate group releases an amine payload (for example, a fluorescent group) in its active form. In the case of ibrutinib, we have demonstrated the release of 7-amino-4-trifluoro coumarine after reaction with BTK, which resulted in enhanced fluorescence (Figure 6B). Since there is a wide scope of compatible leaving group functionalities, numerous potential cargoes should be available for targeted release using this strategy, such as pro-drugs^63,64^ and imaging agents.^63,65^

Another important application of sulfamate-based CoLDR chemistry is site-specific labeling of proteins. The ability to functionalize an amine on a covalent inhibitor into a sulfamate allows the release of said inhibitor upon covalent binding while tagging the protein with an arbitrarily small tag. We demonstrated this here with installation of a single methyl or propargyl on BTK while preserving the protein’s activity. This could be extended to the installation of other functional tags such as fluorophores, bioorthogonal handles, E3 ligase recruiters, or a neo-substrate to potentially induce neo-phosphorylation by BTK. Compared to our previously reported methacrylamides, tagging with sulfamates is “traceless” and allows much smaller tags.

In summary, sulfamate acetamides are a promising new electrophile for targeted covalent inhibitors suitable for in vivo administration and represent tunable and versatile chemistry for a variety of chemical biology applications.

## Methods

### LC/MS Measurements

LC/MS runs were performed on a Waters ACQUITY UPLC class H instrument in positive ion mode using electrospray ionization. UPLC separation for small molecules used a C18-CSH column of (1.7 μm, 2.1 mm × 100 mm) for all the LC/MS-based assays. The column was held at 40 °C, and the autosampler was held at 10 °C. Mobile phase A was 0.1% formic acid in water, and mobile phase B was 0.1% formic acid in acetonitrile. The run flow was 0.3 mL/min. The gradient used was 100% A for 2 min, increasing linearly to 90% B for 5 min, holding at 90% B for 1 min, changing to 0% B in 0.1 min, and holding at 0% for 1.9 min. UPLC separation for proteins used a C4 column (300 Å, 1.7 μm, 2.1 mm × 100 mm). The column was held at 40 °C and the autosampler at 10 °C. Mobile solution A was 0.1% formic acid in the water, and mobile phase B was 0.1% formic acid in acetonitrile. The run flow was 0.4 mL/min with gradient 20% B for 2 min, increasing linearly to 60% B for 3 min, holding at 60% B for 1.5 min, changing to 0% B in 0.1 min, and holding at 0% for 1.4 min. The mass data were collected on a Waters SQD2 detector with an *m*/*z* range of 2–3071.98 at a range of *m*/*z* of 800–1500 Da for BTK, and 750–1550 for Pin1.

### GSH Reactivity Assay for Model Compounds and Ibrutinib Sulfamates

A 100 μM (for **3a**–**3e**) 200 μM (for **1a**–**1h**) (0.5 or 1 μL of 20 mM stock) sample of the electrophile was incubated with 5 mM GSH (5 μL of 100 mM stock, freshly dissolved), 5 mM NaOH (to counter the acidity imparted by GSH), and 100 μM 4-nitrocyano benzene (0.5 μL of 20 mM stock solution) as an internal standard in 100 mM potassium phosphate buffer (pH 8.0) and DMF at a ratio of 9:1, respectively. All solvents were bubbled with argon. Reaction mixtures were kept at 10 °C. Every 1 h, 5 μL from the reaction mixture were injected into the LC/MS. The reaction was followed by the peak area of the electrophile normalized by the area of the 4-nitrocyano benzene (i.e., by the disappearance of the starting material). The natural logarithm of the results was fitted to linear regression, and *t*_1/2_ was calculated as *t*_1/2_ = ln 2/–slope.

### DTNB Thiol Reactivity Assay

The compound 5,5′-dithio-bis(2-nitrobenzoic acid) (DTNB; 50 μM) was incubated with 200 μM tris(2-carboxyethyl)phosphine (TCEP) in 20 mM sodium phosphate buffer (pH 7.4) and 150 mM NaCl for 5 min at room temperature to obtain TNB^–2^. Next, 200 μM compounds were subsequently added to TNB^–2^ followed by immediate ultraviolet (UV) absorbance measurement at 412 nm and 37 °C. UV absorbance was acquired every 15 min for 7 h. The assay was performed in a 384-well plate using a Tecan Spark 10 M plate reader. Background absorbance of compounds was subtracted by measuring the absorbance at 412 nm of each compound under the same conditions without DTNB. Compounds were measured in triplicate. The data were fitted to a second-order reaction equation such that the rate constant (*K*) is the slope of ln([A][B0]/[B][A0]), where [A0] and [B0] are the initial concentrations of the compound (200 μM) and TNB^–2^ (100 μM), respectively, and [A] and [B] are the remaining concentrations as a function of time as deduced from spectrometric measurements. Linear regression using Prism was performed to fit the rate against the first 7 h of measurements.

### Buffer Stability Assay for Model Compounds, Ibrutinib Sulfamates, and Sulfopin Sulfamates

A sample of the electrophile (200 μM for **1a**–**1i** and **4a**–**4g** and 100 μM for **3a–3g)** was incubated with 100 μM of 4-nitrocyano benzene as an internal standard in a 100 mM potassium phosphate buffer of pH 8.0. Reaction mixtures were kept at 37 °C with shaking. After 4 days (unless otherwise mentioned), 5 μL from the reaction mixture were injected into the LC/MS to check the stability of the compounds.

### In-Gel Fluorescence Labeling Profile

Mino cells were cultured in RPMI-medium supplemented with 15% FBS and 1% p/s at 37 °C and 5% CO_2_. The cells were treated for 2 h with either 0.1% DMSO or the indicated concentrations of **2a**–**2c** or **3j**. For the competition experiment, the cells were pre-incubated for 30 min with 1 μM ibrutinib followed by 2 h incubation with 100 nM **3j**. The cells were lysed with RIPA buffer (Sigma, R0278), and protein concentration was determined using BCA protein assay (Thermo Fisher Scientific, 23225). Lysates were then diluted to 2 mg/mL in PBS. Lysates were clicked to TAMRA-azide (Lumiprobe). “Click” reaction was performed using a final concentration of 40 μM TAMRA-azide, 3 mM CuSO_4_, 3 mM Tris(3-hydroxypropyltriazolylmethyl)amine (THPTA, Sigma), and 3.7 mM Sodium l-ascorbate (Sigma) in a final volume of 60 μL. The samples were subjected to precipitation.

Precipitation: 1× chloroform, 4× methanol, and 3× water were added to the samples and vortexed thoroughly. The samples were spun down for 10 min at 4 °C, 21,000*g*. The top layer was aspirated, and the pellet was resuspended in 4× methanol. The sample was vortexed and spun down again for 10 min at 4 °C, 21,000*g*, the solution was removed, and the pellet was dried for 2 min. The pellet was dissolved in 42 μL of PBS followed by a 14 μL of 4× sample buffer. The samples were then loaded on a 4–20% Bis–Tris gel (SurePAGE, GeneScript) and imaged at 532 nm using a Typhoon FLA 9500 scanner.

### In Vitro Labeling Experiments

BTK kinase domain was expressed and purified as previously reported.^66^ Binding experiments were performed in Tris 20 mM (pH 8.0) and 50 mM NaCl at room temperature. The BTK kinase domain was diluted to 2 μM in the buffer, and 2 μM ibrutinib derivatives (**3a**–**3g**) were added by adding 1/100th volume from a 200 μM solution.

Pin1 was expressed and purified as previously reported.^38^ The catalytic domain of Pin1 (2 μM) in 20 mM Tris and 75 mM NaCl (pH 7.5) was incubated with 2 μM sulfopin sulfamate (**4a**–**4g**) for 1 h at 25 °C.

The reaction mixtures, at room temperature for various times, were injected into the LC/MS. For data analysis, the raw spectra were deconvoluted using a 20,000:40,000 Da (For Pin1, 10,000:30,000) window and 1 Da resolution. The labeling percentage for a compound was determined as the labeling of a specific compound (alone or together with other compounds) divided by the overall detected protein species.

### In Vitro Kinase Activity (Carried Out by Nanosyn, Santa Clara, CA, USA)

Kinase reactions are assembled in 384-well plates (Greiner) in a total volume of 20 μL. Test compounds (**3a**–**3g**) were diluted in DMSO to a final concentration, while the final concentration of DMSO in all assays was kept at 1%. The compounds were incubated with the kinases for 30 min. A 0.5 nM concentration of BTK in 100 mM HEPES (pH 7.5), 0.1% BSA, 0.01% Triton X-100, 1 mM DTT, and 5 mM MgCl_2_ were used. The reaction was initiated by 2-fold dilution into a solution containing 5 μM ATP and 1 μM substrate in the kinase buffer.

### BTK Activity in Mino Cells

Mino cells were treated with indicated concentration of the compounds (**3a**–**3g** and **3j**). The cells were then incubated with 10 μg/mL anti-human IgM (Jackson ImmunoResearch, 109-006-129) for 10 min at 37 °C and harvested. The cell pellets were subjected to immunoblotting, and Western blots were performed for p-BTK, BTK, and β-actin.

### Immunoblotting

Cell pellets were washed with ice-cold PBS and lysed using RIPA buffer (Sigma, R0278). Lysates were clarified at 21,000*g* for 15 min at 4 °C and protein concentration was determined using BCA protein assay (Thermo Fisher Scientific, 23225). Samples containing 50 μg total protein were prepared with 4× LDS sample buffer (NuPAGE, Thermo Scientific, NP0007) and 20 mM DTT, which were then resolved on a 4–20% Bis–Tris gel (GeneScript SurePAGE, M00657). Proteins were separated by electrophoresis and were then transferred to a nitrocellulose membrane (BioRad, 1704158) using the Trans-Blot Turbo system (BioRad). The membrane was blocked with 5% BSA in TBS-T (w/v) for 1 h at room temperature, washed 3 times for 5 min with TBS-T, and incubated with the following primary antibodies: rabbit anti-phospho-BTK (#87141s, Cell Signaling, 1:1000, overnight at 4 °C), mouse anti-BTK (#56044s, Cell Signaling, 1:1000, 1 h at room temperature), mouse anti-β-actin (#3700, Cell Signaling, 1:1000, 1 h at room temperature). The membrane was washed 3 times for 5 min with TBS-T and incubated with the corresponding HRP- linked secondary antibody (Mouse #7076 /Rabbit #7074, Cell Signaling) for 1 h at room temperature. An EZ-ECL Kit (Biological Industries, 20-500-1000) was used to detect HRP activity. The membrane was stripped using Restore stripping buffer (Thermo Fisher Scientific, 21059) after each secondary antibody before blotting with the next one.

### B-Cell Response Experiment

Splenic cells from C57BL/6 mice were isolated by forcing spleen tissue through the mesh into PBS containing 2% fetal calf serum and 1 mM EDTA, and red blood cells were depleted by lysis buffer. Cells were cultured in 96-well U-bottom dishes (1 × 10^6^ cells/mL in RPMI 10% FCS) and incubated with ibrutinib, IbrCl-1342 in different concentrations (1, 10, 100, and 1000 nM), for 24 h at 37 °C in 5% humidified CO_2_. Following a 24 h incubation, cells were stimulated with anti-IgM overnight (5 μg/mL, Sigma-Aldrich). Subsequently, cells were stained with anti-B220 (clone RA3-6B2, BioLegend) and anti-CD86 (clone GL-1, BioLegend) antibodies (anti-mouse CD86, BioLegend 105008 (1:400) and anti-mouse/human CD45R/B220, BioLegend 103212 (1:400)) for 30 min at 4 °C. Single-cell suspensions were analyzed by a flow cytometer (CytoFlex, Beckman Coulter).

### BTK Activity in CLL Patient Samples

CLL cells (20 × 10^6^/mL) were incubated with ibrutinib or ibrutinib-based compounds (**3a** and **3c**–**3e**), at the indicated doses at 37 °C. DMSO-treated cells served as controls. After 2 h of incubation, the cells were stimulated with goat F(ab′)2 anti-human IgM (10 μg/mL) for 15 min or left untreated. CLL cells were lysed in RIPA lysis buffer (Cell Signaling Technology, Beverly, MA) containing phosphatase inhibitor cocktail 2 and protease inhibitor cocktail (Sigma-Aldrich, MO, USA). Extract from cell lysates were separated on 4–15% Criterion TGX Precast Midi Protein Gel (BioRad Laboratories) and transferred electrophoretically to nitrocellulose membrane (BioRad Laboratories). The membranes were incubated with the designated antibodies and HRP-conjugated secondary antibodies according to the manufacturer’s instructions. Bands were detected using MyECL Imager (Thermo Scientific, Rockford, IL). A Western blot analysis showed PLCγ2, BTK, Akt, and ERK phosphorylation as well as the total amount of these proteins. Actin was used to verify equal loading. More details are available in the Supplementary Information.

### Pull-Down Proteomics Experiments

Mino cells were incubated for 1 h with DMSO, ibrutinib, **3c**, and **3d** followed by incubation with 10 μM “probe 4” for another hour. The cells were lysed and “clicked” with biotin-azide (Click Chemistry Tools, CAT 1265), and the samples were incubated at room temperature for 1 h. The samples were then precipitated with methanol: chloroform (1 mL of methanol, 250 μL of chloroform, and 750 μL of water), washed with 1 mL of methanol, and air-dried. The samples were solubilized and bound to streptavidin agarose beads in PBS for 3 h at 25 °C. The beads were washed, centrifuged, and resuspended in Tris 50 mM pH 8 and transferred to a clean Eppendorf tube. After this, the bound proteins were eluted by boiling with 5% SDS then reduced with DTT, alkylated with iodoacetamide, and digested with trypsin. The samples were run on LC/MS/MS. The detailed procedure is available in the Supplementary Information. The mass spectrometry proteomics data have been deposited to the ProteomeXchange Consortium via the PRIDE^67^ partner repository with the dataset identifiers PXD038301 and PXD038375.

### μTCL1 Adoptive Transfer Model

TCL1 mice for this model were generated as previously described.^42^ For this experiment, TCL1 mice approximately 12 months of age with a malignant cell population higher than 60% in the PB were sacrificed. Their spleens were excised, and 4 × 10^7^ cells resuspended in PBS^–/–^ were injected into the tail vein of 6 week old recipient mice. Progression of the disease was followed in the PB by using flow cytometry for the +IgM/+CD5 population. Mice with >30% IgM+/CD5+ cells were considered to be diseased and were used for further analysis.

### Staining for Flow Cytometry

Isolated cells were stained using specific antibodies (IgM-PE, CD5-APC, BioLegend) in staining buffer (0.5% bovine serum albumin in phosphate-buffered saline) for 30 min in 4 °C in dark then washed twice. Flow cytometry (FACS) analysis was performed using FACS Canto (BD Biosciences), and data were collected using FACSDIva8 (BD Biosciences). FACS data analysis was done using FlowJo v10.

### BTK Engagement in Treated Mice Spleens

Pellets of harvested spleens were lysed using RIPA buffer (Sigma, R0278) and clarified at 21,000*g* for 15 min at 4 °C, and protein concentration was determined using BCA protein assay (Thermo Scientific, 23225). Lysates were diluted to 2 mg/mL, 50 μL per sample, and incubated for 1 h in room temperature with 1 μM probe 4 to label BTK. Lysates were then clicked to TAMRA-azide and imaged using ChemiDoc MP (546 nm) as described in the in-gel fluorescence protocol.

### Pin1 Pull-Down Using Sulfopin-DTB Probe

OCI-AML2 cells were treated for 4 h with either DMSO (0.1%) or sulfopin, **4c**, **4d**, **4e**, and **4g** (0.5 and 2.5 μM). The cells were lysed using RIPA buffer (Sigma, R0278). Lysates were clarified at 21,000*g* for 15 min at 4 °C, and protein concentration was determined using BCA protein assay (Thermo Scientific, 23225). Lysates were incubated with 1 μM sulfopin-DTB probe for 1 h at room-temperature, using 650 μg per sample. Streptavidin-agarose beads (Thermo Scientific, 20349) were added, 50 μL per sample, and placed on a shaker for 2 h in room temperature. The beads were washed four times with 500 μL of buffer containing 20 mM Hepes (pH 7.5), 10 mM NaCl, 1 mM EDTA, and 10% glycerol. Beads were then pelleted and boiled in 50 mL 2× LDS sample buffer (Invitrogen, NuPAGE, NP0007), and Pin1 immunoblotting was performed. The samples were loaded on a 4–20% Bis–Tris gel (SurePAGE) and transferred to a nitrocellulose membrane (BioRad, 1704158) using Trans-Blot Turbo system (BioRad). The membrane was blocked using 5% BSA in PBS-T (w/v) for 1 h at room temperature, washed 3 times for 5 min with PBS-T, and incubated overnight at 4 °C with Pin1 antibody diluted to 1:1000 (Cell Signaling, 3722). Membrane was washed 3 times for 5 min with PBS-T and incubated with anti-rabbit HRP- linked secondary antibody (Cell Signaling, #7074) for 1 h at room-temperature. An EZ-ECL Kit (Biological Industries, 20–500-1000) was used to detect HRP activity.

### Sulfopin Analogue Docking

We used RDKit (www.rdkit.org) to generate 100 unconstrained conformers of each compound, and an additional 100 conformers where the common substructure was constrained to fit the crystallographic conformation of bound sulfopin (PDB ID: 6VAJ) using the enforceChirality option to include only the stereoisomer observed for sulfopin. We kept only conformers with RMSDs of >0.1 Å to all previous conformers. Each compound was then parametrized for Rosetta using the molfile_to_params.py script provided in the Rosetta software suit^68^ and modeled in the binding pocket of Pin1 (PDB ID: 6VAJ) using the RosettaScripts interface.^69^ We first pre-packed the structure by packing and minimizing the receptor and the ligand separately and then used the high-resolution modeling steps used in the ligand docking XML protocol^70^ to produce 1000 models of each complex while applying distance constraints to enforce the hydrogen bonds between the sulfolane moiety to the backbone of Gln131 and the side chain of His157. We then manually chose one of the top 10 models according to the interface score.

## Supporting Information Available

Additional information including synthesis schemes of all sulfamate acetamides, GSH consumption assay results, DTNB assay results, potential reaction mechanisms, buffer & metabolic stabilities, intact protein MS of compound binding to BTK, in vitro kinase activity assays against BTK and off-targets, fully scanned gels for all WB, primary B cell assay results, FACS analysis and images of spleens from the mice experiment with ibrutinib sulfamates, determination of kinetic parameters and molecular modeling of sulfopin analogues, gels from CLL patient samples, technical details of mice experiments and chemoproteomic methods, and detailed synthetic protocols for preparation of compounds with high-resolution mass spectrometry and NMR analysis (PDF)
Chemoproteomics results for pull-down and IsoDTB ABPP experiments for ibrutinib and sulfopin analogues (XLSX)
