# Supplementary material for: Sulfamate Acetamides as Self-Immolative Electrophiles for Covalent Ligand-Directed Release Chemistry
Source: J Am Chem Soc. 2023 Feb 4;145(6):3346–60. doi: 10.1021/jacs.2c08853 (PMC9936582; doi:10.1021/jacs.2c08853)
Supplement: Supplementary file 1 — ja2c08853_si_001.pdf [file ja2c08853_si_001.pdf]

Sulfamate acetamides as self-immolative electrophiles for covalent ligand-directed release chemistry

Rambabu N. Reddi[1,*], Adi Rogel[1], Ronen Gabizon[1], Dattatraya Gautam Rawale[1], Battu Harish [1], Shir Marom[1], Barr Tivon[1], Yamit Shorer Arbel[2], Neta Gurwicz[3], Roni Oren[4], Keren David[3], Jingjing Liu[3], Shirly Duberstein[5], Maxim Itkin[6], Sergey Malitsky[6], Haim Barr[5], Ben-Zion Katz[2,7], Yair Herishanu[2,7], Idit Shachar[3], Ziv Shulman[3], Nir London[1,*]

[1] Dept. of Chemical and Structural Biology, The Weizmann Institute of Science, Rehovot, 7610001, Israel.

[2] Sackler Faculty of Medicine, Tel Aviv University, Tel-Aviv 6997801, Israel

[3] Dept. of Systems Immunology, The Weizmann Institute of Science, Rehovot, 7610001, Israel.

[4] Department of Veterinary Resources, The Weizmann Institute of Science, Rehovot, 7610001, Israel

[5] Wohl Institute for Drug Discovery of the Nancy and Stephen Grand Israel National Center for Personalized Medicine, The Weizmann Institute of Science, Rehovot, 7610001, Israel.

[6] Life Sciences Core Facilities, The Weizmann Institute of Science, Rehovot, 7610001, Israel

[7] Department of Hematology, Tel Aviv Sourasky Medical Center, Tel Aviv, 6423906, Israel

[*] To whom correspondence should be addressed:

R.N.R. rambabu.reddi78@gmail.com

N.L. nir.london@weizmann.ac.il

Keywords: Sulfamate chemistry, targeted covalent inhibitors, BTK, Pin1, Turn-on fluorescence, site-specific labeling

Supplementary information

Supplementary figures

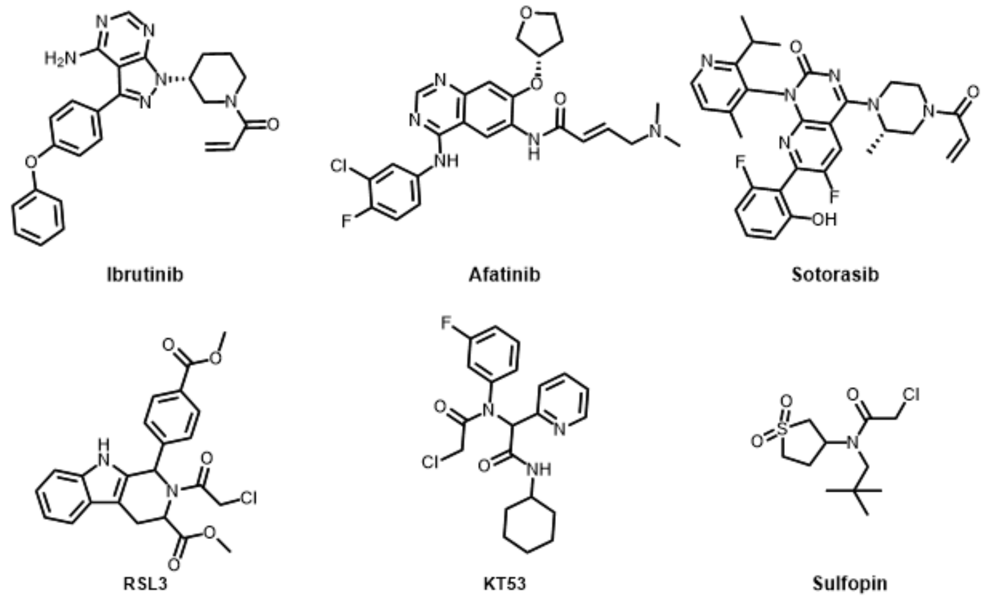

Figure S1: Structures of notable acrylamide and chloroacetamide-based covalent inhibitors. Ibrutinib (BTK inhibitor), Afatinib (EGFR inhibitor), Sotorasib (KRAS^{G12C} inhibitor), RSL3 (GPX4 inhibitor), KT53 (Glutathione *S*-Transferase Omega 1 inhibitor), and Sulfopin (Pin1 inhibitor).

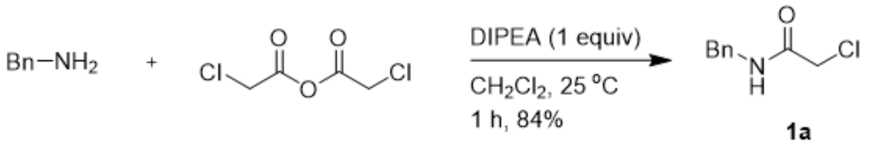

1a

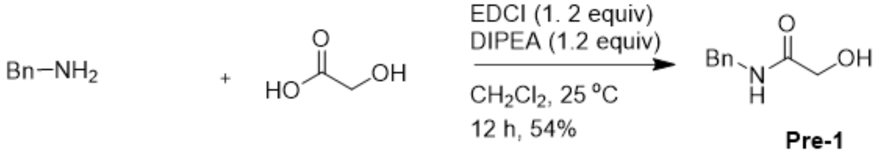

Pre-1

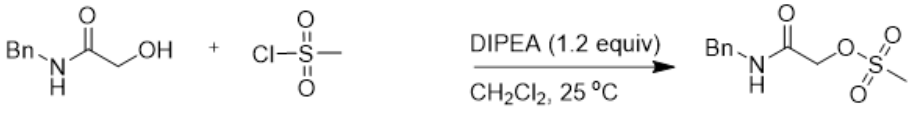

Pre-1 **1b**

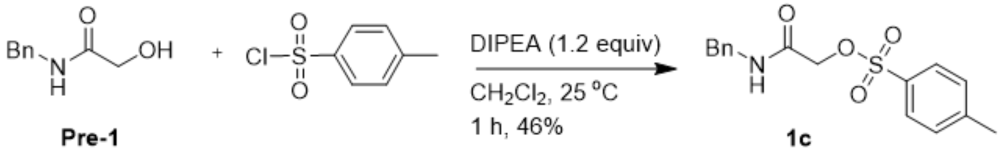

Pre-1 **1c**

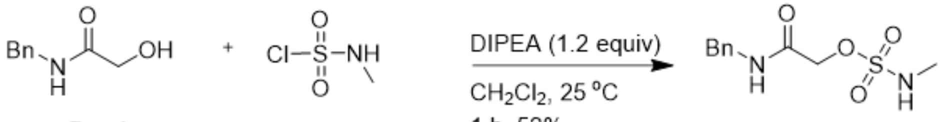

Pre-1 **1d**

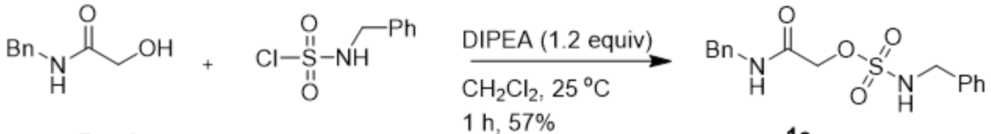

Pre-1 **1e**

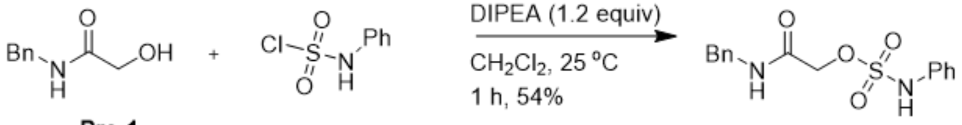

Pre-1 **1f**

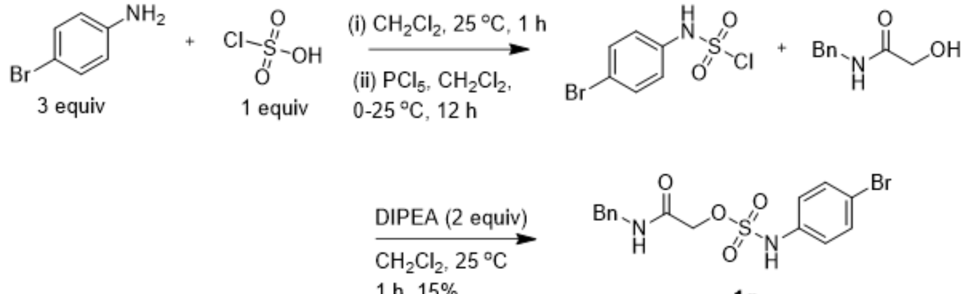

1g

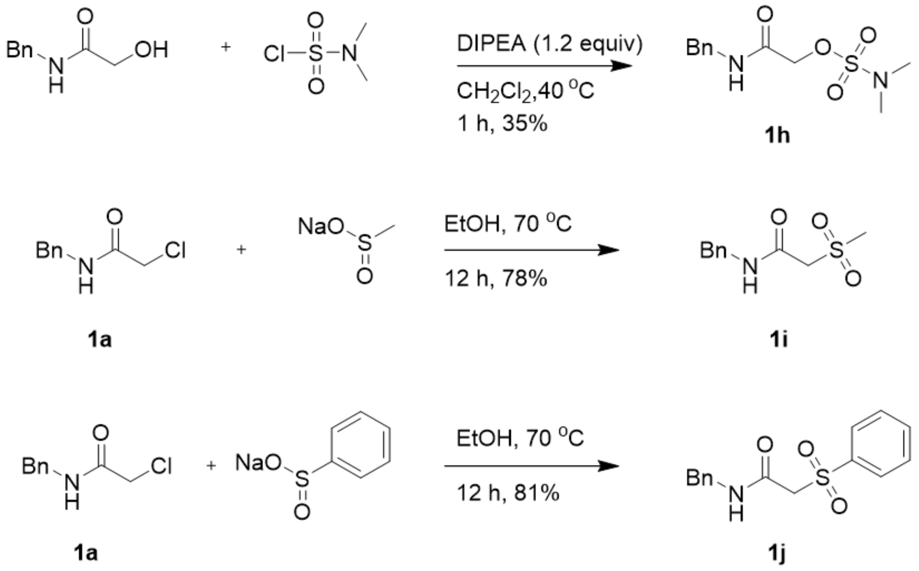

Figure S2: Synthetic schemes of model compounds.

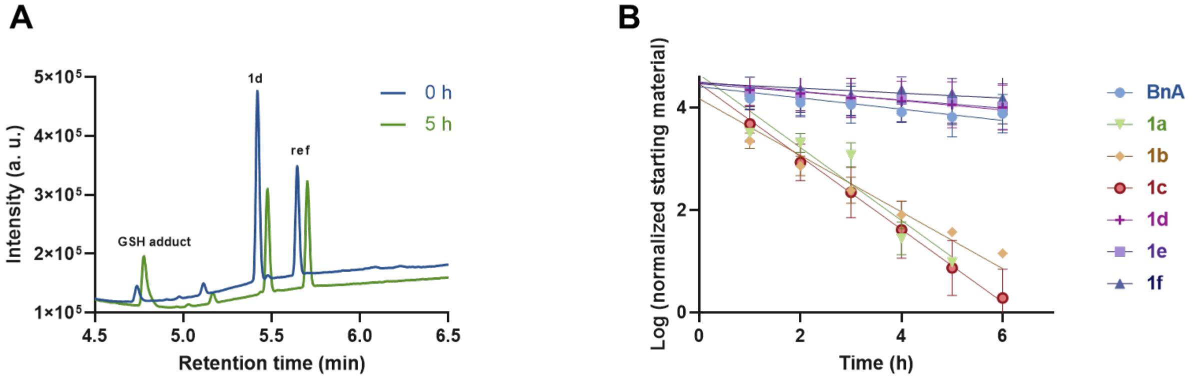

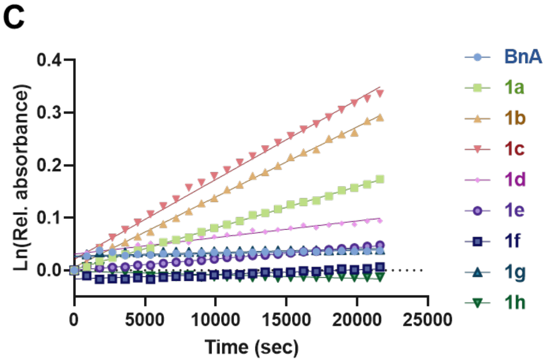

D

Compound	k, $M^{-1}s^{-1}$
**BnA**	0.0025
**1a**	0.0802
**1b**	0.1342
**1c**	0.1610
**1d**	0.0299
**1e**	0.0206
**1f**	0.0089
**1g**	0.0035
**1h**	0.0120

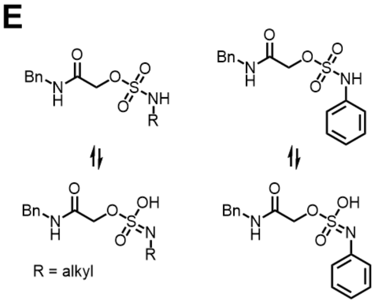

Figure S3: Sulfamate acetamides show varied reactivity profiles: A. An example LC chromatogram shows monitoring of the reaction of **1d** (200 µM) with GSH (5 mM) at 0 h (blue) and 5 h (green). GSH adduct: Retention time (RT) = 4.7 min, m/z = 465; **1d:** RT = 5.45 min, m/z = 253; reference: RT = 5.8 min. UV absorption was measured between 220-400 nm. **B.** Rates of depletion of model compounds (**1a**-**1g**) in a reaction between 200 µM compound and 5 mM GSH in PBS buffer at pH 8, 14 °C (n=2) for 6 h. **C.** Estimation of intrinsic thiol reactivity as determined by a DTNB assay (see methods) **D.** The second-order reaction rate constants for reaction with DTNB. **E.** Possible resonance structures for the sulfamate compounds.

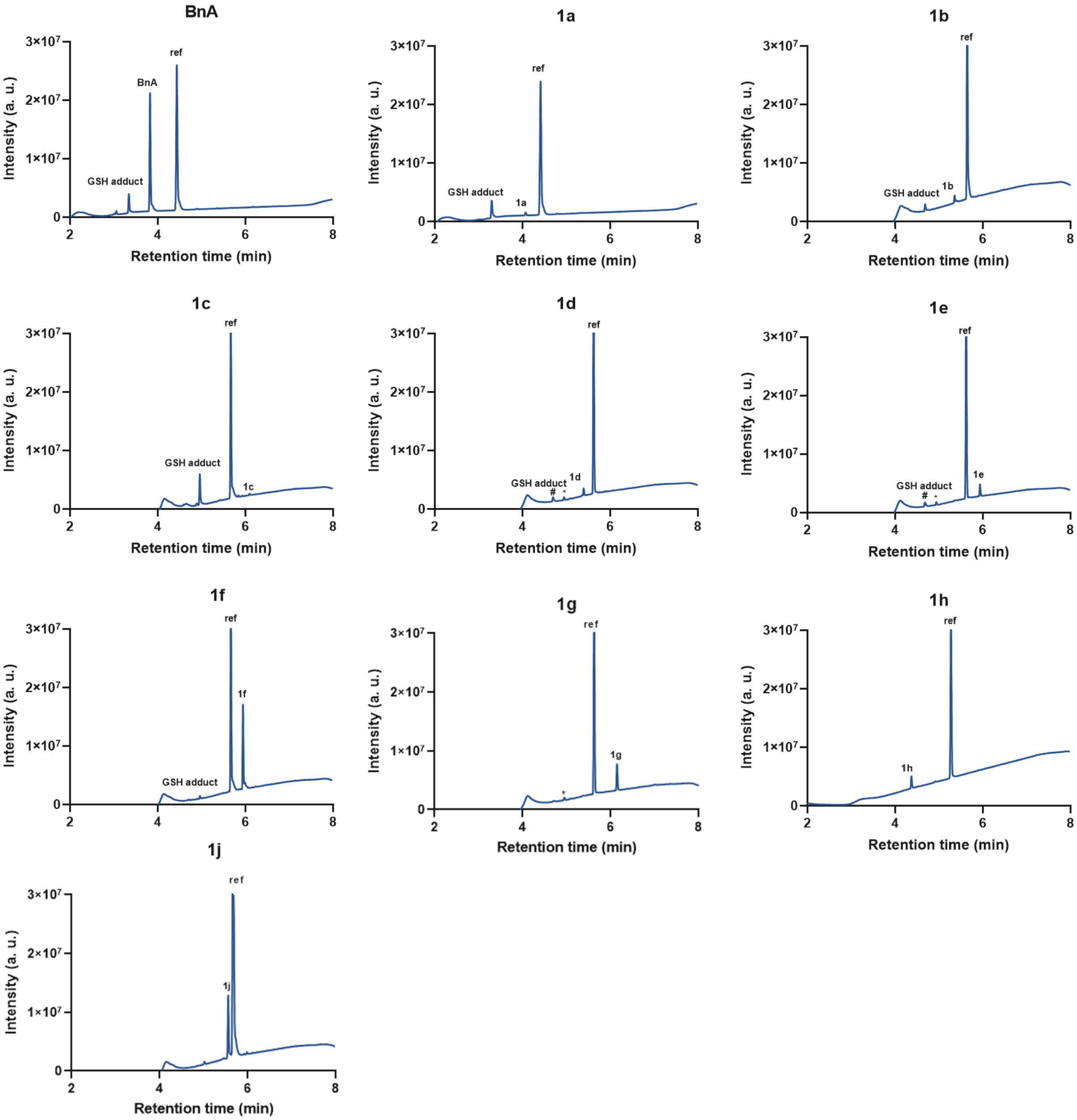

Figure S4: GSH chromatograms for all the model compounds: UV chromatogram (220-400 nm) of the LC/MS traces of model compounds (200 µM; **BnA**, **1a**-**1h,** and **1j**) incubated with reduced glutathione (5 mM) and 4-nitro cyano benzene (100 µM) in PBS buffer at pH 8, 14 °C at the last measured point of the GSH $t_{1/2}$ experiment (Figure S3B) .* Impurity obtained from the column during the assay with **1d**, **1e,** and **1g**.

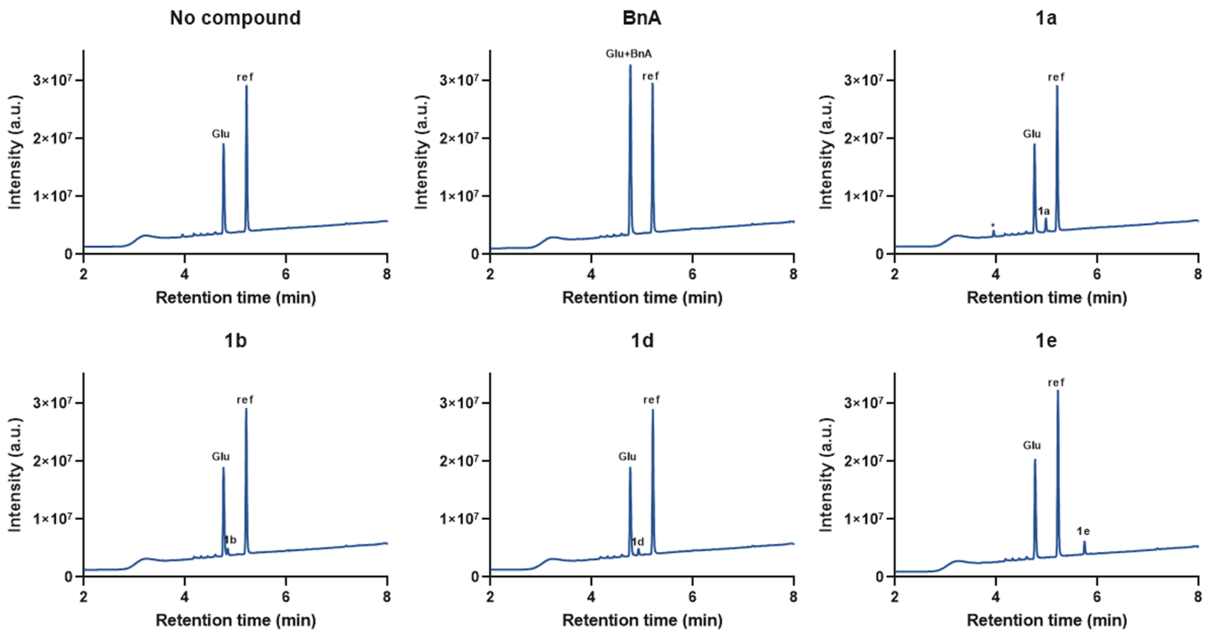

Figure S5: Glutamic acid reactivity of model compounds: UV chromatogram (220-400 nm) of the LC/MS traces of model compounds (200 µM; **BnA**, **1a**, **1b**, **1d,** and **1e**) incubated with N-acyl glutamate methyl ester (5 mM) and 4-nitrocyano benzene (100 µM) in PBS buffer at pH 8, 37 °C, 4 days. No significant reaction has been observed with model compounds.* hydrolysis product.

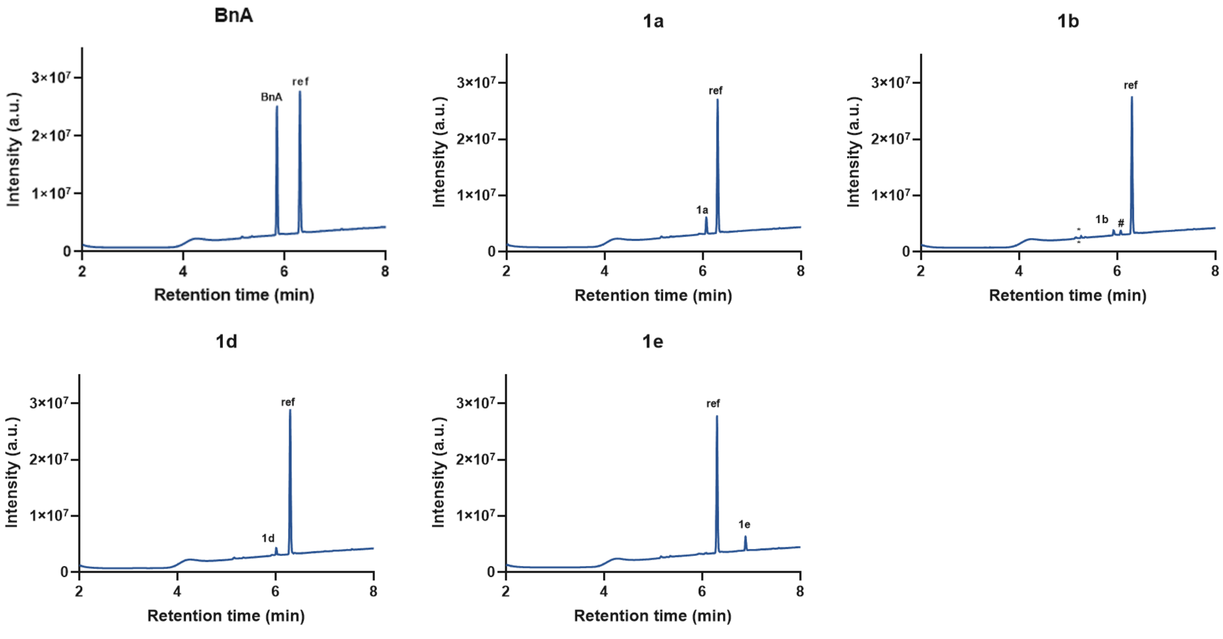

Figure S6: Lysine reactivity of model compounds: UV chromatogram (220-400 nm) of the LC/MS traces of model compounds (200 µM; **BnA**, **1a**, **1b**, **1d** and **1e**) incubated with N-acyl lysine methyl ester (5 mM) and 4-nitrocyano benzene (100 µM) in PBS buffer at pH 8, 37 °C, 4 days. No significant reaction has been observed with model compounds.* hydrolysis product. #**1a** was formed.

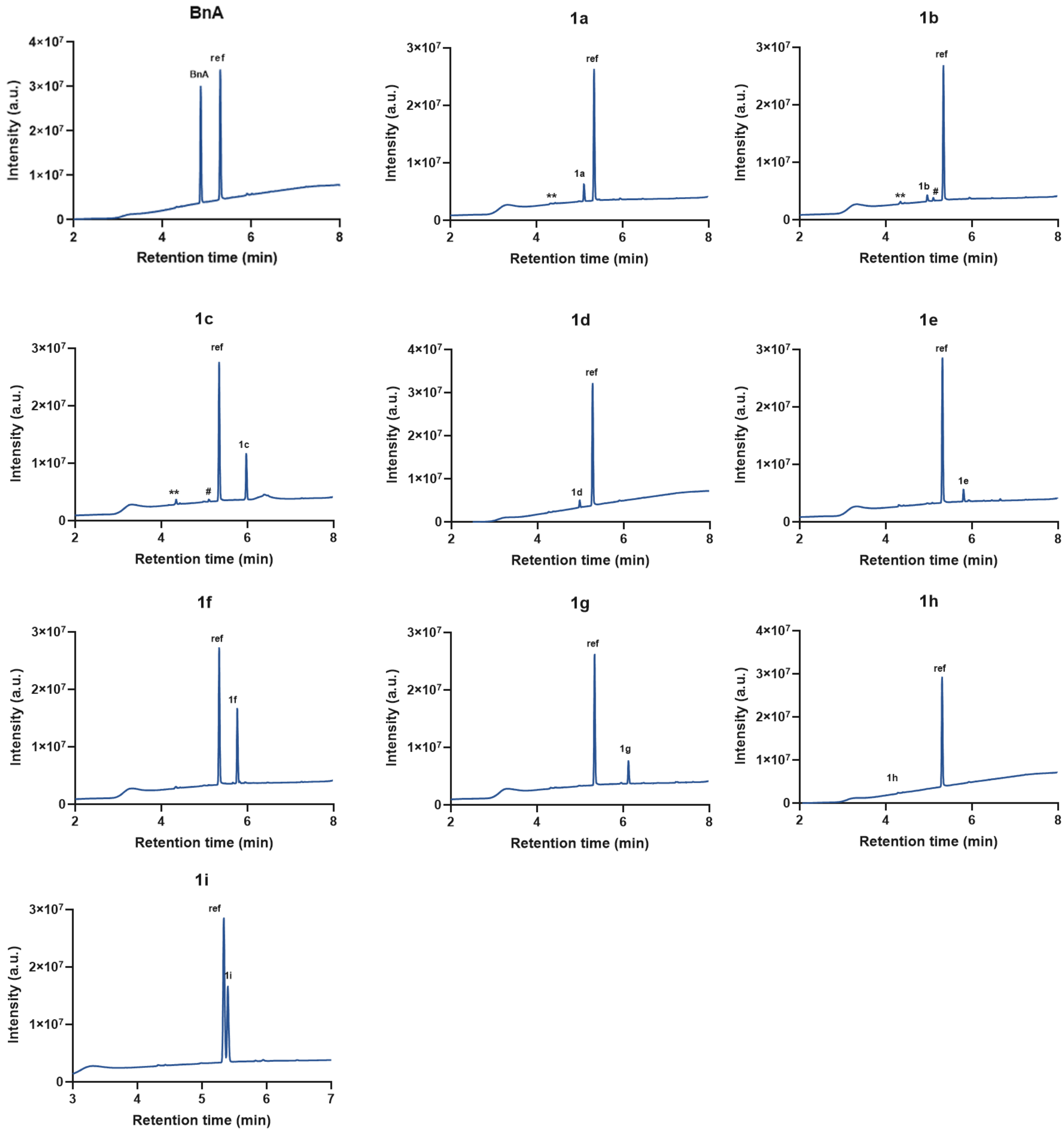

Figure S7: Buffer stability of model compounds. UV chromatogram (220-400 nm) of the LC/MS traces of model compounds (200 µM;**1a**-**1i**) incubated with 4-nitrocyano benzene (100 µM) in PBS buffer at pH 8, 37 °C. The percentage of hydrolysis was quantified using LC/MS at a wavelength of 220 nm. **1a**, **1b**, and **1c** underwent 12, 26, and 51% hydrolysis. A peak at 3.5 min is a contamination of the column. ** hydrolysis product. # For compounds **1b** and **1c**, we detected that sulfonates were substituted with chloride ions.

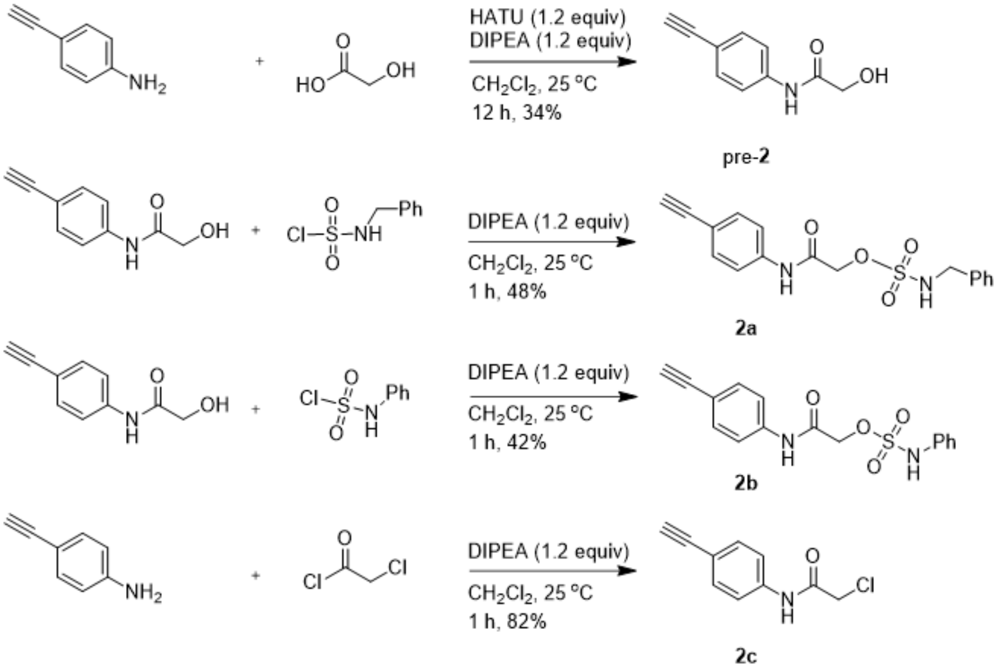

Figure S8: Synthetic schemes of compounds 2a-2c.

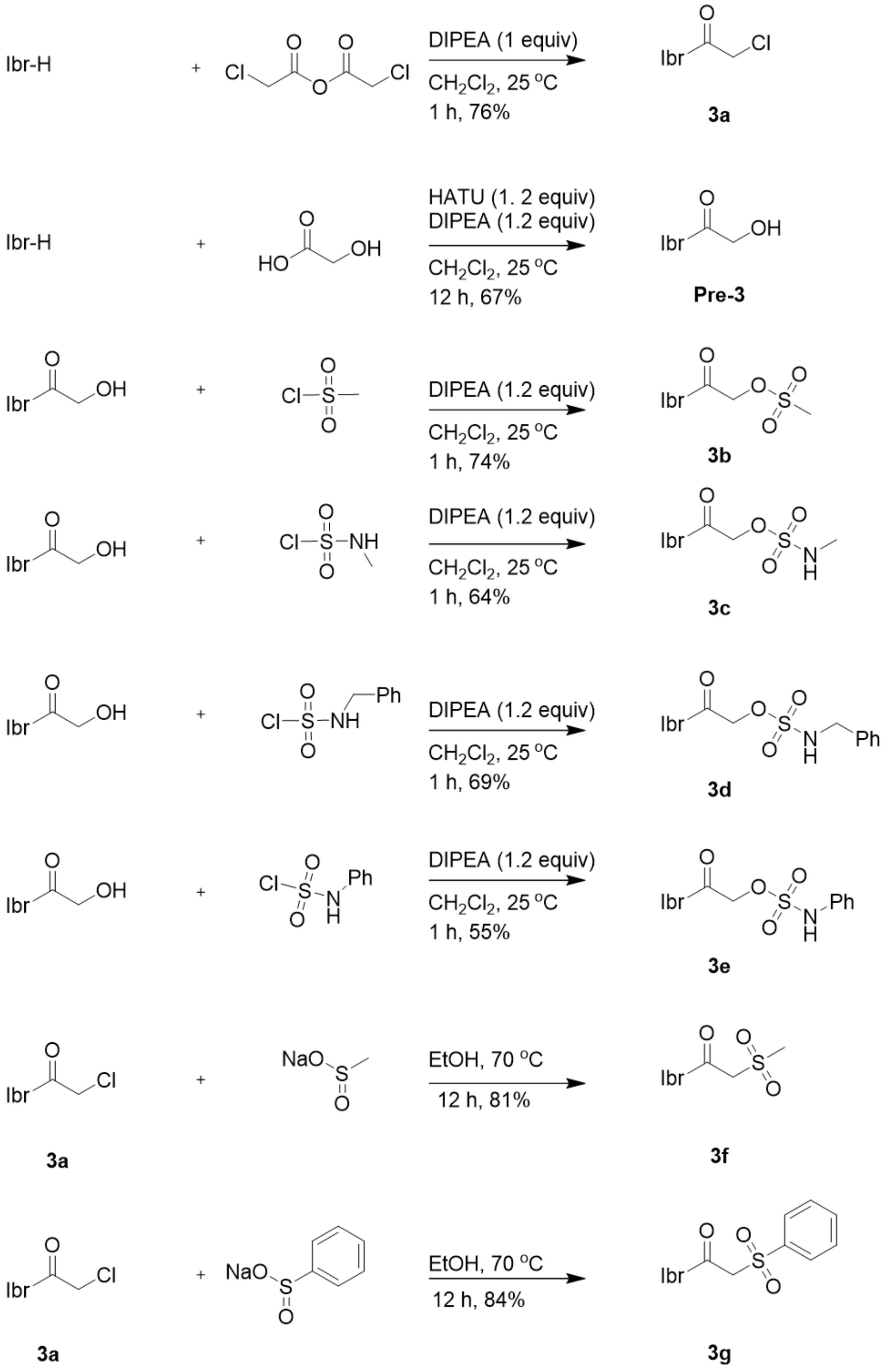

Figure S9: Synthetic schemes of Ibrutinib sulfamate compounds

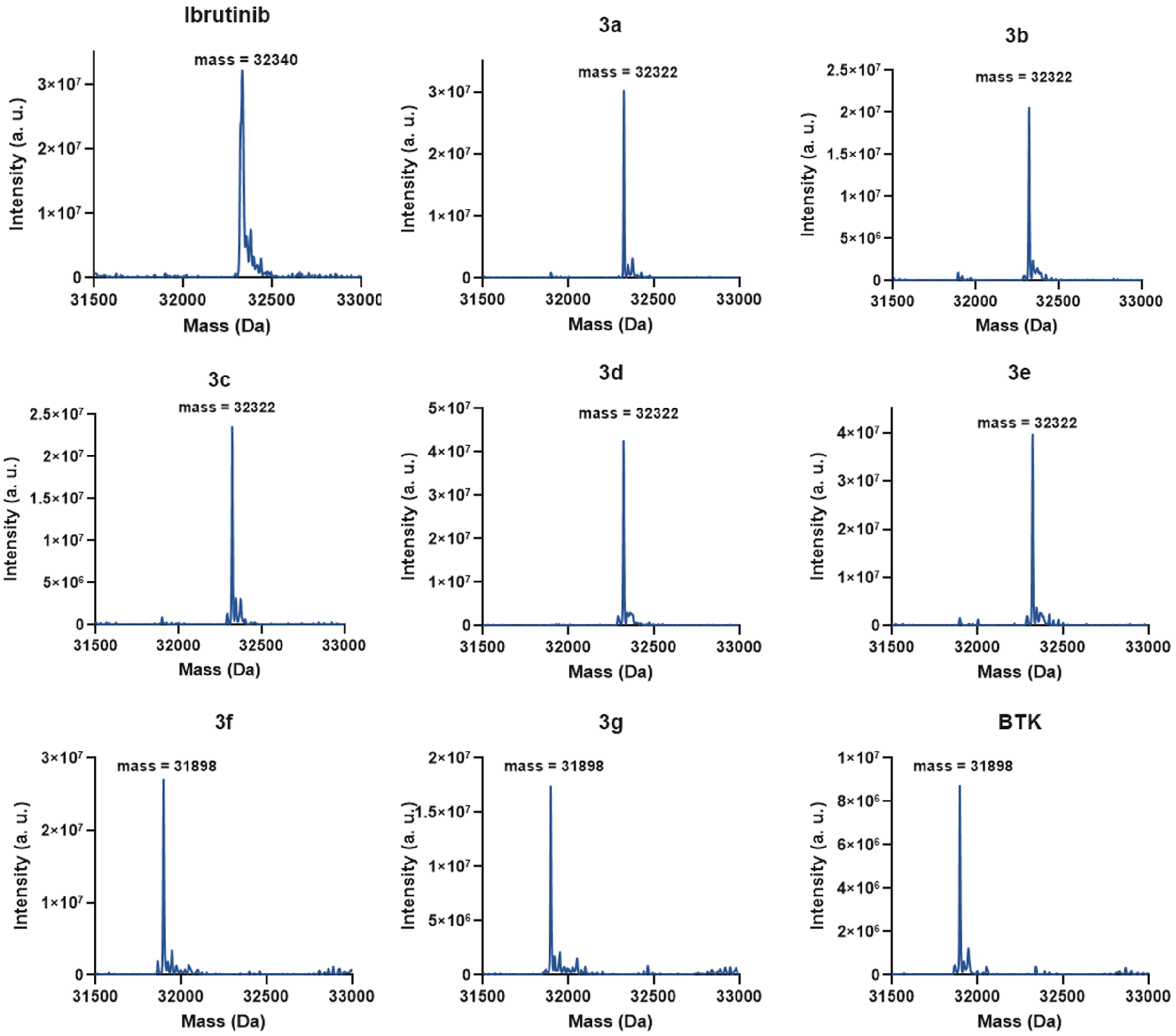

Figure S10: Deconvoluted mass spectra of BTK bound to various compounds. Intact protein LC/MS of 2 µM BTK incubated with 2 µM Ibrutinib derivatives (Ibrutinib, **3a**-**3g**) at pH 8.0, 25 °C. These are the spectra of the final reading of the experiment described in Figure 3C. Compounds **3f** and **3g** show no labeling to BTK.

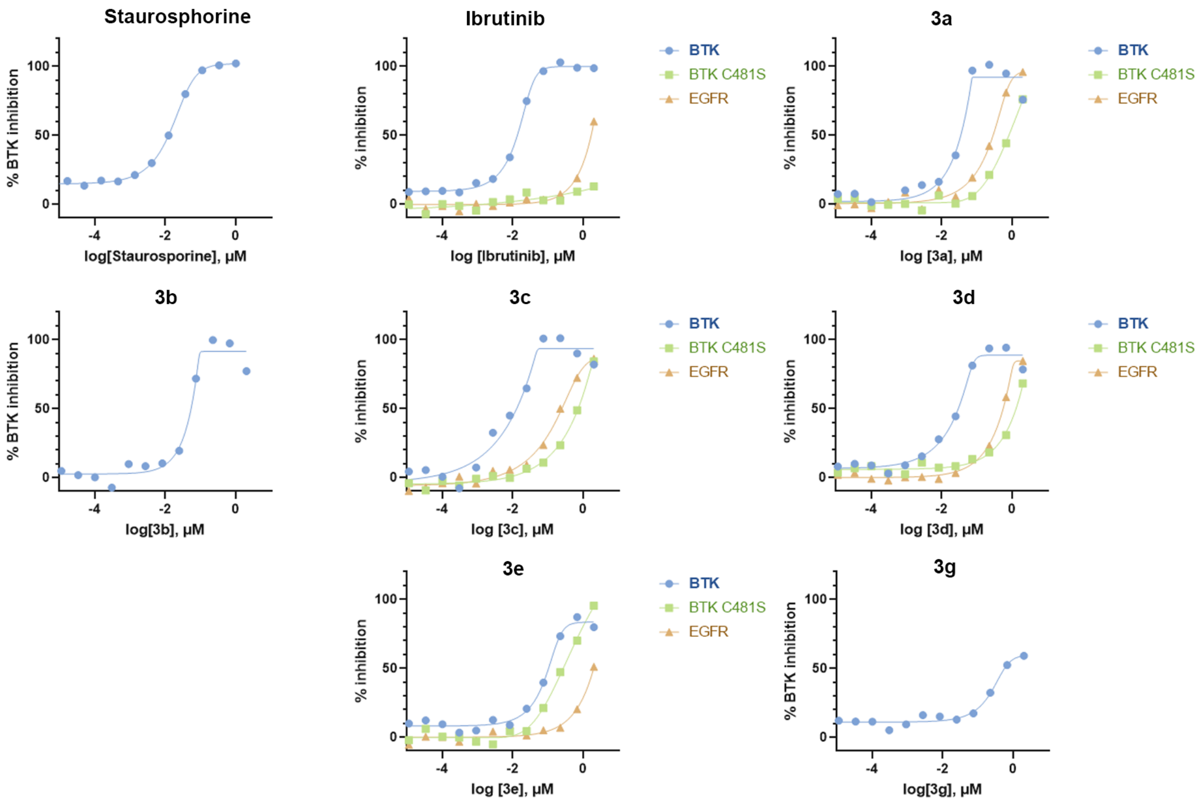

Compound	BTK (IC_{50} μM)	C481S (IC_{50} μM)	EGFR (IC_{50} μM)	C481S/BTK	EGFR/BTK
**Ibrutinib**	0.012	2.000	1.670	160.0	133.6
**3a**	0.029	0.795	0.261	27.0	8.9
**3b**	0.046	-----	-----	-----	-----
**3c**	0.0086	0.740	0.241	85.6	27.9
**3d**	0.024	1.280	0.533	52.8	22.0
**3e**	0.095	0.275	1.960	2.9	20.6
**3g**	0.024	-----	-----	-----	-----

Figure S11. *In vitro* kinase activity assays of Ibrutinib analogs. Activity assay was performed by Nanosyn (Santa Clara, CA) with 0.5 nM BTK and 5 μM ATP with all the Ibr-sulfamates (**3a**-**3g**). Ibrutinib, **3a**, **3c**, **3d,** and **3e** were also assayed against the BTK C481S mutant and EGFR. The IC_{50} values and selectivity ratios are reported in the table.

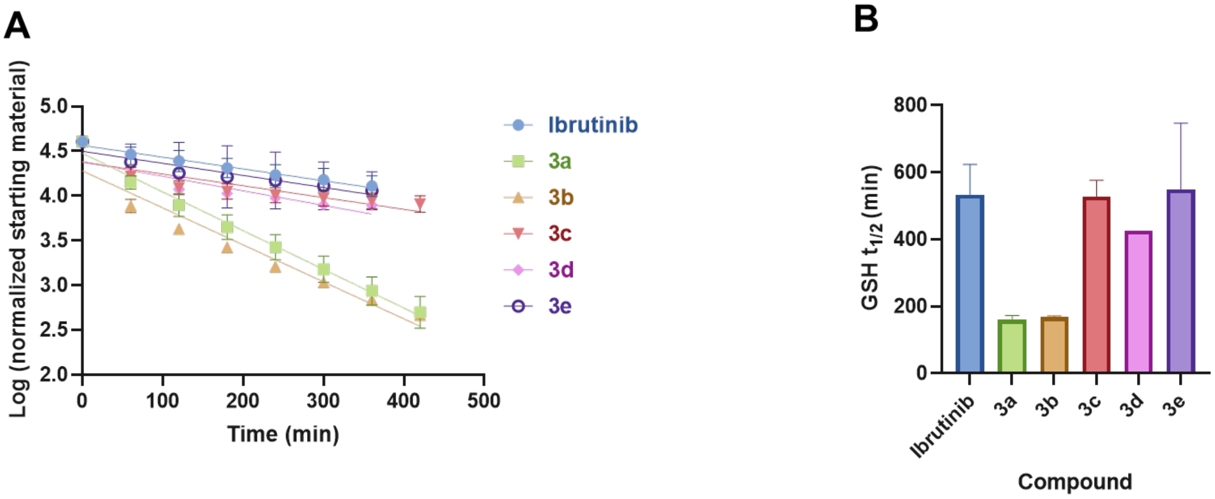

Figure S12: GSH reactivity of Ibrutinib derivatives: **A**. Rates of depletion of Ibrutinib derivatives (**3a**-**3e**) in a reaction between 100 µM compound and 5 mM GSH in PBS buffer at pH 8, 14 °C (n=2) for 6 or 7 h. **B**. Extrapolated GSH half-lives ($t_{1/2}$) of the compounds.

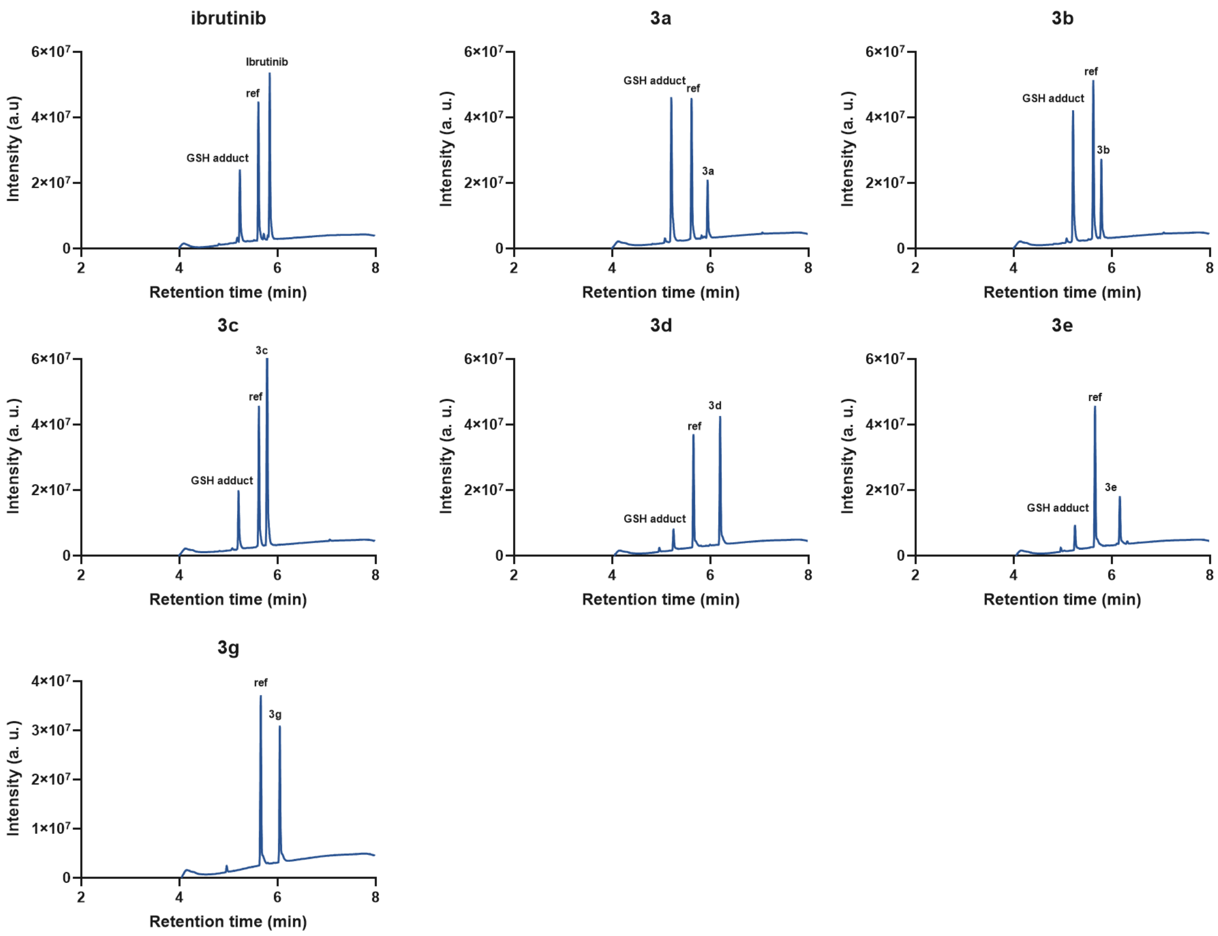

Figure S13. GSH reactivity assay with Ibrutinib analogs. UV chromatogram (220-400 nm) of the LC/MS analysis of 5 mM GSH incubated with 100 µM of compounds **3a**-**3g** at the last measured point of the GSH $t_{1/2}$ experiment (Figure S12).

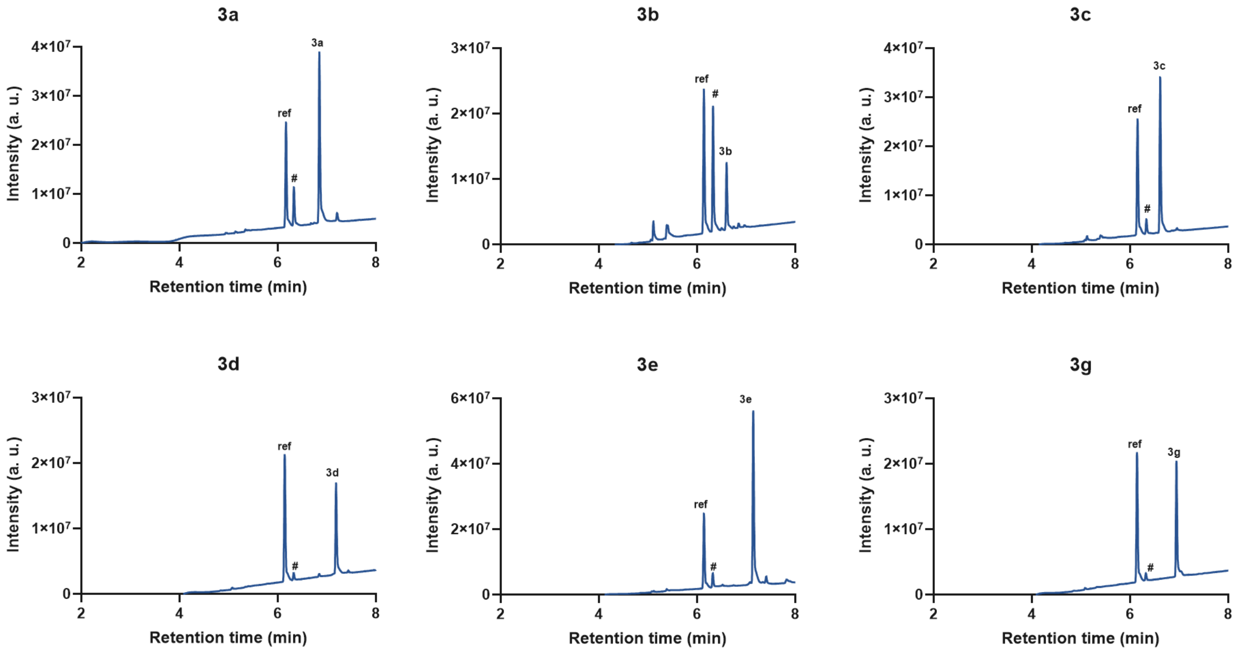

Figure S14. Ibrutinib analogs show prolonged buffer stability. UV chromatogram (220-400 nm) of the LC/MS analysis of 100 µM of compounds (**3a**-**3g**) incubated with a reference (100 µM) in PBS buffer at pH 8, 37 °C for four days. **3a** and **3b** underwent 25 and 75% hydrolysis, whereas other sulfamates underwent <5% hydrolysis. # hydrolysis product

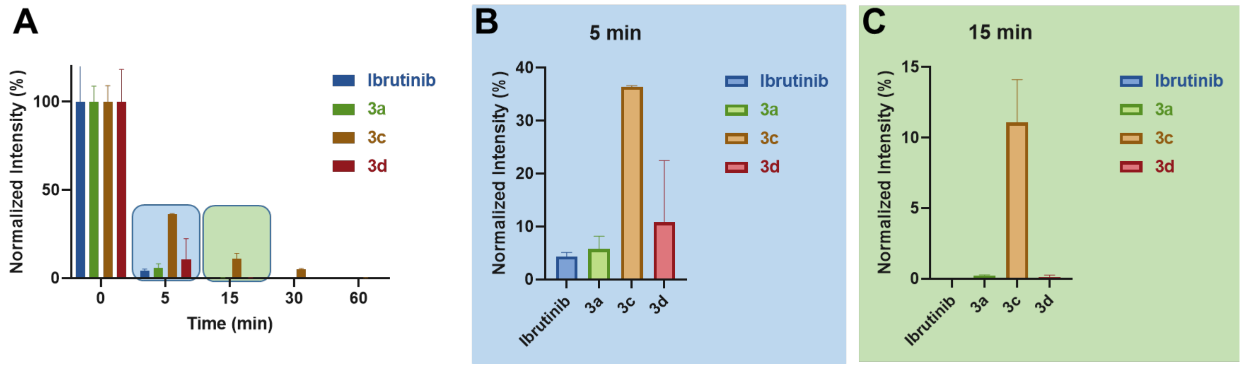

Figure S15: Metabolic stability assay for Ibrutinib sulfamates. A. Time-dependent metabolic stability of Ibrutinib, **3a**, **3c,** and **3d**. **B, C.** Zoom-in of the 5 min and 15 min time points, respectively (n = 2).

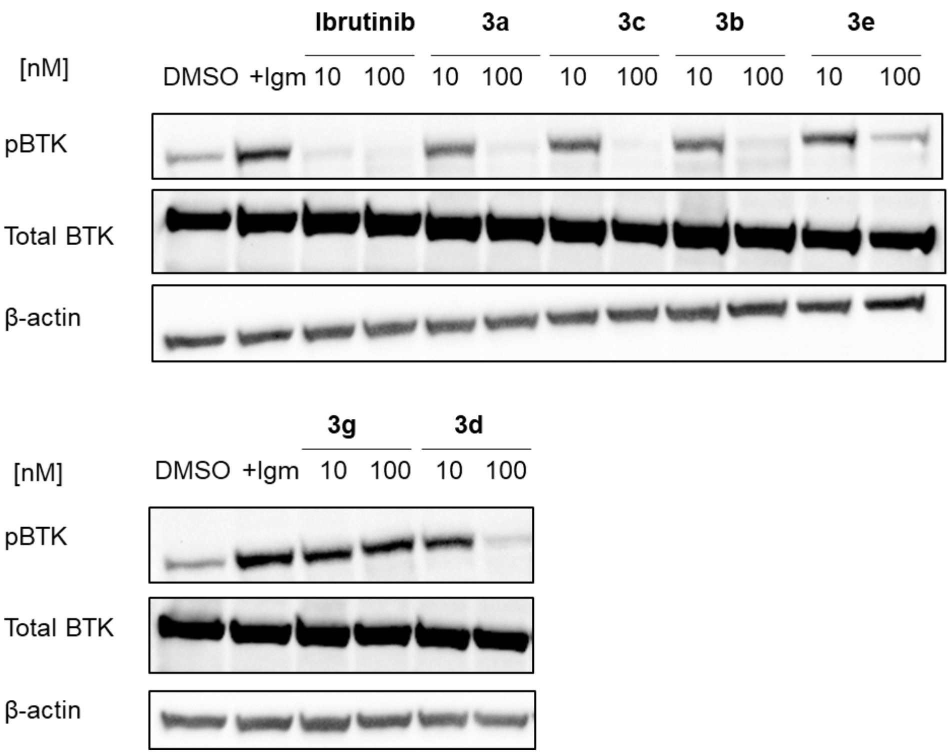

Figure S16: Cellular pBTK inhibition assays. Western blots show inhibition of BTK phosphorylation, after stimulation of cells with an anti-IgM antibody, by Ibrutinib and all sulfamates (**3a**-**3e**) at concentrations of 10 nM and 100 nM.

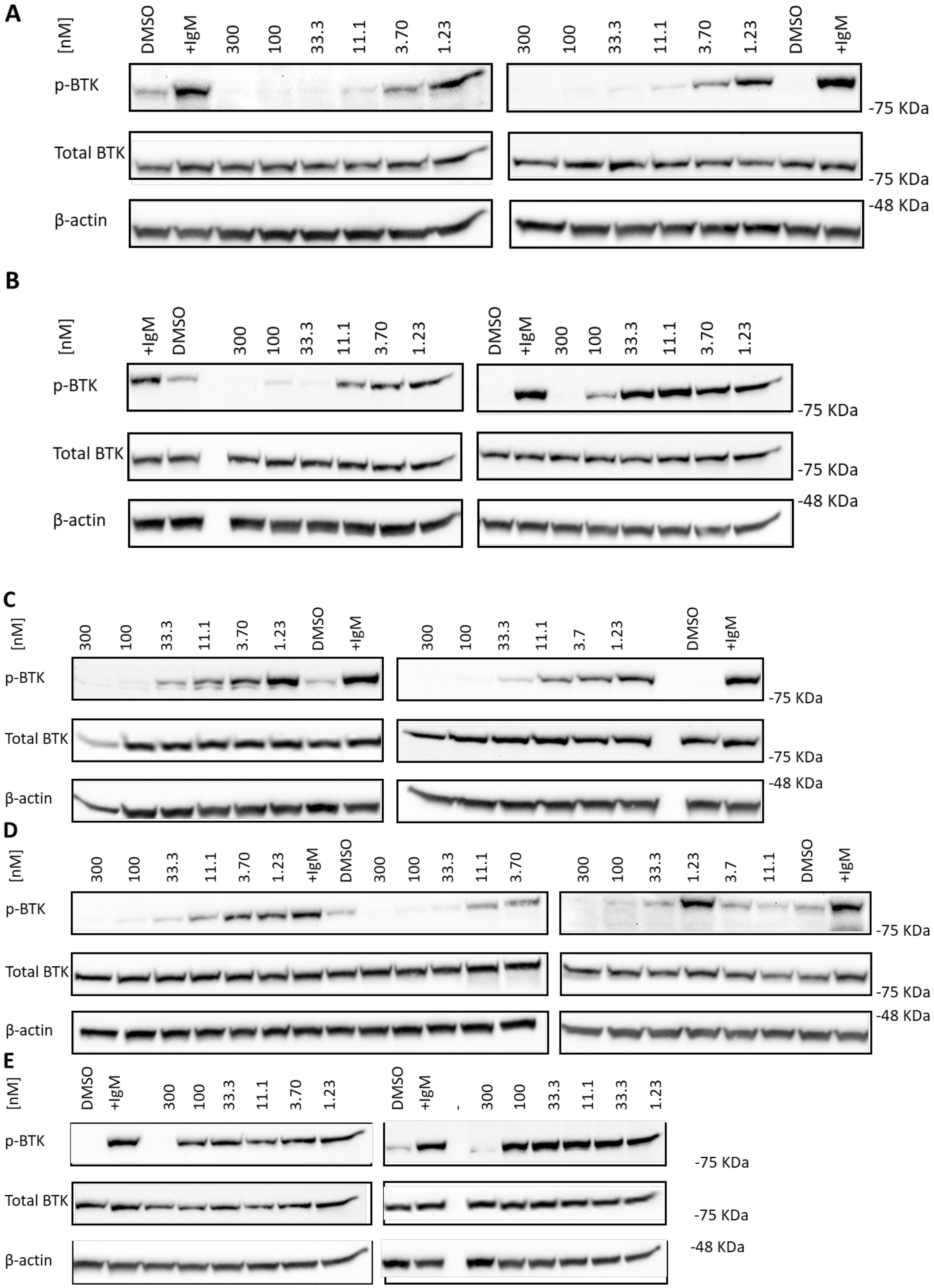
A
[nM]
DMSO
+IgM
300
100
33.3
11.1
3.70
1.23
300
100
33.3
11.1
3.70
1.23
DMSO
+IgM
p-BTK
-75 KDa
Total BTK
-75 KDa
β-actin
-48 KDa
B
[nM]
+IgM
DMSO
300
100
33.3
11.1
3.70
1.23
DMSO
+IgM
300
100
33.3
11.1
3.70
1.23
p-BTK
-75 KDa
Total BTK
-75 KDa
β-actin
-48 KDa
C
[nM]
300
100
33.3
11.1
3.70
1.23
DMSO
+IgM
300
100
33.3
11.1
3.7
1.23
DMSO
+IgM
p-BTK
-75 KDa
Total BTK
-75 KDa
β-actin
-48 KDa
D
[nM]
300
100
33.3
11.1
3.70
1.23
+IgM
DMSO
300
100
33.3
11.1
3.70
300
100
33.3
1.23
3.7
11.1
DMSO
+IgM
p-BTK
-75 KDa
Total BTK
-75 KDa
β-actin
-48 KDa
E
[nM]
DMSO
+IgM
300
100
33.3
11.1
3.70
1.23
DMSO
+IgM
-
300
100
33.3
11.1
33.3
1.23
p-BTK
-75 KDa
Total BTK
-75 KDa
β-actin
-48 KDa

Figure S17: Cellular pBTK inhibition assays with a full dose response. Western blots show dose-dependent inhibition of BTK phosphorylation by **Ibrutinib** (**A**), **3a** (**B**), **3c** (**C**), **3b** (**D**), **3d** (**E**). See quantification in Fig. 4A.

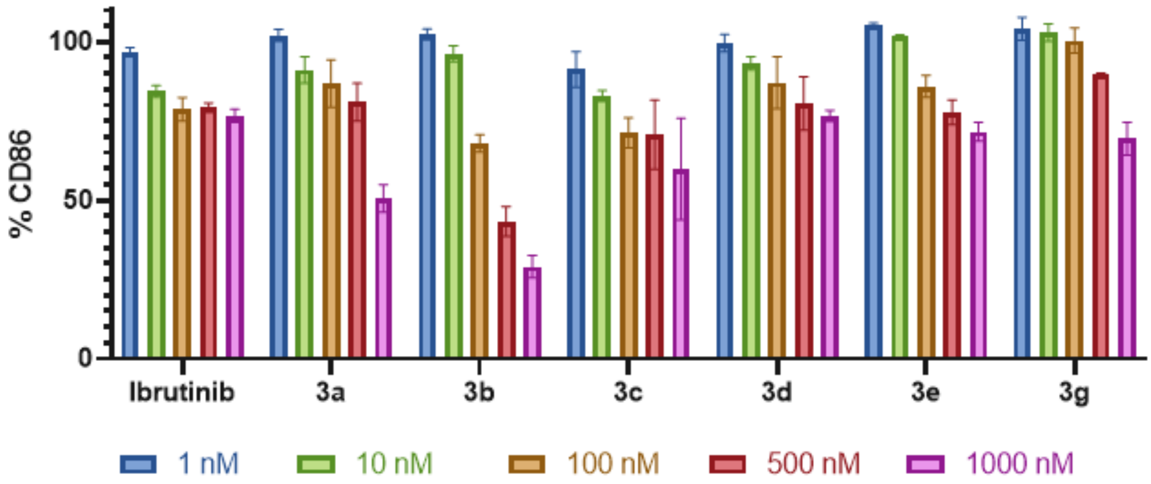

Figure S18: Dose-dependent inhibition of primary mouse B cell response (as measured by %CD86 expression) after anti-IgM-induced activation and treatment with Ibrutinib analogs (**3a-3g**) for 24 h (n=3; error bars indicate standard deviation).

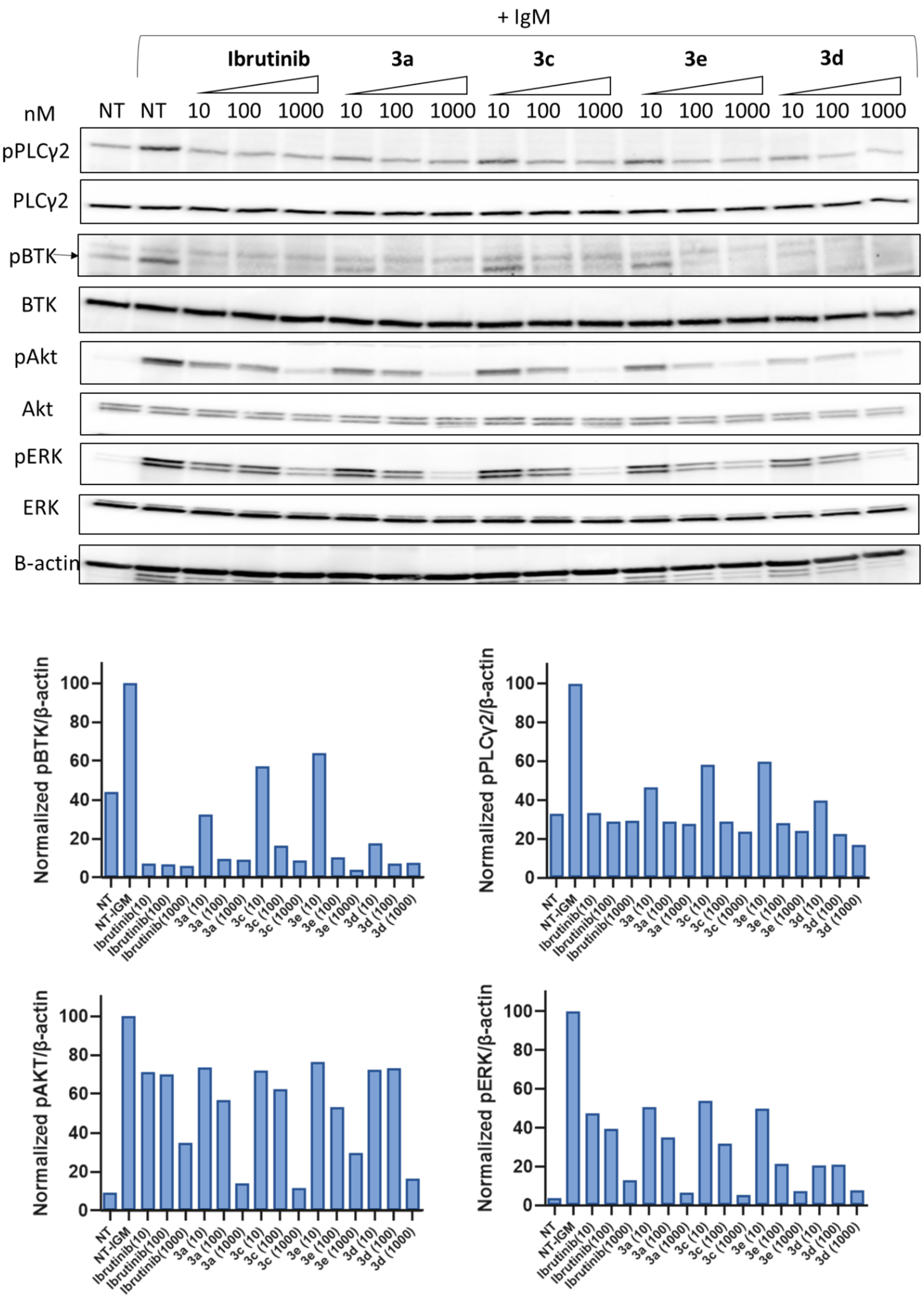

Figure S19: Inhibition of BCR signaling in CLL patient samples. Western blots show inhibition of pBTK and its downstream pathways (pPLCγ2, pAkt, and pERK) by Ibr sulfamates (Ibrutinib**, 1a, 1c, 1e, and 1d**) at 10, 100, 1000 nM in CLL patient samples. Quantification of pBTK, pPLCγ2, pAkt, and pERK levels (normalized to β-actin) was shown in Bar graphs.

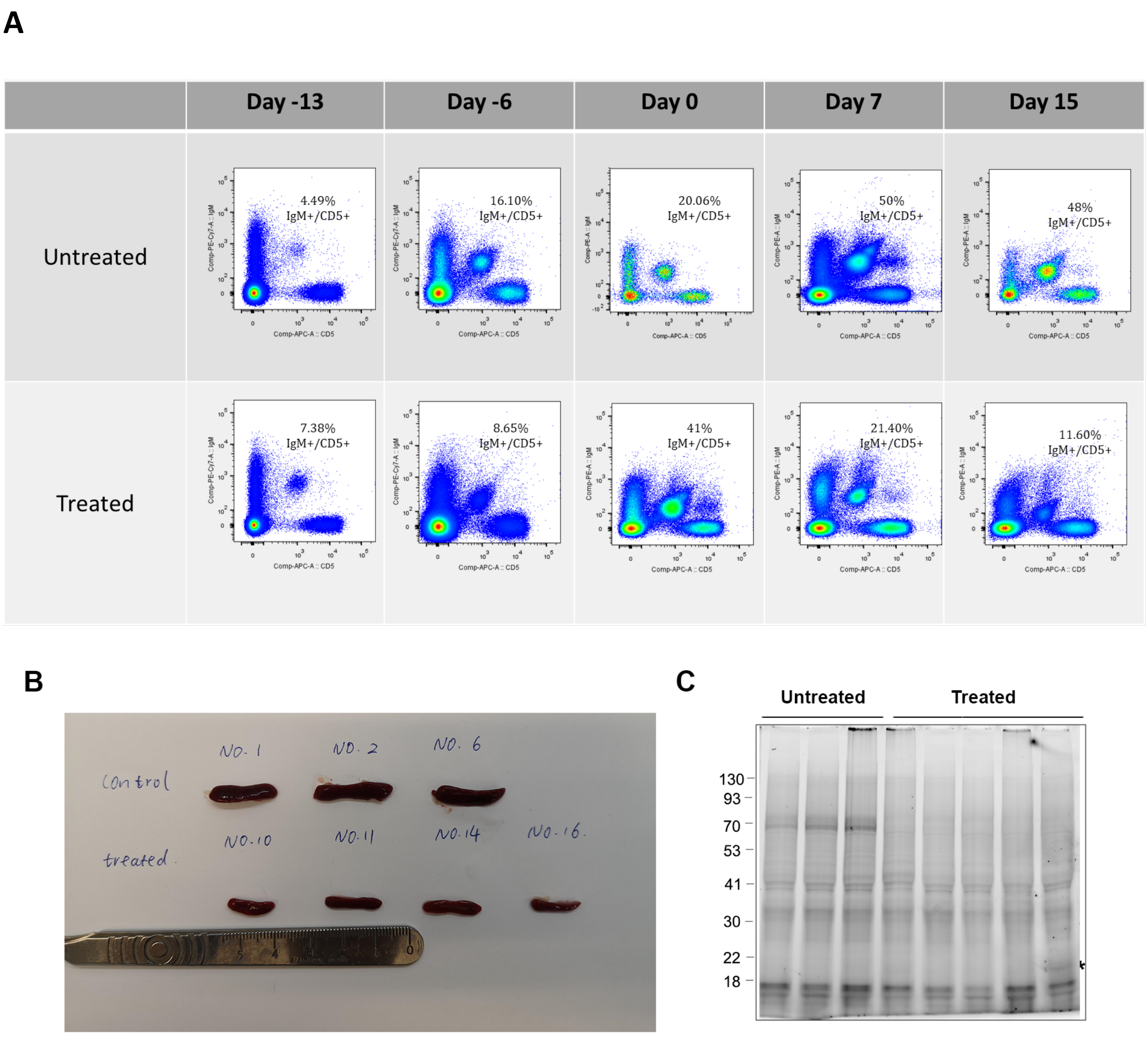

Figure S20: Treatment with Ibr-sulfamates reduces spleen size in a mouse model of CLL. A. Representative FACS plots show the IgM+/CD5+ population in treated and untreated mice. **B.** Mice spleens extracted at the end of the experiment described in Figure 4D,E. Mice treated with **3c** show decreased spleen size compared to vehicle-treated control mice. **C.** Cellular engagement of compound **3c** *in vivo*. The isolated spleens were extracted with RIPA buffer and incubated with 'probe-4' for 1 h, followed by a "click" reaction with TAMRA-azide in lysate before imaging.

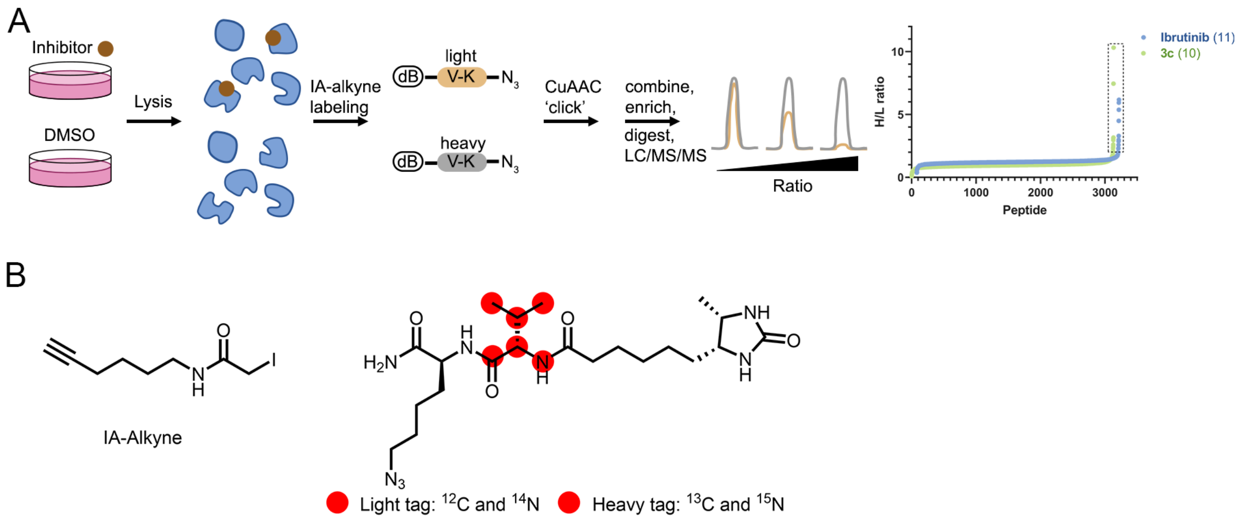

Figure S21: (A) Schematic description of our isoDTB-ABPP protocol. (B) Structures of IA-alkyne and desthiobiotin–valine–azidolysine light or heavy peptide tags.

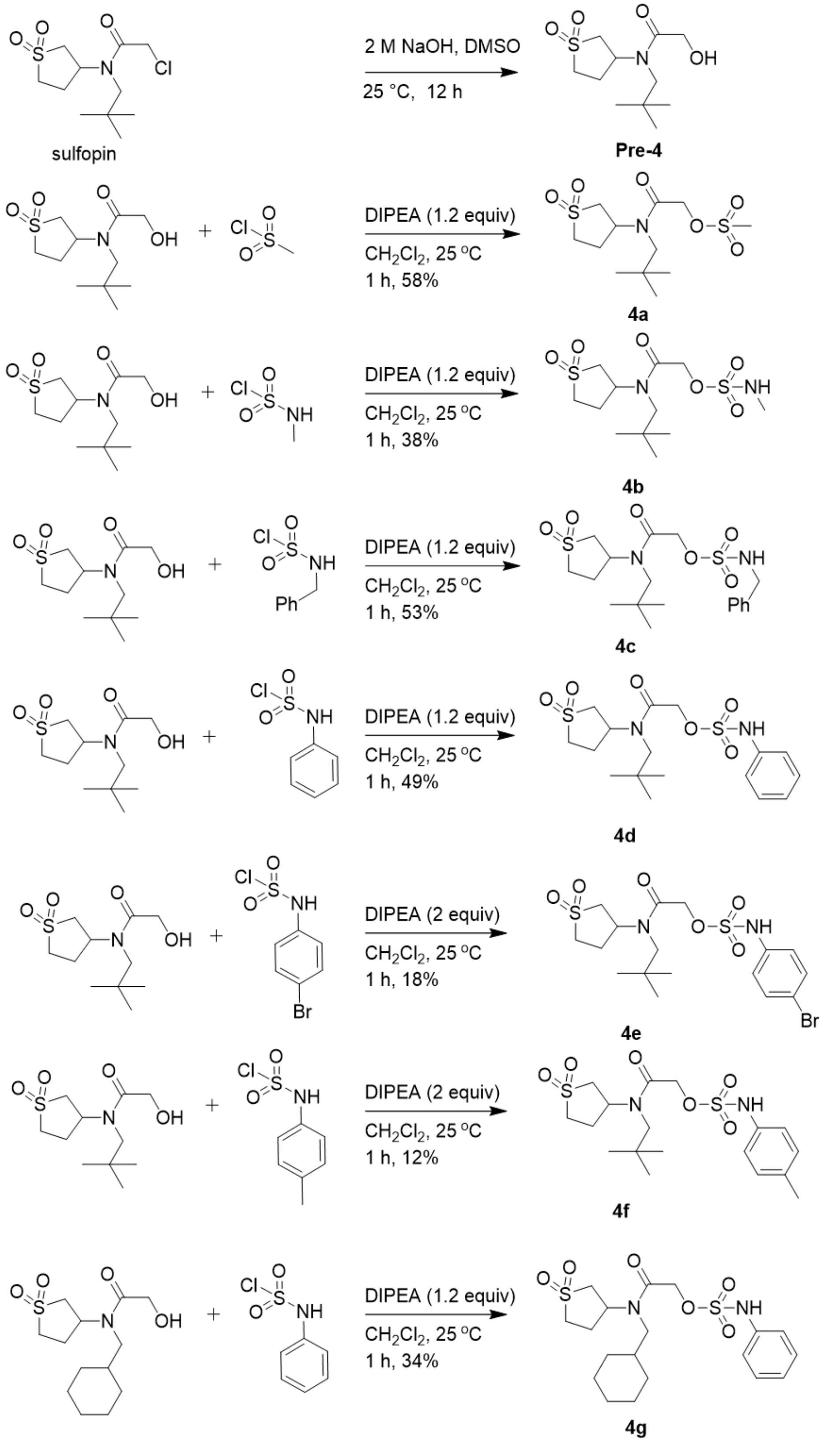

Figure S22: Synthetic schemes of Sulfopin sulfamate analogs.

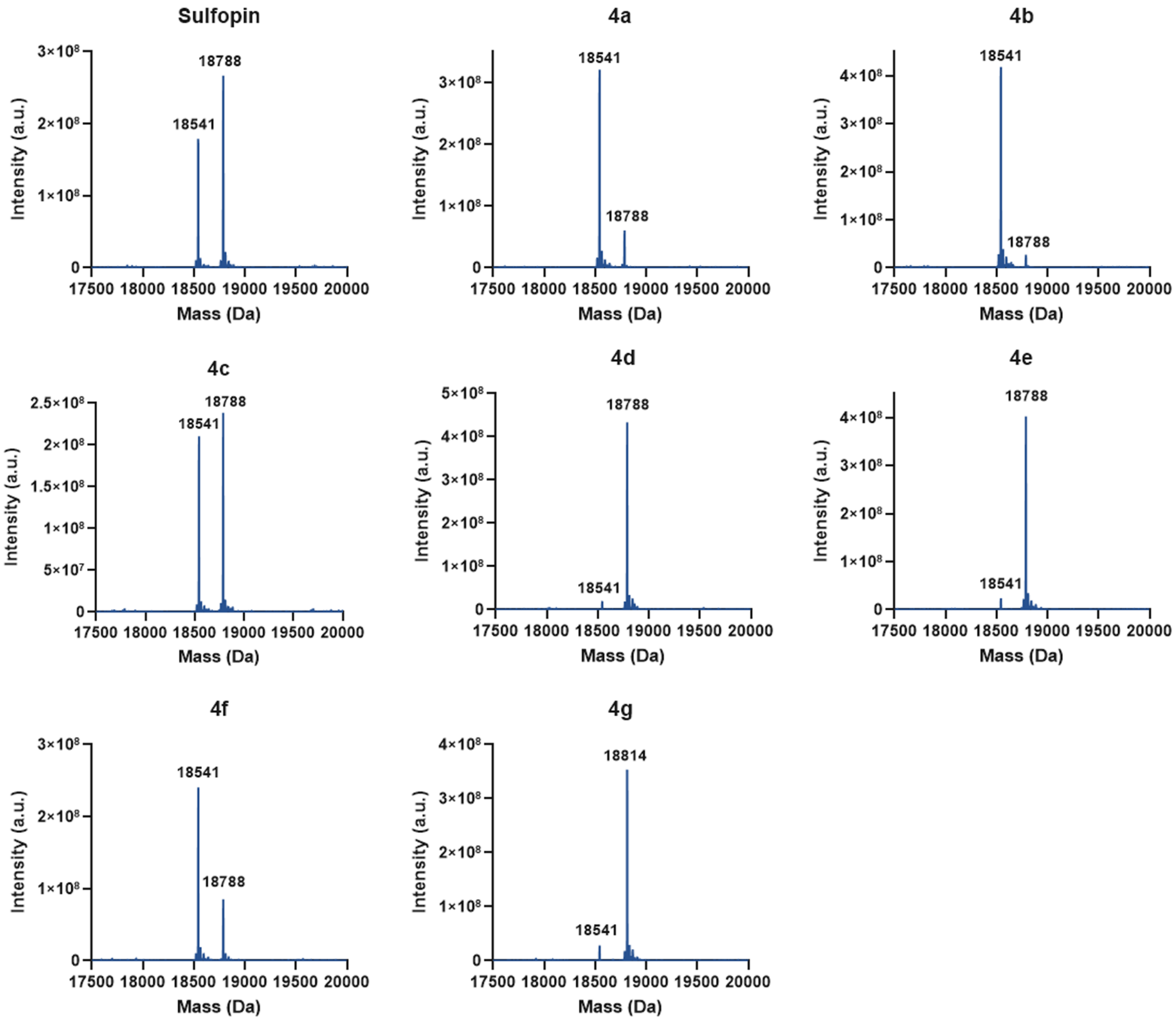

Figure S23: Pin1 labeling with Sulfopin analogs. Deconvoluted mass spectra (intact protein LC/MS) of 2 µM Pin1 incubated with 2 µM Sulfopin sulfamates (**4a**-**4g**) at pH 7.5, 25 °C after 1 h.

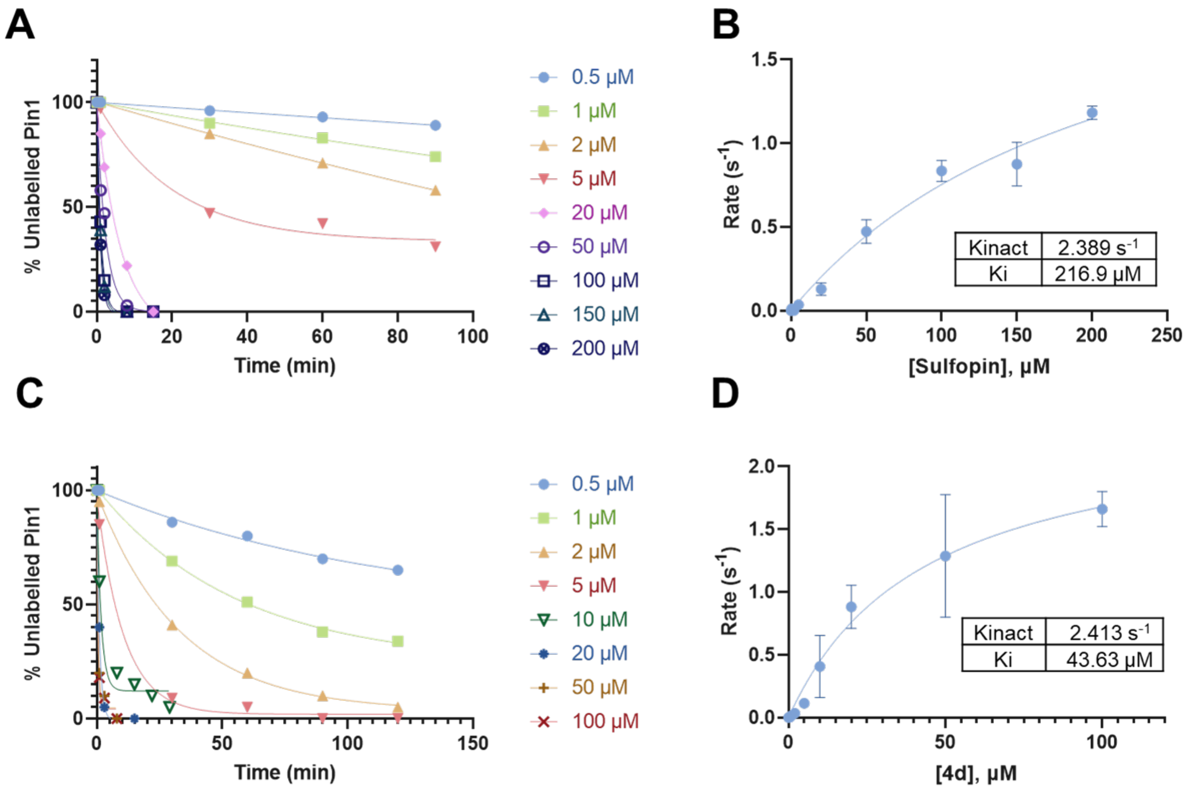

Figure S24: Sulfopin sulfamate 4d shows a five-fold higher binding affinity to Pin1 than Sulfopin: A, **C** Depletion of unlabelled Pin1 (0.5 μM) when incubated with **A.** Sulfopin (0.5, 1, 2, 5, 20, 50, 100, 150, 200 μM) or **C. 4d** (0.5, 1, 2, 5, 10, 20, 50, 100 μM) in 100 mM Tris buffer at pH 7.4, 14 °C over time. The reaction was monitored by LC/MS. **B**, **D**. The rate constants extrapolated from **A** and **C**, respectively, as a function of the concentration of Sulfopin and **4d**. Kinetic parameters were obtained by fitting this data to a Michaelis-Menten saturation curve.

A

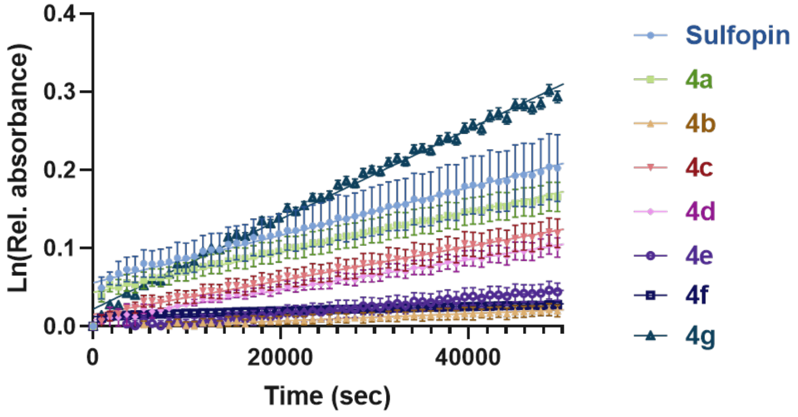

B

Compound	k, $M^{-1}s^{-1}$
**Sulfopin**	**0.0303**
**4a**	**0.0285**
**4b**	**0.0064**
**4c**	**0.0288**
**4d**	**0.0230**
**4e**	**0.0073**
**4f**	**0.0046**
**4g**	**0.0682**

Figure S25: Thiol reactivity of Sulfopin analogs. A. Loss of DTNB absorbance over time as a function of intrinsic thiol reactivity of Sulfopin analogs. **B.** The second-order rate constants for the reaction as extracted from the DTNB thiol reactivity assay (see methods).

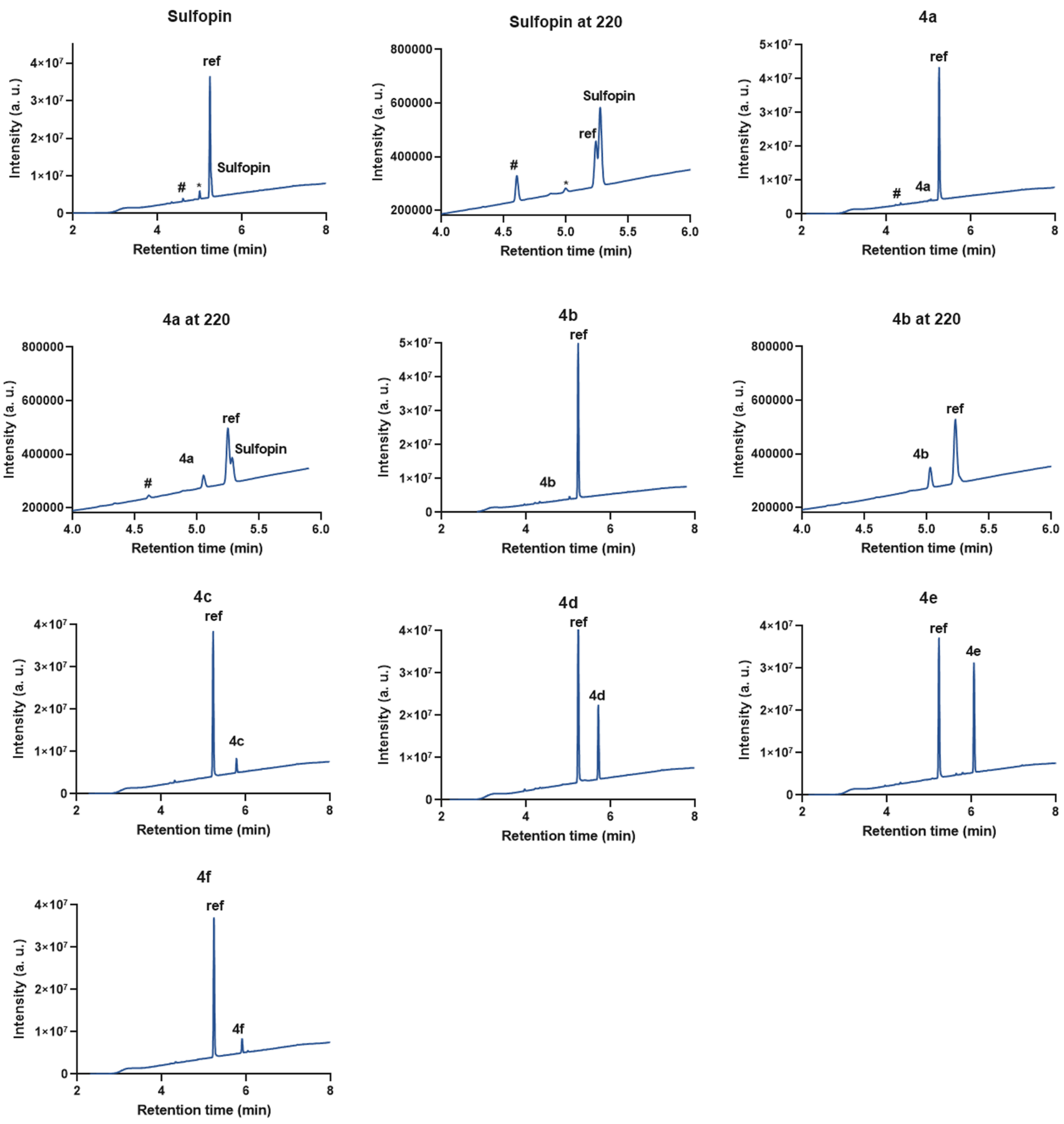

Figure S26: Sulfopin analogs show prolonged buffer stability. UV chromatogram (220-400 nm) of the LC/MS analysis of 200 µM of compounds (**4a**-**4f**) incubated with a reference (100 µM) in the PBS buffer at pH 8, 37 ^{o}C for four days. For compounds **Sulfopin**, **4a,** and **4b**, spectra at 220 nm were taken to show the peaks. **Sulfopin** and **4a** underwent 30 and 15% hydrolysis, respectively. Compound **4a** shows a Sulfopin-like peak, possibly due to displacement of sulfonate group by chloride ion in the buffer. # hydrolysis product * unidentified peak.

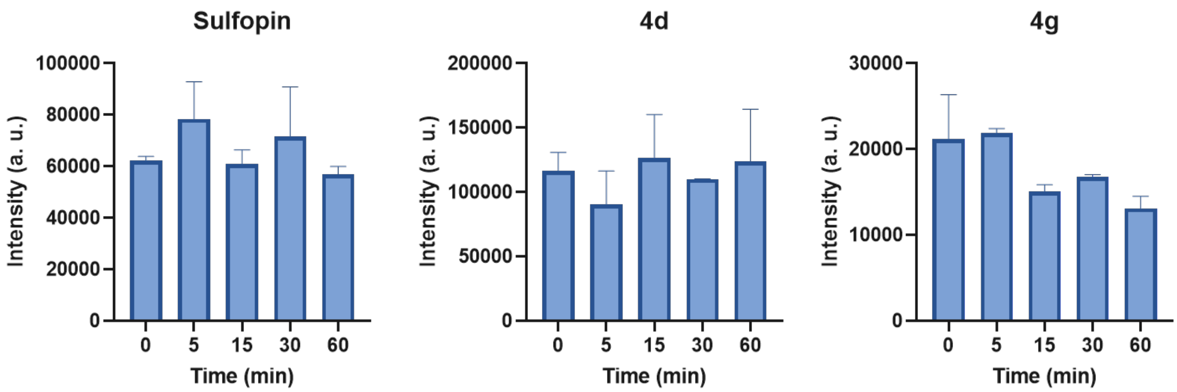

Figure S27: Sulfopin sulfamates show high metabolic stability: Compounds (**Sulfopin**, **4d**, and **4g**) were incubated in liver microsomes and injected to LC/MS/MS at indicated time points. The peak intensities of the compounds were measured in UV spectra.

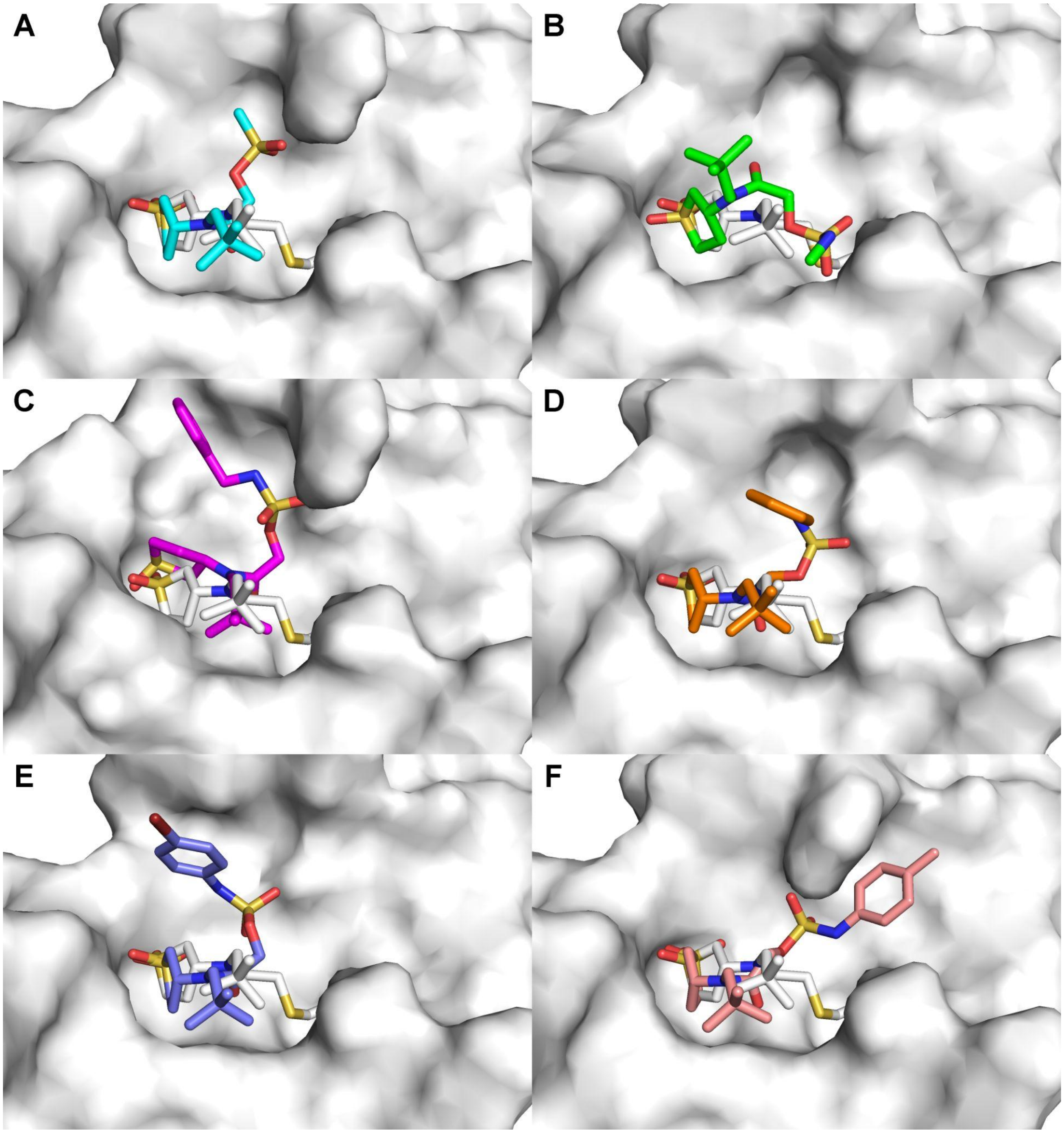

Figure S28: Molecular modeling of Sulfopin sulfamate analogs. We used RosettaLigand to model the non-covalent binding of Sulfopin analogs: **A. 4a** (cyan) **B. 4b** (green) **C. 4c** (magenta) **D. 4d** (orange) **E. 4e** (purple) **F. 4f** (pink) to Pin1 (PDB: 6VAJ; white). Covalently bound Suflopin (white) is shown for reference with each model. The docking suggests there is room to accommodate the sulfamate modification in the enzyme binding site next to the active pocket. In several of these models, both the sulfamate and the additional modification on the amine side make additional interactions with Pin1.

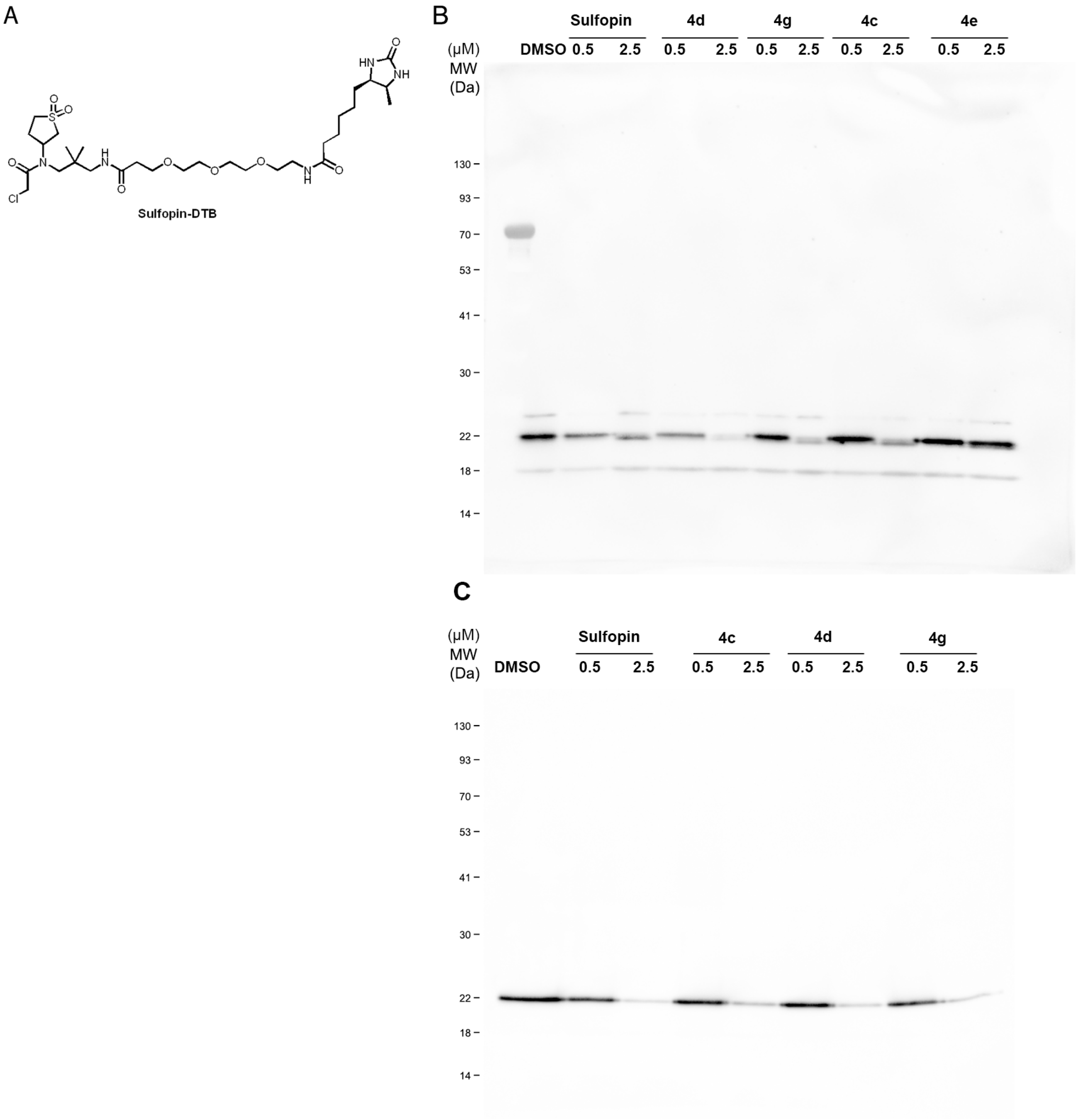

Figure S29: Sulfopin sulfamates show cellular engagement with Pin1: A. Chemical structures of Sulfopin-DTB probe. **B.** Cellular engagement of the Sulfopin sulfamates. OCI-AML2 cells were treated with DMSO, Sulfopin, and sulfamates (**4c**, **4d**, **4e**, **4g**) at 0.5, 2.5 µM concentration for 4 hours. Lysates were prepared and incubated with Sulfopin DTB probe (1 µM) and then pulled down using streptavidin beads before running a western blot against Pin1.

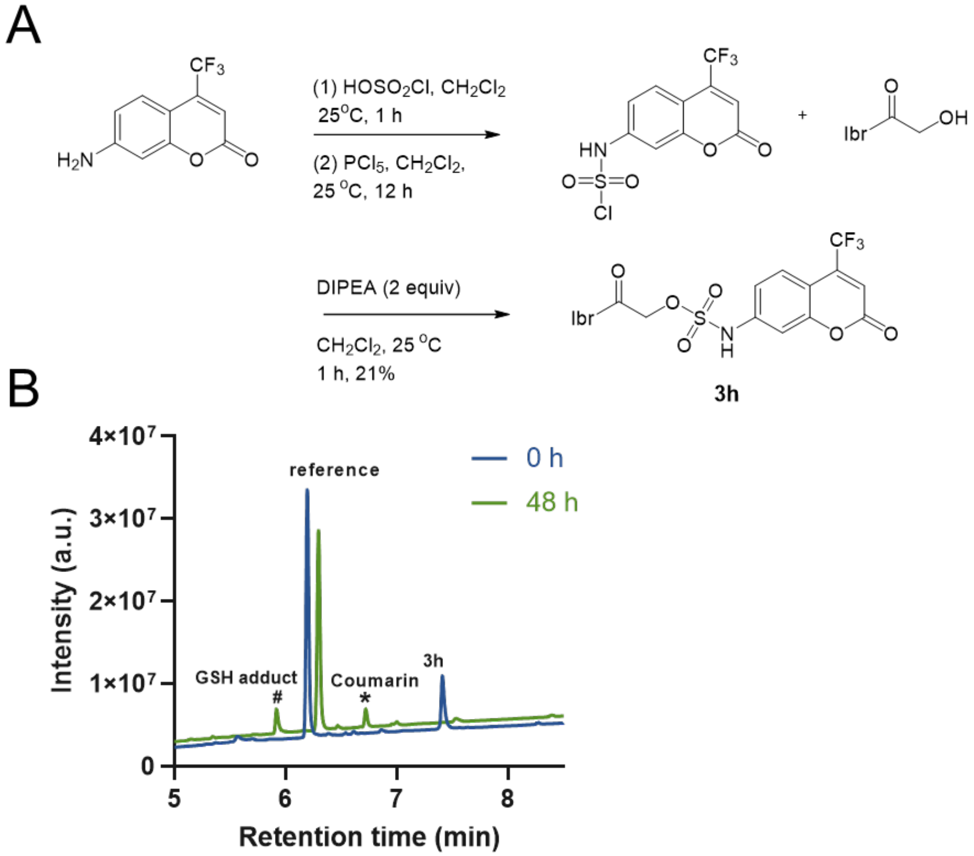

Figure S30: A CoLDR sulfamate probe. A. Synthesis of CoLDR probe **3h B.** LC/MS trace of the reaction of compound (100 µM) with 5 mM GSH at pH 8, 37 °C. 4-nitrocyano benzene has been used as a reference. The spectrum shows the formation of GSH adduct with the release of coumarin moiety.

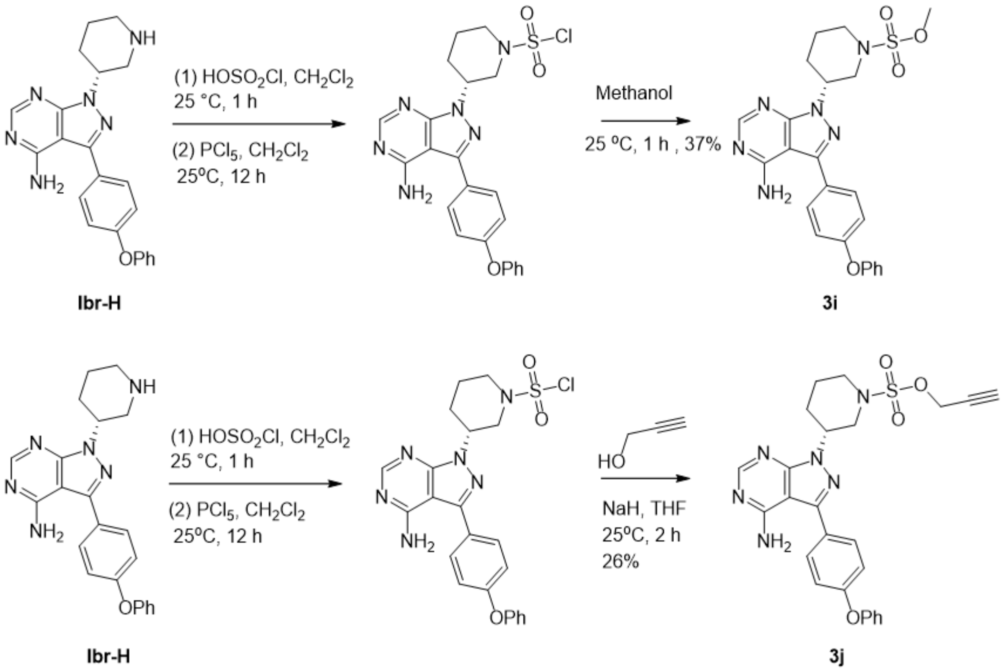

Figure S31: Synthesis scheme of BTK labeling probes 3i and 3j.

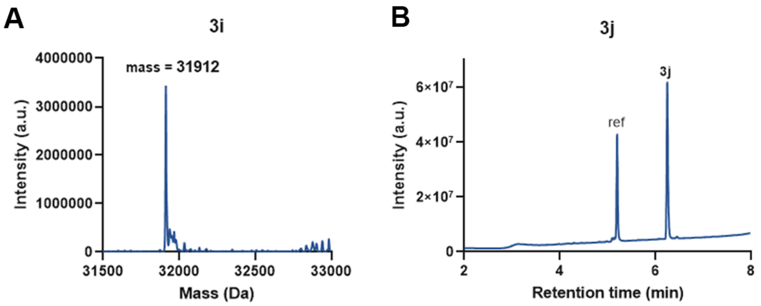

Figure S32. Labeling and GSH reactivity assay of CoLDR probes. A. Deconvoluted MS spectra (intact protein LC/MS) of 2 µM BTK incubated with 200 µM **3i** at pH 8.0, 25 °C, 40 min. **B.** UV spectra (220-400 nm) of the LC/MS analysis of 5 mM GSH incubated with 100 µM of compounds **3j** at the last measured point of the GSH $t_{1/2}$ experiment, similar to Figure S13.

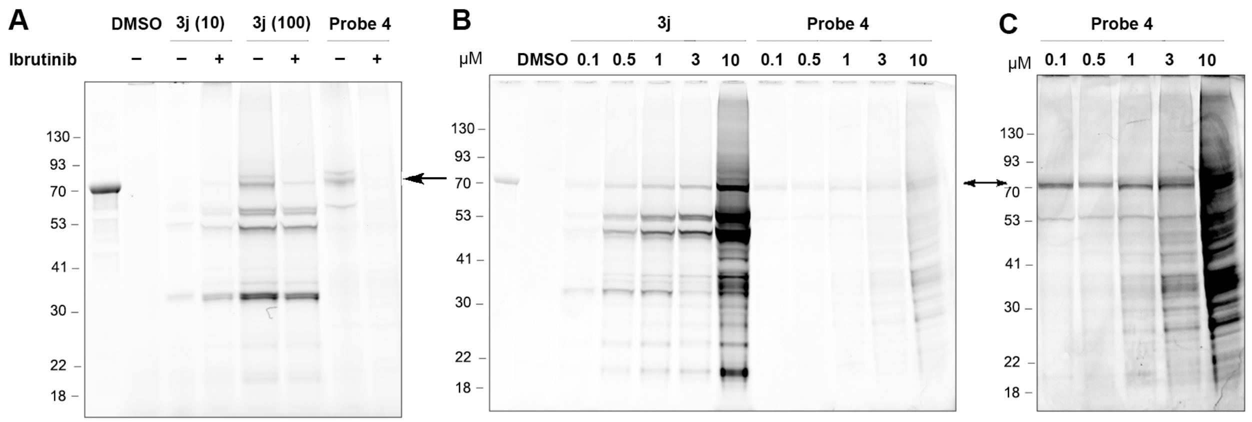

Figure S33: In-gel fluorescence characterization of compound 3j. A. Cellular labeling profile of **3j** after 2 h of incubation with Mino cells. Competition experiment of **3j** & Ibrutinib-alkyne ('**probe 4**') with Ibrutinib. Cells were preincubated for 30 min with either 0.1% DMSO or 1 µM Ibrutinib, followed by 2 h of incubation with 10 or 100 nM **3j** or 200 nM **probe 4**. The samples were further reacted with TAMRA-azide in lysate before imaging. **B.** Dose-dependent labeling of cellular BTK by **3j** and **probe 4** after incubation for 2h in Mino cells. An arrow indicates BTK's MW. **C.** The five rightmost lanes from panel **B.** with adjusted brightness show that probe 4 also binds many off-targets at higher concentrations, but its overall fluorescence signal is lower.

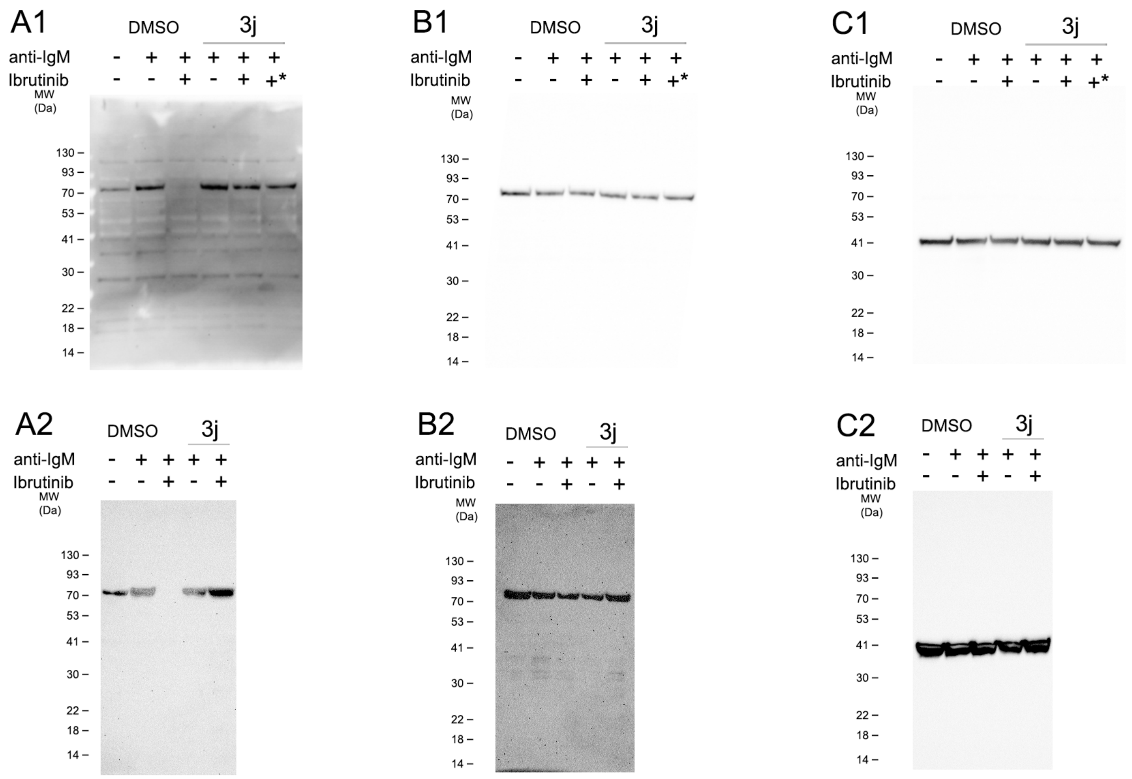

Figure S34. Labeling BTK with CoLDR probes does not inhibit its activity in cells. Mino cells were incubated for 2 h with either DMSO or 1 µM **3g** and then incubated for 45 min with Ibrutinib (100 nM). The cells were washed again before induction of BTK activity by anti-IgM. A1, A2(phospho BTK), B1, B2 (total BTK), C1, C2 (β-actin) show western blots for BTK activity assay. * 1 µM of Ibrutinib was used.

Supplementary Methods

Targeting BTK in CLL patient samples

Patients and samples

Cells were obtained from peripheral blood samples donated by patients fulfilling the standard criteria for CLL after signing informed consent approved by the Tel-Aviv Sourasky Medical's Institutional Review Board according to the Helsinki Accords. Peripheral blood mononuclear cells (PBMC) were isolated by Ficoll density-gradient centrifugation. Viable frozen cells were kept in FCS containing 10% DMSO and stored in liquid nitrogen. Before use, frozen cells were thawed and cultured at 37°C, 5% CO_2, in RPMI medium supplemented with 10% FCS, penicillin-streptomycin, and L-glutamine. The samples used contained more than 90% CLL cells.

Antibodies

ERK1/2, phospho-ERK1/2 (Thr202/Tyr204), Akt (pan), phospho-Akt (S473), PLC γ2, phospho-PLCγ2(Tyr1217) ,BTK, phospho-BTK (Tyr223) were from Cell Signaling Technology (Beverly,MA).Purified anti-human actin antibody was obtained from MP Biomedicals (Illkirch, France). Goat anti Rabbit IgG (H+L)-HRP conjugate and Goat anti Mouse IgG (H+L)-HRP conjugate and Goat F(ab')2 anti-human IgM were from Jackson Immunoresearch Laboratories (West Grove, PA). All antibodies utilized in the study were used in concentrations according to the manufacturer's instructions.

Western blotting

CLL cells ($20X10^6$/mL) were incubated with Ibrutinib or Ibrutinib-based compounds (**3a**, **3c**, **3d, 3e**) at the indicated doses at 37°C. DMSO treated cells served as controls. After 2 hours of incubation, the cells were stimulated with goat F(ab')2 anti-human IgM (10 µg/mL) for 15 minutes or left untreated. CLL cells were lysed in RIPA lysis buffer (Cell Signaling Technology, Beverly, MA) containing phosphatase inhibitor cocktail 2 and protease inhibitor cocktail (Sigma-Aldrich, MO, USA). Extract from cell lysates were separated on 4-15% Criterion™ TGX™ Precast Midi Protein Gel (Bio-Rad Laboratories) and transferred electrophoretically to nitrocellulose membrane (Bio-Rad Laboratories). The membranes were incubated with the designated antibodies and HRP conjugated secondary antibodies according to the manufacturer's instructions. Bands were detected using MyECL Imager (Thermo Scientific, Rockford, IL). A Western blot analysis showed PLCγ2, BTK, Akt, and ERK phosphorylation as well as the total amount of these proteins. Actin was used to verify equal loading.

Effect of 3c on TCL1 transgenic mouse model

µTCL adoptive transfer model

Generation of this mouse model was performed as previously described. [1]

Briefly, TCL-1 mice were sacrificed at approximately 12 months of age, with a malignant cell population higher than 60% in the PB. Their spleens were excised, and 4×10^7 cells resuspended in $PBS^{-/-}$ were injected into the tail vein of 6-weeks-old recipient mice. Progression of the disease was followed in the PB by using flow cytometry for the IgM/CD5 population. Mice with >30% IgM+/CD5+ cells were considered diseased and used for further analysis.

Staining for flow cytometry.

Isolated cells were stained using specific antibodies (IgM-PE, CD5-APC, BioLegend®) in staining buffer (0.5% bovine serum albumin in phosphate-buffered saline) for 30' in 4 ^{o}C in dark then washed twice. Flow cytometry (FACS) analysis was performed using FACS Canto (BD Biosciences) and data were collected using FACSDIva8 (BD Biosciences). FACS data analysis was done using Flowjo v10.

IsoDTB ABPP sample preparation

The probe peptide was synthesized using standard solid phase synthesis on rink amide resin. The resin was swelled in dichloromethane for 30 minutes, washed with DMF, and deprotected using 20% piperidine/DMF (3X5 minutes). 2 equivalents Fmoc-(azidolysine)-OH were coupled in DMF using 2 equivalents of HATU and 4 equivalents of diisopropylethylamine for 2 hours with tumbling, followed by 3 washes with DMF and fmoc deprotection using the same method used above. At this step, 2 equivalents of fmoc-Val-OH (for the light probe) or fmoc-Val-OH(13C5, 99%, 15N, 99%; Cambridge isotope laboratories) were coupled using the same method as before, followed by fmoc deprotection. This was followed by coupling to 2 equivalents of desthiobiotin (using the same method), followed by 3 washes with DMF, 3 washes with dichloromethane, and drying in a vacuum desiccator.

The peptides were cleaved from the resin using 95% TFA, 2.5% TIPS and 2.5% water for 3 hours, followed by thorough evaporation of the cleavage mixture using nitrogen bubbling and

purification by reverse phase HPLC. The purified peptides were dissolved in DMSO to a concentration of 5 mM and used directly.

The preparation of IsoDTB-ABPP samples was performed essentially as described in Zanon et al.[2.] Experiments were conducted in quadruplicates. PATU cells were incubated for 2 h with 5 µM compounds Sulfopin or **3g** (or with DMSO), and collected by centrifuge at 300 g for 5 min followed by ice cold PBS wash. For lysis, samples containing 10 million cells were dispersed in 0.5 mL of RIPA buffer (Sigma, R0278), incubated with occasional vortexing for 30 min on ice, followed by centrifugation at 21,000 g for 15 min. The protein concentration in the samples was determined using BCA assay (Pierce 23227), and each sample was diluted to 1.7 mg/mL using PBS. To each sample, 5 µL of 10 mM IA-alkyne was added, followed by 1 h incubation at room temperature in the dark. 10 µL of 5 mM DesThioTag was added (Light for the compound treated samples, heavy for the DMSO-treated samples), followed by 18 µL of CuSO4:THPTA (100 mM), and the click reaction was initiated by addition of 15 µL of 150 mM sodium ascorbate (freshly dissolved in water). The samples were incubated on a rotary shaker for 1 h at room temperature. The compound-treated and DMSO-treated samples were mixed with 4 mL methanol, 1 mL chloroform and 2 mL water on ice, vortexed and centriduged at 3200 g for 10 min at 4°C. The top layer was aspirated, and 3 mL methanol was added, followed by centrifugation and aspiration of the supernatant. The pellets were air dried and stored at -80°C until the following treatment. The pellets were resuspended in 0.3 mL of 8 M urea freshly dissolved in PBS using probe sonication (8 sec total at 40% amplitude, 1 sec on/2 sec off, at room temperature). Following the resuspension, the samples were diluted with 1 mL of PBS. Then each sample was mixed with 1.3 mL of slurry containing 110 µL of streptavidin agarose beads (Thermo Streptavidin Agarose cat # 20349), prewashed, and dispersed in 0.2% IGEPAL. The samples were incubated with rotation for 3 h at room temperature. The beads were pelleted by centrifugation at 2000 g for 2 min, transferred to spin columns, and washed 3 times with 0.1% IGEPAL/PBS, 3 times PBS, and 3 times water. The beads were then suspended in 8 M Urea/50 mM ammonium bicarbonate, and 15 µL of 31 mg/mL DTT were added, followed by incubation at 37°C for 45 min. The samples were cooled to room temperature, and 15 µL of 74 mg/mL iodoacetamide were added, followed by 30 min incubation at room temperature in the dark, and the addition of a further 15 µL of 31 mg/mL DTT and incubation at room temperature for 30 min. 900 µL of 50 mM ammonium bicarbonate were added, and after 30 min incubation, the beads were pelleted by centrifugation at 2000 g for 2 min, and resuspended in 200 µL of 1 M Urea/50 mM ammonium bicarbonate. At this point, modified trypsin (Promega V511A) was dissolved in trypsin buffer at 0.5 µg/µL, and 4 µL were added to each sample, followed by overnight incubation at 37°C with shaking.

400 µL of 0.1% IGEPAL/PBS were added, and the beads were washed 3 times with 0.1% IGEPAL/PBS, 3 times PBS and 3 times water. The peptides were eluted by incubation with 200 µL of 50% acetonitrile + 0.1% TFA for 5 min, followed by two more portions of 100 µL of 50% acetonitrile + 0.1% TFA. The samples were dried by speedvac, and further purified using Oasis desalting columns (Waters), after which they were dried and run on the instrument.

Pull-down samples preparation

Mino cells (15 million) were incubated for 1 h with either DMSO, Ibrutinib, **3c,** or **3d,** followed by the incubation with 10 µM "probe 4" for another hour. The samples were prepared in quadruplicates. The cells were washed with cold PBS, from the flasks and frozen. Then, each sample was lysed in 200 µL of RIPA for 15 min on ice and centrifuged at 20,000g at 4 °C. The protein in the supernatant was quantified using BCA. For each sample, 250 µL of 1.7 mg/mL was prepared. At this point, 5 µL of 5 mM biotin azide and 9 µL of 100 mM $CuSO_4$/THPTA complex were added. The click reaction was initiated by the addition of 7.5 µL of 150 mM sodium ascorbate, and the samples are incubated at room temperature for 1 h. The samples were then precipitated with methanol/chloroform (1 mL methanol, 250 µL chloroform, 750 µL water), washed with 1 mL of methanol, and air-dried.

The dry pellet was resuspended in 1.2% SDS in Ca/Mg free PBS (250 µL), sonicated (4 × 2 s with 2 s off), and heated to 95 °C for 5 min. The samples were then diluted to 1.5 mL with PBS, and 50 µL of streptavidin agarose beads, prewashed with 0.2% SDS in PBS, was added, followed by 3 h incubation at room temperature. Following the incubation, the beads were centrifuged at 2000g for 2 min and washed four times with the following buffers (4 mL in each wash): 2% SDS; 0.1% sodium deoxycholate, 1% Triton X-100, 0.5 M NaCl, 1 mM EDTA, 50 mM HEPES pH = 7.5; 0.25 M NaCl, 0.5% IGEPAL, 0.5% sodium deoxycholate, 1 mM EDTA, 10 mM Tris pH = 8.1; 50 mM Tris pH = 7.4, 50 mM NaCl.

Following the last wash, the buffer was removed and 100 µL of 7.5% SDS in Tris 50 mM pH = 8 was added, with heating to 95 °C for 6 min and occasional vortexing. Finally, the beads were spun down (2 min 2000g) and 80 µL of supernatant was removed to separate tubes.

Then, 4 µL of 0.1 M DTT was added and samples were incubated at 65 °C for 45 min. After the samples had cooled, 4 µL of iodoacetamide (0.2 M) were added, and the samples were incubated in the dark for 40 min at room temperature. At this point, 1/10 volume of 12% phosphoric acid was added, and the samples were diluted 6-fold with 90% methanol + 50 mM ammonium bicarbonate. The samples were then

loaded on S-trap micro columns (Protify), and the columns were washed three times with 150 µL of 90% methanol + 50 mM ammonium bicarbonate. Then, 20 µL of 0.05 µg/µL of trypsin in 50 mM ammonium bicarbonate was added to the columns and the samples were incubated at 47 °C for 90 min. Then, 40 µL of 50 mM ammonium bicarbonate was added, followed by centrifugation and addition of 1 µL of 0.5 µg/µL trypsin to the eluate, which was incubated at 37 °C overnight. The column itself was then eluted using 40 µL of 0.2% formic acid and 40 µL 0.2% formic acid in 50% acetonitrile into a separate tube, which was kept at 4 °C. The two eluates were then combined and evaporated. The samples were further desalted using Oasis desalting columns (Waters) and then evaporated again and dissolved in 30 µL of 3% acetonitrile with 0.1% formic acid.

Running Proteomics samples

Samples were analyzed using EASY-nLC 1200 nano-flow UPLC system, using PepMap RSLC C18 column (2 µm particle size, 100 Å pore size, 75 µm diameter × 50 cm length), mounted using an EASY-Spray source onto an Exploris 240 mass spectrometer. uLC/MS-grade solvents were used for all chromatographic steps at 300 nL/min. The mobile phase was: (A) H2O + 0.1% formic acid and (B) 80% acetonitrile + 0.1% formic acid. Each sample (2 µL) was injected. Peptides were eluted from the column into the mass spectrometer using the following gradient: 1–40% B in 160 min, 40–100% B in 5 min, maintained at 100% for 20 min, 100 to 1% in 10 min, and finally 1% for 5 min. Ionization was achieved using a 1900 V spray voltage with an ion transfer tube temperature of 275 °C. Data were acquired in data-dependent acquisition (DDA) mode. MS1 resolution was set to 120,000 (at 200 m/z), a mass range of 375–1650 m/z, normalized AGC of 300%, and the maximum injection time was set to 20 ms. MS2 resolution was set to 15,000, quadrupole isolation 1.4 m/z, normalized AGC of 50%, dynamic exclusion of 45 s, and automatic maximum injection time.

Proteomics Data Analysis

Data analysis for pull down samples was performed using MaxQuant 1.6.3.4. Human proteome (updated November 2020) was downloaded from Uniprot. Carbamidomethyl was used as a fixed modification and methionine oxidation and N terminal acetylation as variable modification. The digestion enzyme was set to Trypsin/P with a maximum number of missed cleavages of 2. The “Re-quantify” option was enabled. Contaminants were included. Peptides were searched with a minimum peptide length of 7 and a maximum peptide mass of 4,600 Da. “Second peptides” was enabled and “Dependent peptides” were disabled. The option “Match between run” was enabled with a Match time window of 0.7 min and an

alignment window of 20 min. An FDR of 0.01 was used for Protein FDR, PSM FDR and XPSM FDR. Label free quantification was used to quantify the proteins, with each data set (DMSO-treated samples, compound-treated samples, etc.) analyzed with a separate parameter group. The results were analyzed with Perseus. Contaminant proteins, proteins only identified by modified peptides, and proteins identified from reverse peptides were removed. LFQ intensities were transformed into Log2 values and only proteins in which at least one data set contained 3 non zero intensities (out of 4) were retained. Non-valid values were replaced by values from a normal distribution with a width of 0.3 sigma and a down shift of 1.8 sigma. The data was finally analyzed using students T-test.

Data analysis for IsoDTB ABPP data was performed similarly to Zanon et al.[2] using MaxQuant 1.6.3.4. Human proteome (updated November 2020) was downloaded from Uniprot. For each protein not containing selenocysteine, a copy of the protein sequence containing a single mutation of cysteine to selenocysteine (C→U) was created for each cysteine in the sequence to an unmutated copy. The IsoTOPP labeles were then defined as Heavy/Light labels with the following formulae: C(24)H(49)N(8)Cx(5)Nx(1)S(1)Se(-1) for the heavy label, and C(29)H(49)N(9)S(1)Se(-1) for the light label. In addition we added diagnostic peaks corresponding to the free amine generated by cleavage of the iodoacetamide alkyne (C(22)H(49)N(8)O(4)Cx(5)Nx(1)/C(27)H(49)N(9)O(4)), internal cleavage caused by attack of the triazole on the alpha carbon of the iodoacetamide moiety[2] (C(22)H(46)N(7)O(4)Cx(5)Nx(1)/ C(27)H(46)N(8)O(4)), cleavage of the peptide bond between azidolsyine and valine (C(10)H(25)N(2)O(3)Cx(5)Nx(1)/C(15)H(25)N(3)O(3)), and cleavage of the peptide bond between valine and desthiobiotin (C(10)H(16)N(2)O(2)). A multiplicity of 2 was set, and maximum number of labeled amino acids of 1. The digestion enzyme was set to Trypsin/P with a maximum number of missed cleavages of 2. No variable modifications were included. The “Re-quantify” option was enabled. Carbamidomethyl (C2H3NO) was used as a fixed modification on cysteine. Contaminants were included. Peptides were searched with a minimum peptide length of 7 and a maximum peptide mass of 4,600 Da. “Second peptides” and “Dependent peptides” were disabled and the option “Match between run” was enabled with a Match time window of 0.7 min and an alignment window of 20 min. An FDR of 0.01 was used for Protein FDR, PSM FDR and XPSM FDR. After MaxQuant analysis, the data for each compound was analyzed separately. Following data analysis, reverse and contaminant peptides were removed. Only peptides for which non-zero total intensities were measured for at least three of the replicates were analyzed. Average H/L ratios were calculated as the ratio of the sum of heavy intensities in the replicates to the sum of the light

intensities. Ratios that were above 20 or infinite (due to the sum being zero for the low intensities) were defined as 20. Intensities measured for different peptides containing the same modified cysteine were combined.

Determination of K_I and K_{inact} for Sulfopin and 4d

Pin 1 (500 nM) was incubated at various concentrations of Sulfopin (0.5, 1, 2, 5, 20, 50, 100, 150, 200 µM) or **4d** (0.5, 1, 2, 5, 10, 20, 50, 100 µM) by adding 1/100th volume from the stock solution in 100 mM Tris buffer at pH 7.4. After the addition, the samples are put at 14 °C. The reaction mixtures, at various time points (indicated in the graph), were injected into the LC/MS. For data analysis, the raw spectra were deconvoluted using a 10000:30000 Da window and 1 Da resolution. The labeling percentage for a compound was determined as the labeling of a specific compound (alone or together with other compounds) divided by the overall detected protein species. The % of unlabelled Pin 1 was plotted against time and fitted to the pseudo-first-order reaction kinetics formula (Figure S24A & C). The obtained rate constants were fitted to the Michaelis-Menten-type saturation curve (Fig. S24B & D) to extrapolate k_{inact} and K_I.

References

(1) Hofbauer, J. P.; Heyder, C.; Denk, U.; Kocher, T.; Holler, C.; Trapin, D.; Asslaber, D.; Tinhofer, I.; Greil, R.; Egle, A. Development of CLL in the TCL1 Transgenic Mouse Model Is Associated with Severe Skewing of the T-Cell Compartment Homologous to Human CLL. *Leukemia* **2011**, *25* (9), 1452–1458.

(2) Zanon, P. R. A.; Lewald, L.; Hacker, S. M. Isotopically Labeled Desthiobiotin Azide (isoDTB) Tags Enable Global Profiling of the Bacterial Cysteinome. *Angew. Chem. Int. Ed Engl.* **2020**, *59* (7). https://doi.org/10.1002/anie.201912075.

Supporting Chemistry

1. **Materials and methods**: All reagents and solvents were obtained from commercial suppliers unless otherwise mentioned. Ibr-H (CAS 1022150-12-4) was purchased from BLD pharmatech. Deuterated solvents were purchased from Cambridge isotope laboratories and all other reagents are purchased from Sigma Aldrich and used as such without further purification. Aluminum-backed silica plates (Merck silica gel 60 F254) were used for thin layer chromatography (TLC) to monitor solution phase reactions. The purification of compounds was carried out on a combi flash chromatography and waters RP-HPLC with Prep C18 column. All the compounds used in the reactivity assays/cellular assays were waters RP-HPLC with Prep C18 column. The ^{1}H-NMR and ^{13}CNMR spectra were recorded using a 400 MHz and 500 MHz Bruker advance spectrometers and were calibrated using residual undeuterated solvent as the internal references ($CDCl_3$: 7.26:; DMSO-d6: 2.50:; D_2O: 4.79: and CD_3OD = 3.31:). Chemical shifts are reported in: on a δ scale. The following abbreviations were used to explain NMR peak multiplicities: s = singlet, d = doublet, t = triplet, q = quartet, p = pentet, m = multiplet, br = broad. Most of the Ibrutinib derivatives appeared as a mixture of rotamers. The high-resolution mass spectra were recorded on Waters Xevo G2-XS QTof mass spectrometer using electrospray ionization time-of-flight (ESI-TOF) reflectron experiments.

2. **Abbreviations.** Acetonitrile (ACN), dichloromethane (DCM), N,N'-diisopropylethylamine (DIPEA), N,N'-dimethylformamide (DMF), Ethyl acetate (EtOAc), dimethyl sulfoxide (DMSO), 1-Ethyl-3-(3-dimethylaminopropyl) carbodiimide (EDC), 1-[bis(dimethylamino)methylene]-1H-1,2,3- triazolo[4,5-b]pyridinium3-oxid hexafluorophosphate (HATU), methanol (MeOH), Phosphate buffer saline (PBS), high-performance liquid chromatography (HPLC), trifluoroacetic acid (TFA), 1,4-diazabicyclo[2.2.2]octane (DABCO), diisopropylethyl amine (DIPEA)

N-benzyl-2-chloroacetamide (1a):

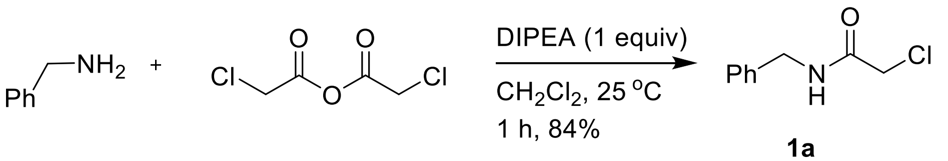

1a

To a stirred solution of Bn-NH_2 (108 µL, 1 mmol) in anhydrous DCM (2 mL), DIPEA (178 µL, 1 mmol) and chloroacetic anhydride (170 mg, 1 mmol) were added at 25 °C. The reaction mixture was stirred at room temperature for 1 h. After completion of the reaction (as monitored by LC-MS), CH_2Cl_2 was concentrated *in vacuo* and the crude product was purified using combi flash column chromatography using EtOAc:Hexane as eluent to give **1a** in 154 mg (yield = 84%)

^{1}H NMR (500 MHz, $CDCl_3$): δ 4.12 (s, 2H), 4.51 (d, J = 5.8 Hz, 2H), 6.88 (br. s., 1H), 7.28-7.34 (m, 3H), 7.34-7.42 (m, 2H): **^{13}C NMR** (126 MHz, $CDCl_3$): δ 42.6, 43.9, 127.8, 127.8, 128.8, 137.2, 165.8. : **ESI-MS** (m/z): calculated for $C_9H_{11}ClNO$ [M+H]+: 184.05; found: [M+H]+:184.82.

N-benzyl-2-hydroxyacetamide (Pre-1):

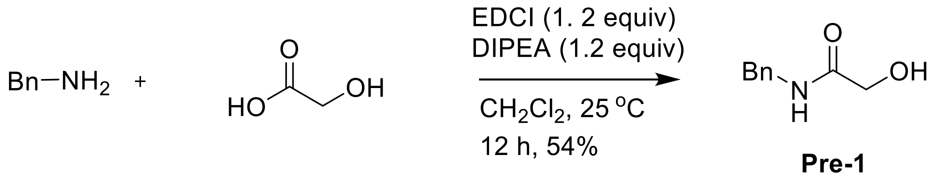

Pre-1

To a stirred solution of Bn-NH_2 (324 µL, 3 mmol) in anhydrous DCM (6 mL), 1-Ethyl-3-(3-dimethylaminopropyl)carbodiimide hydrochloride (690 mg, 3.6 mmol) and DIPEA (600 µL, 3.6 mmol) were added at 25 °C. The reaction mixture was stirred at room temperature for 12 h. After completion of the reaction (as monitored by LC-MS), the reaction mixture was evaporated under *vacuo* and the crude product was purified using combi flash column chromatography with MeOH:EtOAc (2:8) as eluent to give **Pre-1** was colorless solid in 267 mg (yield = 54 %).

ESI-MS (m/z): calculated for $C_9H_{12}NO_2$ [M+H]+: 166.08; found: [M+H]+:166.30.

2-(benzylamino)-2-oxoethyl methanesulfonate (1b):

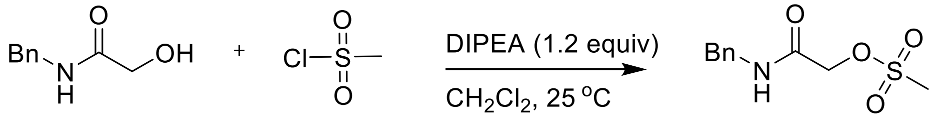

Pre-1 1b

To a stirred solution of **Pre-1** (16.5 mg, 0.1 mmol) in CH_2Cl_2 (1 mL), methane sulfonyl chloride (9.2 μL, 0.12 mmol, d = 1.48), and DIPEA (20.4 μL, 0.12 mmol) were added at 25 °C. The reaction mixture was stirred at room temperature for 1 h. After completion of the reaction (as monitored by LC-MS), water (1 mL) was added. The aqueous layer was extracted with CH_2Cl_2 (3 × 1 mL). The combined organic layer was concentrated *in vacuo* and the crude product was purified by preparative HPLC using water:ACN (0.1% formic acid) solvent gradient to afford white solid **1b** in 13.8 mg (57% yield).

^{1}H NMR (400 MHz, $CDCl_3$) δ: 2.50 (br. s., 1H), 3.07 (s, 3H), 4.47 (d, J = 5.7 Hz, 2H), 4.66 (s, 2H), 6.67 (br. s., 1H), 7.19-7.40 (m, 4H): **^{13}C NMR** (101 MHz, $CDCl_3$): δ37.8, 43.4, 66.6, 127.8, 127.8, 128.8, 137.0, 165.2. **ESI-MS** (m/z): calculated for $C_{10}H_{14}NO_4S$ [M+H]+: 244.06; found: [M+H]+:244.64.

2-(benzylamino)-2-oxoethyl 4-methylbenzenesulfonate (1c)

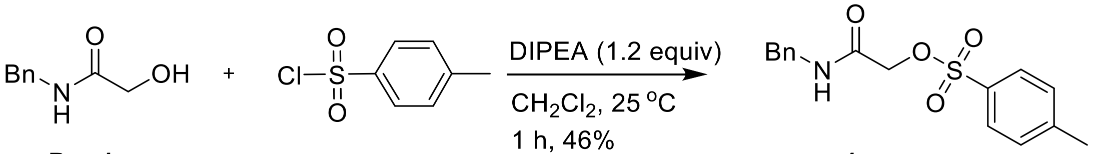

Pre-1 **1c**

To a stirred solution of **Pre-1** (16.5 mg, 0.1 mmol) in CH_2Cl_2 (1 mL), 4-toluenesulfonyl chloride (22.8 mg, 0.12 mmol), and DIPEA (20.4 μL, 0.12 mmol) were added at 25 °C. The reaction mixture was stirred at room temperature for 1 h. After completion of the reaction (as monitored by LC-MS), water (1 mL) was added. The aqueous layer was extracted with CH_2Cl_2 (3 × 1 mL). The combined organic layer was concentrated *in vacuo* and the crude product was purified by preparative HPLC using water:ACN (0.1% formic acid) solvent gradient to afford white solid **1c** in 14.6 mg (46% yield).

^{1}H NMR (500 MHz, $CDCl_3$) δ: 2.47 (s, 3H), 4.46 (d, J = 5.9 Hz, 2H), 4.49 (s, 2H), 6.66 (br. s., 1H), 7.25 (d, J = 7.0 Hz, 2H), 7.28-7.33 (m, 1H), 7.37 (d, J = 8.1 Hz, 2H), 7.34 (d, J = 7.6 Hz, 2H), 7.79 (d, J = 8.3 Hz, 2H): **^{13}C NMR** (126 MHz, $CDCl_3$) δ: 21.7, 43.3, 66.9, 127.8, 128.1, 128.8, 130.2, 131.6, 137.0, 145.9, 165.2. **ESI-MS** (m/z): calculated for $C_{16}H_{18}NO_4S$ [M+H]+: 320.10; found: [M+H]+:320.55.

2-(benzylamino)-2-oxoethyl methylsulfamate (1d)

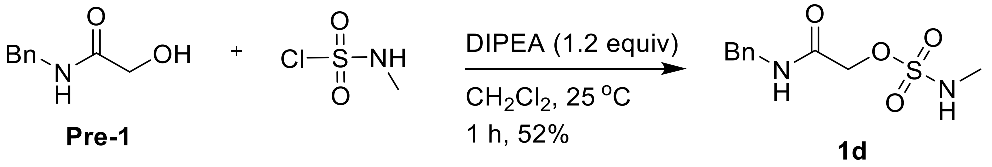

To a stirred solution of **Pre-1** (16.5 mg, 0.1 mmol) in CH_2Cl_2 (1 mL), N-methylsulfamoyl chloride (15.4 mg, 0.12 mmol), and DIPEA (20.4 µL, 0.12 mmol) were added at 25 °C. The reaction mixture was stirred at room temperature for 1 h. After completion of the reaction (as monitored by LC-MS), water (1 mL) was added. The aqueous layer was extracted with CH_2Cl_2 (3 × 1 mL). The combined organic layer was concentrated *in vacuo* and the crude product was purified by preparative HPLC using water:ACN (0.1% formic acid) solvent gradient to afford white solid **1d** in 13.4 mg (52% yield). **^{1}H NMR** (400 MHz, $CDCl_3$) δ: 2.79 (d, J = 5.1 Hz, 3H), 4.50 (d, J = 5.7 Hz, 2H), 4.59 (s, 2H), 5.24 (d, J = 4.6 Hz, 1H), 6.79 (br. s., 1H), 7.18-7.45 (m, 4H):**^{13}C NMR** (100 MHz, $CDCl_3$) δ: 29.8, 43.3, 67.2, 127.8, 128.8, 137.0, 166.0. **ESI-MS** (m/z): calculated for $C_{10}H_{15}N_2O_4S$ [M+H]+: 259.08; found: [M+H]+:259.64.

2-(benzylamino)-2-oxoethyl benzylsulfamate (1e)

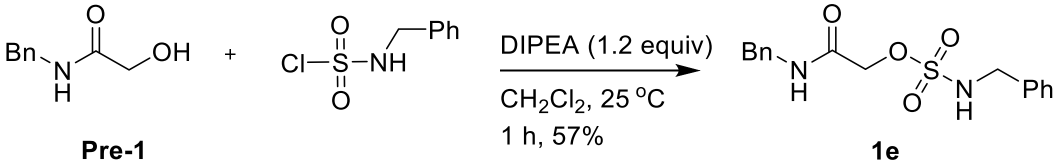

To a stirred solution of **Pre-1** (16.5 mg, 0.1 mmol) in CH_2Cl_2 (1 mL), benzylsulfamoyl chloride (24 mg, 0.12 mmol), and DIPEA (20.4 µL, 0.12 mmol) were added at 25 °C. The reaction mixture was stirred at room temperature for 1 h. After completion of the reaction (as monitored by LC-MS), water (1 mL) was added. The aqueous layer was extracted with CH_2Cl_2 (3 × 1 mL). The combined organic layer was concentrated *in vacuo* and the crude product was purified by preparative HPLC using water:ACN (0.1% formic acid) solvent gradient to afford white solid **1e** in 19 mg (57% yield).
^{1}H NMR (500 MHz, $CDCl_3$) δ: 4.31 (d, J = 5.6 Hz, 2H), 4.45 (s, 2H), 4.49 (s, 2H), 5.40 (t, J = 5.4 Hz, 1H), 6.48 (br. s., 1H), 7.24-7.29 (m, 3H), 7.29-7.35 (m, 6H), 7.35-7.40 (m, 2H): **^{13}C NMR** (126 MHz, $CDCl_3$) δ: 43.3, 48.0, 67.1, 127.8, 127.8, 128.1, 128.5, 128.8, 129.0, 135.7, 137.0, 165.6. **ESI-MS** (m/z): calculated for $C_{16}H_{19}N_2O_4S$ [M+H]+: 335.11; found: [M+H]+:335.18.

2-(benzylamino)-2-oxoethyl phenylsulfamate (1f)

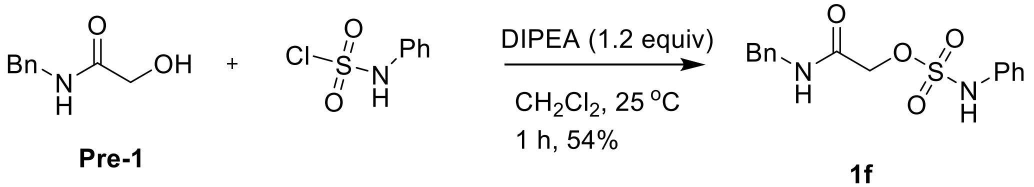

Pre-1 **1f**

To a stirred solution of **Pre-1** (16.5 mg, 0.1 mmol) in CH_2Cl_2 (1 mL), phenylsulfamoyl chloride (22.5 mg, 0.12 mmol), and DIPEA (20.4 µL, 0.12 mmol) were added at 25 °C. The reaction mixture was stirred at room temperature for 1 h. After completion of the reaction (as monitored by LC-MS), water (1 mL) was added. The aqueous layer was extracted with CH_2Cl_2 (3 × 1 mL). The combined organic layer was concentrated *in vacuo* and the crude product was purified by preparative HPLC using water:ACN (0.1% formic acid) solvent gradient to afford white solid **1f** in 17.2 mg (54% yield).
^{1}H NMR (400 MHz, $CDCl_3$) δ: 4.42 (d, J = 5.7 Hz, 2H), 4.66 (s, 2H), 6.55 (br. s., 1H), 7.21 (d, J = 7.5 Hz, 4H), 7.33 (d, J = 7.7 Hz, 4H), 7.49 (s, 1H): **^{13}C NMR** (126 MHz, $CDCl_3$) δ: 29.3, 42.7, 66.6, 119.7, 121.0, 124.7, 126.8, 127.3, 128.3, 129.1, 135.9, 162.4 (s) **ESI-MS** (m/z): calculated for $C_{15}H_{17}N_2O_4S$ [M+H]+: 321.09; found: [M+H]+:321.57.

2-(benzylamino)-2-oxoethyl (4-bromophenyl)sulfamate (1g):

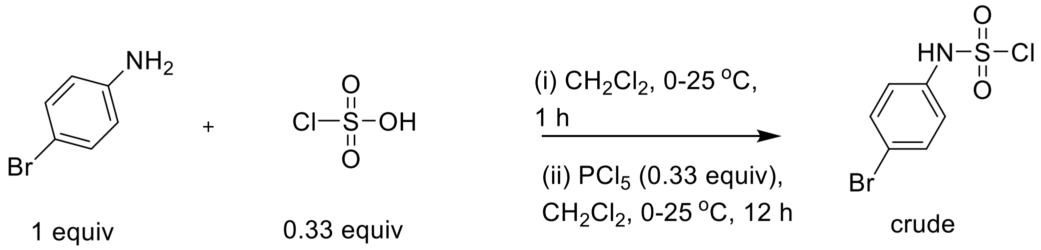

1 equiv 0.33 equiv crude

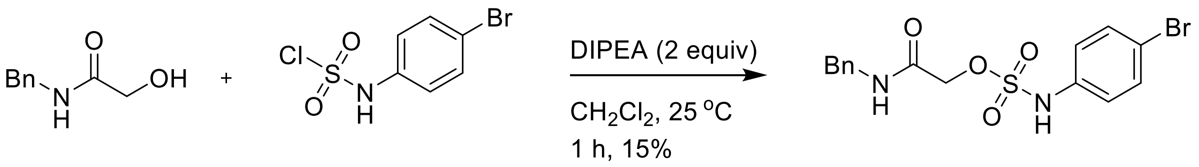

1 equiv 2 equiv **1g**

To a stirred solution of 4-bromo aniline (172 mg, 1 mmol) in CH_2Cl_2 (1 mL), chloro methane sulfonyl chloride (22 µL, 0.33 mmol,) was added at 0 °C. The reaction mixture was stirred at room temperature

for 1 h. After completion of the reaction organic layer was concentrated *in vacuo*. The crude product was dissolved CH_2Cl_2 (1 mL) and PCl_5 (68 mg, 0.33 mmol) was added at 0 °C. The reaction mixture was stirred at room temperature for 12 h. After completion of the reaction (as monitored by LC-MS), the reaction mixture is filtered and washed with dichloromethane. The filtrate was concentrated and used as such for the next reaction.

To a stirred solution of **Pre-1** (16.5 mg, 0.1 mmol) in CH_2Cl_2 (1 mL), 4-bromo phenylsulfamoyl chloride (0.2 mmol), and DIPEA (35 µL, 0.2 mmol) were added at 25 °C. The reaction mixture was stirred at room temperature for 1 h. After completion of the reaction (as monitored by LC-MS), water (1 mL) was added. The aqueous layer was extracted with CH_2Cl_2 (3 × 2 mL). The combined organic layer was concentrated *in vacuo* and the crude product was purified by preparative HPLC using water:ACN (0.1% formic acid) solvent gradient to afford white solid **1g** in 6 mg (15% yield).

^{1}H NMR (500 MHz, $CDCl_3$) δ: 4.45 (d, J = 5.8 Hz, 2H), 4.66 (s, 2H), 6.36-6.49 (m, 1H), 7.04-7.11 (m, 2H), 7.17 (s, 1H), 7.23 (d, J = 6.9 Hz, 2H), 7.32-7.39 (m, 3H), 7.43-7.50 (m, 2H): ^{13}C NMR (126 MHz, $CDCl_3$) δ: 43.7, 68.3, 114.6, 122.8, 128.1, 128.2, 129.2, 133.1, 137.6, 165.8, 173.3. **ESI-MS** (m/z): calculated for $C_{15}H_{16}Br^{81}N_2O_4S$ [M+H]+:401.00; found: [M+H]+:401.22. $C_{15}H_{16}Br^{79}N_2O_4S$ [M+H]+: 399.00; found: [M+H]+:399.15.

2-(benzylamino)-2-oxoethyl dimethylsulfamate (1h)

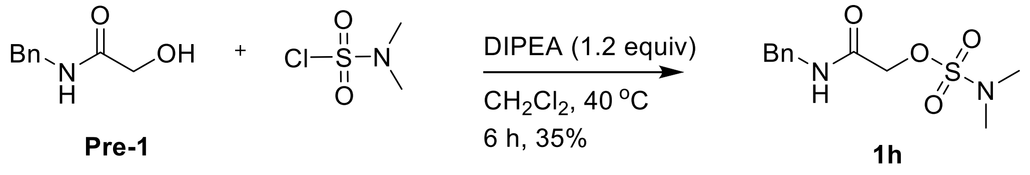

To a stirred solution of **Pre-1** (16.5 mg, 0.1 mmol) in CH_2Cl_2 (1 mL), N,N-dimethylsulfamoyl chloride (16.9 mg, 0.12 mmol), and DIPEA (20.4 µL, 0.12 mmol) were added at 40 °C. The reaction mixture was stirred at room temperature for 6 h. After completion of the reaction (as monitored by LC-MS), water (1 mL) was added. The aqueous layer was extracted with CH_2Cl_2 (3 × 1 mL). The combined organic layer was concentrated *in vacuo* and the crude product was purified by preparative HPLC using water:ACN (0.1% formic acid) solvent gradient to afford white solid **1h** in 9.5 mg (35% yield).

^{1}**H NMR** (500 MHz, CD_3OD) δ: 2.92 (s, 6H), 4.46 (s, 2H), 4.63 (s, 2H), 7.23-7.31 (m, 1H), 7.33 (s, 4H): ^{13}**C NMR** (125 MHz, CD_3OD) δ: 38.8, 44.0, 48.6, 68.3, 128.5, 128.8, 129.7, 139.7, and 168.6. **ESI-MS** (m/z): calculated for $C_{11}H_{17}N_2O_4S$ [M+H]+: 273.09; found: [M+H]+:273.86.

N-benzyl-2-(methylsulfonyl)acetamide (1i):

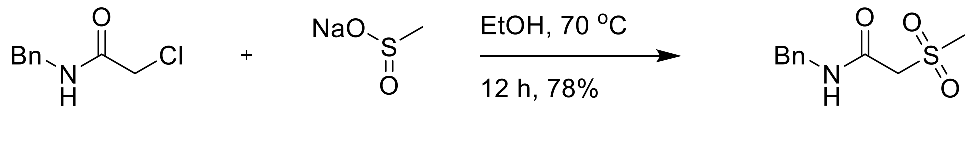

To a stirred solution of **1a** (18.3 mg, 0.1 mmol) in ethanol (1 mL), sodium methanesulfinate (20.4 mg, 0.2 mmol) was added at 25 °C. The reaction mixture was stirred at room temperature for 12 h at 70 °C. After completion of the reaction (as monitored by LC-MS), ethanol was concentrated *in vacuo* and the crude product was purified by preparative HPLC using water:ACN (0.1% formic acid) solvent gradient to afford white solid **1i** in 17.7 mg (78% yield).

ESI-MS (m/z): calculated for $C_{10}H_{14}NO_3S$ [M+H]+: 228.07; found: [M+H]+:228.22.

N-benzyl-2-(phenylsulfonyl)acetamide (3g):

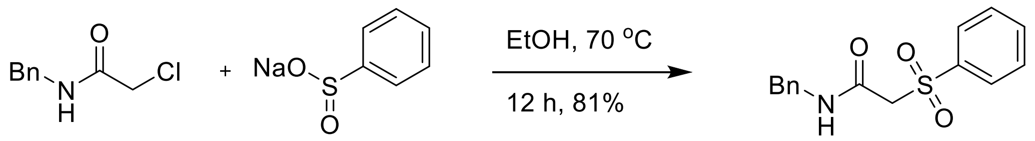

To a stirred solution of **1a** (23 mg, 0.1 mmol) in ethanol (1 mL), sodium phenylsulfinate (20.2 mg, 0.2 mmol) was added at 25 °C. The reaction mixture was stirred at room temperature for 12 h at 70 °C. After completion of the reaction (as monitored by LC-MS), ethanol was concentrated *in vacuo* and the crude product was purified by preparative HPLC using water:ACN (0.1% formic acid) solvent gradient to afford white solid **1j** in 23.4 mg (81% yield).

ESI-MS (m/z): calculated for $C_{15}H_{16}NO_3S$ [M+H]+: 290.09; found: [M+H]+:290.65.

N-benzyl-2-hydroxyacetamide (Pre-2):

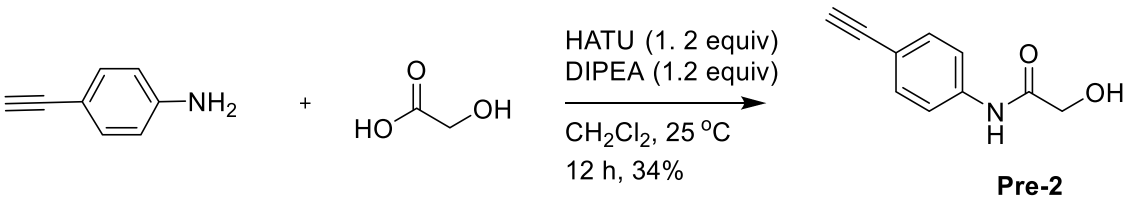

Pre-2

To a stirred solution of 4-ethynylaniline (117 mg, 1 mmol) in anhydrous DCM (6 mL), HATU (456 mg, 1.2 mmol) and DIPEA (200 µL, 1.2 mmol) were added at 25 °C. The reaction mixture was stirred at room temperature for 12 h. After completion of the reaction (as monitored by LC-MS), the reaction mixture was evaporated under *vacuo* and the crude product was purified using combi flash column chromatography with MeOH:EtOAc (2:8) as eluent to give **Pre-2** was colorless solid in 59 mg (yield = 34 %).

^{1}H NMR (500 MHz, $CDCl_3$) δ: 3.00 (s, 1H), 3.24 (br. s., 1H), 4.02 (s, 3H), 7.35 (d, J = 8.0 Hz, 2H), 7.46 (d, J = 8.1 Hz, 2H): **^{13}C NMR** (126 MHz, $CDCl_3$) δ: 65.8, 80.8, 87.2, 121.9, 123.4, 136.8, 141.5, 175.3 (s) : **ESI-MS** (m/z): calculated for $C_{10}H_9NO_2S$ [M+H]+: 176.07; found: [M+H]+:176.09.

2-((4-ethynylphenyl)amino)-2-oxoethyl benzylsulfamate (2a)

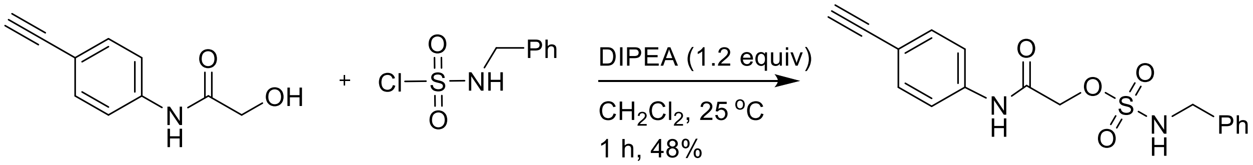

2a

To a stirred solution of **Pre-2** (17.5 mg, 0.1 mmol) in CH_2Cl_2 (1 mL), benzylsulfamoyl chloride (24 mg, 0.12 mmol), and DIPEA (20.4 µL, 0.12 mmol) were added at 25 °C. The reaction mixture was stirred at room temperature for 1 h. After completion of the reaction (as monitored by LC-MS), water (1 mL) was added. The aqueous layer was extracted with CH_2Cl_2 (3 × 1 mL). The combined organic layer was concentrated *in vacuo* and the crude product was purified by preparative HPLC using water:ACN (0.1% formic acid) solvent gradient to afford white solid **2a** in 16.5 mg (48% yield).

^{1}H NMR (400 MHz, $CDCl_3$) δ: 3.08 (s, 1H), 4.38 (d, J = 5.7 Hz, 2H), 4.54-4.57 (m, 2H), 5.17 (t, J = 5.5 Hz, 1H), 7.33-7.41 (m, 4H), 7.42-7.53 (m, 4H), 7.80 (br. s., 1H). **^{13}C NMR** (100 MHz, $CDCl_3$) δ: 48.4, 67.6, 83.4, 120.2, 128.4, 129.0, 129.4, 133.3, 135.7, 137.1, 163.7. **ESI-MS** (m/z): calculated for $C_{17}H_{17}N_2O_4S$ [M+H]+: 345.09; found: [M+H]+:345.67.

2-((4-ethynylphenyl)amino)-2-oxoethyl phenylsulfamate (2b)

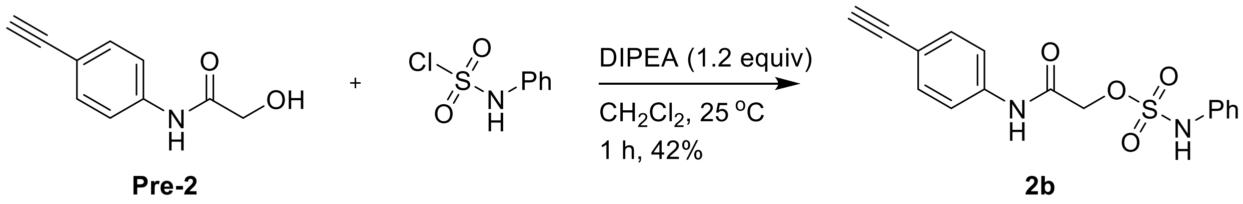

To a stirred solution of **Pre-2** (17.5 mg, 0.1 mmol) in CH_2Cl_2 (1 mL), phenylsulfamoyl chloride (22.5 mg, 0.12 mmol), and DIPEA (20.4 µL, 0.12 mmol) were added at 25 °C. The reaction mixture was stirred at room temperature for 1 h. After completion of the reaction (as monitored by LC-MS), water (1 mL) was added. The aqueous layer was extracted with CH_2Cl_2 (3 × 1 mL). The combined organic layer was concentrated *in vacuo* and the crude product was purified by preparative HPLC using water:ACN (0.1% formic acid) solvent gradient to afford white solid **2b** in 13.8 mg (42% yield).
^{1}H NMR (500 MHz, $CDCl_3$) δ: 3.07 (s, 1H), 4.74 (s, 2H), 7.26 (br. s., 2H), 7.28 (br. s., 2H), 7.37-7.42 (m, 3H), 7.44-7.47 (m, 2H): **^{13}C NMR** (126 MHz, $CDCl_3$) δ: 67.9, 68.6, 83.0, 119.8, 121.2, 126.5, 129.9, 133.0, 137.3, 141.0, 163.2: ESI-MS (m/z): calculated for $C_{16}H_{15}N_2O_4S$ [M+H]+: 331.08; found: [M+H]+:331.97

2-chloro-N-(4-ethynylphenyl)acetamide (2a):

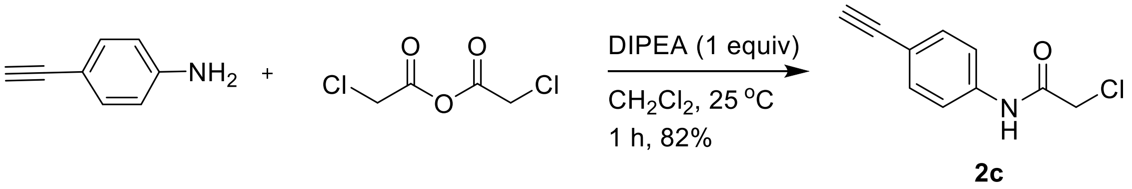

To a stirred solution of 4-ethynylaniline (11.7 mg, 1 mmol) in anhydrous DCM (0.5 mL), DIPEA (17.8 µL, 1 mmol) and chloroacetic anhydride (17 mg, 1 mmol) were added at 25 °C. The reaction mixture was stirred at room temperature for 1 h. After completion of the reaction (as monitored by LC-MS), CH_2Cl_2 was concentrated *in vacuo* and the crude compound was purified by preparative HPLC using water:ACN (0.1% formic acid) solvent gradient to afford white solid **2c** in 15.8 mg (yield = 82%).

^{1}H NMR (400 MHz, $CDCl_3$) δ: 3.08 (s, 1H), 4.20 (s, 2H), 7.41-7.60 (m, 4H), 8.28 (br. s., 1H): **^{13}C NMR** (100 MHz, $CDCl_3$) δ: 42.8, 83.0, 118.8, 119.6, 133.0, 137.0, 163.8. : ESI-MS (m/z): calculated for $C_{10}H_9ClNO$ [M+H]+: 194.04; found: [M+H]+:194.82.

(R)-3-(4-phenoxyphenyl)-1-(piperidin-3-yl)-1H-pyrazolo[3,4-d]pyrimidin-4-amine (Ibrutinib):

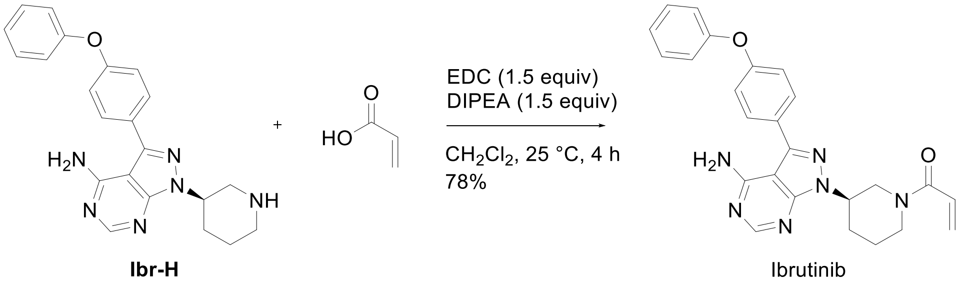

To a stirred solution of acrylic acid (1.02 mL, 15 mmol) in anhydrous CH_2Cl_2 (50 mL), N-(3-dimethylaminopropyl)-N′-ethylcarbodiimide hydrochloride (EDC.HCl) (2.88 g, 15 mmol), N,N-Diisopropylethylamine (2.60 mL, 15 mmol) and Ibr-H (3.87 g, 10 mmol) were added at 0 °C under N_2 atmosphere. The reaction mixture was stirred at room temperature for 4 h. After completion (as monitored by LC-MS), of the reaction, H_2O (30 mL) was added. The organic layer was extracted with CH_2Cl_2 (3 x 50 mL) and evaporated under *vacuo.* The crude product was purified by column chromatography over silica gel using EtOAc:MeOH (9:1)/Pet. ether as eluent to give pure Ibrutinib as colorless solid 3.47 g (yield = 78%). This compound is reported in the literature.[3]

^{1}H NMR (500 MHz, CD_3OD) (as a mixture of rotamers) δ: 1.67-1.78 (m, 1H), 2.04-2.15 (m, 1H), 2.26 (dd, *J* = 12.7, 3.6 Hz, 1H), 2.33-2.44 (m, 1H), 3.26 (t, J = 10.4, 0.4H) (t, *J* = 11.3 Hz, 0.6H), 3.58 (dd, *J* = 12.2, 10.2 Hz, 0.6H), 3.88 (m, 0.4H), 4.05 (d, *J* = 13.6 Hz, 0.6H), 4.23 (m, 0.8H), 4.56 (d, *J* = 12.0 Hz, 0.6H), 4.88 (m, 1H), 5.76 (d, *J* = 10.7 Hz, 1H), 6.11-6.23 (m, 1H), 6.81 (dd, *J* = 16.7, 10.7 Hz, 1H), 7.10 (d, *J* = 7.7 Hz, 2H), 7.15 (d, *J* = 8.7 Hz, 2H), 7.18 (t, *J* = 7.4 Hz, 1H), 7.41 (t, *J* = 8.0 Hz, 2H), 7.68 (d, *J* = 8.7 Hz, 2H), 8.37-8.46 (m, 1H); **^{13}C NMR** (126 MHz, CD_3OD) (as a mixture of rotamers) δ: 24.2, 25.7, 30.5, 30.7, 43.5, 47.1, 47.1, 50.9, 54.4, 55.0, 98.1, 120.0, 120.7, 125.2, 127.1, 128.9, 131.1, 131.3, 147.9, 148.3, 153.3, 154.5, 157.7, 160.5, 167.9. **HR-MS** (m/z): Calculated for $C_{25}H_{24}N_6O_2$ $[M+H]^+$: 441.2039; Found: $[M+H]^+$: 441.2030

(R)-1-(3-(4-amino-3-(4-phenoxyphenyl)-1H-pyrazolo[3,4-d]pyrimidin-1-yl)piperidin-1-yl)-2-chloroethan-1-one (3a)

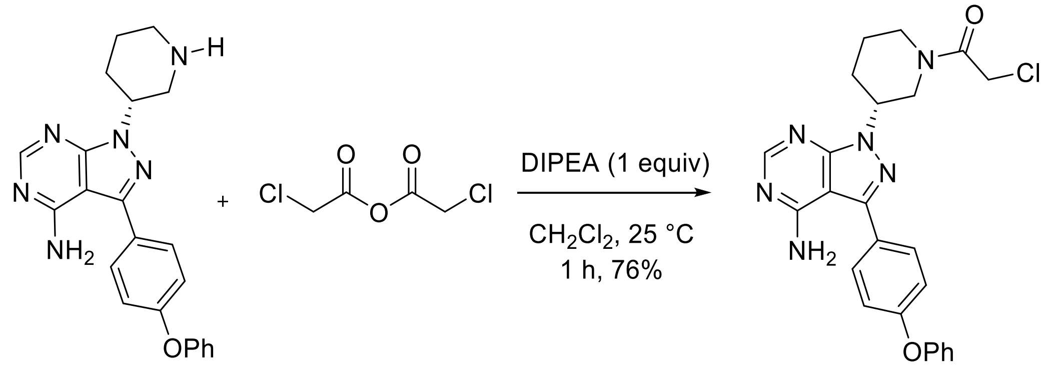

Ibr-H **3a**

To a stirred solution of Ibr-H (387 mg, 1 mmol) in anhydrous DCM (6 mL), DIPEA (178 µL, 1 mmol) and chloroacetic anhydride (170 mg, 1 mmol) were added at 25 °C. The reaction mixture was stirred at room temperature for 1 h. After completion of the reaction (as monitored by LC-MS), CH_2Cl_2 was concentrated *in vacuo* and the crude carboxylic acid was purified using combi flash column chromatography using MeOH:EtOAc (2:8) as eluent to give **3a** in 353 mg (yield = 76%).

^{1}H NMR (500 MHz, CD3OD) (as a mixture of rotamers) δ□ 1.68-1.74 (m, 0.5H), 1.79-1.89 (m, 0.5H), 1.92-2.03 (m, 0.5H), 2.04-2.14 (m, 0.5H), 2.16-2.27 (m, 1H), 2.28-2.43 (m, 1H), 3.11-3.16 (m, 0.5H), 3.46-3.50 (m, 0.5H), 3.84-3.99 (m, 1H), 4.07-4.16 (m, 0.5H), 4.21-4.40 (m, 2H), 4.55 (d, J = 16.4 Hz, 0.5H), 4.78-4.84 (m, 0.5H), 4.94-4.98 (m, 1H), 7.10 (d, J = 8.0 Hz, 2H), 7.13-7.23 (m, 3H), 7.36-7.46 (m, 2H), 7.63-7.73 (m, 2H), 8.27 (d, J = 13.3 Hz, 1H): **^{13}C NMR** (125 MHz, CD3OD) (as a mixture of rotamers) δ□ 24.6, 25.8, 31.0, 42.4, 43.8, 47.4, 51.2, 51.9, 53.8, 54.3, 99.3, 119.3, 120.1, 120.7, 125.2, 128.9, 131.2, 131.4, 131.4, 146.3, 155.1, 155.2, 156.2, 156.5, 158.1, 160.1, 168.0. **HR-MS** (m/z): calculated for $C_{24}H_{24}ClN_6O_2$ [M+H]+: 463.1649; found: [M+H]+:463.1645.

(R)-1-(3-(4-amino-3-(4-phenoxyphenyl)-1H-pyrazolo[3,4-d]pyrimidin-1-yl)piperidin-1-yl)-2-hydroxyethan-1-one (pre-3)

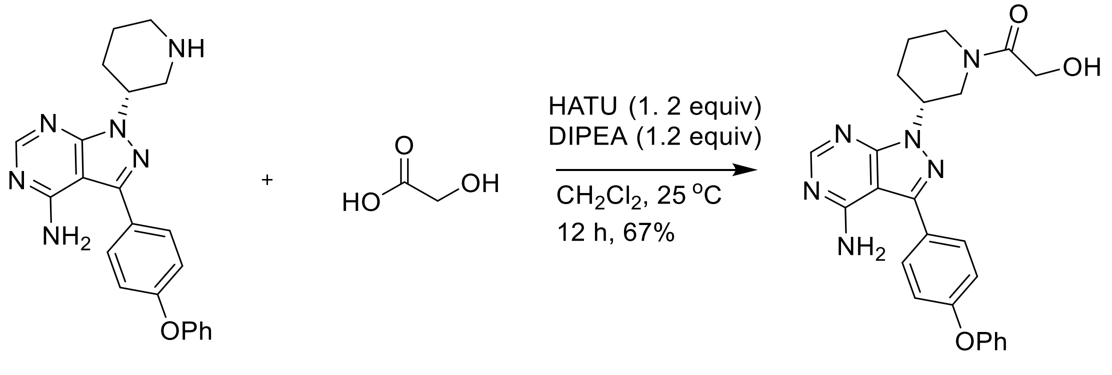

pre-3

To a stirred solution of hydroxyl acetic acid (225 mg, 3 mmol) in anhydrous DCM (6 mL), HATU (1368 mg, 3.6 mmol) and DIPEA (615 µL, 3.6 mmol) and Ibr-H (1161 mg, 3 mmol) were added at 25 °C. The reaction mixture was stirred at room temperature for 12 h. After completion of the reaction (as monitored by LC-MS), the reaction mixture was evaporated under *vacuo* and the crude product was purified using combi flash column chromatography with MeOH:EtOAc (2:8) as eluent to give Pre-3 was colorless solid in 894 mg (yield = 67 %).

^{1}H NMR (500 MHz, CD_3OD) δ: 1.47-1.70 (m, 1H), 1.89-1.97 (m, 1H), 2.12 (br. s., 1H), 2.15-2.32 (m, 1H), 3.10 (t, J = 11.4 Hz, 1H), 3.35-3.45 (m, 1H), 3.52-3.66 (m, 1H), 4.08-4.30 (m, 2H), 4.42 (d, J = 10.3 Hz, 1H), 4.79 (br. s., 1H), 5.20 (br. s., 1H), 6.90-7.14 (m, 5H), 7.27 (t, J = 7.7 Hz, 2H), 7.54 (d, J = 7.8 Hz, 2H), 8.28 (d, J = 19.8 Hz, 1H)

^{13}C NMR (126 MHz, CD_3OD) δ: 24.4, 25.3, 30.8, 43.4, 45.3, 47.1, 54.4, 54.8, 61.3, 61.5, 98.2, 120.1, 120.7, 125.4, 127.3, 131.2, 131.3, 148.3, 153.3, 154.6, 157.7, 160.5, 172.5, 172.6. : **ESI-MS** (m/z): calculated for $C_{24}H_{25}N_6O_3$ [M+H]+: 445.19; found: [M+H]+:445.16.

(R)-2-(3-(4-amino-3-(4-phenoxyphenyl)-1H-pyrazolo[3,4-d]pyrimidin-1-yl)piperidin-1-yl)-2-oxoethyl methanesulfonate (3b)

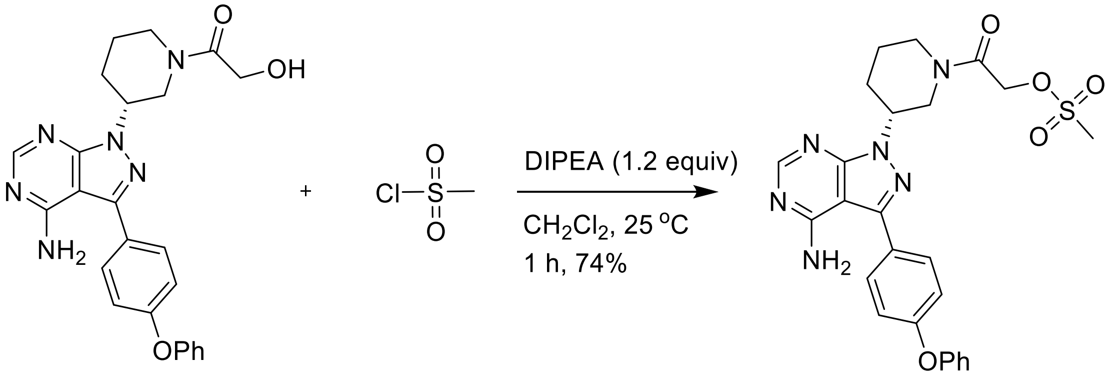

3b

To a stirred solution of **Pre-3** (23 mg, 0.05 mmol) in CH_2Cl_2 (1 mL), methane sulfonyl chloride (4.6 µL, 0.06 mmol, d = 1.48), and DIPEA (10.2 µL, 0.06 mmol) were added at 25 °C. The reaction mixture was stirred at room temperature for 1 h. After completion of the reaction (as monitored by LC-MS), water (1 mL) was added. The aqueous layer was extracted with CH_2Cl_2 (3 × 2 mL). The combined organic layer was concentrated *in vacuo* and the crude product was purified by preparative HPLC using water:ACN (0.1% formic acid) solvent gradient to afford white solid **3b** in 19.3 mg (74% yield).

^{1}H NMR (400 MHz, CD_3OD) (as a mixture of rotamers): δ 1.67 -1.90 (m, 1H), 1.98-2.20 (m, 1H), 2.21-2.32 (m, 1H), 2.36-2.49 (m, 1H), 3.12-3.23 (m, 3H), 3.35-3.43 (m, 1H), 3.63 (dd, J = 12.8, 9.5 Hz, 0.7H), 3.76 (d, J = 13.6 Hz, 0.7H), 3.84-3.93 (m, 0.5H), 3.94-4.01 (m, 0.5H), 4.07 (d, J = 13.4 Hz, 0.5H), 4.46 (dd, J = 12.8, 3.3 Hz, 0.5H), 4.96 (br. s., 1H), 4.99-5.15 (m, 2H), 7.13 (d, J = 8.1 Hz, 2H), 7.16-7.25 (m, 3H), 7.46 (s, 2H), 7.71 (d, J = 8.4 Hz, 2H), 8.38-8.44 (m, 1H): **^{13}C NMR** (101 MHz, CD_3OD) δ: 24.0, 25.3, 30.7, 38.5, 43.6, 46.1, 47.2, 54.2, 54.6, 67.6, 98.5, 120.2, 120.8, 125.4, 127.6, 131.3, 131.5, 148.3, 149.4, 150.0, 153.6, 157.9, 160.7, 166.8. **HR-MS** (m/z): Calculated for $C_{25}H_{27}N_6O_5S$ [M+H]+: 523.1764; found: [M+H]+:523.1762.

(R)-2-(3-(4-amino-3-(4-phenoxyphenyl)-1H-pyrazolo[3,4-d]pyrimidin-1-yl)piperidin-1-yl)-2-oxoethyl methylsulfamate (3c):

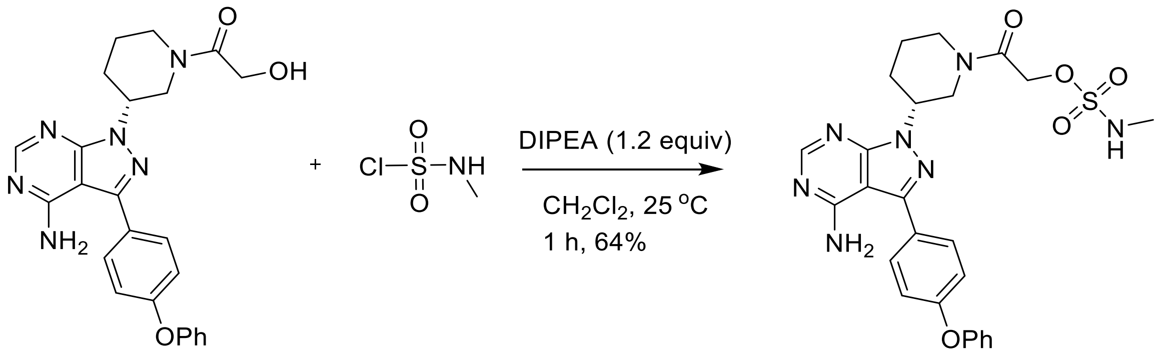

3c

To a stirred solution of **Pre-3** (230 mg, 0.5 mmol) in CH_2Cl_2 (1 mL), *N*-methylsulfamoyl chloride (77.4 mg, 0.6 mmol), and DIPEA (102 µL, 0.6 mmol) were added at 25 °C. The reaction mixture was stirred at room temperature for 1 h. After completion of the reaction (as monitored by LC-MS), water (1 mL) was added. The aqueous layer was extracted with CH_2Cl_2 (3 × 2 mL). The combined organic layer was concentrated *in vacuo* and the crude product was purified by preparative HPLC using water:ACN (0.1% formic acid) solvent gradient to afford white solid **3c** in 171 mg (64% yield).

^{1}H NMR (400 MHz, CD_3OD) (as a mixture of rotamers) δ 1.73-1.83 (d, J = 10.1 Hz, 1H), 2.02-2.19 (m, 1H), 2.22-2.32 (m, 1H), 2.40 (br. s., 1H), 2.68 (s, 1.2H), 2.74 (s, 1.6H), 3.39 (d, J = 12.5 Hz, 0.5H), 3.61 (dd, J = 12.2, 10.0 Hz, 0.7H), 3.76-3.94 (m, 1H), 3.97-4.13 (m, 1H), 4.48 (dd, J = 12.8, 3.3 Hz, 0.7H), 4.77-4.84 (m, 0.4H), 4.95 (dd, J = 9.4, 4.7 Hz, 0.7H), 5.04 (td, J = 8.3, 4.2 Hz, 0.5H), 7.13 (d, J = 7.9 Hz, 2H), 7.16-7.24 (m, 3H), 7.40-7.47 (m, 2H), 7.71 (d, J = 8.4 Hz, 2H), 8.40 (s, 1H): **^{13}C NMR** (101 MHz, CD_3OD) δ: 24.1, 25.4, 29.7, 30.6, 30.7, 43.6, 46.4, 47.2, 54.2, 54.6, 67.4, 67.6, 98.8, 120.2, 120.8, 125.4, 127.7, 127.7, 131.3, 131.5, 149.9, 150.0, 150.6, 153.7, 157.9, 160.7, 166.8, 167.0. **HR-MS** (m/z): calculated for $C_{25}H_{28}N_7O_5S$ [M+H]+: 538.1873; found: [M+H]+:538.1875.

(R)-2-(3-(4-amino-3-(4-phenoxyphenyl)-1H-pyrazolo[3,4-d]pyrimidin-1-yl)piperidin-1-yl)-2-oxoethyl benzylsulfamate (3d):

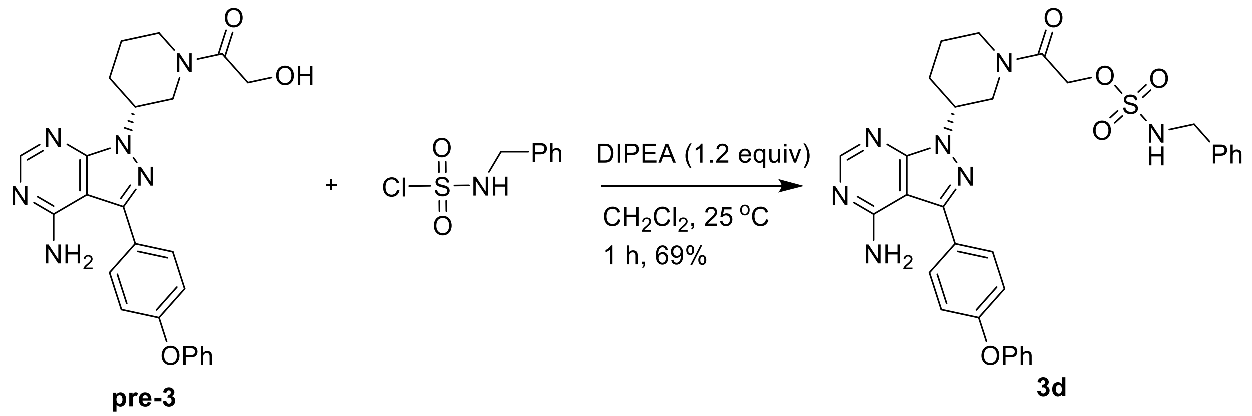

To a stirred solution of **Pre-3** (23 mg, 0.05 mmol) in CH_2Cl_2 (1 mL), benzylsulfamoyl chloride (12.3 mg, 0.06 mmol), and DIPEA (10.2 μL, 0.06 mmol) were added at 25 °C. The reaction mixture was stirred at room temperature for 1 h. After completion of the reaction (as monitored by LC-MS), water (1 mL) was added. The aqueous layer was extracted with CH_2Cl_2 (3 × 2 mL). The combined organic layer was concentrated *in vacuo* and the crude product was purified by preparative HPLC using water:ACN (0.1% formic acid) solvent gradient to afford white solid **3d** in 21.1 mg (69% yield).

^{1}H NMR (400 MHz, CD3OD) δ: 1.75 (dd, J = 17.2, 3.7 Hz, 1H), 1.98-2.17 (m, 1H), 2.18-2.32 (m, 1H), 2.32-2.46 (m, 1H), 3.35-3.43 (m, 0.5H), 3.60 (dd, J = 12.8, 9.5 Hz, 0.5H), 3.69 (d, J = 13.9 Hz, 0.5H), 3.85 (dd, J = 13.6, 8.1 Hz, 0.5H), 3.94 (dd, J = 13.8, 3.6 Hz, 0.5H), 4.04 (d, J = 13.2 Hz, 0.5H), 4.19 (s, 1H), 4.27 (s, 1H), 4.44 (dd, J = 12.9, 3.2 Hz, 0.6H), 4.60-4.60 (m, 0.5H) 4.67-4.79 (m, 2H), 4.92-4.98 (m, 0.7H), 4.99-5.07 (m, 0.5H), 7.12 (d, J = 7.9 Hz, 2H), 7.14-7.23 (m, 3H), 7.31 (d, J = 4.2 Hz, 2H), 7.37 (d, J = 4.6 Hz, 2H), 7.40-7.47 (m, 2H), 7.65-7.76 (m, 2H), 8.40 (d, J = 12.1 Hz, 1H): **^{13}C NMR** (101 MHz, CD3OD) δ: 24.0, 25.3, 30.6, 30.7, 43.6, 46.3, 47.2, 50.0, 54.2, 54.6, 67.3, 67.6, 98.8, 120.2, 120.8, 125.4, 127.5, 129.0, 129.2, 129.4, 129.8, 131.3, 131.5, 138.8, 149.3, 150.1, 153.6, 155.3, 157.9, 160.7, 166.8. **HR-MS** (m/z): calculated for $C_{31}H_{32}N_7O_5S$ [M+H]+: 614.2186; found: [M+H]+:614.2188.

(R)-2-(3-(4-amino-3-(4-phenoxyphenyl)-1H-pyrazolo[3,4-d]pyrimidin-1-yl)piperidin-1-yl)-2-oxoethyl phenylsulfamate (3e)

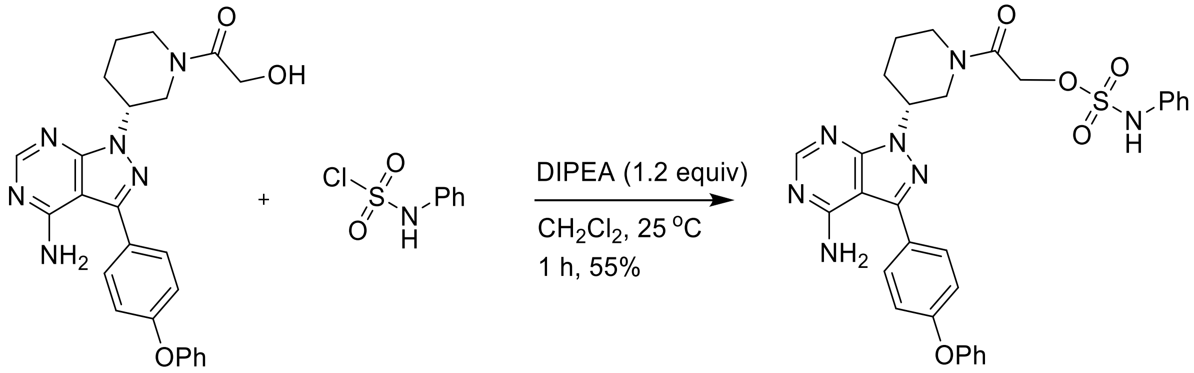

To a stirred solution of **Pre-3** (23 mg, 0.05 mmol) in CH_2Cl_2 (1 mL), phenylsulfamoyl chloride (11.5 mg, 0.06 mmol), and DIPEA (10.2 μL, 0.06 mmol) were added at 25 °C. The reaction mixture was stirred at room temperature for 1 h. After completion of the reaction (as monitored by LC-MS), water (1 mL) was added. The aqueous layer was extracted with CH_2Cl_2 (3 × 1 mL). The combined organic layer was concentrated *in vacuo* and the crude product was purified by preparative HPLC using water:ACN (0.1% formic acid) solvent gradient to afford white solid **3e** in 16.4 mg (55% yield).

^{1}H NMR (500 MHz, CD3OD) (as a mixture of rotamers) δ: 1.60-1.72 (m, 1H), 1.96-2.07 (m, 1H), 2.15-2.25 (m, 1H), 2.29-2.40 (m, 1H), 3.21-3.28 (m, 1H), 3.48 (d, J = 13.6 Hz, 1H), 3.73-3.81 (m, 1H), 3.89 (d, J = 9.9 Hz, 0.6H), 4.02 (d, J = 13.1 Hz, 0.6H), 4.40 (d, J = 12.5 Hz, 0.6H), 4.73-4.84 (m, 2H), 7.02 (t, J = 7.2 Hz, 1H), 7.09 (d, J = 8.0 Hz, 1H), 7.13-7.24 (m, 6H), 7.26 (d, J = 8.0 Hz, 1H), 7.35 (t, J = 7.8 Hz, 1H), 7.44 (t, J = 7.1 Hz, 2H), 7.65-7.74 (m, 2H), 8.37 (d, J = 8.0 Hz, 1H). **HR-MS** (m/z): calculated for $C_{30}H_{30}N_7O_5S$ [M+H]+: 600.2029; found: [M+H]+:600.2032.

Synthesis of (R)-1-(3-(4-amino-3-(4-phenoxyphenyl)-1H-pyrazolo[3,4-d]pyrimidin-1-yl)piperidin-1-yl)-2-(methylsulfonyl)ethan-1-one (3f):

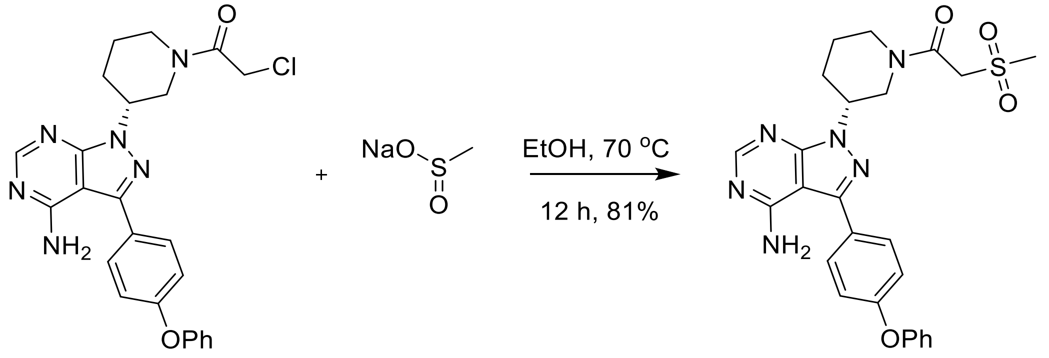

3a **3f**

To a stirred solution of **3a** (23 mg, 0.05 mmol) in ethanol (1 mL), sodium methanesulfinate (10.2 mg, 0.1 mmol) was added at 25 °C. The reaction mixture was stirred at room temperature for 12 h at 70 °C. After completion of the reaction (as monitored by LC-MS), ethanol was concentrated *in vacuo* and the crude product was purified by preparative HPLC using water:ACN (0.1% formic acid) solvent gradient to afford white solid **3f** in 20.4 mg (81% yield).

^{1}**H NMR** (400 MHz, CD3OD) (as a mixture of rotamers) δ: 1.71-1.77 (m, 0.5H),1.83-1.94 (m, 0.5H), 1.97-2.03 (m, 0.5H), 2.06-2.15 (m, 0.5H), 2.21-2.31 (m, 1H), 2.32-2.44 (m, 1H), 3.12 (d, J = 2.6 Hz, 3H), 3.17-3.24 (m, 0.5H), 3.38-3.47 (m, 0.5H), 3.58 (dd, J = 12.7, 10.0 Hz, 0.6H), 3.95-4.09 (m, 1H), 4.23-4.38 (m, 0.5H), 4.32 (d, J = 14.5 Hz, 1H), 4.39-4.52 (m, 1.5H), 4.60 (dd, J = 12.8, 3.5 Hz, 0.6H), 4.91-4.98 (m, 0.6H), 5.06-5.12 (m, 1H), 7.13 (d, J = 8.1 Hz, 2H), 7.16-7.24 (m, 3H), 7.36-7.47 (m, 2H), 7.71 (dd, J = 8.6, 4.2 Hz, 2H), 8.41 (d, J = 3.7 Hz, 1H). ^{13}**C NMR** (101 MHz, CD3OD) (as a mixture of rotamers) δ: 24.4, 25.5, 31.0, 42.3, 43.7, 47.2, 51.7, 54.4, 55.0, 58.3, 98.5, 120.2, 120.8, 125.4, 127.6, 131.3, 131.5, 137.5 (m) 148.3, 149.5, 149.7, 153.6, 153.8, 157.9, 160.7, 163.5. **ESI-MS** (m/z): calculated for $C_{25}H_{27}N_6O_4S$ [M+H]+: 507.18; found: [M+H]+:507.19.

Synthesis of (R)-1-(3-(4-amino-3-(4-phenoxyphenyl)-1H-pyrazolo[3,4-d]pyrimidin-1-yl)piperidin-1-yl)-2-(phenylsulfonyl)ethan-1-one (3g):

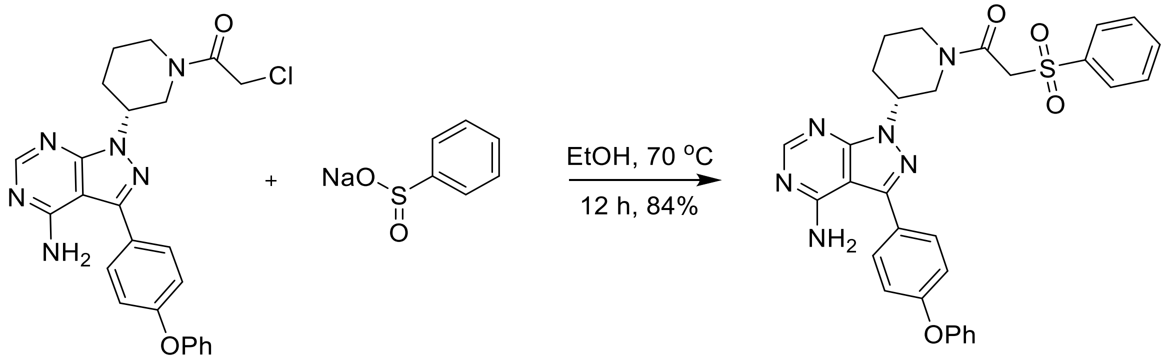

3a **3g**

To a stirred solution of **3a** (23 mg, 0.05 mmol) in ethanol (1 mL), sodium phenylsulfinate (10.2 mg, 0.1 mmol) was added at 25 °C. The reaction mixture was stirred at room temperature for 12 h at 70 °C. After completion of the reaction (as monitored by LC-MS), ethanol was concentrated *in vacuo* and the crude product was purified by preparative HPLC using water:ACN (0.1% formic acid) solvent gradient to afford white solid **3g** in 23.8 mg (84% yield).

^{1}H NMR (500 MHz, CD3OD) δ: 1.61-1.72 (m, 0.5H), 1.76-1.86 (m, 0.5H), 1.93-2.01 (m, 0.5H), 2.04-2.12 (m, 0.5H), 2.19-2. 29 (m, 1H), 2.29-2.41 (m, 1H), 3.09-3.14 (m, 0.5H), 3.35 (m, 0.5H), 3.50 (dd, *J* = 12.8, 10.0 Hz, 0.5H), 4.03 (d, *J* = 13.9 Hz, 0.6H), 4.19-4.29 (m, 1H), 4.37-4.40 (m, 0.5H), 4.51 (d, *J* = 14.3 Hz, 1H), 4.58-4.68 (m, 1H), 4.80-4.81 (m, 0.5H), 5.05-5.15 (m, 0.5H), 7.09-7.15 (m, 2H), 7.15-7.24 (m, 3H), 7.40-7.47 (m, 2H), 7.60-7.68 (m, 2H), 7.71 (d, *J* = 8.5 Hz, 2H), 7.73-7.79 (m, 1H), 7.93 (d, *J* = 8.4 Hz, 1H), 7.98 (d, *J* = 8.5 Hz, 1H), 8.43 (d, *J* = 10.7 Hz, 1H): **^{13}C NMR** (126 MHz, CD3OD) δ: 24.4, 25.5, 30.7, 31.0, 43.8, 47.3, 51.8, 54.5, 55.1, 60.3, 98.4, 98.5, 120.2, 120.9, 125.4, 127.4, 129.6, 130.6, 131.3, 131.4, 131.5, 135.6, 140.7, 140.9, 148.4, 148.5, 148.8, 149.0, 153.5, 153.7, 154.9, 155.1, 157.9, 160.8, 162.7, 162.8. **ESI-MS** (m/z): calculated for $C_{30}H_{29}N_6O_4S$ [M+H]+: 569.20; found: [M+H]+:569.29.

N-(1,1-dioxidotetrahydrothiophen-3-yl)-2-hydroxy-N-neopentylacetamide (Pre-4):

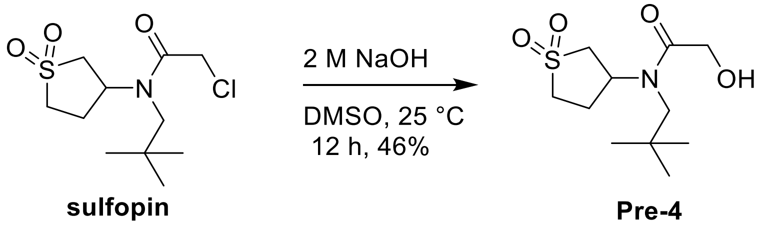

To a stirred solution of sulfopin (281 mg, 1 mmol) in DMSO (2 mL), NaOH (5 M, 2 mL) was added at 25 °C. The reaction mixture was stirred at room temperature for 12 h. After completion of the reaction (as monitored by LC-MS), water (1 mL) was added. The aqueous layer was extracted with Ethyl acetate (3 × 3 mL). The combined organic layer was concentrated *in vacuo* and the crude product was purified by preparative HPLC using water:ACN (0.1% formic acid) solvent gradient to afford white solid **Pre-4** in 121 mg (46% yield).

^{1}H NMR (500 MHz, $CDCl_3$) δ: 1.01 (s, 10H), 2.49-2.64 (m, 2H), 2.92-2.99 (m, 1H), 3.02 (s, 1H), 3.05-3.10 (m, 1H), 3.16 (dd, J = 12.6, 8.3 Hz, 2H), 3.71 (dt, J = 12.9, 9.2 Hz, 1H), 3.79 (dd, J = 12.6, 9.8 Hz, 1H), 3.90-3.99 (m, 1H), 4.11-4.22 (m, 2H): **^{13}C NMR** (126 MHz, CDC13) δ: 26.6, 28.1, 33.6, 49.5, 50.5, 57.2, 60.1, 60.6, 173.2. : **ESI-MS** (m/z): calculated for $C_{11}H_{22}NO_4S$ [M+H]+: 264.12; found: [M+H]+:264.16.

2-((1,1-dioxidotetrahydrothiophen-3-yl)(neopentyl)amino)-2-oxoethyl methanesulfonate (4a):

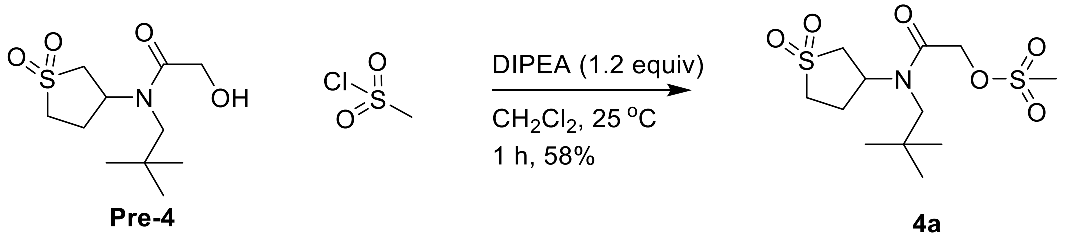

To a stirred solution of **Pre-4** (13 mg, 0.05 mmol) in CH_2Cl_2 (1 mL), methane sulfonyl chloride (4.6 µL, 0.06 mmol, d = 1.48), and DIPEA (10.2 µL, 0.06 mmol) were added at 25 °C. The reaction mixture was stirred at room temperature for 1 h. After completion of the reaction (as monitored by LC-MS), water (1 mL) was added. The aqueous layer was extracted with CH_2Cl_2 (3 × 1 mL). The combined organic layer was concentrated *in vacuo* and the crude product was purified by preparative HPLC using water:ACN (0.1% formic acid) solvent gradient to afford white solid **4a** in 9.8 mg (74% yield).

^{1}H NMR (500 MHz, CDCl3): *δ*: 1.03 (s, 9H), 2.50-2.59 (m, 2H), 3.01 (s, 1H), 3.04-3.19 (m, 3H), 3.27 (s, 3H), 3.64-3.83 (m, 2H), 3.93 (d, *J* = 8.0 Hz, 1H), 4.86 (s, 2H): **^{13}C NMR** (125 MHz, CDCl3): δ 26.6, 27.9, 33.6, 42.4, 49.3, 50.4, 58.1, 58.8, 63.1, 164.1. **HR-MS** (m/z): calculated for $C_{12}H_{24}NO_6S_2$ [M+Na]+: 342.1045; found: [M+Na]+:342.1045.

2-((1,1-dioxidotetrahydrothiophen-3-yl)(neopentyl)amino)-2-oxoethyl methylsulfamate (4b):

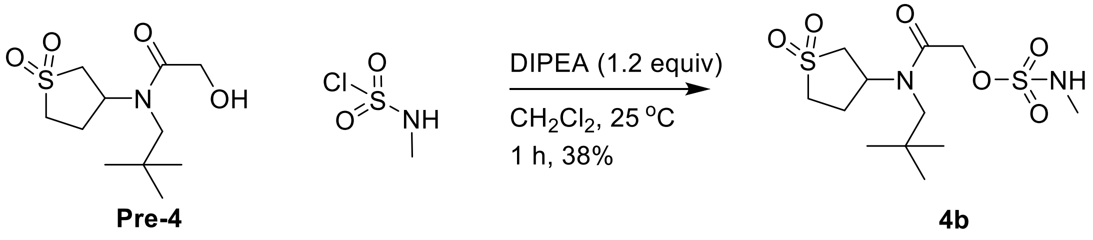

To a stirred solution of **pre-4** (13 mg, 0.05 mmol) in CH_2Cl_2 (1 mL), N-methylsulfamoyl chloride (7.74 mg, 0.06 mmol), and DIPEA (10.2 μL, 0.06 mmol) were added at 25 °C. The reaction mixture was stirred at room temperature for 1 h. After completion of the reaction (as monitored by LC-MS), water (1 mL) was added. The aqueous layer was extracted with CH_2Cl_2 (3 × 1 mL). The combined organic layer was concentrated *in vacuo* and the crude product was purified by preparative HPLC using water:ACN (0.1% formic acid) solvent gradient to afford white solid **4b** in 6.74 mg (38% yield). **^{1}H NMR** (500 MHz, $CDCl_3$) *δ*: 1.03 (s, 9H), 2.55 (d, *J* = 8.5 Hz, 2H), 2.91 (s, 3H), 2.97-3.09 (m, 2H), 3.09-3.20 (m, 2H), 3.63-3.71 (m, 1H), 3.73-3.82 (m, 1H), 3.85-3.98 (m, 1H), 4.80 (s, 2H), 5.21 (br. s., 1H): **^{13}C NMR** (125 MHz, $CDCl_3$) *δ*: 26.6, 28.0, 30.4, 33.7, 49.3, 50.5, 57.7, 61.3, 67.5, 168.0. : **HR-MS** (m/z): calculated for $C_{12}H_{24}N_2NaO_6S_2$ [M+Na]+: 379.0973; found: [M+Na]+:379.0964.

2-((1,1-dioxidotetrahydrothiophen-3-yl)(neopentyl)amino)-2-oxoethyl benzylsulfamate (4c):

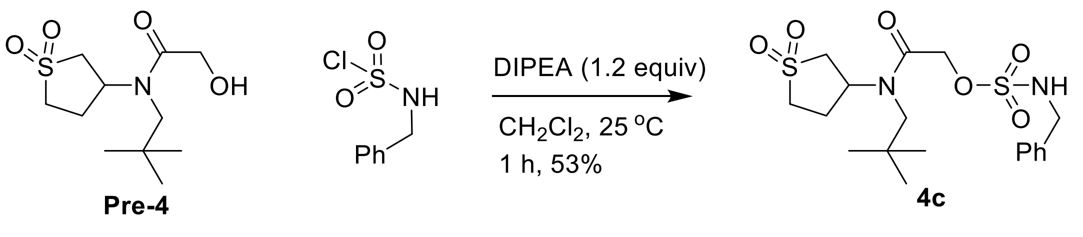

To a stirred solution of **Pre-4** (13 mg, 0.05 mmol) in CH_2Cl_2 (1 mL), benzylsulfamoyl chloride (12.3 mg, 0.06 mmol), and DIPEA (10.2 μL, 0.06 mmol) were added at 25 °C. The reaction mixture was

stirred at room temperature for 1 h. After completion of the reaction (as monitored by LC-MS), water (1 mL) was added. The aqueous layer was extracted with CH_2Cl_2 (3 × 1 mL). The combined organic layer was concentrated *in vacuo* and the crude product was purified by preparative HPLC using water:ACN (0.1% formic acid) solvent gradient to afford white solid **4c** in 11.4 mg (53% yield).

^{1}H NMR (500 MHz, $CDCl_3$) δ: 1.00 (s, 9H), 2.50 (m, 2H), 3.00 (m, 2H), 3.11 (m, 2H), 3.60 (m, 1H), 3.71 (m, 1H), 3.89 (quin, J = 7.9 Hz, 1H), 4.39 (d, J = 5.4 Hz, 2H), 4.76 (s, 2H), 5.64 (br. s., 1H), 7.35 (m, 5H): **^{13}C NMR** (125 MHz, $CDCl_3$) δ: 26.6, 28.0, 33.7, 48.2, 49.3, 50.4, 57.6, 61.2, 67.4, 128.1, 128.2, 128.8, 136.1, 167.7. **HR-MS** (m/z): calculated for $C_{18}H_{28}N_2NaO_6S_2$ [M+Na]+: 455.1286; found: [M+Na]+:455.1279.

2-((1,1-dioxidotetrahydrothiophen-3-yl)(neopentyl)amino)-2-oxoethyl phenylsulfamate (4d):

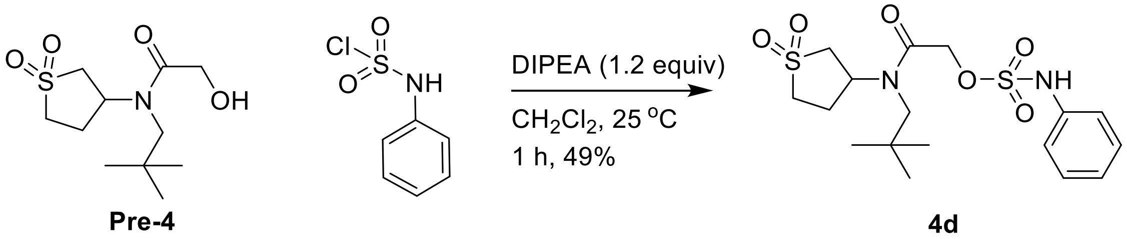

To a stirred solution of **pre-4** (13 mg, 0.05 mmol) in CH_2Cl_2 (1 mL), phenylsulfamoyl chloride (11.5 mg, 0.06 mmol), and DIPEA (10.2 µL, 0.06 mmol) were added at 25 °C. The reaction mixture was stirred at room temperature for 1 h. After completion of the reaction (as monitored by LC-MS), water (1 mL) was added. The aqueous layer was extracted with CH_2Cl_2 (3 × 2 mL). The combined organic layer was concentrated *in vacuo* and the crude product was purified by preparative HPLC using water:ACN (0.1% formic acid) solvent gradient to afford white solid **4d** in 10.2 mg (49% yield).

^{1}H NMR (500 MHz, CD3OD) δ: 0.98 (s, 10H), 2.45-2.52 (m, 2H), 2.57 (s, 1H), 3.05 (dt, J = 13.0, 6.4 Hz, 1H), 3.11-3.26 (m, 3H), 3.51-3.63 (m, 2H), 4.02 (quin, J = 8.0 Hz, 1H), 4.80 (s, 2H), 7.16 (s, 1H), 7.25 (d, J = 8.0 Hz, 2H), 7.35 (t, J = 7.8 Hz, 2H): ^{13}C NMR (126 MHz, CD3OD) δ: 27.8, 28.4, 34.5, 50.7, 51.9, 59.2, 61.7, 68.4, 121.1, 125.7, 130.5, 138.7, 145.7, 168.9. : **HR-MS** (m/z): calculated for $C_{17}H_{26}N_2NaO_6S_2$ [M+Na]+: 441.1130; found: [M+Na]+:441.1120.

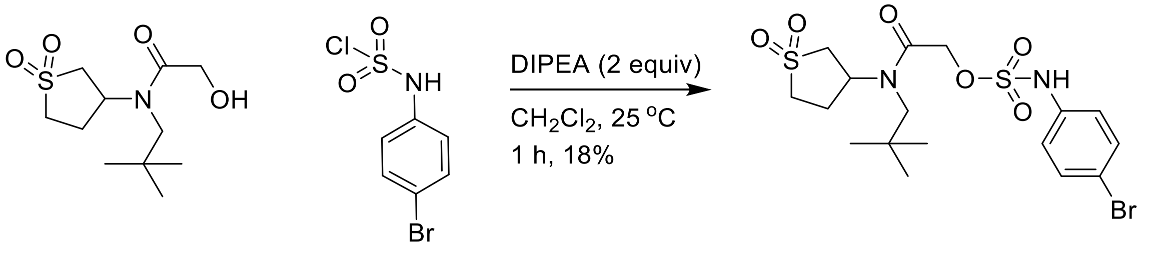

pre-4 crude **4e**

To a stirred solution of **Pre-4** (13 mg, 0.05 mmol) in CH_2Cl_2 (1 mL), 4-bromo phenylsulfamoyl chloride (see synthesis of **1g** for preparation) (27 mg, 0.1 mmol,), and DIPEA (17.6 µL, 0.1 mmol) were added at 25 °C. The reaction mixture was stirred at room temperature for 1 h. After completion of the reaction (as monitored by LC-MS), water (1 mL) was added. The aqueous layer was extracted with CH_2Cl_2 (3 × 2 mL). The combined organic layer was concentrated *in vacuo* and the crude product was purified by preparative HPLC using water:ACN (0.1% formic acid) solvent gradient to afford white solid **4d** in 4.4 mg (18% yield).

^{1}H NMR (500 MHz, CDCl3) δ: 1.02 (s, 9H), 2.50-2.63 (m, 2H), 2.98-3.05 (m, 1H), 3.05-3.13 (m, 2H), 3.13-3.21 (m, 1H), 3.62-3.71 (m, 1H), 3.73-3.81 (m, 1H), 3.87-3.98 (m, 1H), 4.89 (s, 2H), 7.22 (d, J = 8.8 Hz, 2H), 7.46-7.54 (m, 3H): **^{13}C NMR** (125 MHz, CDCl3) δ: 26.6, 28.1, 33.8, 49.5, 50.6, 57.9, 61.4, 68.8, 124.3, 132.5. quartnary carbons were not been detected. : **HR-MS** (m/z): calculated for $C_{17}H_{25}Br^{79}N_2NaO_6S_2$ [M+Na]+: 519.0235; found: [M+Na]+:519.0233, calculated for $C_{17}H_{25}Br^{81}N_2NaO_6S_2$ [M+Na]+: 521.0215; found: [M+Na]+:521.0214.

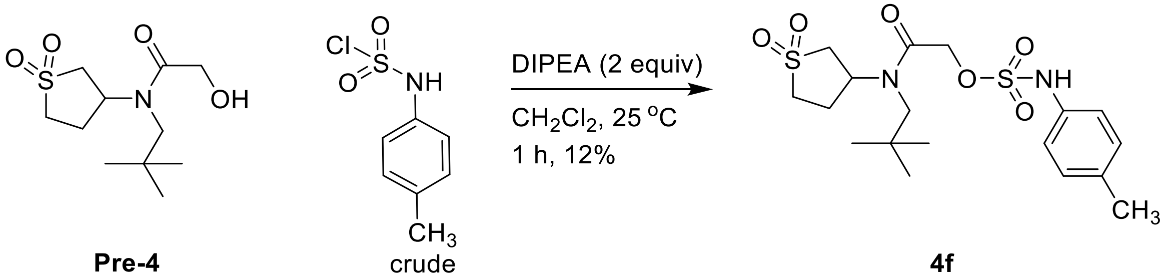

Pre-4 crude **4f**

To a stirred solution of **pre-4** (13 mg, 0.05 mmol) in CH_2Cl_2 (1 mL), 4-methyl phenylsulfamoyl chloride (see synthesis of **1g** for preparation) (11.5 mg, 0.06 mmol,), and DIPEA (10.2 µL, 0.06 mmol) were added at 25 °C. The reaction mixture was stirred at room temperature for 1 h. After completion of the reaction (as monitored by LC-MS), water (1 mL) was added. The aqueous layer was extracted with CH_2Cl_2 (3 × 2 mL). The combined organic layer was concentrated *in vacuo* and

the crude product was purified by preparative HPLC using water:ACN (0.1% formic acid) solvent gradient to afford white solid **4d** in 2.5 mg (12% yield).

^{1}H NMR (500 MHz, CD_3OD) δ: 0.95 (s, 10H), 1.26-1.38 (m, 2H), 2.29 (s, 3H), 2.42-2.51 (m, 2H), 3.03 (dt, J = 12.9, 6.5 Hz, 1H), 3.15-3.21 (m, 2H), 3.56 (dd, J = 13.4, 8.9 Hz, 2H), 3.95-4.05 (m, 1H), 4.71-4.80 (m, 2H), 7.08-7.21 (m, 5H): **^{13}C NMR** (126 MHz, CD_3OD) δ: 21.4, 28.2, 28.9, 32.3, 51.2, 52.4, 59.7, 62.2, 68.9, 122.3, 131.5, 168.5. : **HR-MS** (m/z): calculated for $C_{18}H_{29}N_2O_6S_2$ [M+H]+: 433.1467; found: [M+H]+:433.1467.

2-((cyclohexylmethyl)(1,1-dioxidotetrahydrothiophen-3-yl)amino)-2-oxoethyl phenylsulfamate (4g)

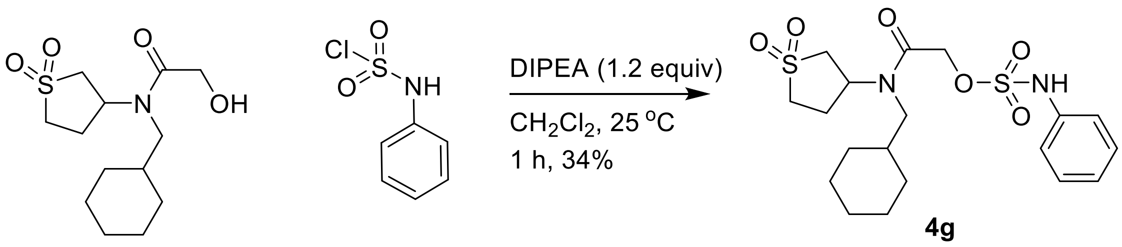

To a stirred solution of alcohol (14.5 mg, 0.05 mmol) in CH_2Cl_2 (1 mL), phenylsulfamoyl chloride (11.5 mg, 0.06 mmol), and DIPEA (10.2 µL, 0.06 mmol) were added at 25 °C. The reaction mixture was stirred at room temperature for 1 h. After completion of the reaction (as monitored by LC-MS), water (1 mL) was added. The aqueous layer was extracted with CH_2Cl_2 (3 × 2 mL). The combined organic layer was concentrated *in vacuo* and the crude product was purified by preparative HPLC using water:ACN (0.1% formic acid) solvent gradient to afford white solid **4g** in 7.5 mg (34% yield).

^{1}H NMR (500 MHz, $CDCl_3$) δ: 0.87 (d, J = 14.3 Hz, 3H), 0.91-0.98 (m, 2H), 1.13-1.33 (m, 6H), 1.54 (br. s., 2H), 2.41-2.65 (m, 2H), 2.99-3.10 (m, 3H), 3.12 (s, 1H), 3.16 (br. s., 1H), 3.58 (d, J = 8.1 Hz, 1H), 3.64-3.76 (m, 1H), 4.00-4.11 (m, 1H), 4.86 (s, 2H), 7.26-7.46 (m, 5H): **^{13}C NMR** (126 MHz, $CDCl_3$) δ: 25.7, 26.0, 26.2, 30.7, 30.8, 37.8, 49.9, 50.7, 55.5, 55.9, 68.6, 122.4, 126.1, 129.4, 144.7: **HR-MS** (m/z): calculated for $C_{19}H_{28}N_2NaO_6S_2$ [M+Na]+: 467.1286; found: [M+Na]+:467.1286.

Synthesis of 3h

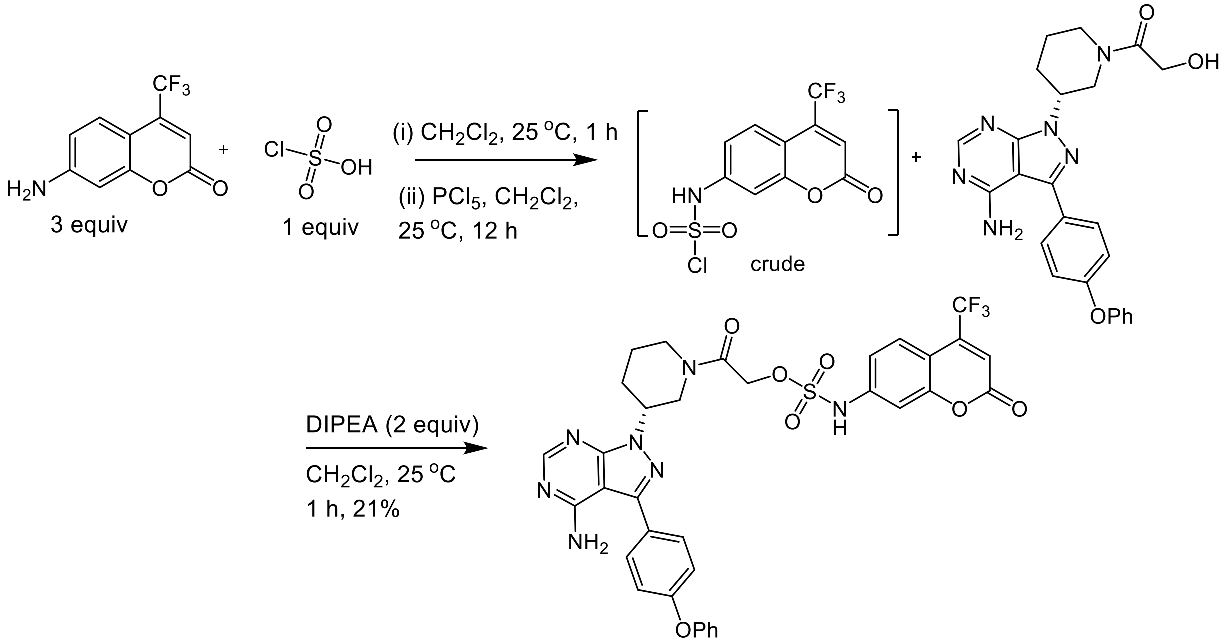

3h

To a stirred solution of amine (69 mg, 0.3 mmol) in CH_2Cl_2 (1 mL), chloro methane sulfonyl chloride (7.7 µL, 0.1 mmol,) was added at 0 °C. The reaction mixture was stirred at room temperature for 1 h. After completion of the reaction organic layer was concentrated *in vacuo*. The crude product was dissolved CH_2Cl_2 (1 mL) and PCl_5 (20 mg, 0.1 mmol) was added at 0 °C. The reaction mixture was stirred at room temperature for 2 h. After completion of the reaction (as monitored by LC-MS), the reaction mixture is filtered and washed with dichloromethane. The filtrate was concentrated and used as such for the next reaction.

To a stirred solution of **Pre-3** (23 mg, 0.05 mmol) in CH_2Cl_2 (1 mL), 7-amino, 4-trifluoro coumarine sulfamoyl chloride (0.1 mmol), and DIPEA (17.5 µL, 0.1 mmol) were added at 25 °C. The reaction mixture was stirred at room temperature for 1 h. After completion of the reaction (as monitored by LC-MS), water (1 mL) was added. The aqueous layer was extracted with CH_2Cl_2 (3 × 2 mL). The combined organic layer was concentrated *in vacuo* and the crude product was purified by preparative HPLC using water:ACN (0.1% formic acid) solvent gradient to afford white solid **3h** in 7.5 mg (21% yield).

^{1}H NMR (500 MHz, DMSO-d_6): δ 1.64-1.76 (m, 1H), 1.77-1.93 (m, 1H), 2.05-2.29 (m, 2H), 3.07 - 3.17 (m, 1.4H), 3.87 (d, J = 12.4 Hz, 0.6H), 4.08 (br. s., 0.6H), 4.12-4.14 (m, 0.7 H). 4.37 (br. s.,

0.7H), 4.42-4.51 (m, 0.5H), 4.59 (br. s., 0.5H), 4.68-4.71 (m, 0.4H), 4.77-4.94 (m, 1H), 4.98-5.09 (m, 1H), 6.59-6.71 (br. s., 2H), 6.89 (s, 1H), 6.93 (s, 1H), 7.10 - 7.25 (m, 4H), 7.28 (s, 1H), 7.44 (t, *J*=7.9 Hz, 2H), 7.60-7.76 (m, 2H), 7.96 (s, 1H), 8.28 (d, *J*=6.7 Hz, 1H), 11.52 (s, 1H): **^{13}C NMR** (126 MHz, DMSO-*d*$_6$) δ: 20.5, 24.8, 31.2, 36.2, 42.5, 52.5, 67.2, 99.4, 104.7, 108.4, 112.7, 114.9, 115.1, 119.5, 121.0, 124.3, 126.3, 126.4, 128.1, 130.6, 139.4, 142.4, 154.6, 155.4, 158.9, 159.9, 174.0: **HR-MS** (m/z): calculated for $C_{34}H_{29}F_3N_7O_7S$ [M+H]+: 736.1801; found: [M+H]+:736.1802.

methyl (R)-3-(4-amino-3-(4-phenoxyphenyl)-1H-pyrazolo[3,4-d]pyrimidin-1-yl)piperidine-1-sulfonate (3i):

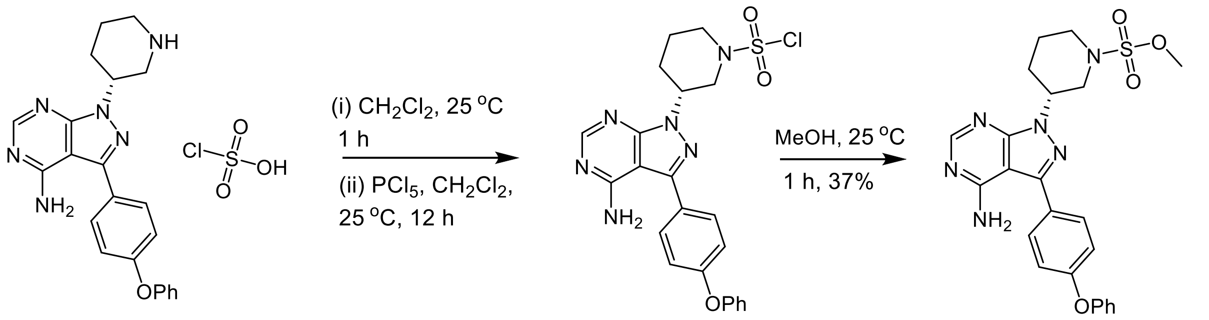

To a stirred solution of amine (116 mg, 0.3 mmol) in CH_2Cl_2 (1 mL), chloro methane sulfonyl chloride (7.7 µL, 0.1 mmol,) was added at 0 °C. The reaction mixture was stirred at room temperature for 1 h. After completion of the reaction organic layer was concentrated *in vacuo*. The crude product was dissolved CH_2Cl_2 (1 mL) and PCl_5 (20 mg, 0.1 mmol) was added at 0 °C. The reaction mixture was stirred at room temperature for 2 h. After completion of the reaction (as monitored by LC-MS), the reaction mixture is filtered and washed with dichloromethane. The filtrate was concentrated and used as such for the next reaction. 1/3rd of the crude product was dissolved in MeOH and allowed it to stir for 1 hour. After completion of the reaction (as monitored by LC-MS), the reaction mixture is concentrated *in vacuo* and the crude product was purified by preparative HPLC using water:ACN (0.1% formic acid) solvent gradient to afford white solid **3i** in 7.5 mg (21% yield).

^{1}H NMR (500 MHz, CD_3OD) *δ*: 1.91 (q, *J* = 12.7 Hz, 1H), 2.02-2.10 (m, 1H), 2.19-2.26 (m, 1H), 2.26-2.33 (m, 1H), 3.07 (t, *J* = 12.2 Hz, 1H), 3.52 (t, *J* = 11.3 Hz, 1H), 3.81 (d, *J* = 12.7 Hz, 1H), 3.88 (s, 3H), 3.97 (d, *J* = 11.7 Hz, 1H), 5.07 (br. s., 1H), 7.13 (d, *J* = 7.7 Hz, 2H), 7.16-7.27 (m, 4H), 7.44 (t, *J* = 7.3 Hz, 2H), 7.71 (d, *J* = 7.3 Hz, 2H), 8.40 (s, 1H): **^{13}C NMR** (126 MHz, CD_3OD) *δ*:

23.5, 28.8, 46.4, 49.7, 52.9, 56.1, 97.2, 118.6, 119.3, 123.9, 126.2, 129.7, 129.9, 146.4, 149.4, 152.5, 156.4, 159.1. **HR-MS** (m/z): calculated for $C_{23}H_{25}N_6O_4S$ [M+H]+: 481.1658; found: [M+H]+:481.1655.

prop-2-yn-1-yl (R)-3-(4-amino-3-(4-phenoxyphenyl)-1H-pyrazolo[3,4-d]pyrimidin-1-yl)piperidine-1-sulfonate (3j):

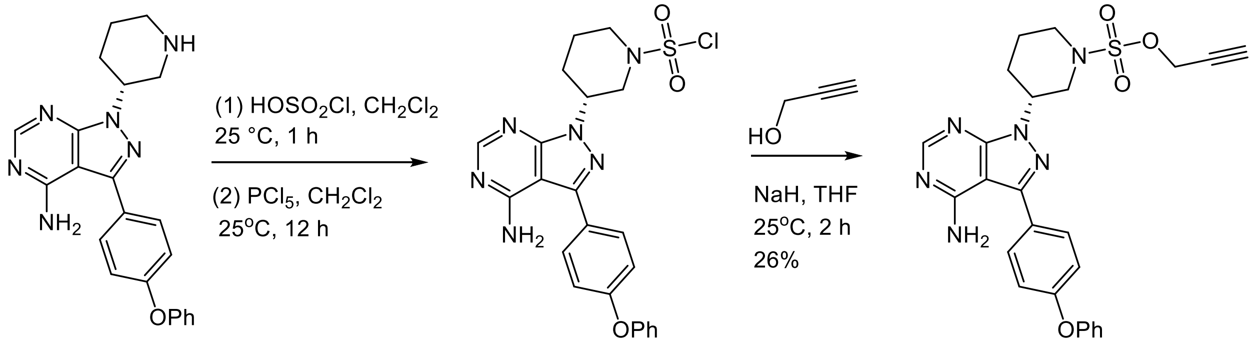

The synthesized Ibrutinib sulfamoyl chloride (1/3rd of the crude from the previous step, 0.1 mmol) was dissolved in THF (0.3 mL) followed by the addition of propargyl alcohol (27.2 mg, 10 mmol) and sodium hydride (10 mmol, 40 mg) at 0 °C. The reaction mixture was stirred at room temperature for 2 h. After completion of the reaction (as monitored by LC-MS), the reaction mixture is filtered and washed with dichloromethane. The filtrate was concentrated and purified by preparative HPLC using water:ACN (0.1% formic acid) solvent gradient to afford white solid **3j** in 13.1 mg (6% yield). ^{1}**H NMR** (500 MHz, CD_3OD) δ: 1.84-1.97 (m, 1H), 2.03-2.10 (m, 1H), 2.21 (dd, J = 12.7, 3.2 Hz, 1H), 2.31 (qd, J = 12.2, 3.8 Hz, 1H), 3.10 (td, J = 12.2, 2.5 Hz, 1H), 3.23 (t, J = 2.3 Hz, 1H), 3.56 (t, J = 11.3 Hz, 1H), 3.81 (d, J = 12.7 Hz, 1H), 3.97 (dd, J = 11.9, 3.9 Hz, 1H), 4.83 (d, J = 2.3 Hz, 2H), 5.00-5.09 (m, 1H), 7.12 (m, J = 8.1 Hz, 2H), 7.15-7.24 (m, 3H), 7.43 (t, J = 7.8 Hz, 2H), 7.70 (m, J = 8.5 Hz, 2H), 8.38 (s, 1H): ^{13}**C NMR** (125 MHz, CD_3OD) δ: 25.0, 30.3, 47.9, 51.2, 54.3, 59.3, 79.0, 97.4, 120.2, 120.8, 125.4, 127.3, 131.3, 131.5, 151.7, 155.9, 166.9. : **HR-MS** (m/z): calculated for $C_{25}H_{25}N_6O_4S$ [M+H]+: 505.1658; found: [M+H]+:481.1652.

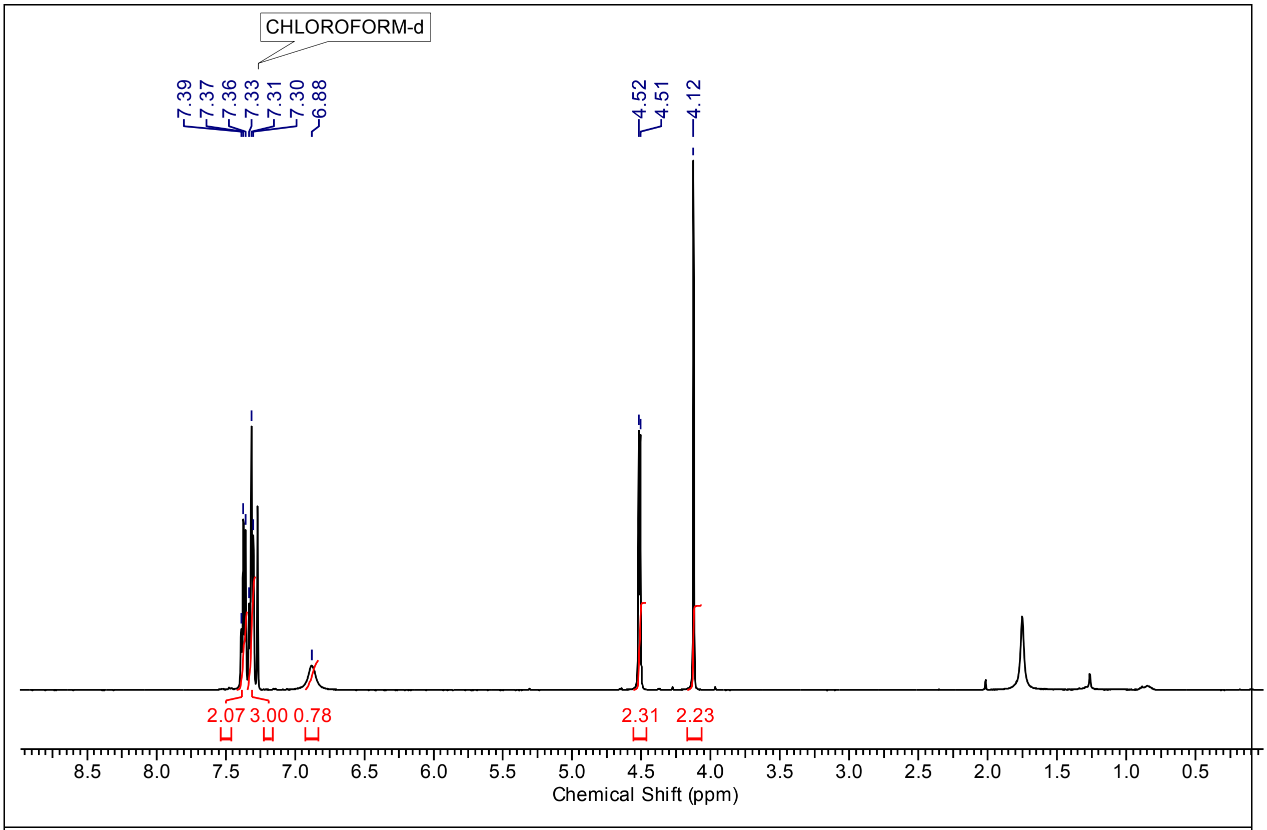

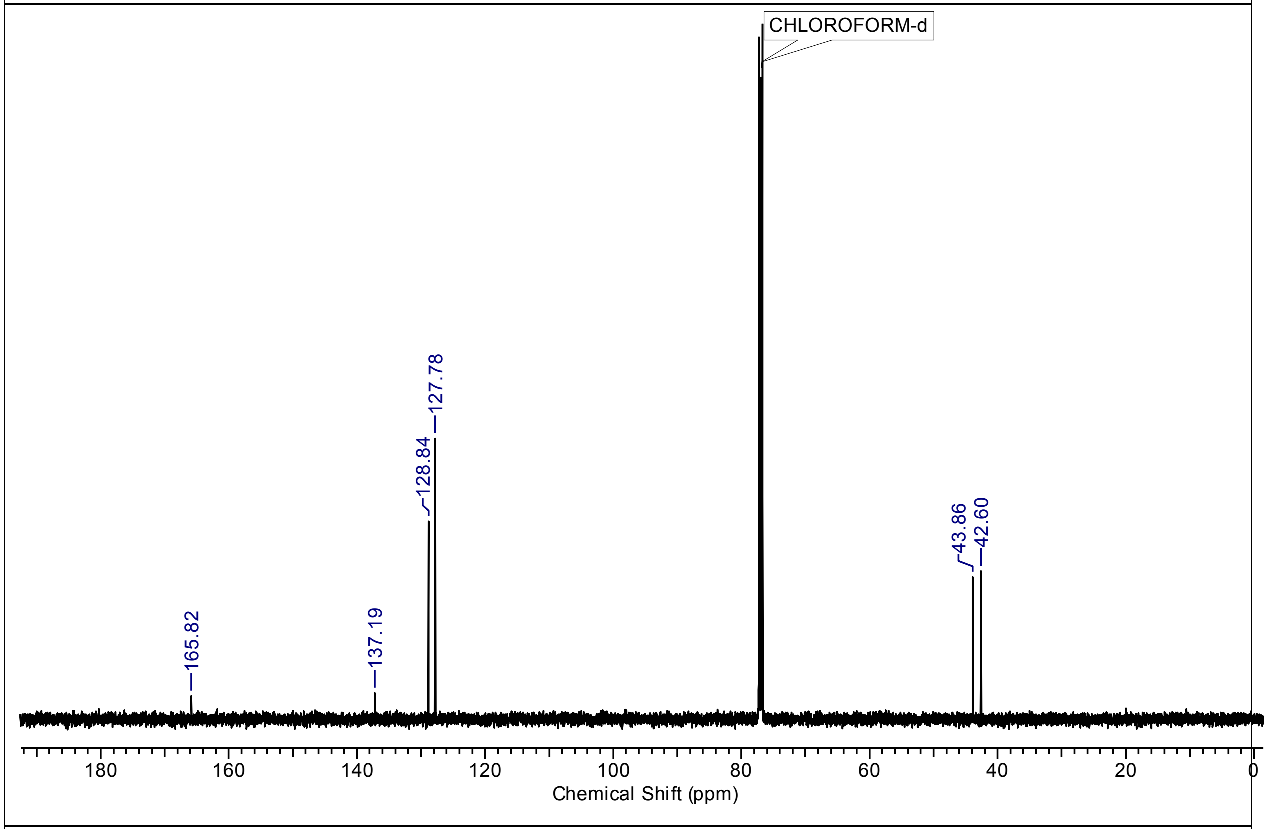

^{1}H and ^{13}C NMR spectra of **1a**

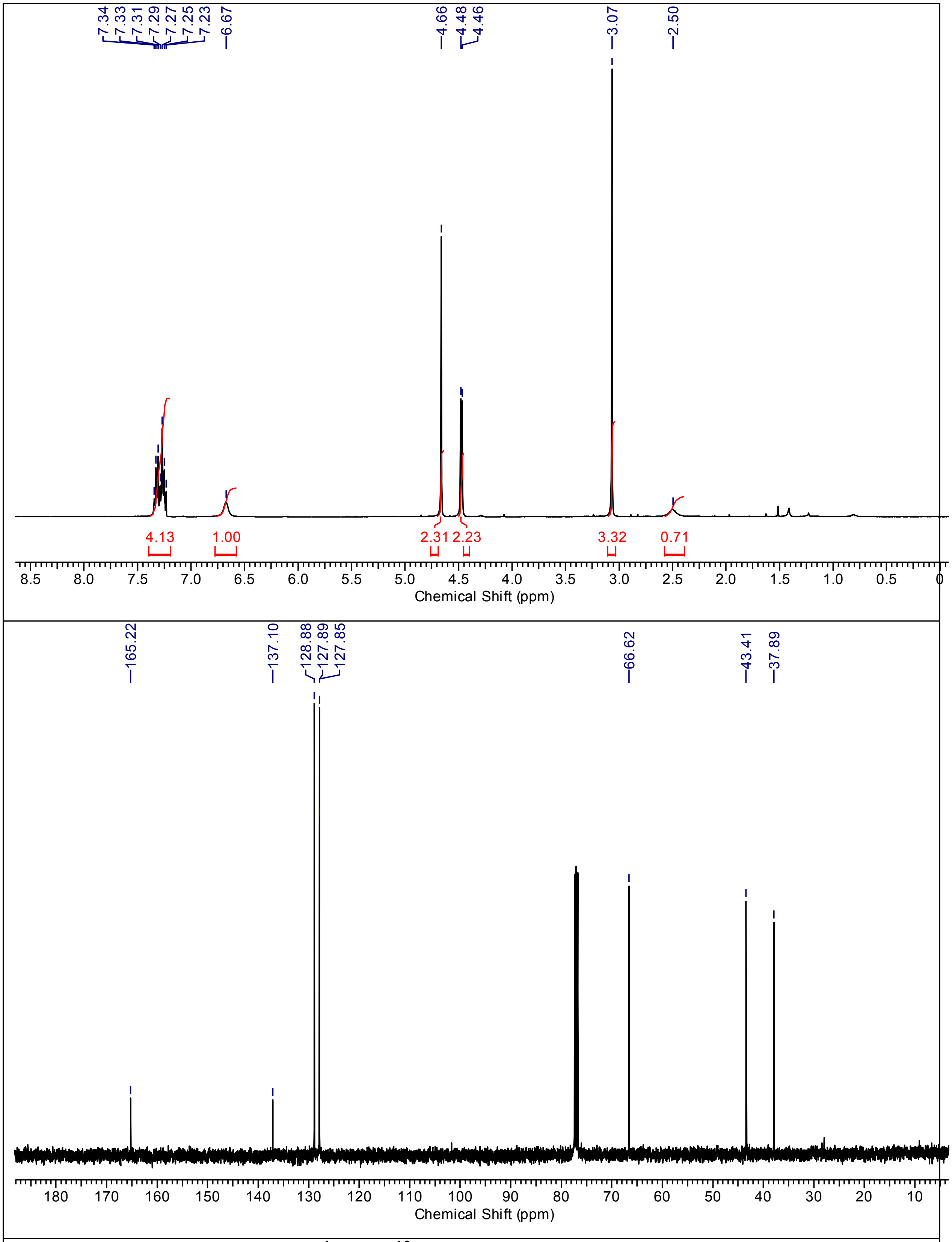

^{1}H and ^{13}C NMR spectra of **1b**

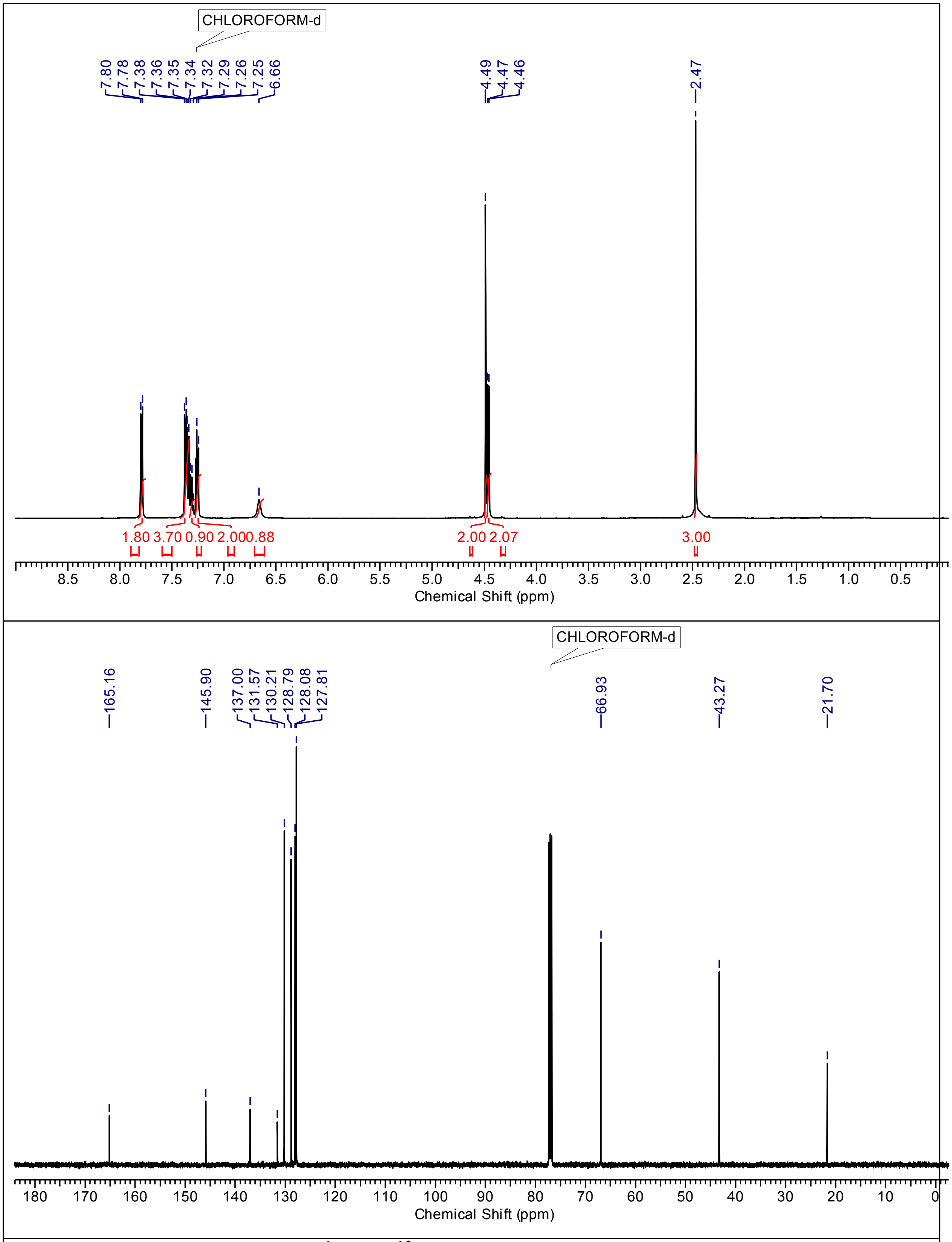

^{1}H and ^{13}C NMR spectra of **1c**

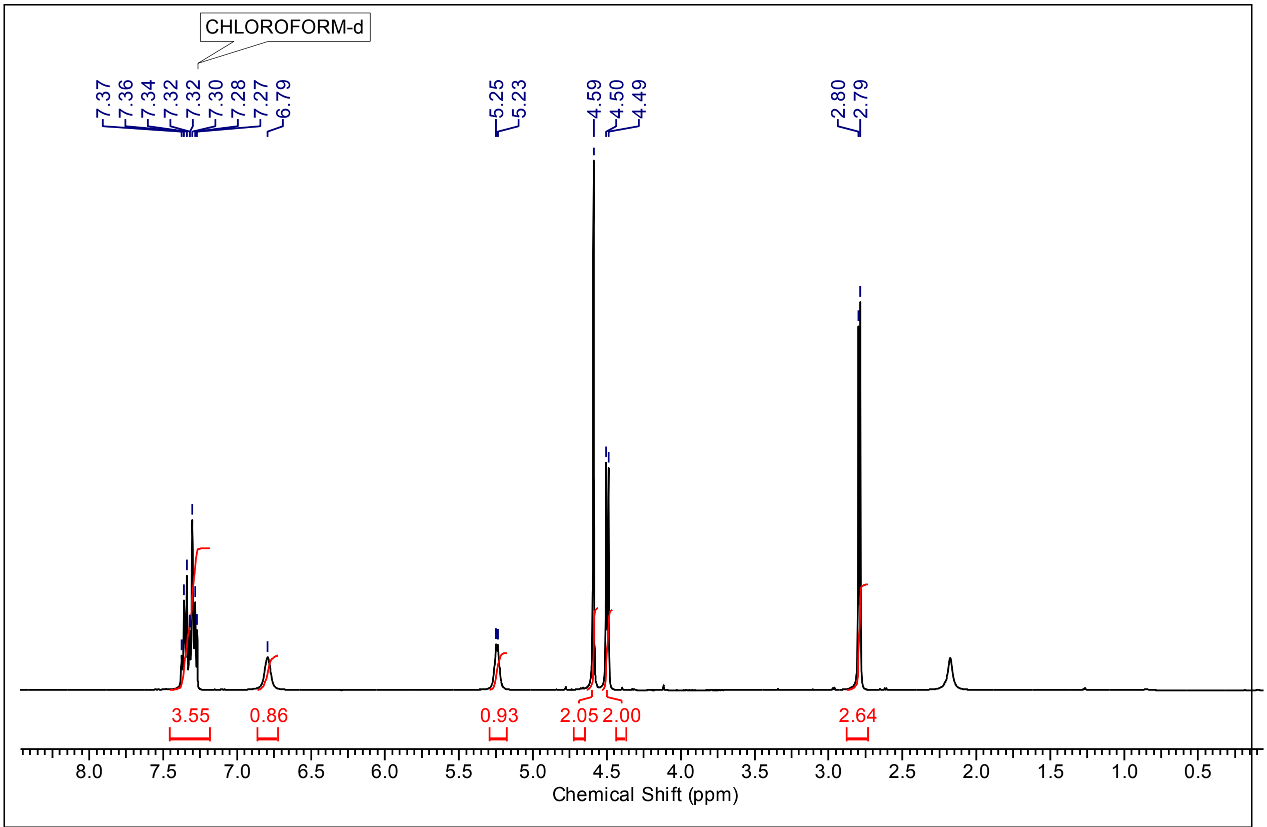

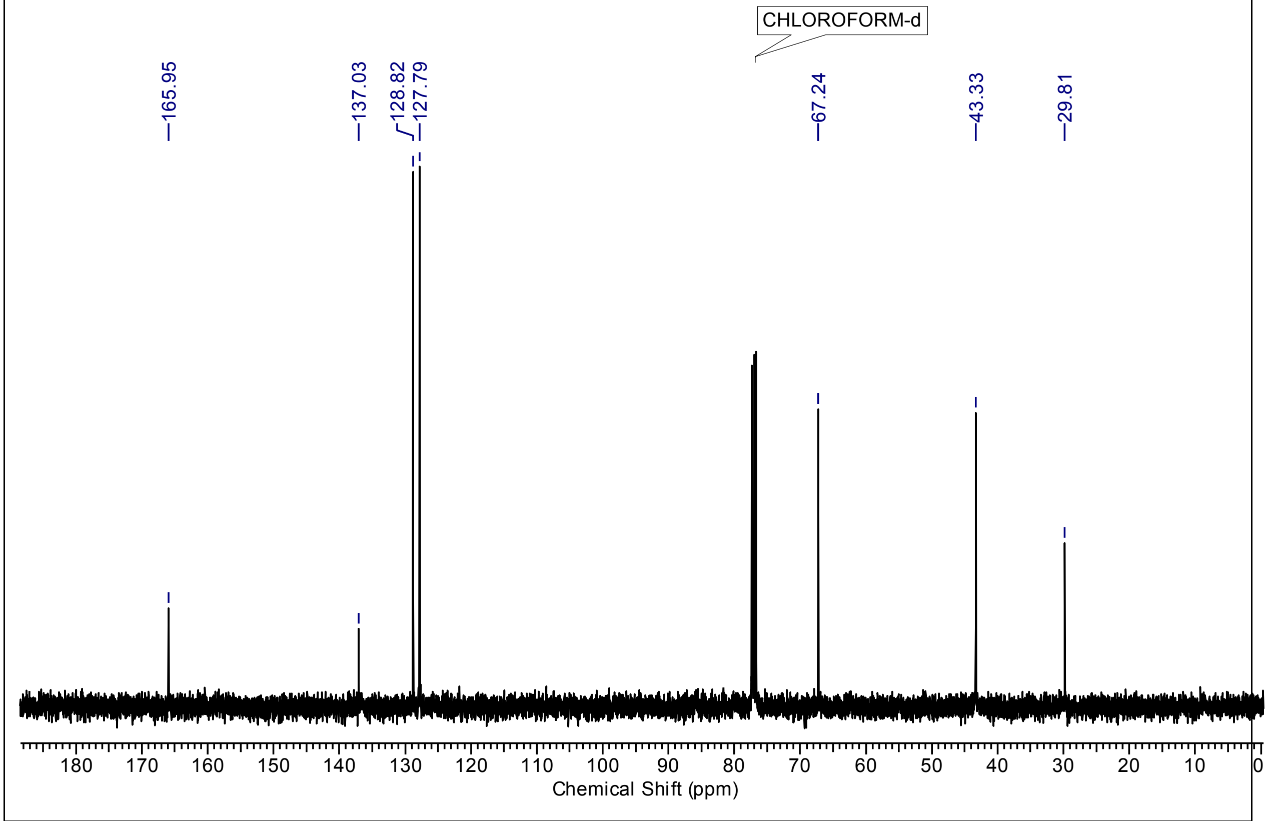

^{1}H and ^{13}C NMR spectra of **1d**

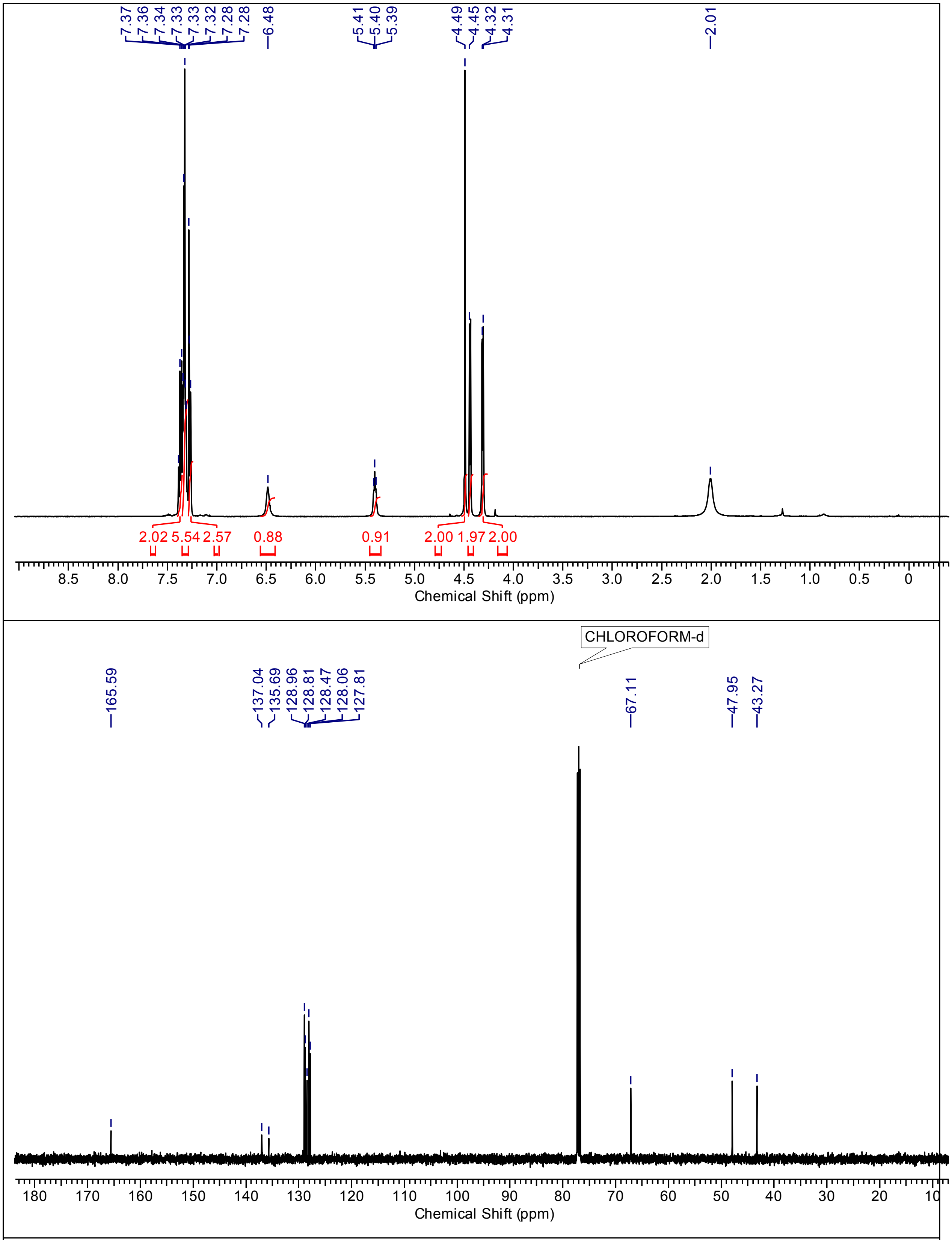

^{1}H and ^{13}C NMR spectra of **1e**

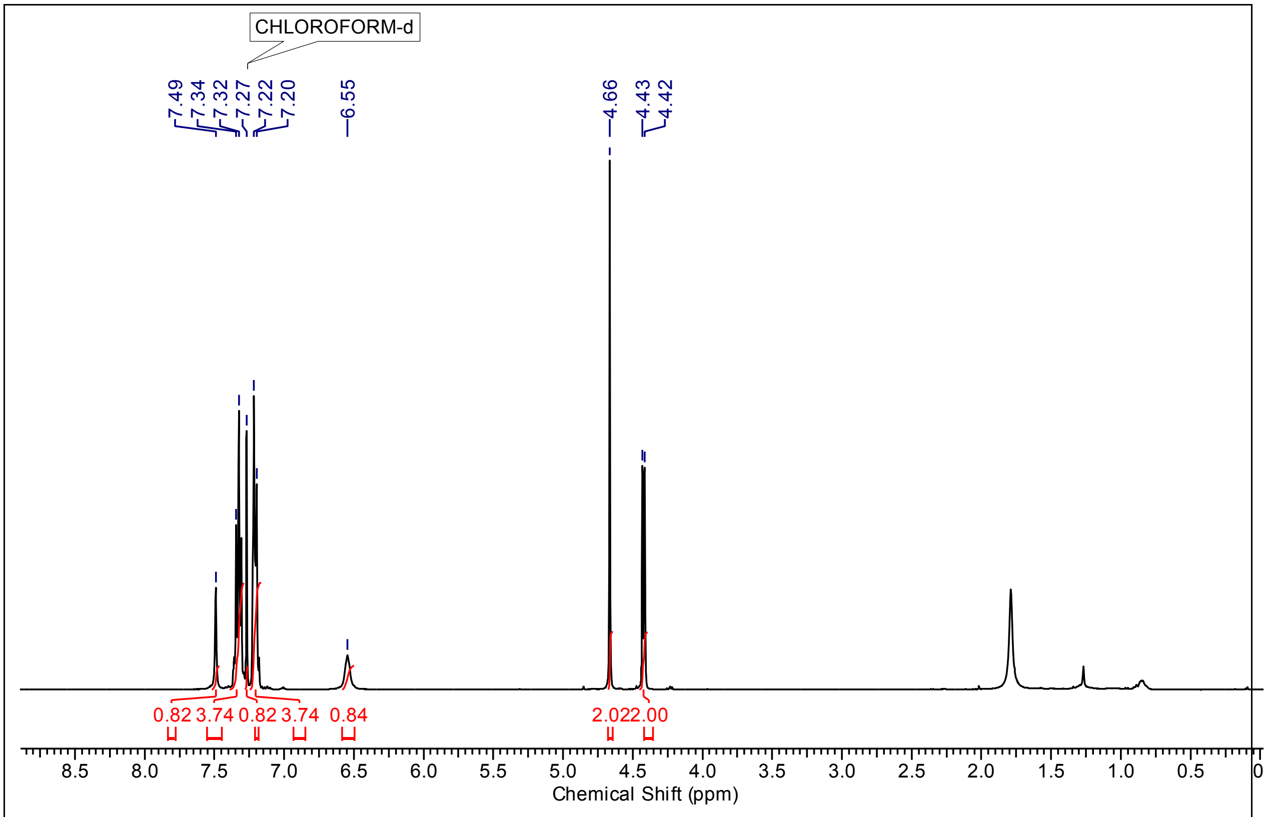

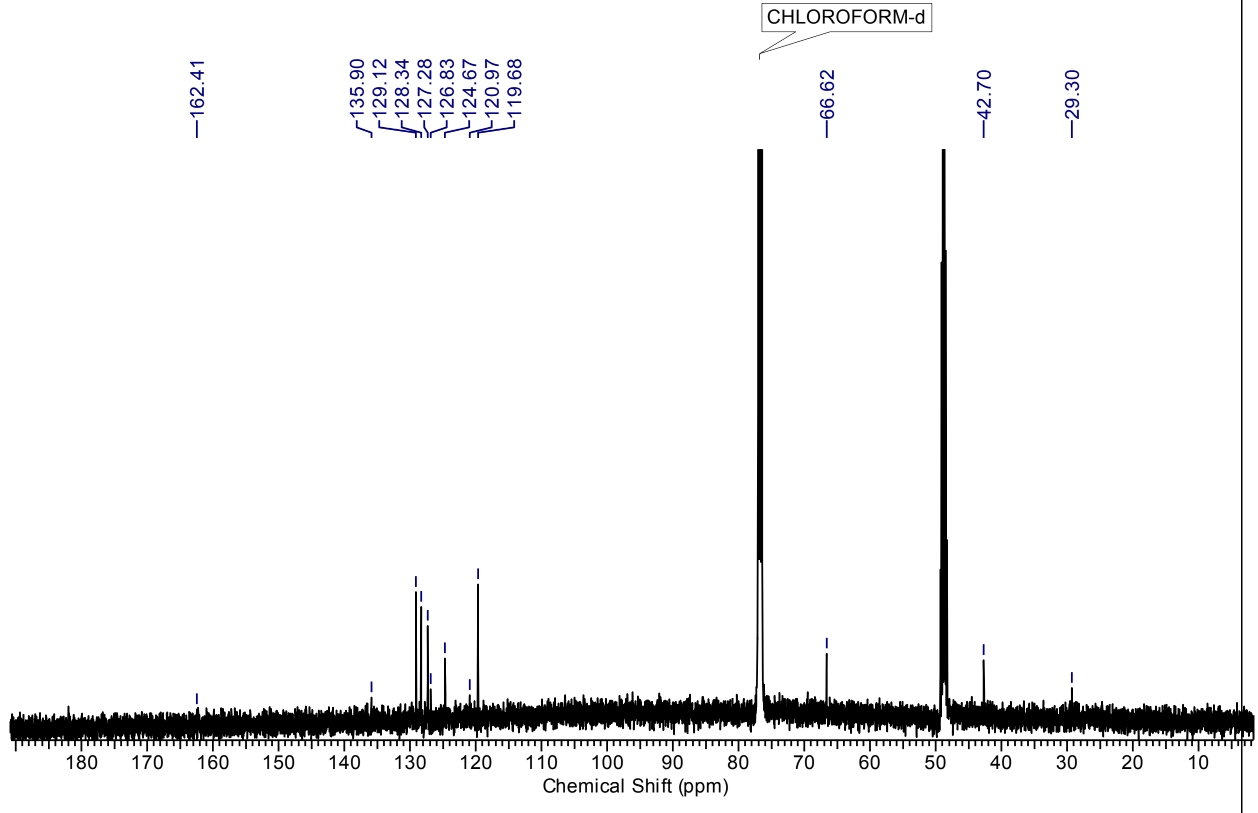

^{1}H NMR spectra of **1f**

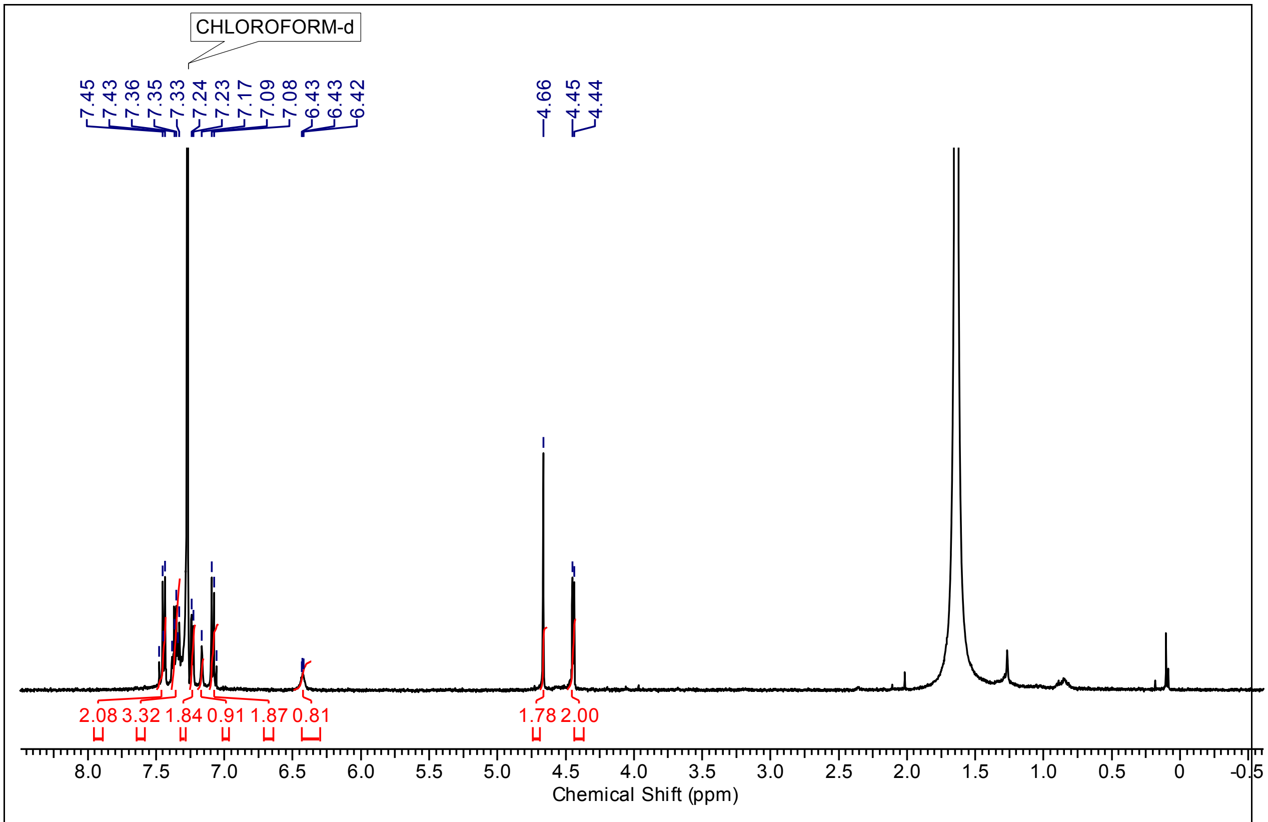

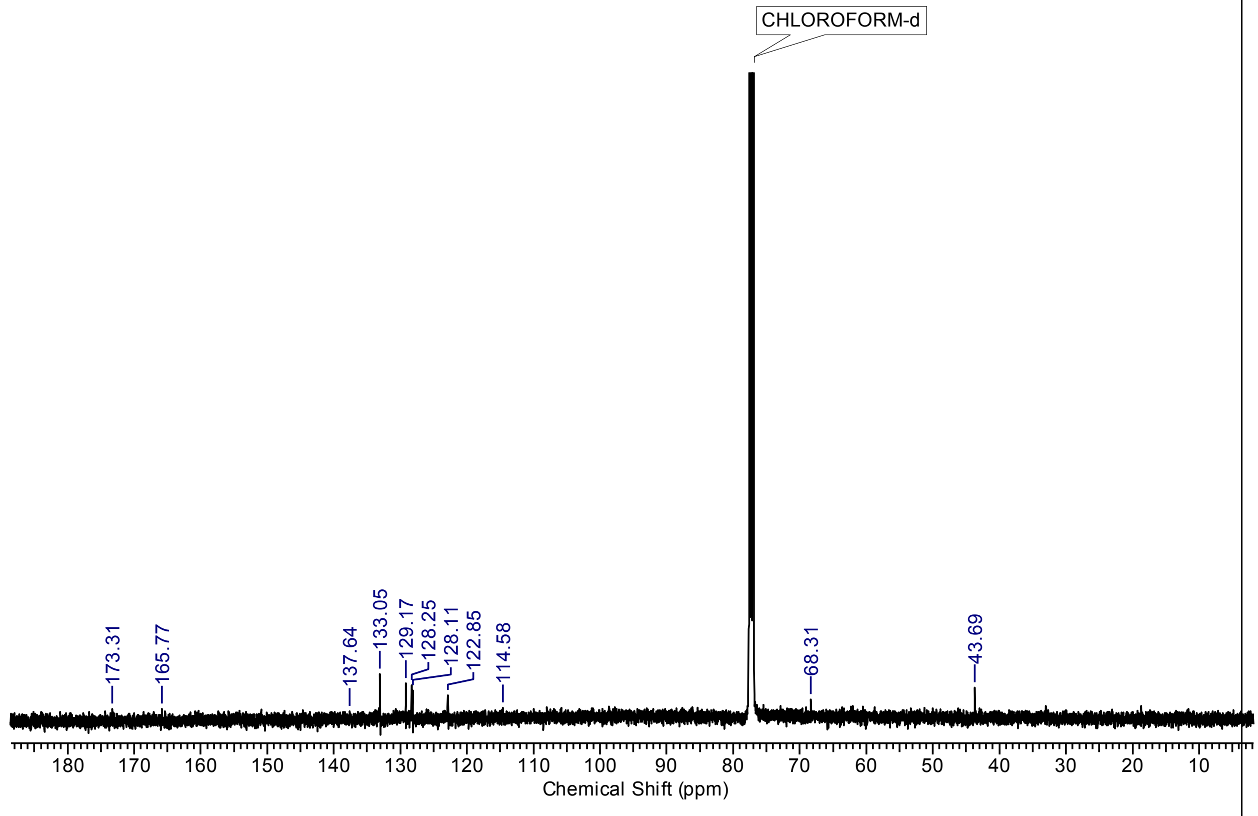

^{1}H and ^{13}C NMR spectra of **1g**

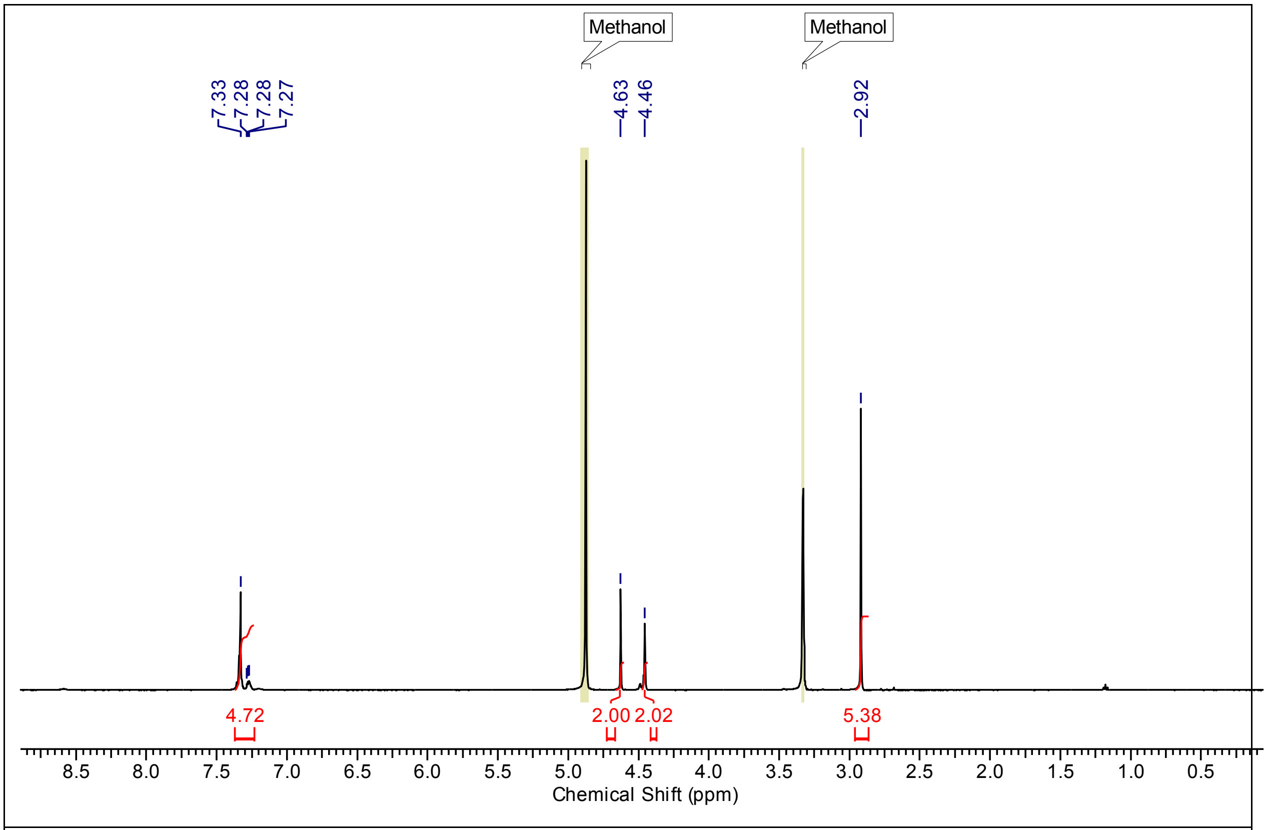

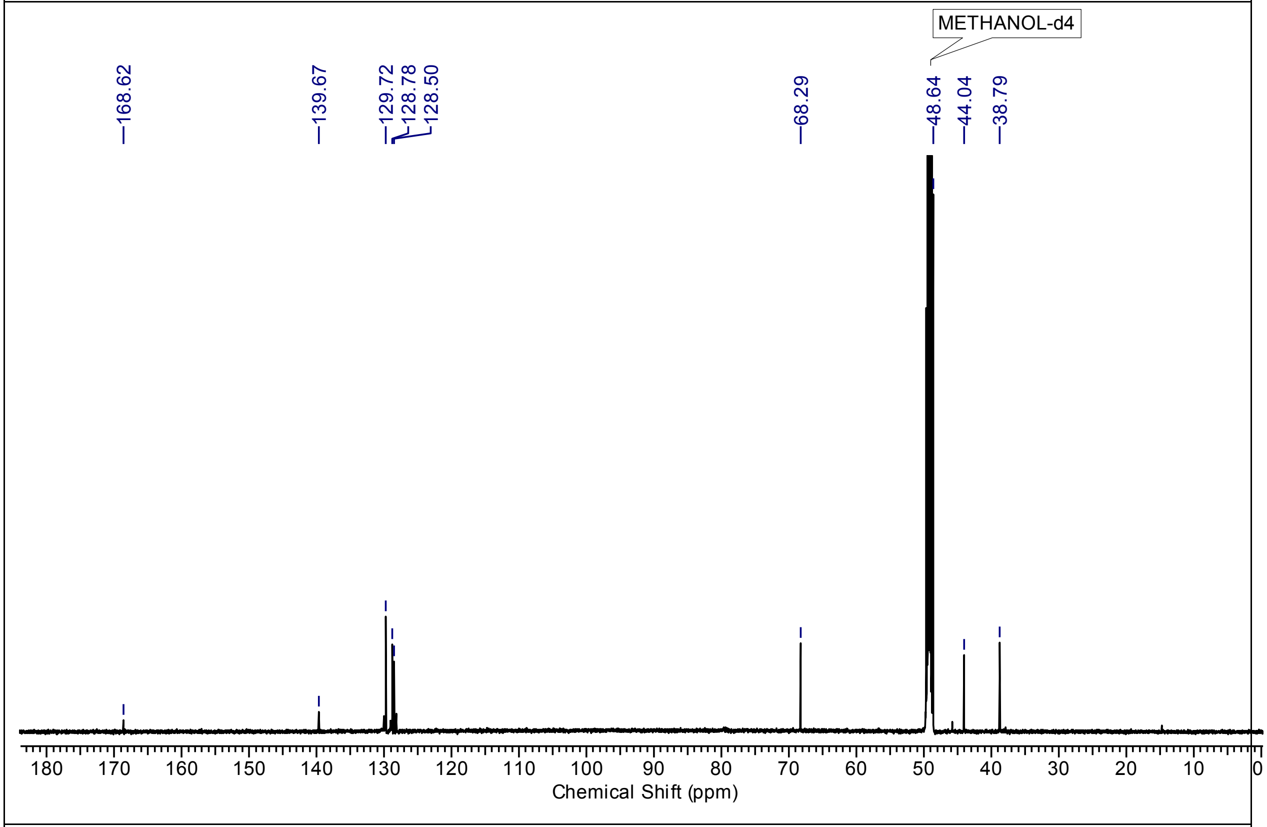

^{1}H and ^{13}C NMR spectra of **1h**

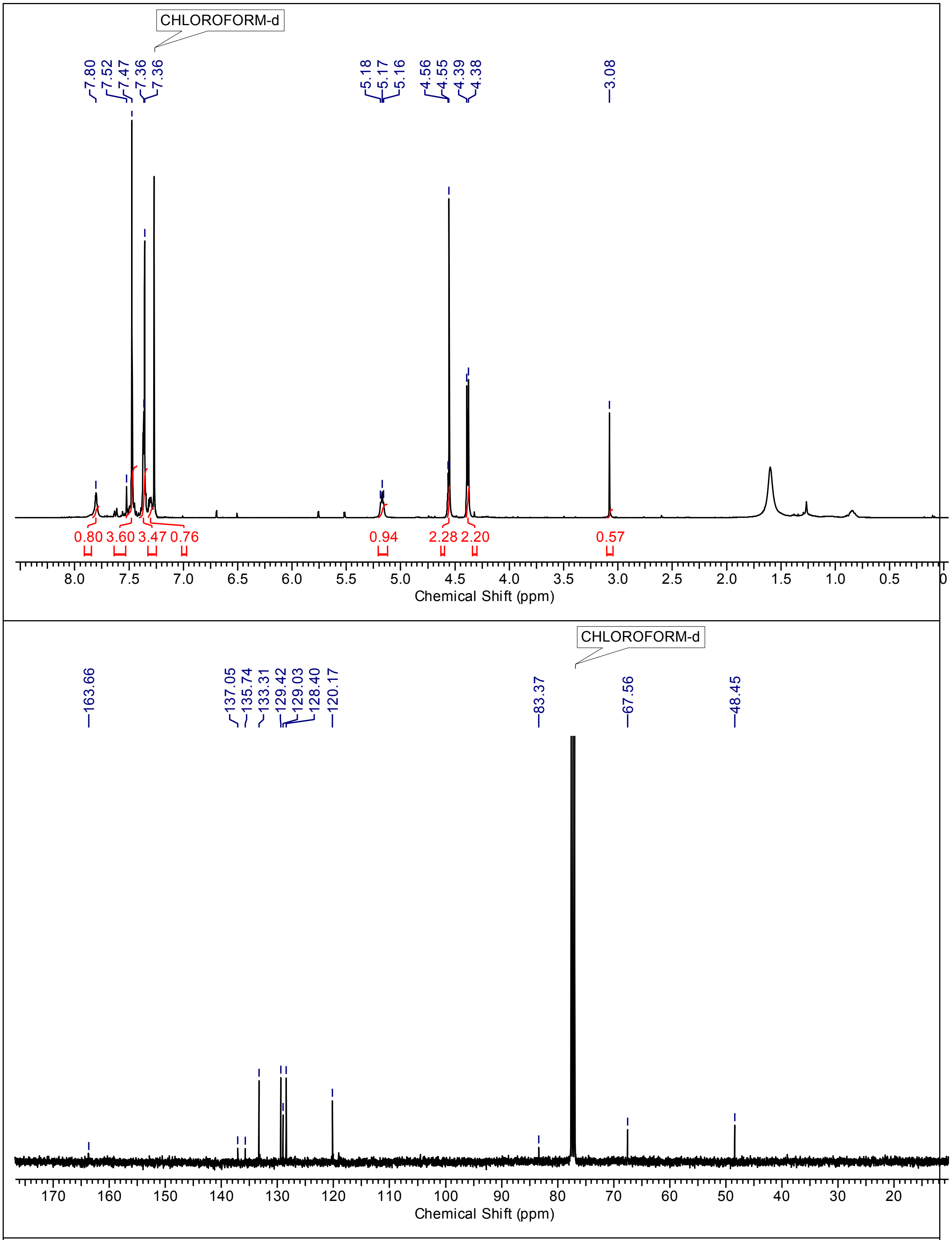

^{1}H and ^{13}C NMR spectra of **2a**

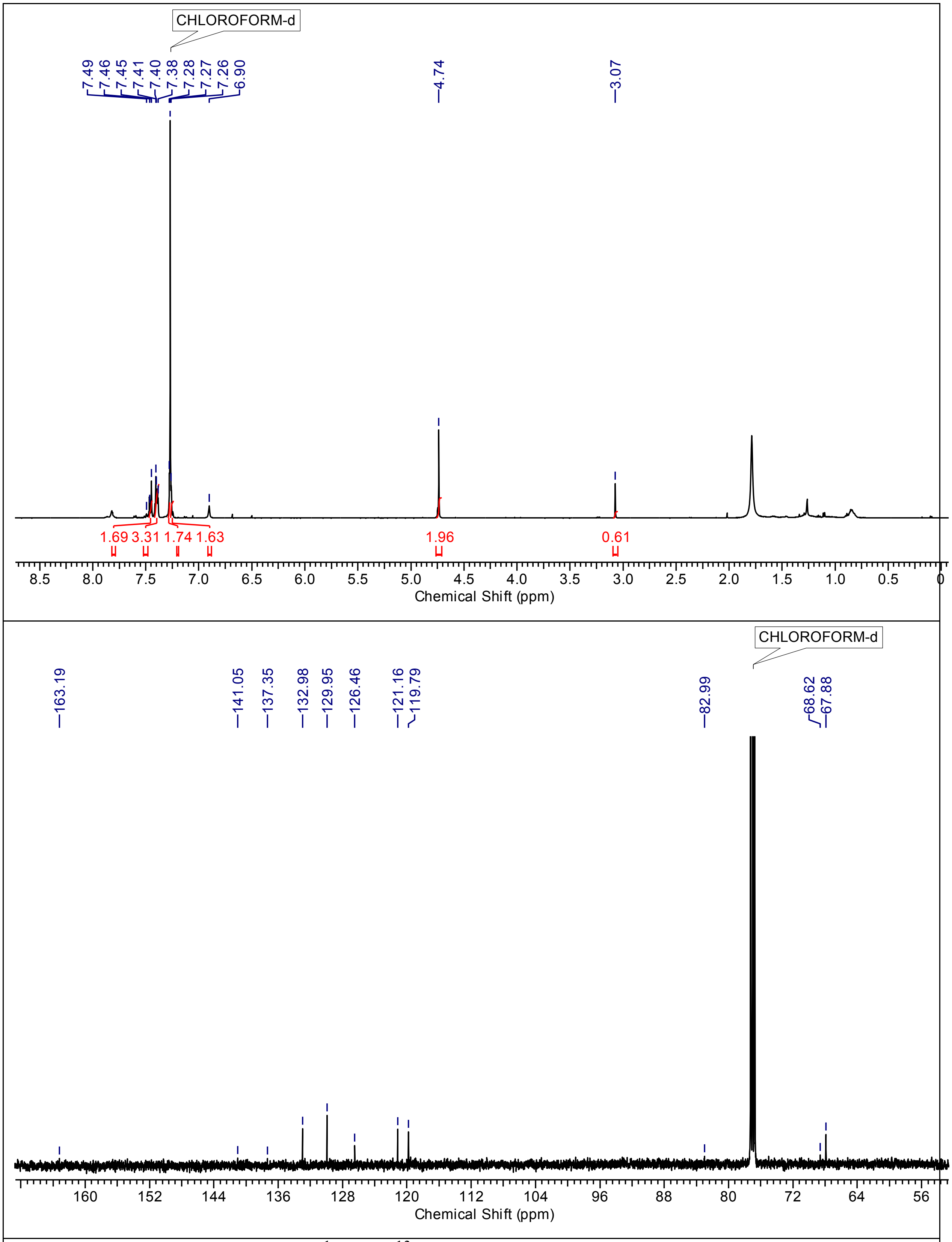

^{1}H and ^{13}C NMR spectra of **2b**

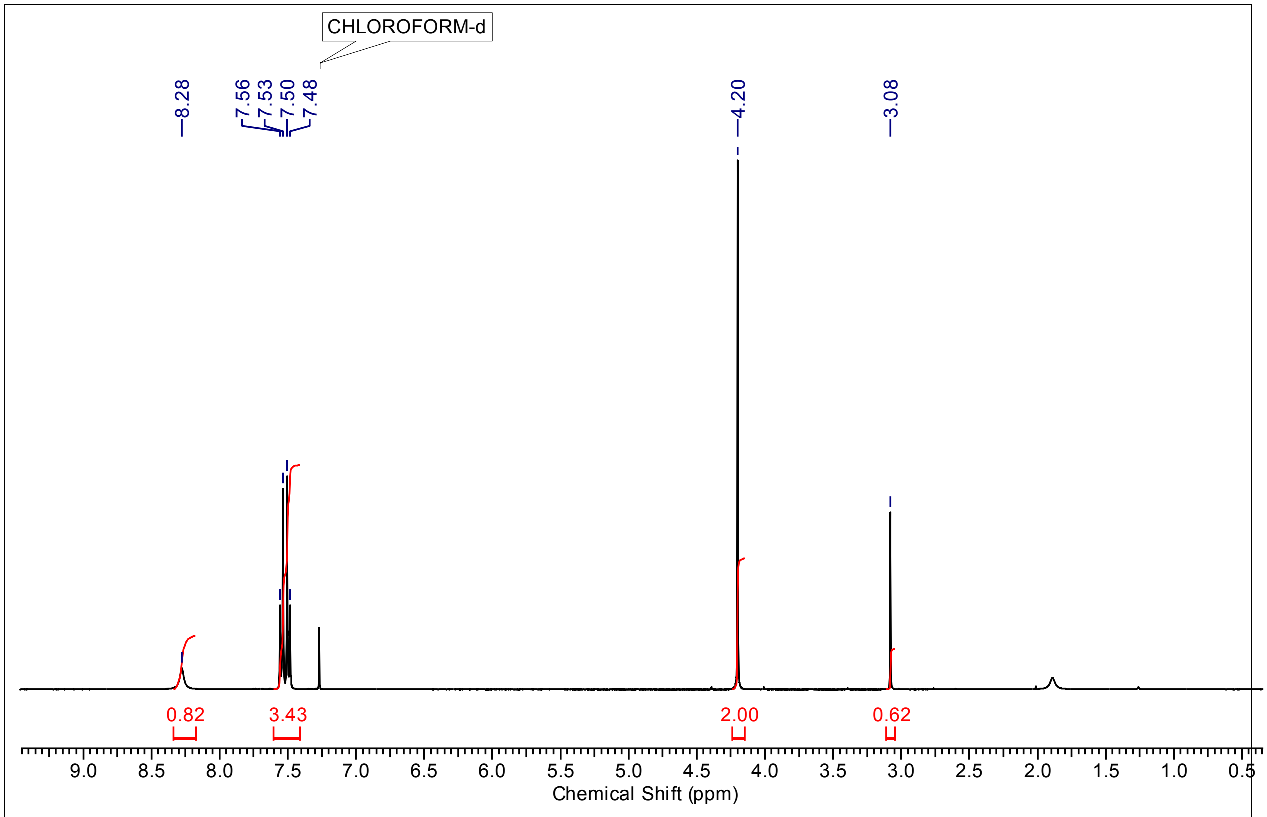

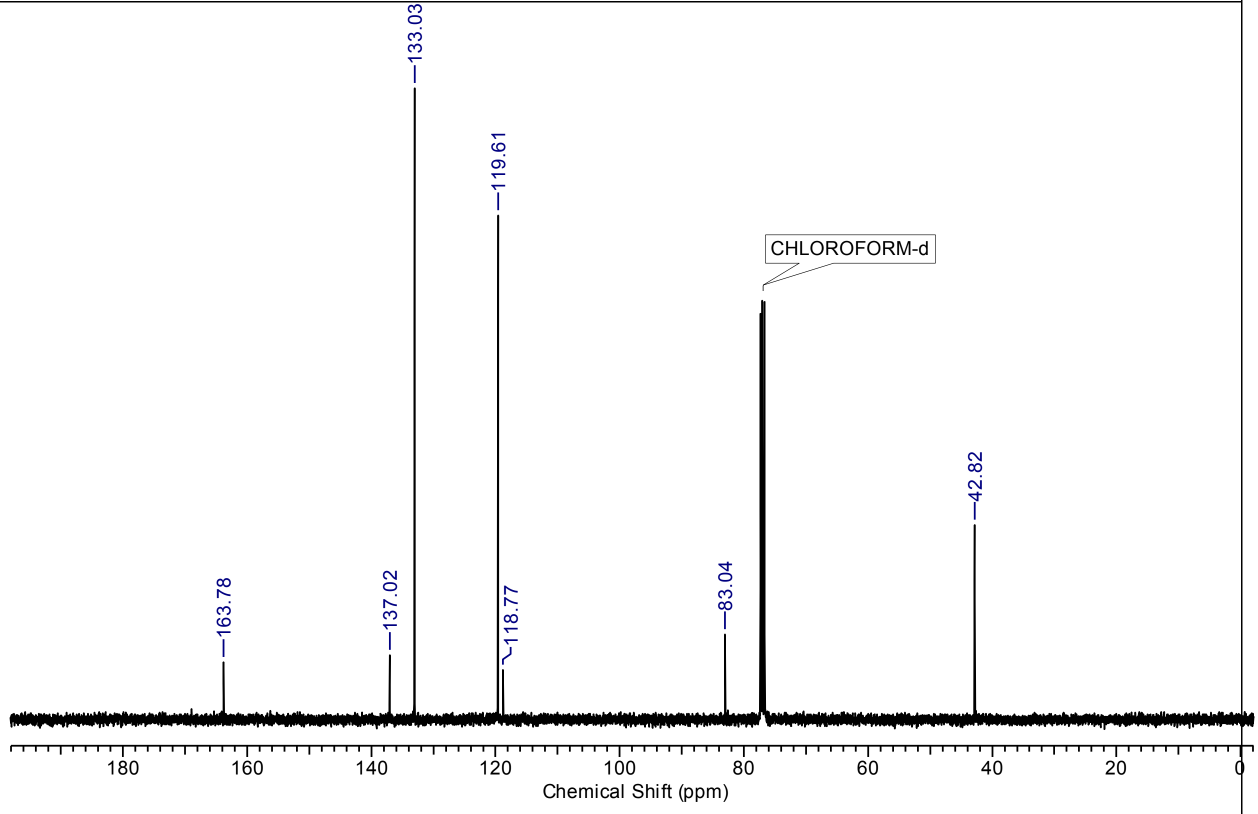

^{1}H and ^{13}C NMR spectra of **2c**

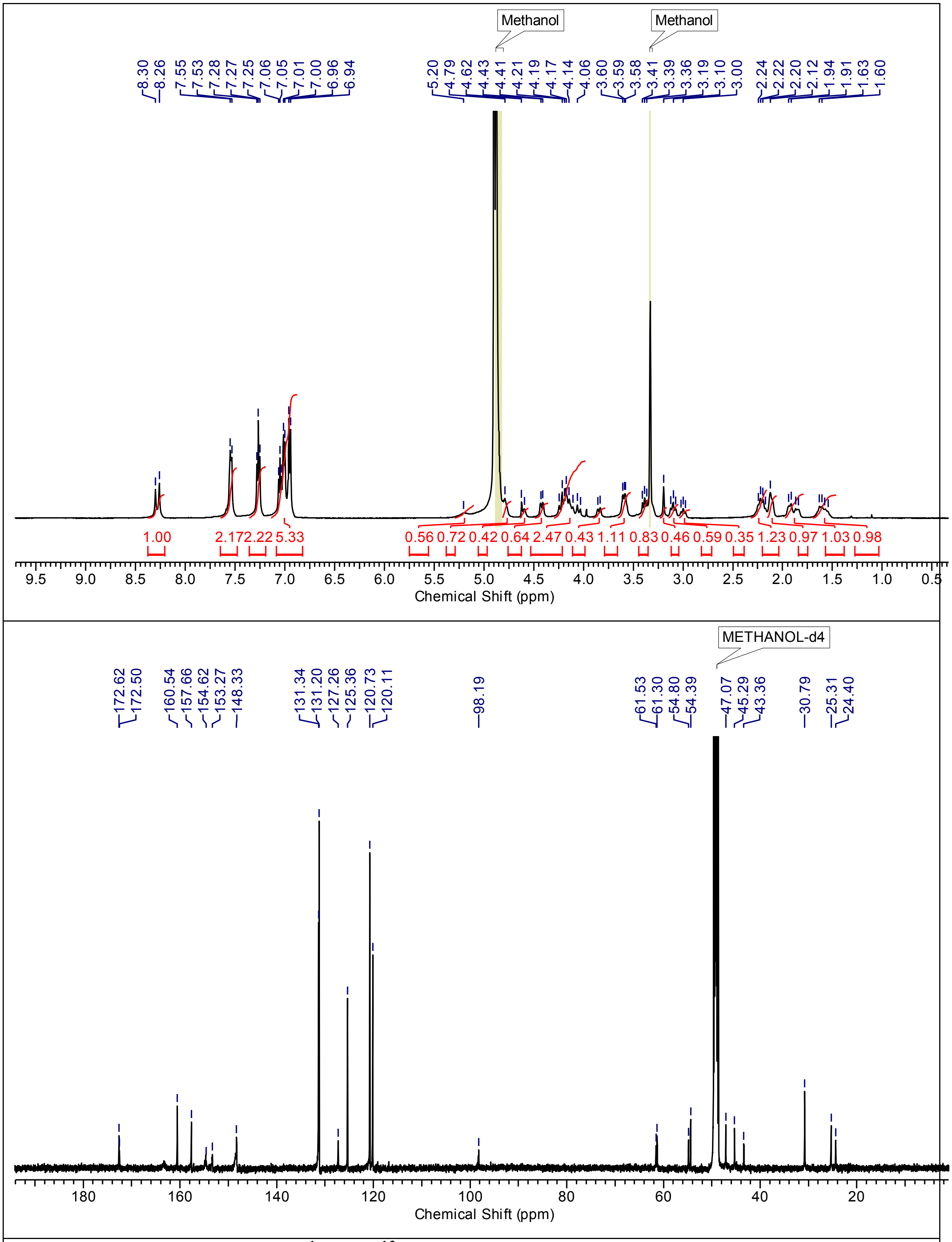

^{1}H and ^{13}C NMR spectra of **pre-3**

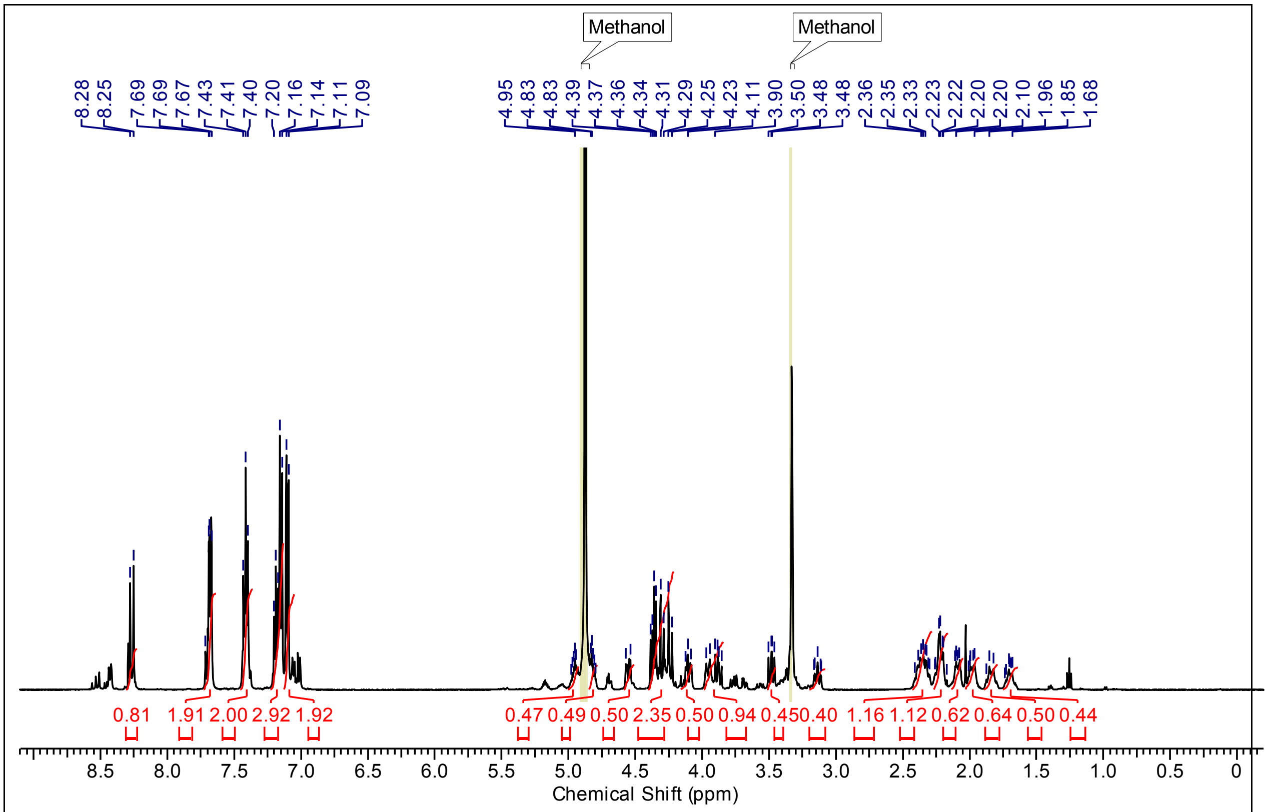

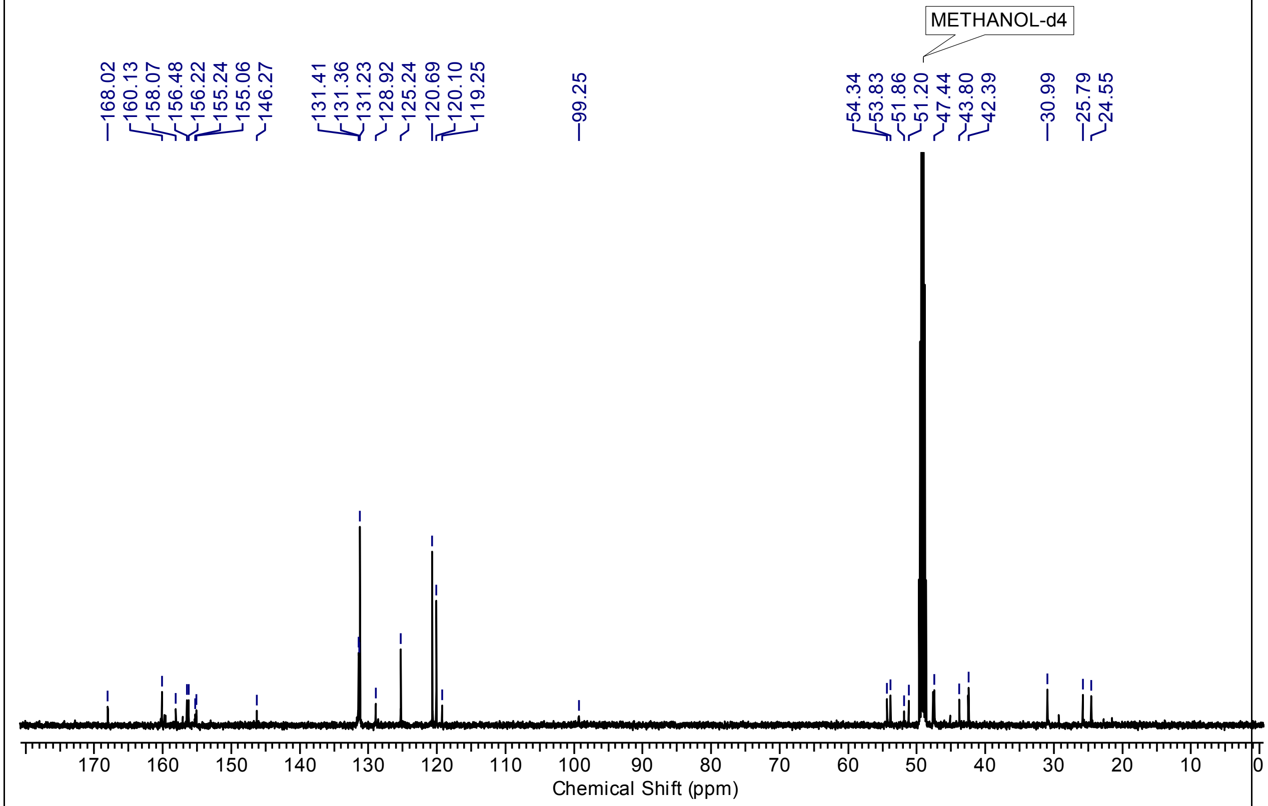

^{1}H and ^{13}C NMR spectra of **3a**

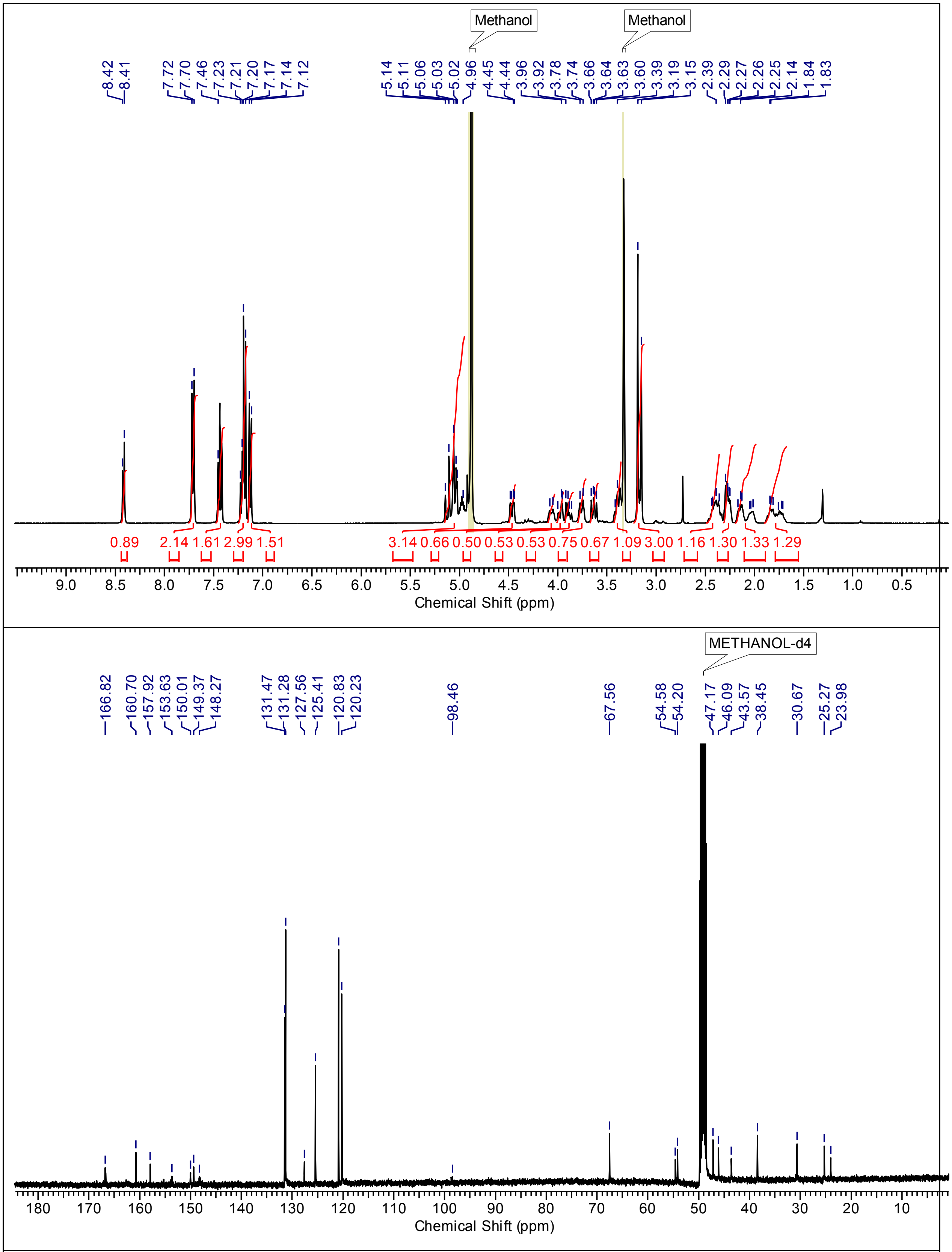

^{1}H and ^{13}C NMR spectra of **3b**

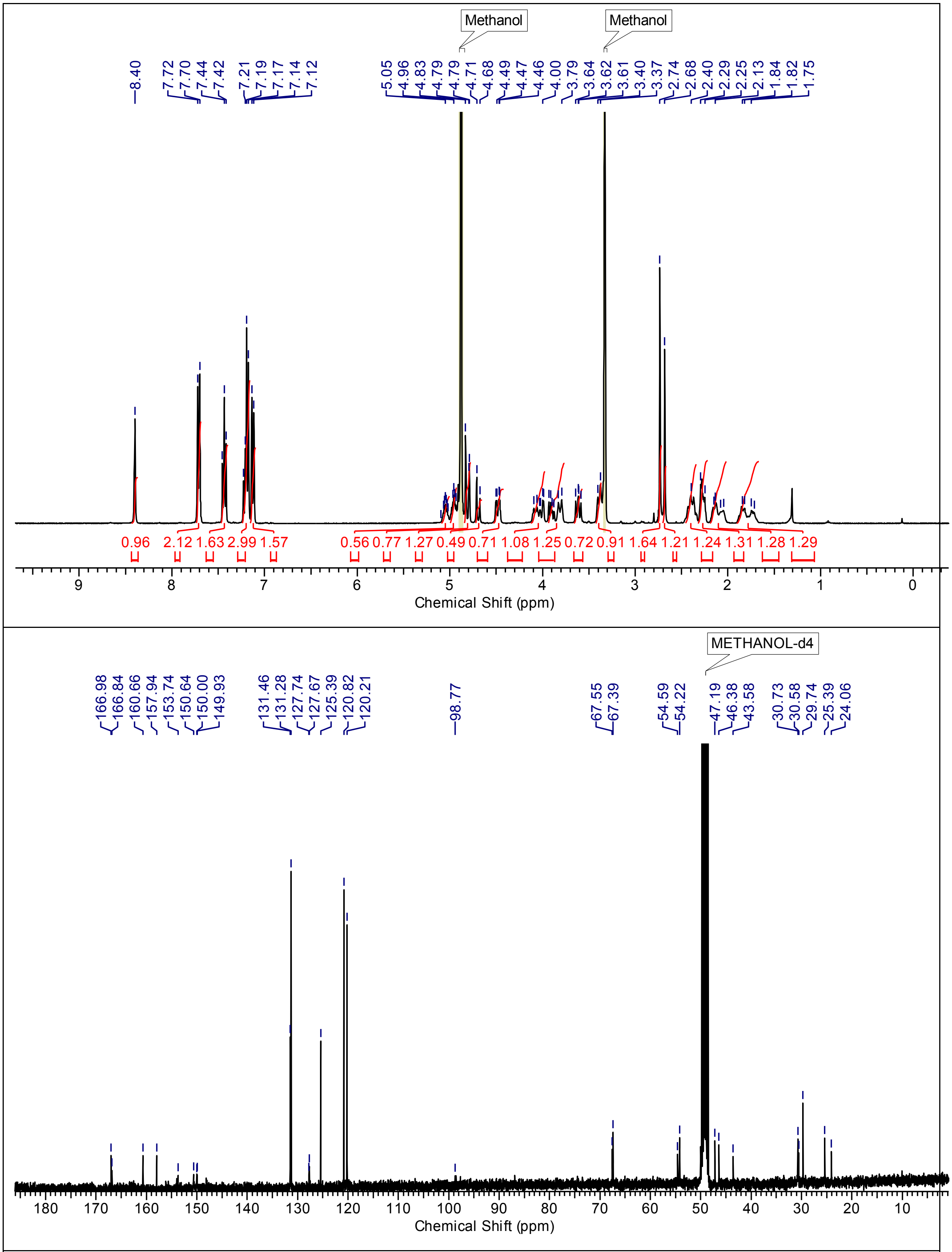

^{1}H and ^{13}C NMR spectra of **3c**

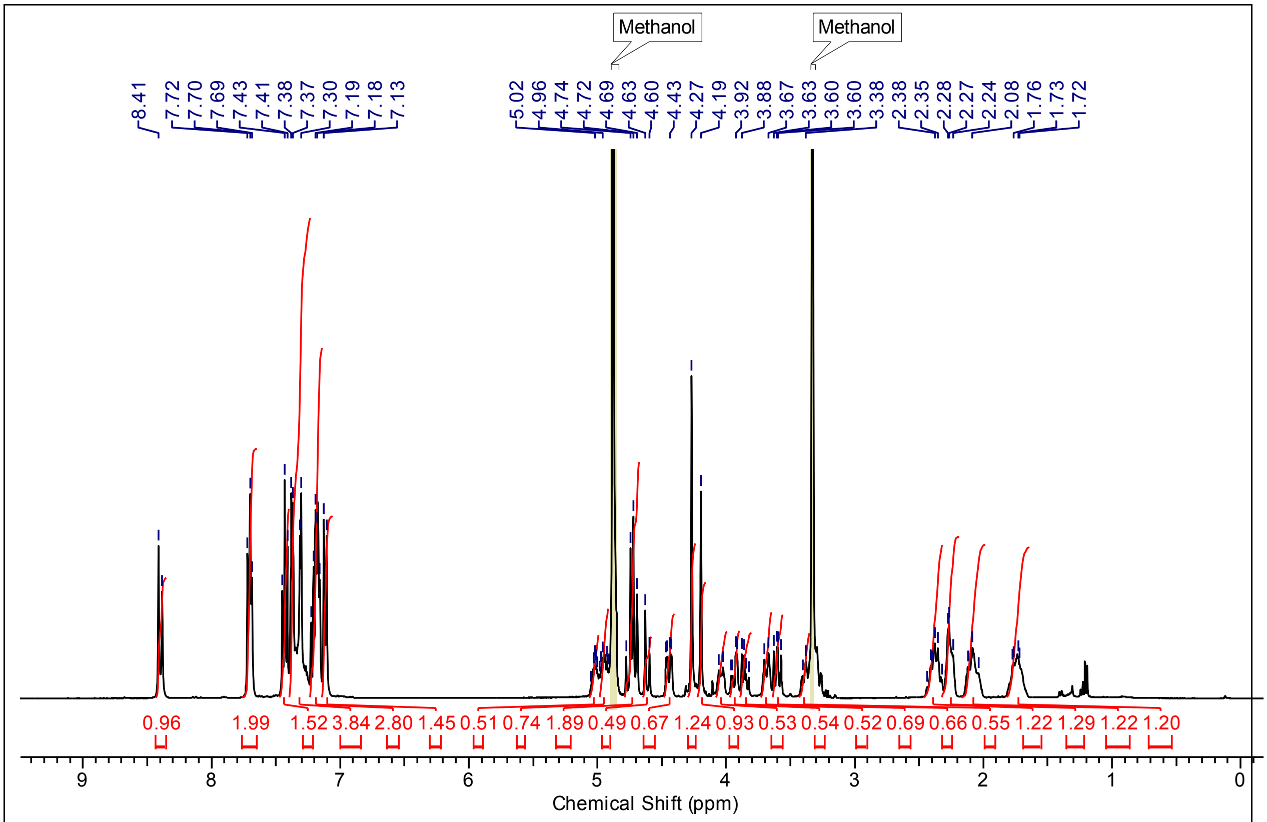

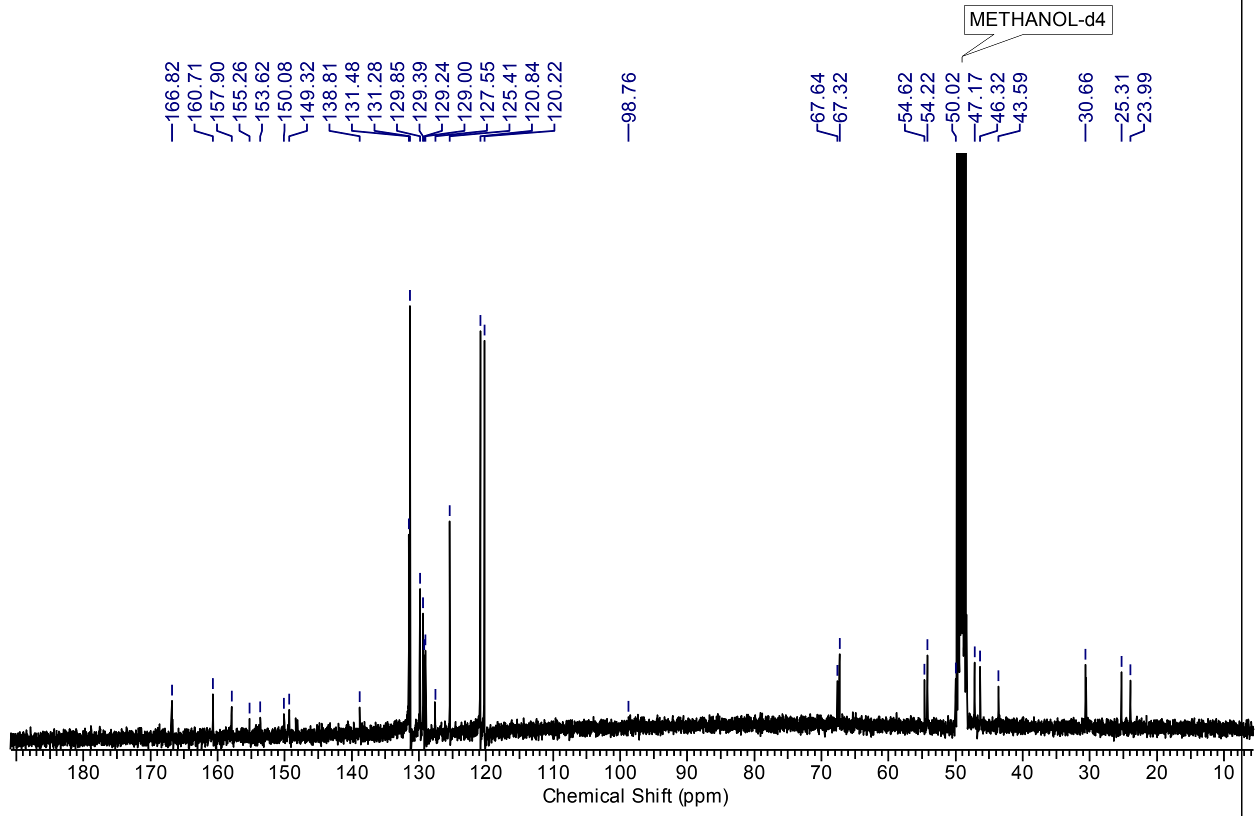

^{1}H and ^{13}C NMR spectra of **3d**

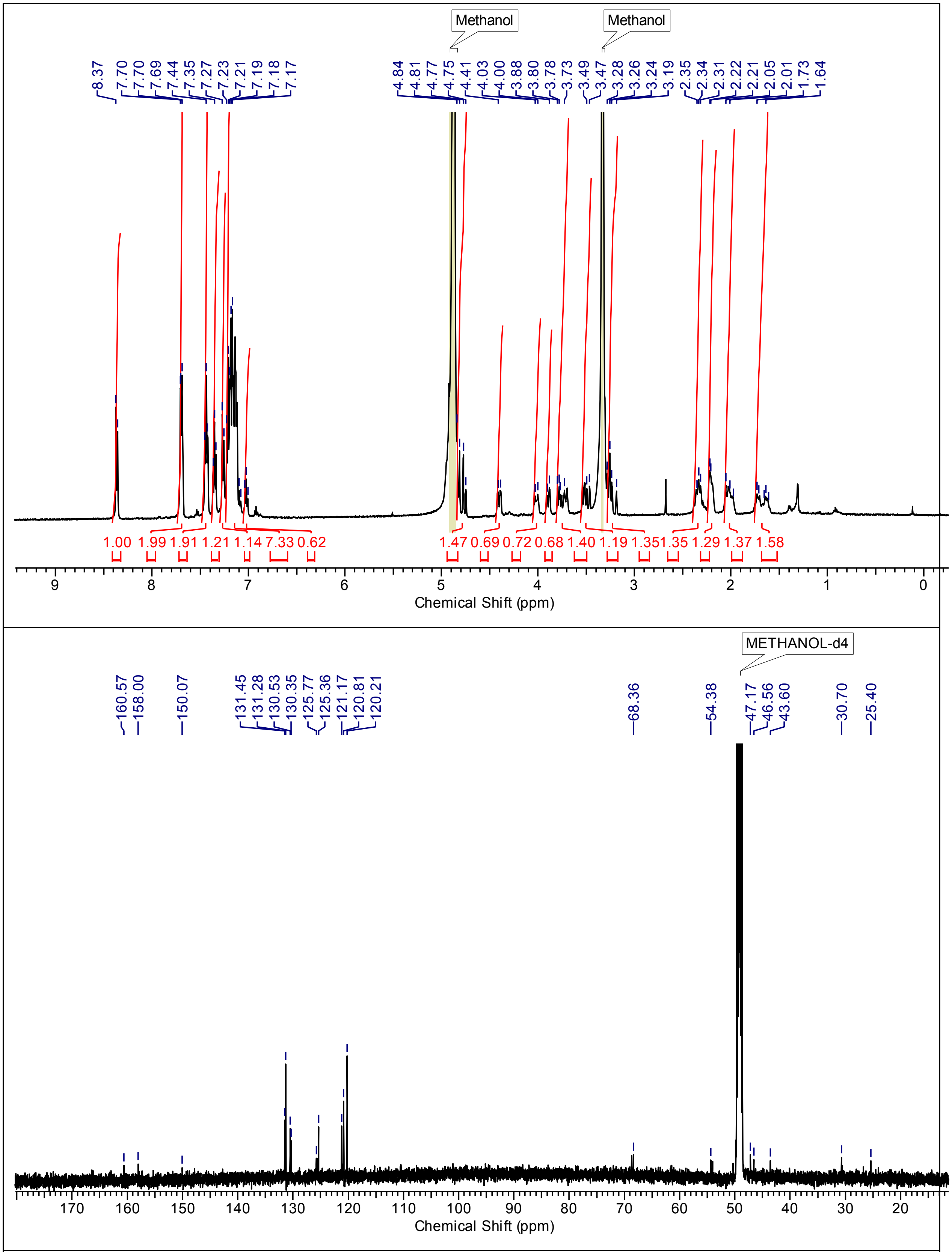

^{1}H and ^{13}C NMR spectra of **3e**

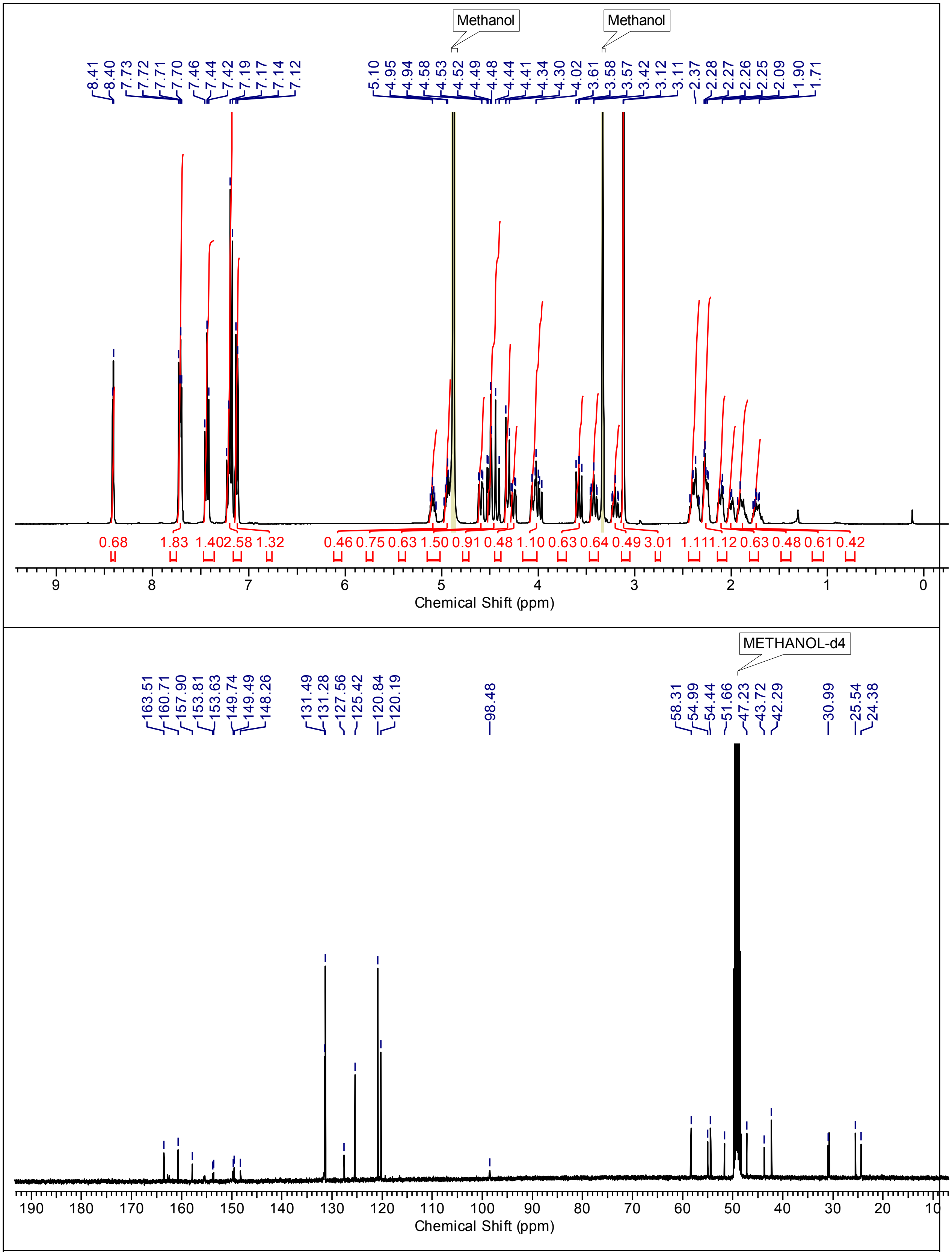

^{1}H and ^{13}C NMR spectra of **3f**

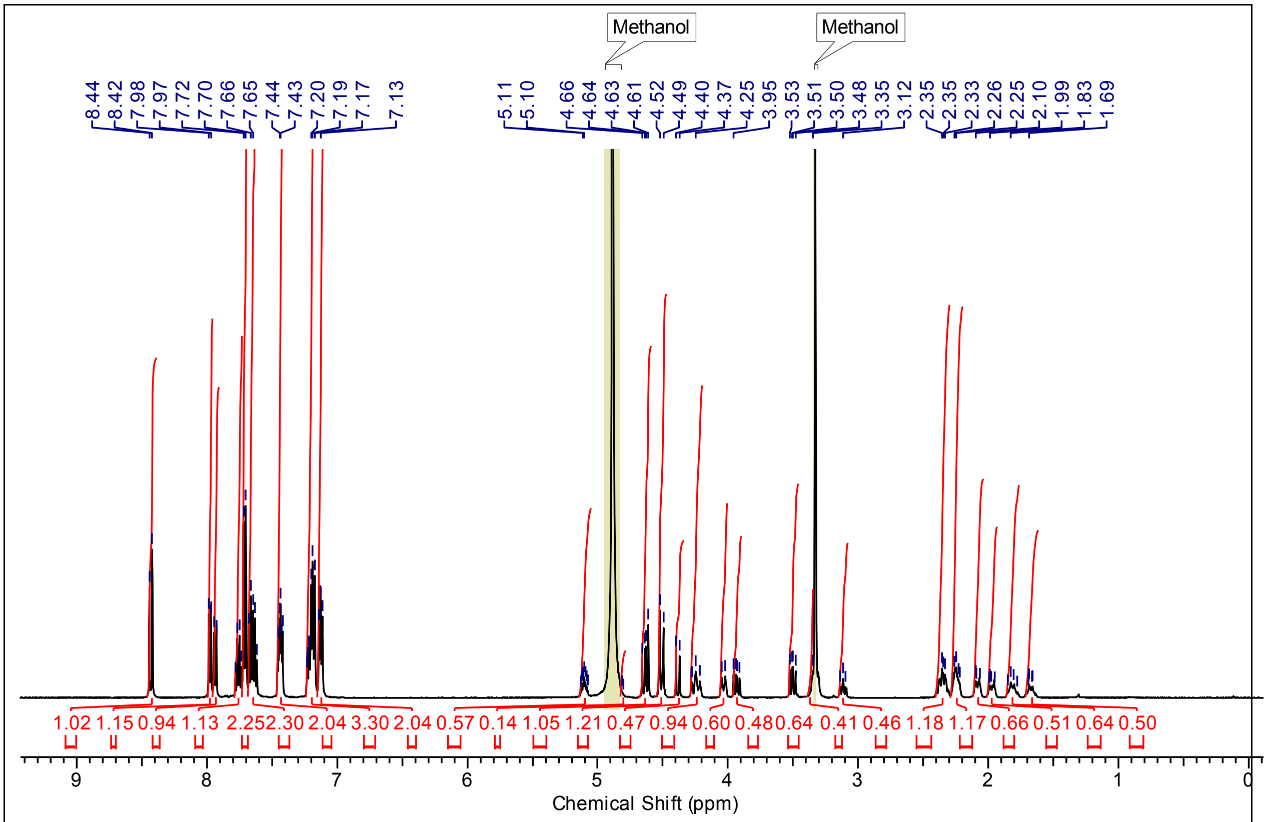

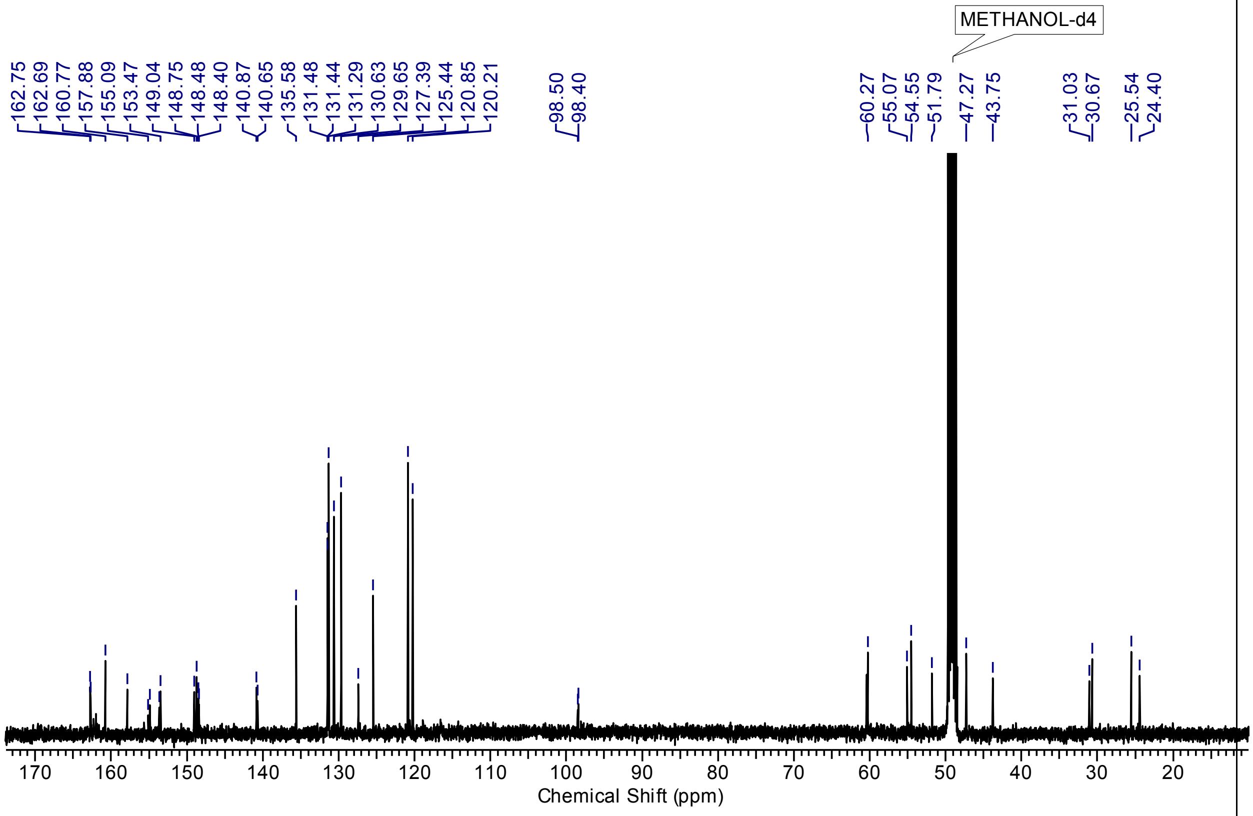

^{1}H and ^{13}C NMR spectra of **3g**

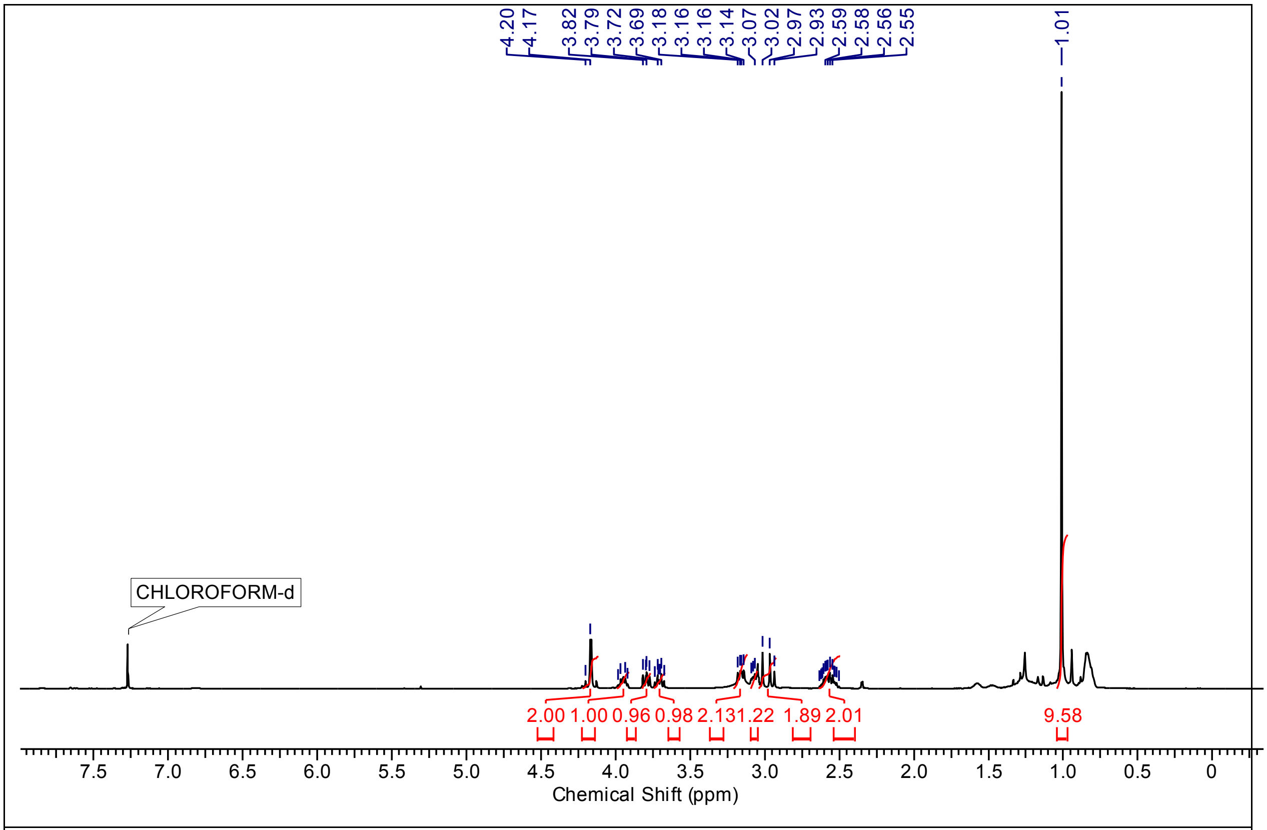

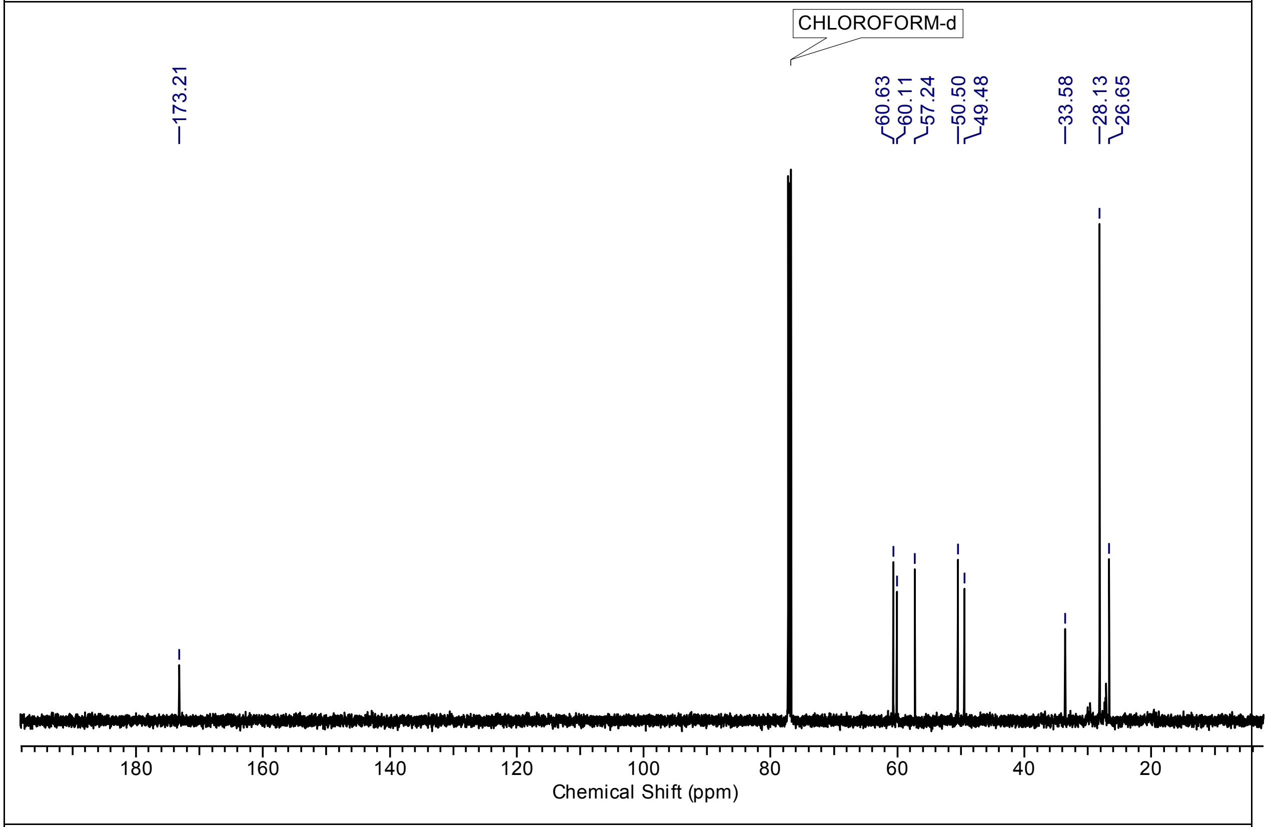

^{1}H and ^{13}C NMR spectra of **pre-4**

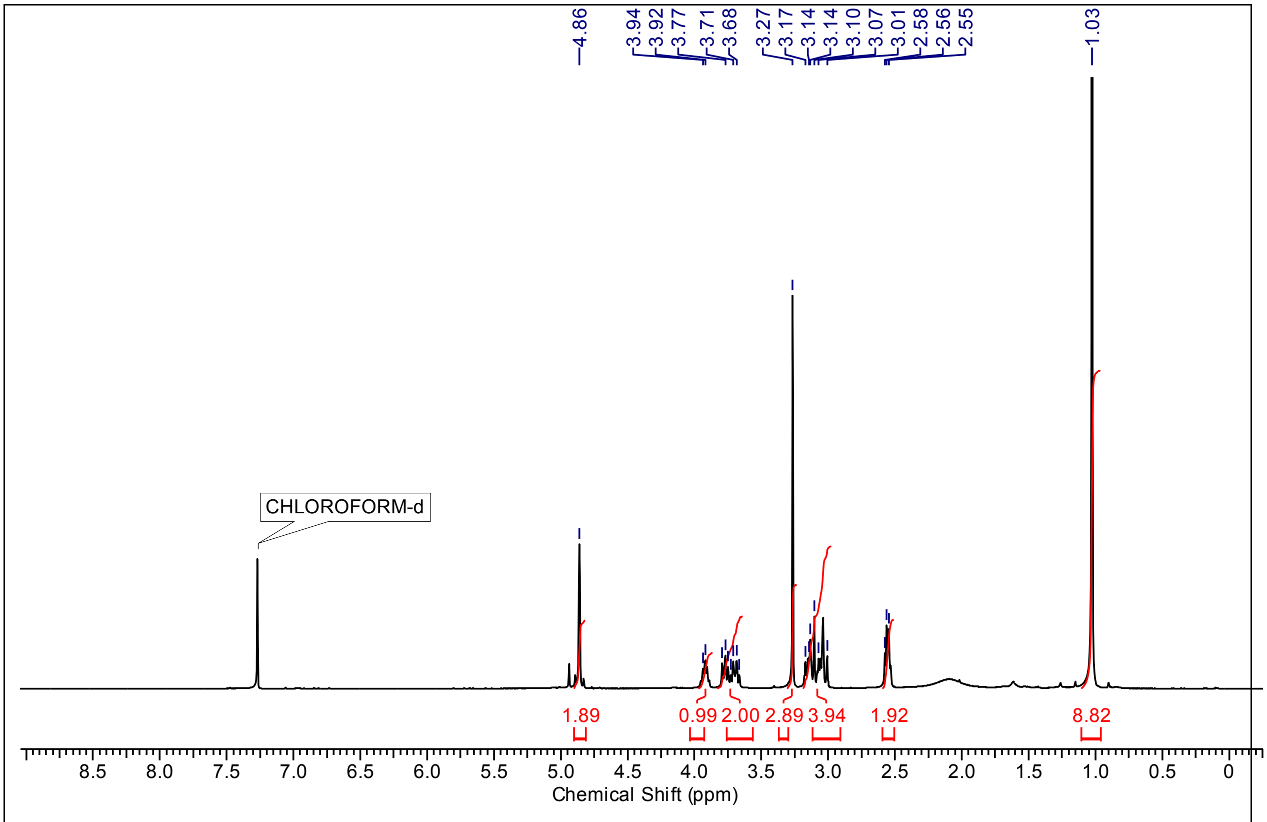

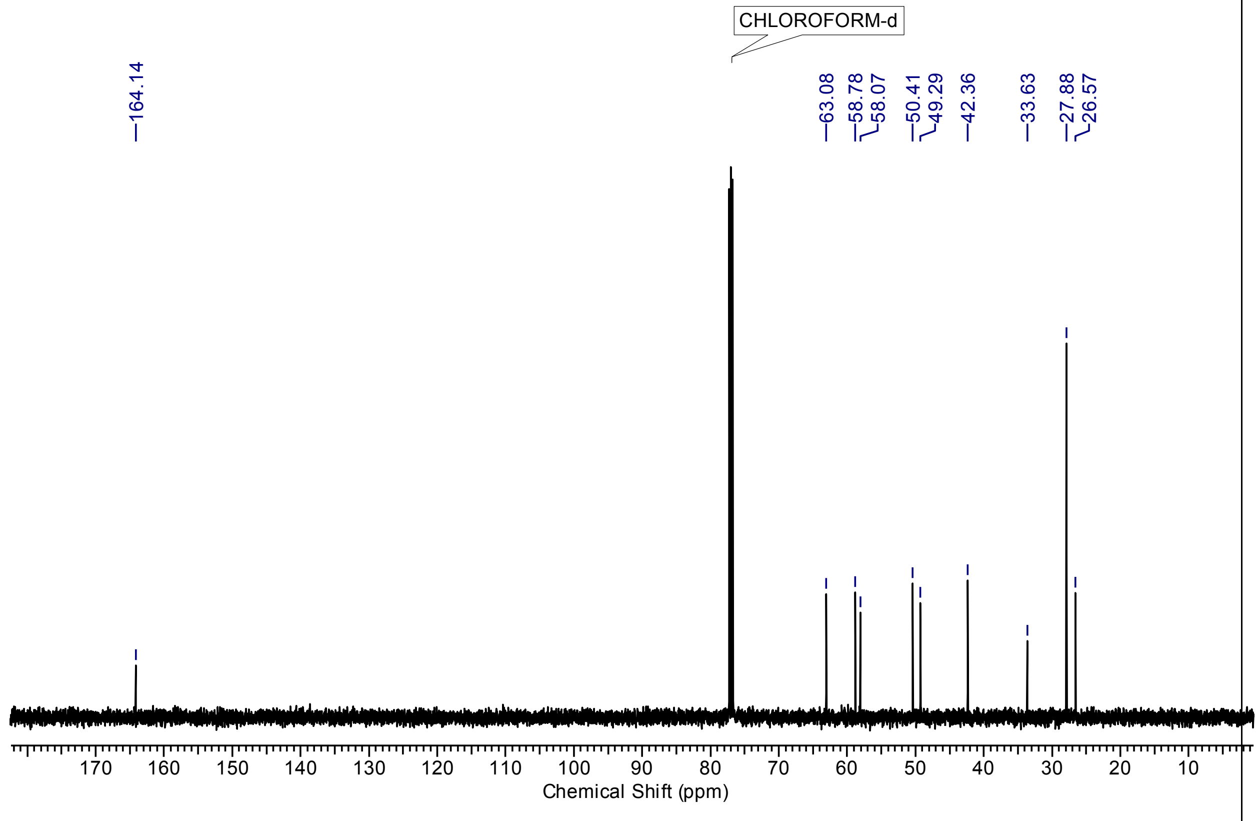

^{1}H spectra of **4a**

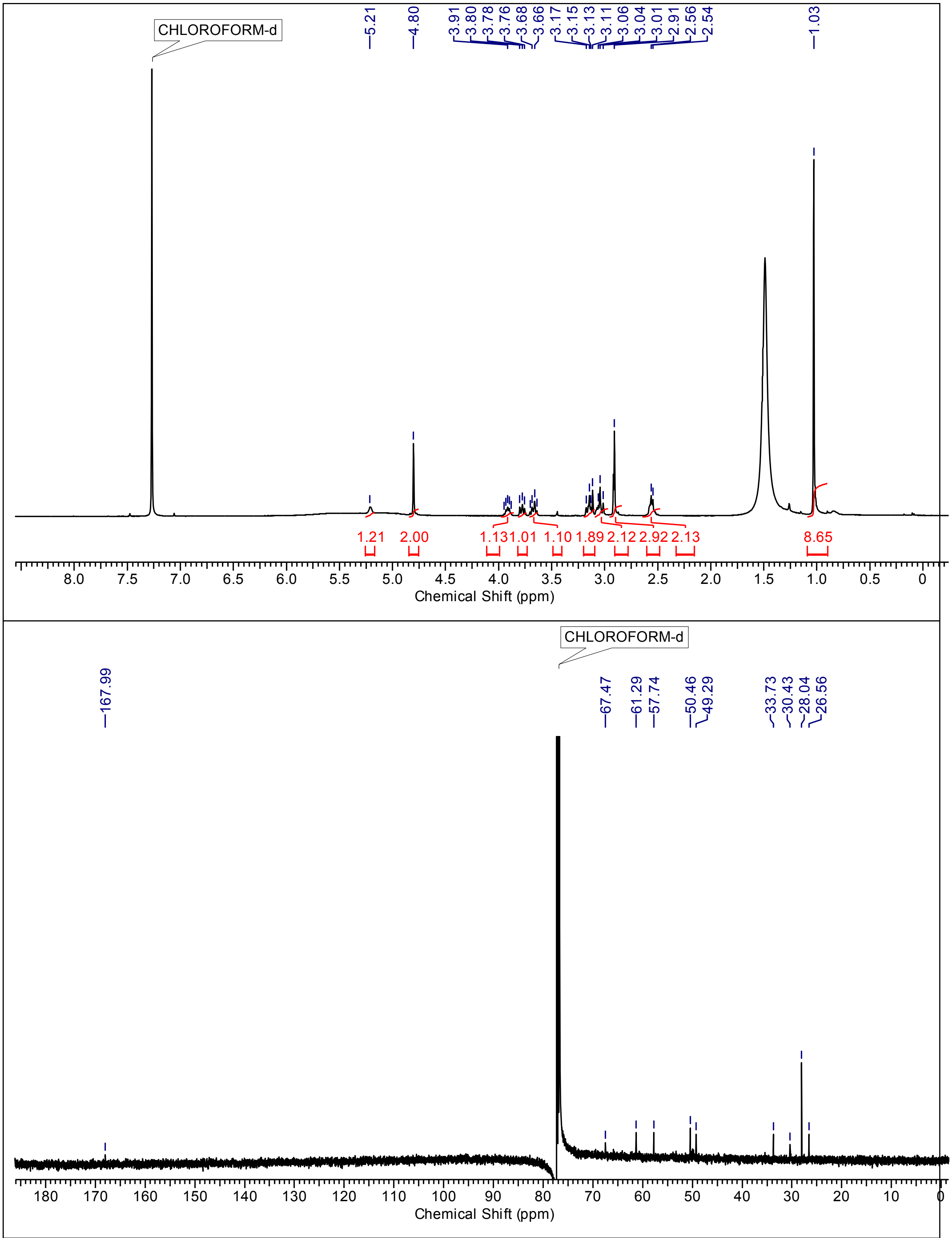

^{1}H and ^{13}C NMR spectra of **4b**

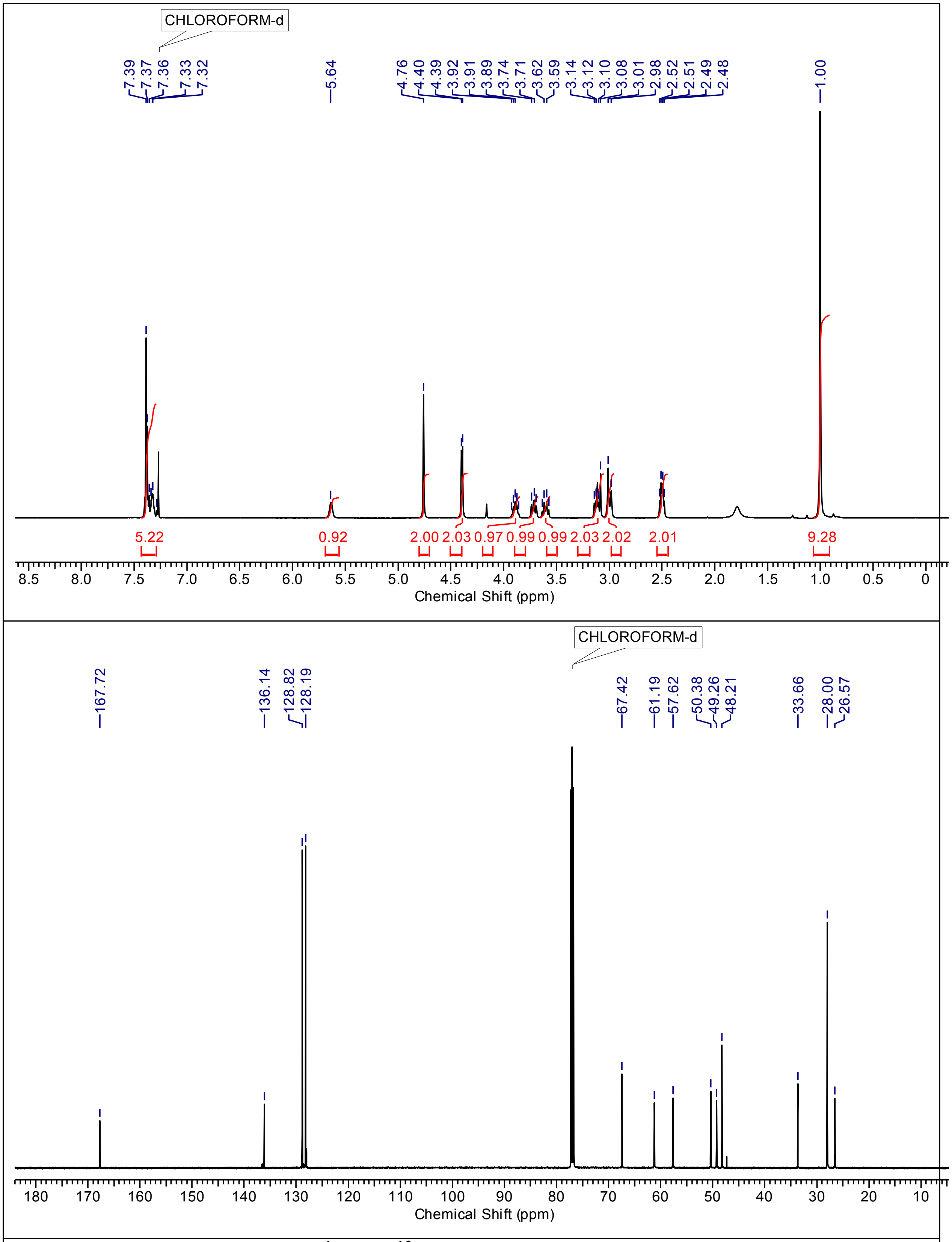

^{1}H and ^{13}C NMR spectra of **4c**

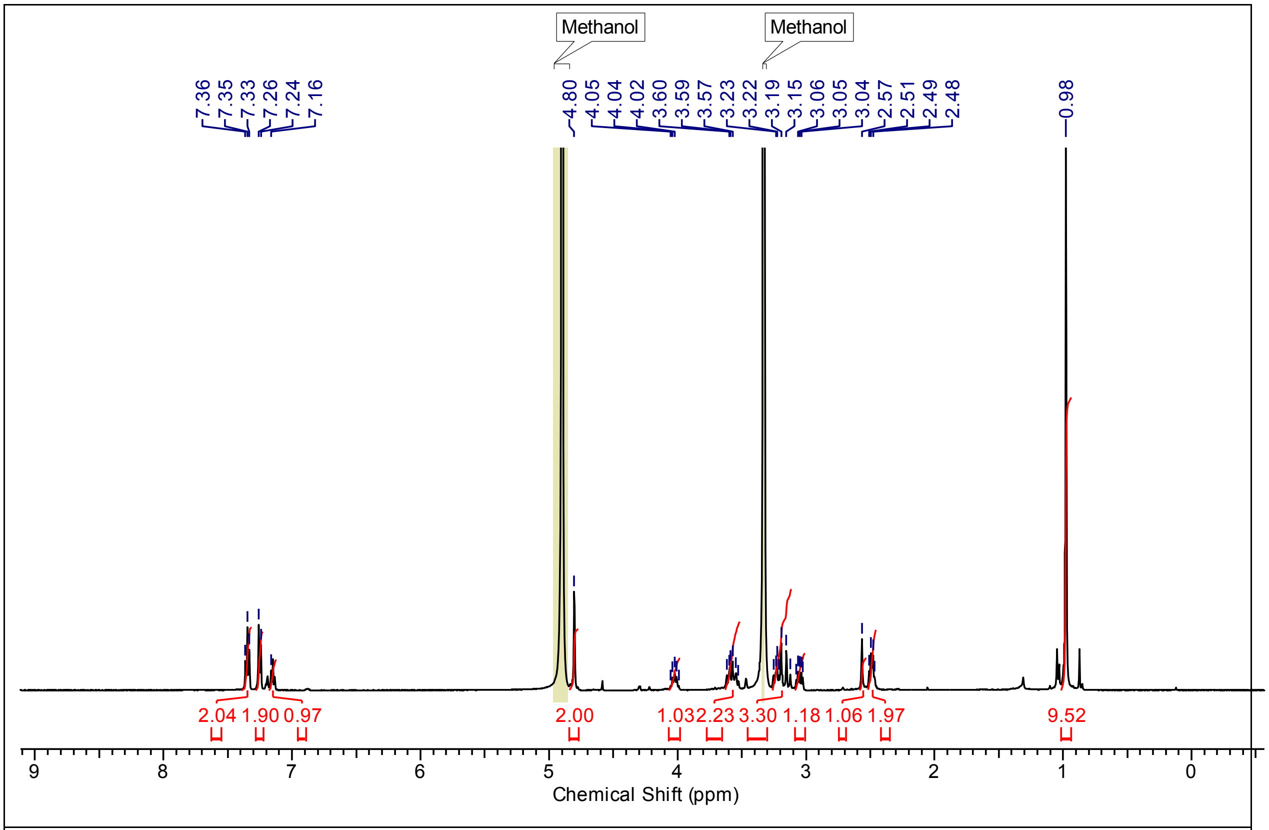

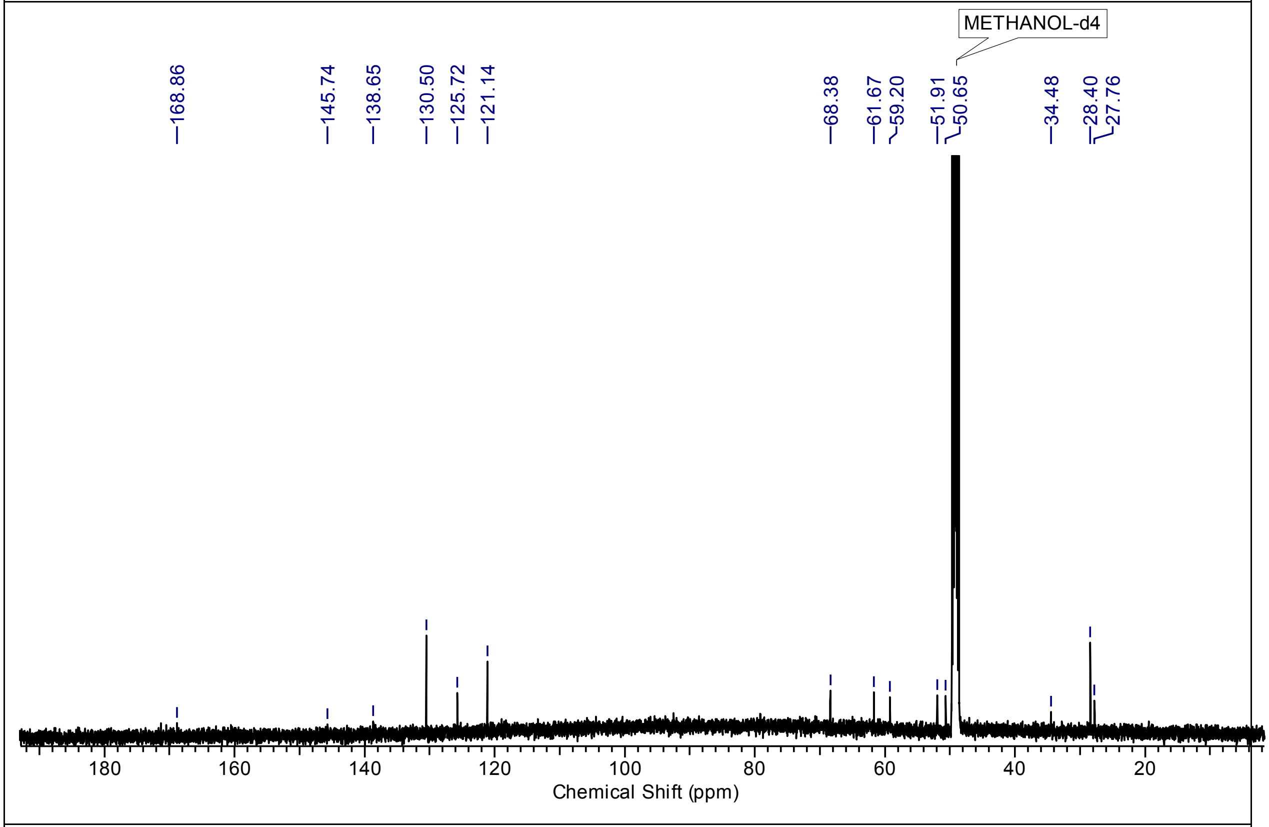

^{1}H and ^{13}C NMR spectra of **4d**

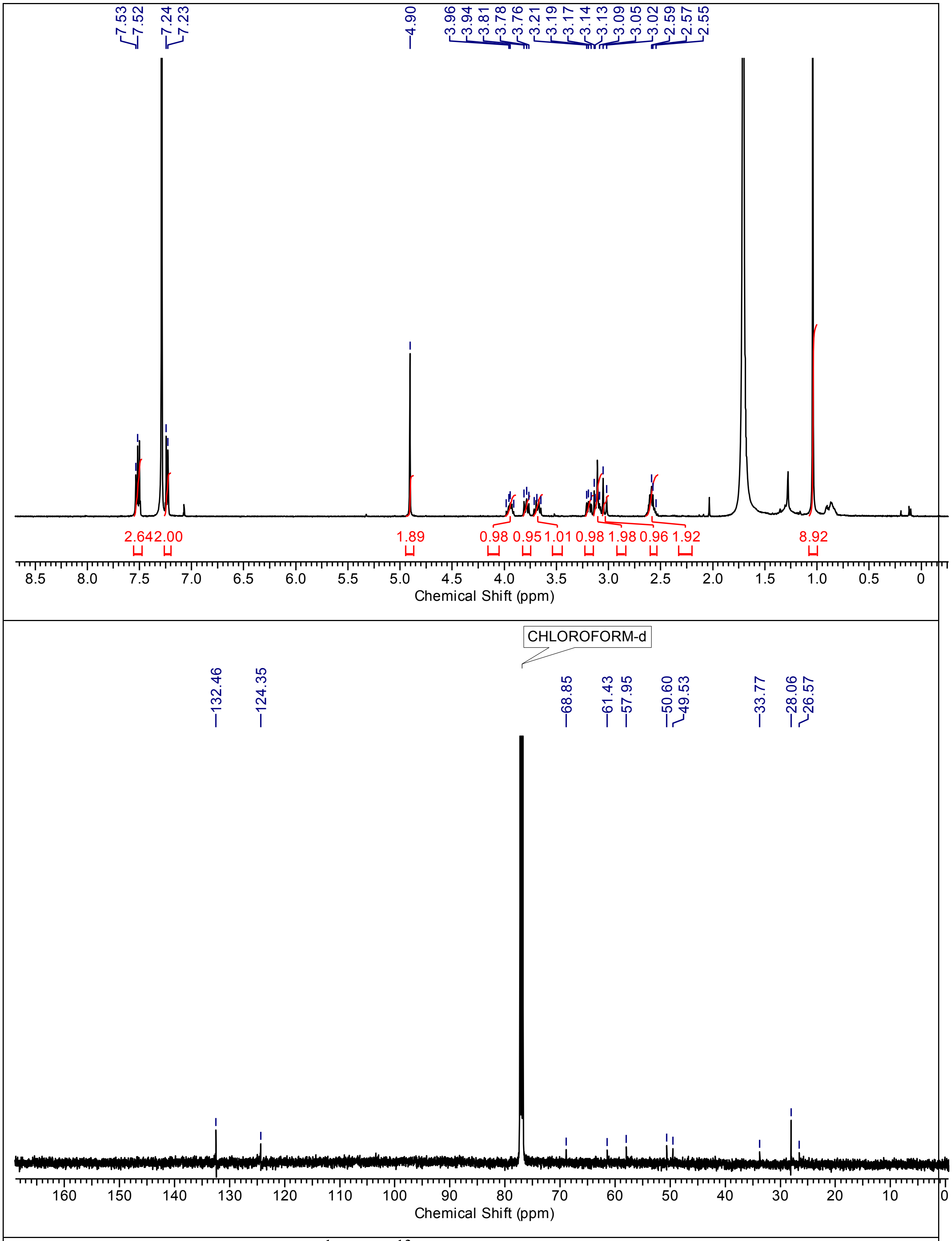

^{1}H and ^{13}C NMR spectra of **4e**

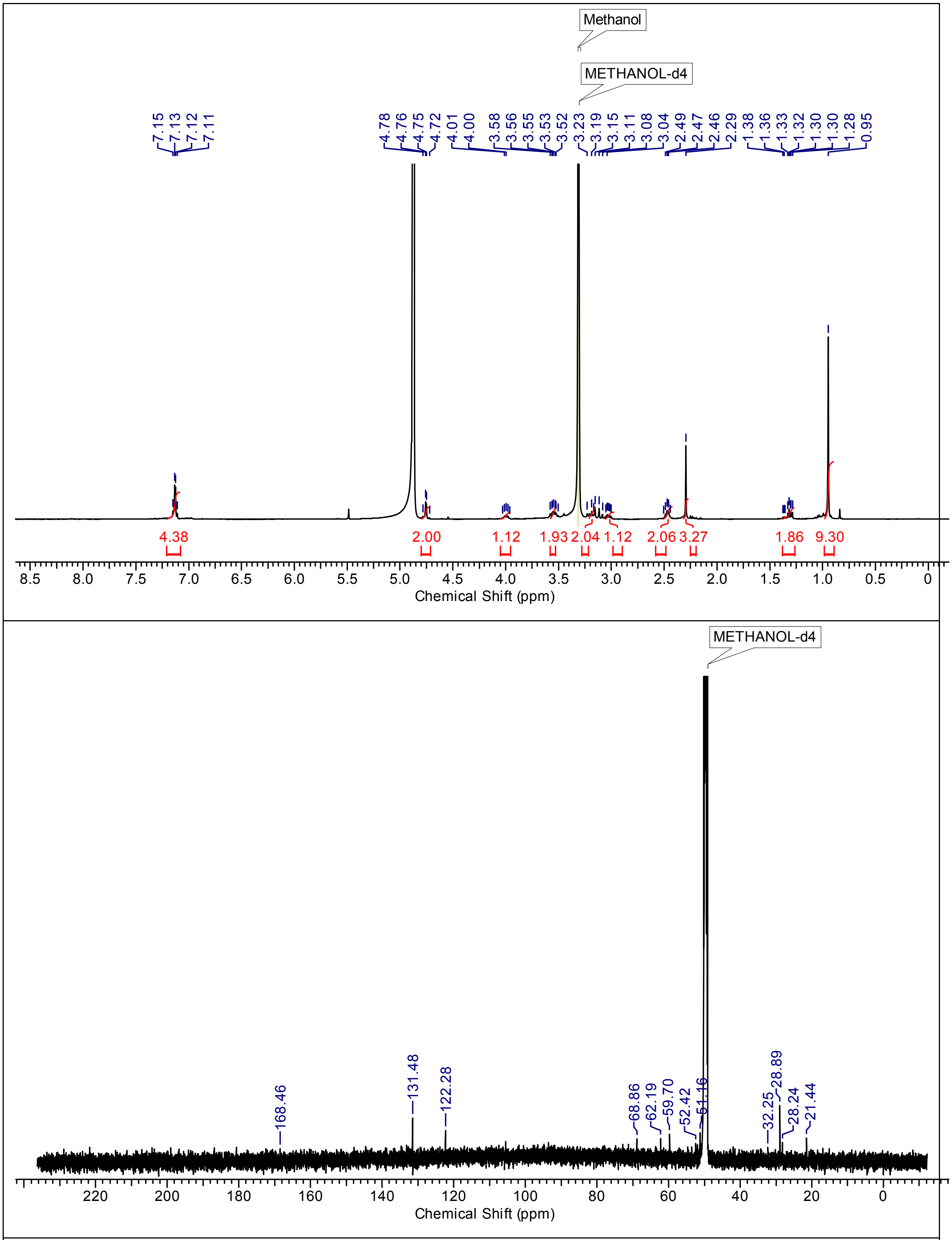

^{1}H and ^{13}C NMR spectra of **4f**

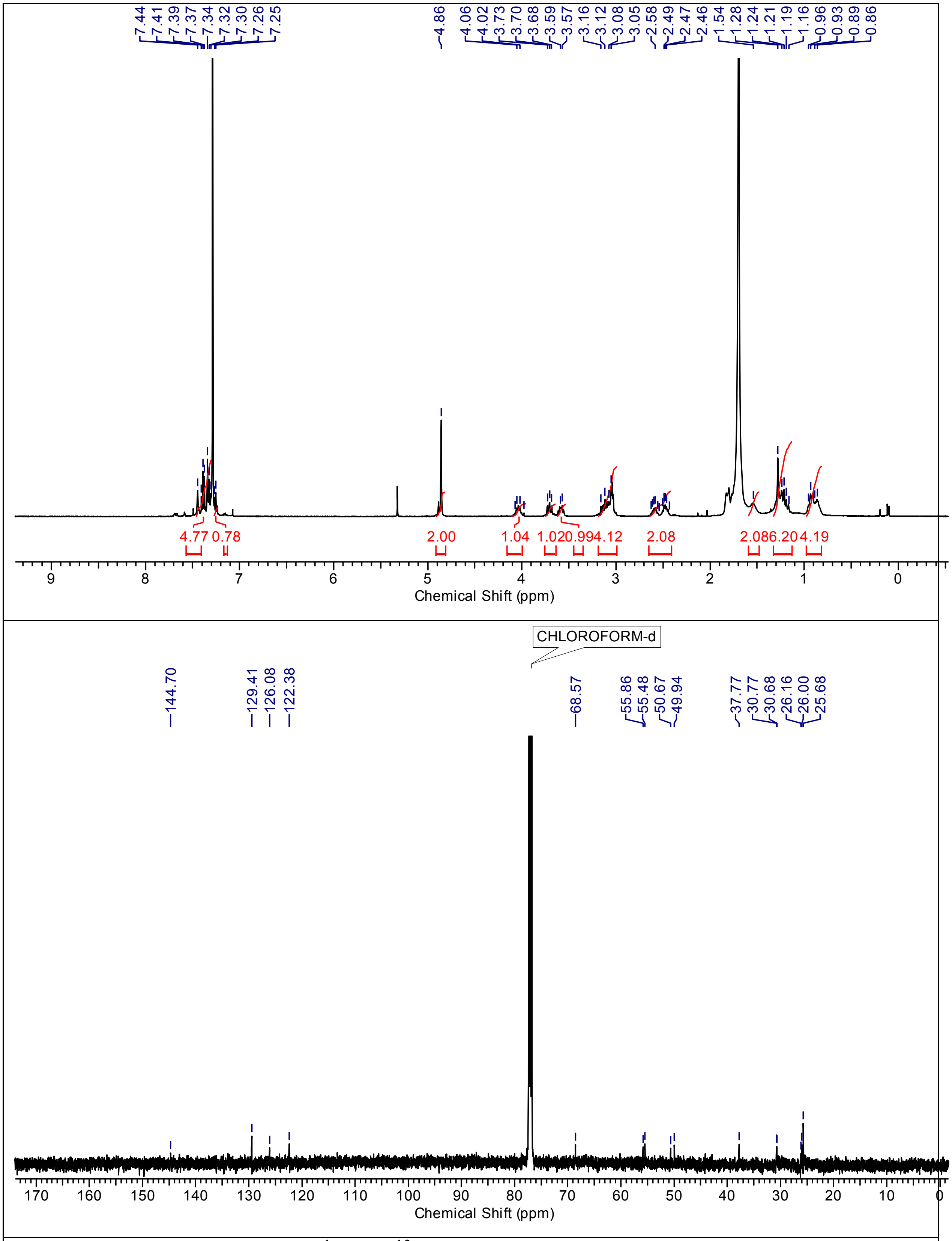

^1H and ^{13}C NMR spectra of **4g**

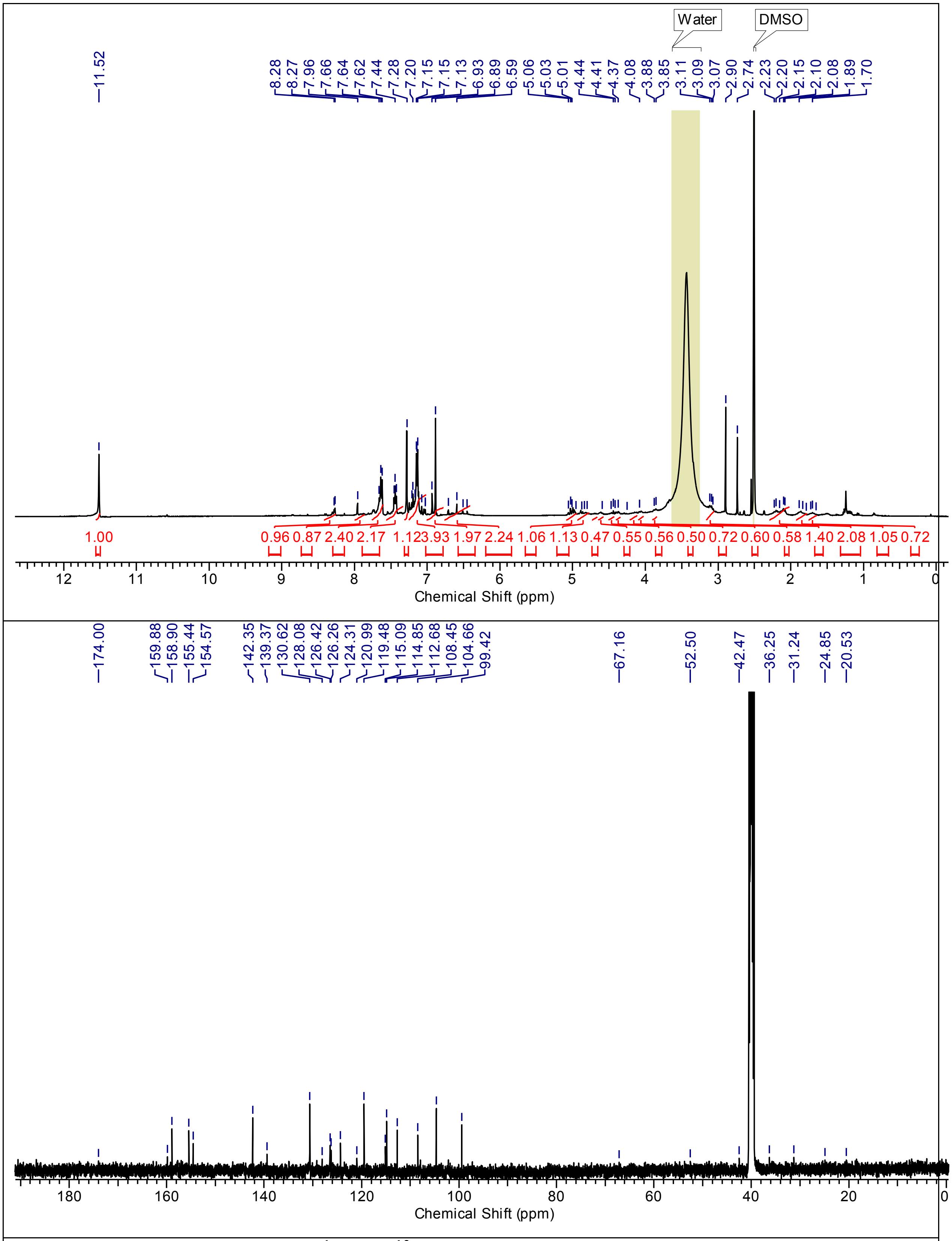

^{1}H and ^{13}C NMR spectra of **3h**

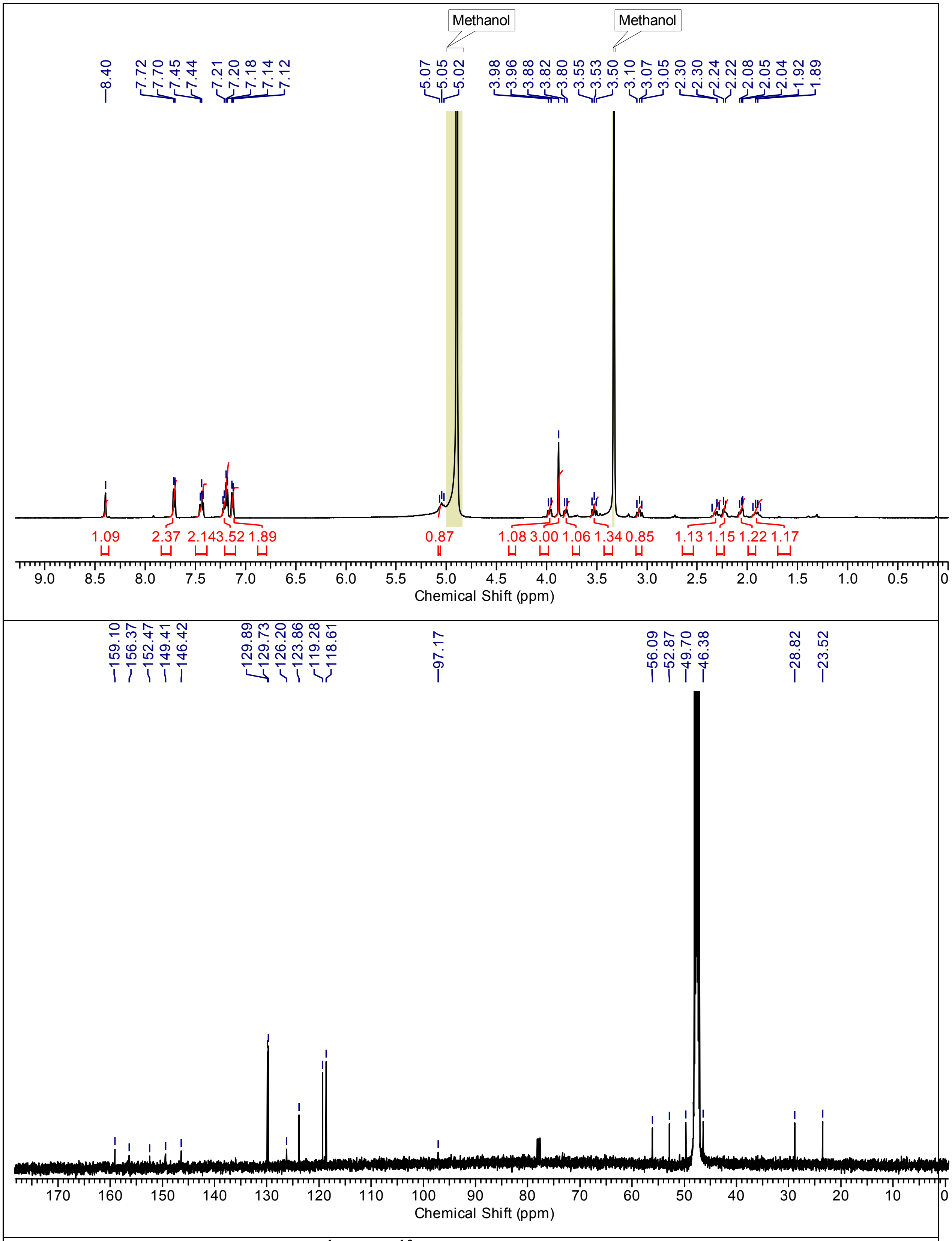

^{1}H and ^{13}C NMR spectra of **3i**

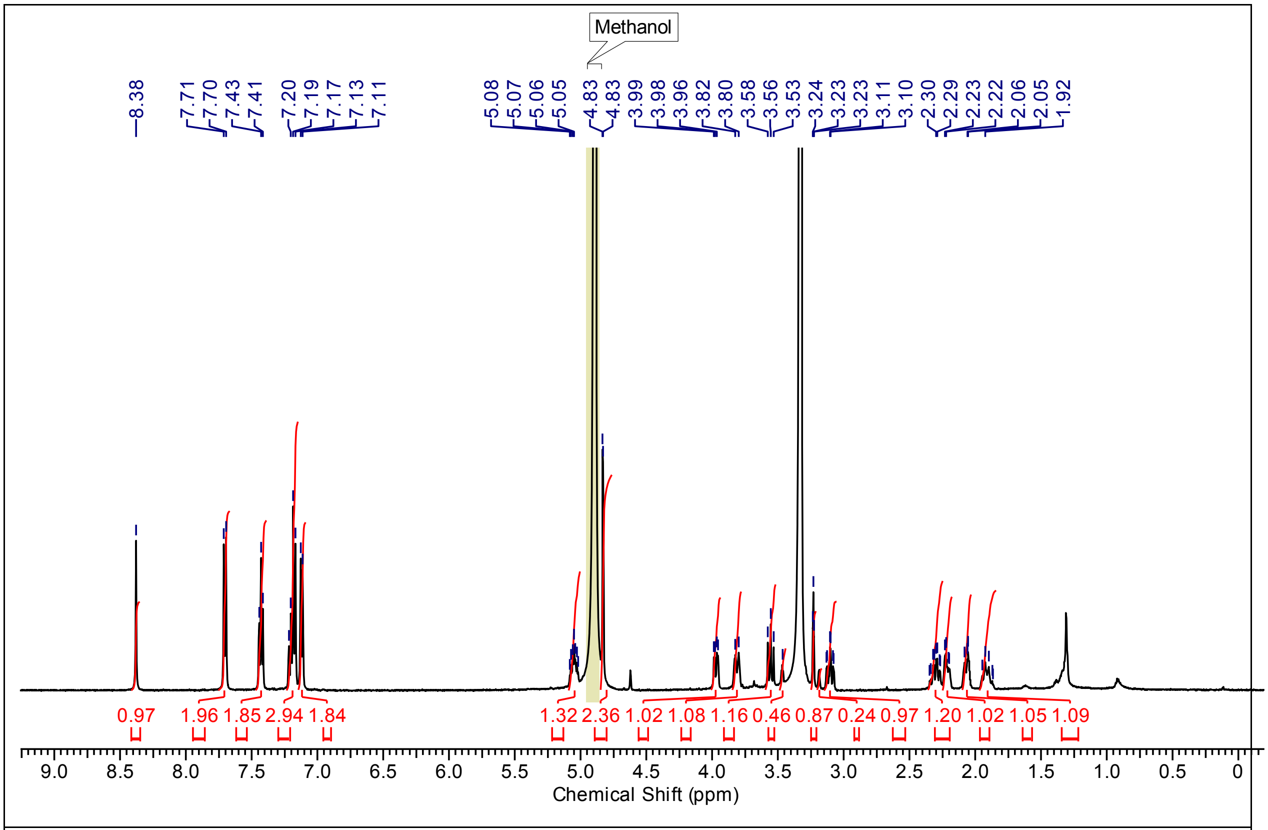

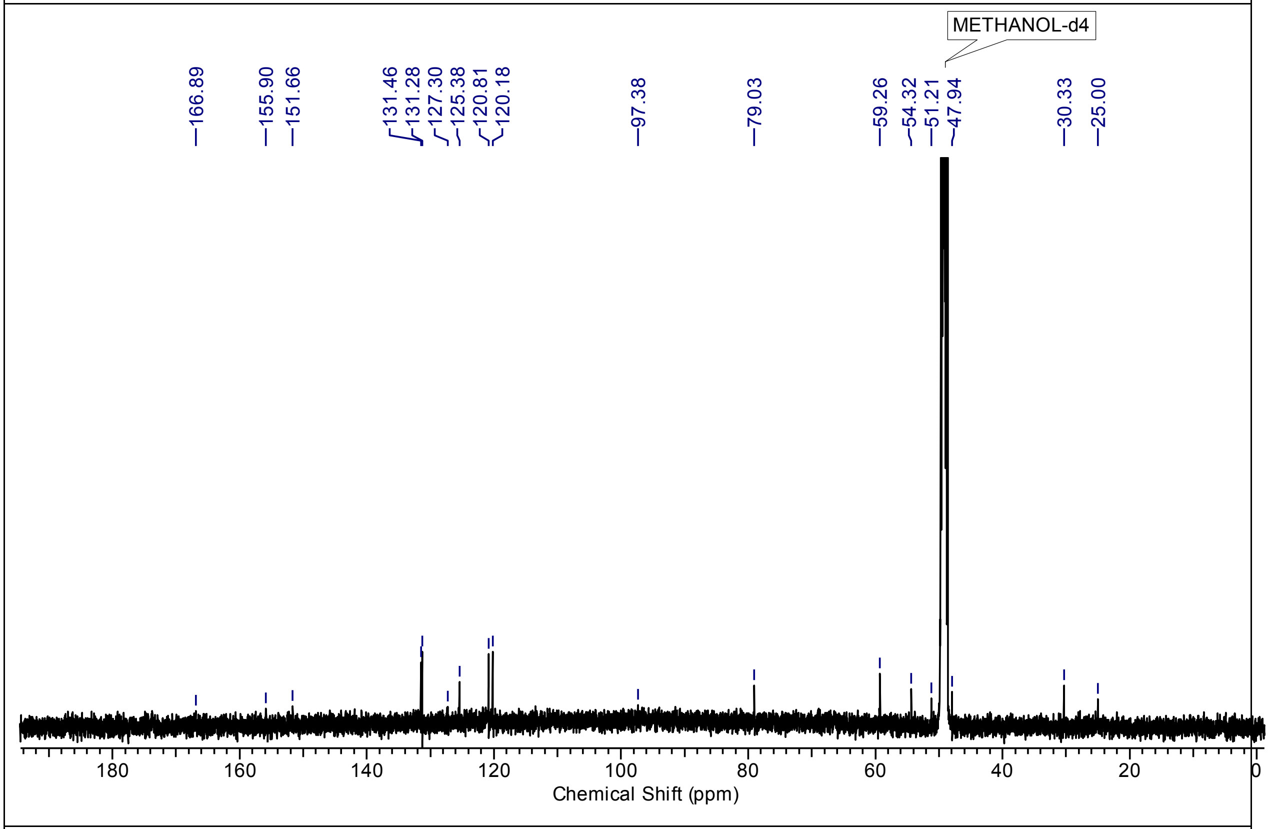

^{1}H and ^{13}C NMR spectra of **3j**
